# Barriers and Facilitators to the Uptake and Maintenance of Healthy Behaviours by People at Mid-Life: A Rapid Systematic Review

**DOI:** 10.1371/journal.pone.0145074

**Published:** 2016-01-27

**Authors:** Sarah Kelly, Steven Martin, Isla Kuhn, Andy Cowan, Carol Brayne, Louise Lafortune

**Affiliations:** 1 Institute of Public Health, Forvie Site, University of Cambridge School of Clinical Medicine, Box 113 Cambridge Biomedical Campus, Cambridge CB2 0SR, United Kingdom; 2 University of Cambridge Medical Library, University of Cambridge School of Clinical Medicine, Box 111 Cambridge Biomedical Campus, Cambridge CB2 0SP, United Kingdom; National Institute for Viral Disease Control and Prevention, CDC, China, CHINA

## Abstract

**Background:**

With an ageing population, there is an increasing societal impact of ill health in later life. People who adopt healthy behaviours are more likely to age successfully. To engage people in health promotion initiatives in mid-life, a good understanding is needed of why people do not undertake healthy behaviours or engage in unhealthy ones.

**Methods:**

Searches were conducted to identify systematic reviews and qualitative or longitudinal cohort studies that reported mid-life barriers and facilitators to healthy behaviours. Mid-life ranged from 40 to 64 years, but younger adults in disadvantaged or minority groups were also eligible to reflect potential earlier disease onset. Two reviewers independently conducted reference screening and study inclusion. Included studies were assessed for quality. Barriers and facilitators were identified and synthesised into broader themes to allow comparisons across behavioural risks.

**Findings:**

From 16,426 titles reviewed, 28 qualitative studies, 11 longitudinal cohort studies and 46 systematic reviews were included. Evidence was found relating to uptake and maintenance of physical activity, diet and eating behaviours, smoking, alcohol, eye care, and other health promoting behaviours and grouped into six themes: health and quality of life, sociocultural factors, the physical environment, access, psychological factors, evidence relating to health inequalities. Most of the available evidence was from developed countries. Barriers that recur across different health behaviours include lack of time (due to family, household and occupational responsibilities), access issues (to transport, facilities and resources), financial costs, entrenched attitudes and behaviours, restrictions in the physical environment, low socioeconomic status, lack of knowledge. Facilitators include a focus on enjoyment, health benefits including healthy ageing, social support, clear messages, and integration of behaviours into lifestyle. Specific issues relating to population and culture were identified relating to health inequalities.

**Conclusions:**

The barriers and facilitators identified can inform the design of tailored interventions for people in mid-life.

## Introduction

With an ageing population, there is an increasing economic, societal and health and social care impact of dementia, disability, frailty and non-communicable chronic diseases (NCDs) in later life. Ill health in later life is heavily influenced by behaviours across the life course, which in turn are influenced by a variety of wider contextual social, economic, and organisational factors [1,2]. People who adopt healthy behaviours are more likely to age successfully and have improved quality of life [3–5].

As they age, those people now in mid-life have a greater risk of development of disease and frailty than younger people in the next decades [6–8]. There is also considerable potential for inequalities in health promoting behaviours and health outcomes, arising from poverty, social and environmental factors [9,10], so risk factors for ill health in later life may manifest at a younger age in disadvantaged, minority or harder to reach groups. To minimise the risk of ill health in later life in these populations, effective ways to change people’s behaviours are needed. In order to engage people in health promotion initiatives in mid-life and inform the design of effective interventions that consider their specific circumstances, a good understanding is needed of why people do not undertake healthy behaviours or engage in unhealthy ones.

This systematic review was one of a series of reviews conducted to inform the development of UK national public health guidance on mid-life approaches to prevent dementia, disability and frailty (DDF) in later life (https://www.nice.org.uk/guidance/ng16). The aim of the review was 1) to identify key issues (barriers and facilitators) for people in mid-life that prevent or limit or which help or motivate them to take up and maintain healthy behaviours that may ultimately impact on healthy ageing including prevention or delay of dementia, disability, frailty or NCDs, and 2) to identify any specific issues that may influence health inequalities, for example by ethnicity, socioeconomic status, gender or in minority or harder to reach groups. Clearly, the goal was not to summarise the whole of the public health literature on barriers and facilitators to behaviour change. Rather, we aimed to identify evidence targeted at or of particular relevance to people at mid-life. People in this segment of the population are likely to share some of the same issues and challenges when it comes to changing or maintaining behaviours. With a similar focus, the other two reviews in the series looked at the association between mid-life risk factors and late life outcomes, and at the effectiveness of mid-life interventions on behavioural risks and late life outcomes.

## Methods

The review was conducted as a rapid systematic review to provide best available evidence within limited timescales. The scope of the review was defined by the funders (National Institute for Health and Care Excellence—NICE), after open consultation with stakeholders and the protocol (available on request) was agreed prior to the start of work. Established systematic review methods of NICE [11] were broadly followed, except as described below.

Searches: The following electronic sources were searched for peer-reviewed studies published in the English language (to March 2014): MEDLINE; EMBASE; PsycINFO; CINAHL; HMIC; Cochrane Central Register of Controlled Trials; Cochrane Database of Systematic reviews; Database of Abstracts of Reviews of Effectiveness; HTA database; NHS EED database; Web of Science, Applied Social Sciences Index and Abstracts and relevant websites (S1 Text). Time constraints precluded hand searches or contact of authors for additional data.

Searching was conducted 1) for systematic reviews using a search filter (SIGN filter [12]) for a broad range of health behaviours, 2) for primary studies for a broad range of health behaviours specifically at mid-life, and 3) specific targeted searches for studies where there were gaps in the evidence, relating to vision, hearing and inequalities. Due to an initial large number of search hits, searches were limited by the use of mid-life terms and indexing (S2 Text). Studies from any country, published in English, from year 2000 onwards were eligible.

### Inclusion and exclusion criteria

Populations: The populations covered by this review included 1) mid-life adults (aged 40–64 years), including those at increased risk of disability, dementia, frailty, or other NCDs or 2) adults aged 39 and younger in populations at higher risk of health inequalities, which refer to people from disadvantaged and minority groups. These groups include (but are not limited to) people of low socioeconomic status, ethnic minority groups, lesbian, gay, bisexual and transgender (LGBT) groups; travellers, and other groups with protected characteristics under the equality and diversity legislation.

Outcomes: Barriers and facilitators which prevent or limit or which help and motivate people to take up and maintain healthy behaviour, including (but not limited to): 1) physical activity or inactivity; diet and nutrition; weight loss (in overweight people) or control; smoking or tobacco consumption; alcohol consumption; multiple behavioural risk factors; healthy behaviours in general, social activity or prevention of loneliness; prevention of sight or hearing loss 2) behaviour at individual, group, family, community level in any setting. Barriers and facilitators relating to participation in or effectiveness of specific interventions are not included.

Study design: Systematic reviews, primary qualitative studies and primary longitudinal cohort studies were considered for inclusion in this review. As few reviews focused on mid-life, systematic reviews in adults in general were included.

Exclusions: Studies in populations with: existing dementia or cognitive impairment, disability, frailty and NCDs, including obesity or their diagnosis, care and management. Barriers and facilitators to: use of medications; recreational drugs; supplements; national policies, legislation or implementation. Cross-sectional studies, systematic reviews that only included cross-sectional studies, abstracts, letters, editorials and unpublished theses were excluded.

**Fig 1:**
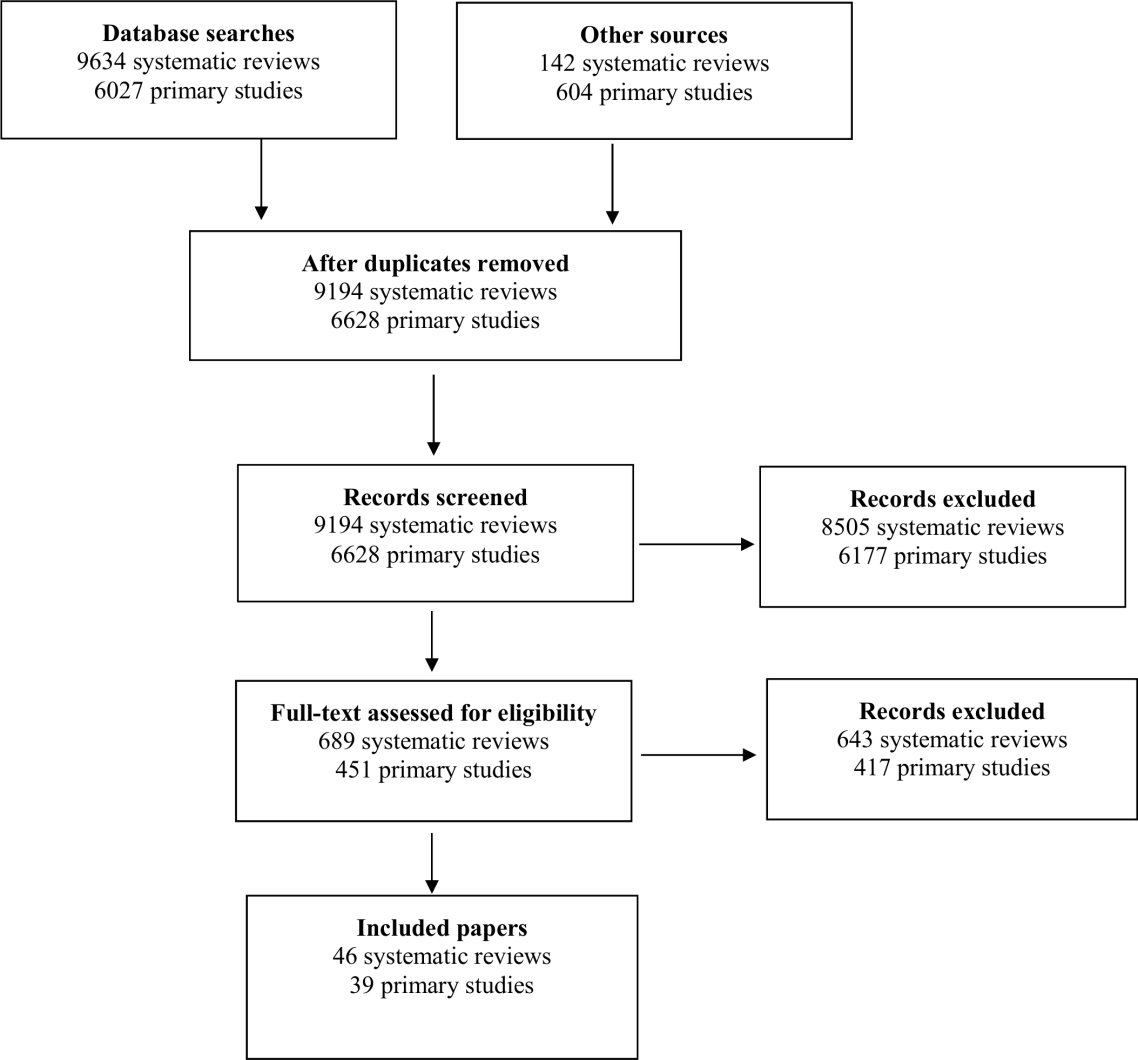
Flow Chart of Searches for Systematic Reviews and Primary Studies.

Identification of relevant studies: Titles and abstracts were screened, and then checked, independently by two reviewers. Differences were resolved by discussion or with a third reviewer. Fig 1 illustrates the flow chart for the study selection process. Studies excluded at the full paper screening stage are reported in S1 Table along with reason for exclusion.

Assessment of methodological quality: Systematic reviews were assessed using AMSTAR [13]. Primary studies were assessed using NICE checklists [14,15] and rated as ++, + or–based on the checklist criteria (Table 1; detailed assessments are in S2 Table). A minimum of 10% of the studies were assessed by two reviewers and discrepancies resolved by discussion. No studies were excluded on the basis of methodological quality.

**Table 1:** Overview of Included Studies.

First Author, Year	Location	Aims	Included population	Quality ^1^
***Physical Activity***				
Systematic Reviews				
Amireault 2013	International	Psychosocial and socio-demographic determinants of physical activity maintenance	Mid-life (18–64 yrs)	+
Babakus 2012	Can, UK, US, Australia	Physical activity and sedentary time among South Asian women	Adults (16–90+ yrs). Ethnic group	++
Beenackers 2012	Europe	Socioeconomic inequalities in occupational, leisure-time, and transport related physical activity among European adults	Mid-life (18–65 yrs)	-
Daniel 2011	International	Correlates of physical activity among South Asian Indian immigrants	Adults (17–91 yrs). Ethnic group	-
Engberg 2012	Can, UK, US, Australia	Life events and change in leisure time physical activity	Adults (17–83 yrs)	-
Fischbacher 2004	UK	Levels of physical activity in South Asian population in the UK	Adults. Children	+
Eyler 2002	US	Correlates of physical activity among women from diverse racial/ethnic groups	Women (age not specified). Ethnic group	-
Fransson 2012	Europe	Job strain as a risk factor for leisure-time physical inactivity	Adults (mean 43.5 yrs)	-
Gidlow 2005	UK	Attendance of exercise referral schemes in the UK	Adults (> 18 yrs)	-
Gidlow 2006	International	Relationship between socio-economic position and physical activity	Adults (18–89 yrs). Socioeconomic	+
Kirk 2011	International	Occupation correlates of adults’ participation in leisure-time physical activity	Adults (18–64 yrs). Occupation	+
Lewis 2002	Not reported	Psychosocial mediators of physical activity behaviour among adults (and children)	Adults (> 18 yrs)	-
Pavey 2012	UK, others not reported	Levels and predictors of exercise referral scheme uptake and adherence	Middle aged (mean 51–64 yrs)	-
Rhodes 2013	International	Moderators of the intention-behaviour relationship in the physical activity domain	Adults (> 18 yrs)	+
Rhodes 2012	International	Factors linked to adult sedentary behaviour	Adults (18–91 yrs)	+
Siddiqi 2011	USA	Understanding impediments and enablers to physical activity among African American adults	Adults (18–89 yrs). Ethnic group	+
Trost 2002	Not reported	Correlates of adults’ participation in physical activity	Adults (age not specified)	-
Vrazel 2008	USA, Latin America	Framework of social-environmental influences on the physical-activity behaviour of women	Women (20–60 yrs)	-
Wendell-Vos 2007	International	Potential environmental determinants of physical activity in adults	Adults (> 18 yrs)	-
Cohort Studies				
Segar 2008	US	To investigate the effects of physical activity goals on physical activity participation	Mid-life. Women	+
Sorensen 2005	Finland	Correlates of physical activity among middle-aged Finnish male police officers	Mid-life. Male police officers	+
Wurm 2010	Germany	Study the effect of a positive view on aging on physical exercise among middle-aged and older adults	Mid-life. Old age	+
Qualitative Studies				
Berg 2002	US	Physical activity perspectives of Mexican American and Anglo American midlife women (focus groups)	Mid-life. Women. Ethnic group	+
Caperchione 2012	Australia	Understanding the challenges and motivations to physical activity participation and healthy eating in middle-aged Australian men (focus groups)	Mid-life. Men	+
DH 2010	England	Insight research conducted in middle-aged adults to inform the Change4Life campaign (a national marketing programme which aims to help people in England change their dietary and physical behaviours) (focus groups)	Mid-life	-
Hooker 2011	US	Factors related to physical activity and recommended intervention strategies as told by midlife and older African American men (interviews)	Mid-life. Old age. Men. Ethnic group	+
Hooker 2012	US	The potential influence of masculine identity on health-improving behaviour in mid-life and older African American men (interviews)	Mid-life. Old age. Men. Ethnic group	+
Im 2013	US	Exploring midlife women’s attitudes toward physical activity (online forum)	Mid-life. Women	+
Im 2012	US	Asian American midlife women’s attitudes towards physical activity (online forum)	Mid-life. Women. Ethnic group	+
Rimmer 2004	US	Physical activity participation among persons with disabilities (focus groups)	Mid-life. Disabilities	+
Segar 2006	US	To investigate the relationship between midlife women’s physical activity motives and their participation in physical activity (surveys)	Mid-life. Women	+
Vandelanotte 2013	Australia	What kinds of website and mobile phone-delivered physical activity and nutrition interventions do middle-aged men want? (focus groups)	Mid-life. Men. Technology	+
Vaughn, 2009	Latin America	Factors that influence the participation of middle-aged and older Latin-American women in physical activity (participant observation and questionnaire)	Mid-life. Old age. Women. Ethnic group	+
Withall 2010	UK	Who attends physical activity programmes in deprived neighbourhoods (questionnaire)	Adolescent. Adults (74%). Deprived neighbourhoods	++
Yarwood 2005	US	Factors influencing ability of midlife women to maintain physical activity over time (interviews)	Mid-life. Women	+
***Diet***				
Systematic Reviews				
Bisogni 2012	Not reported	How people interpret healthy eating	Adults (age not specified)	-
De Irala-Estevez 2000	Europe	Socio-economic differences in food habits in Europe: consumption of fruit and vegetables	Adults (18–85 yrs). Socioeconomic inequalities	-
Fleischhacker 2011	International	Fast food access studies	Children and adults (age not specified)	-
Guillaumie 2010	USA, Netherlands, Great-Britain	Psychosocial determinants of fruit and vegetable intake in adult population	Adults (18–65 yrs)	-
Kamphuis 2006	International	Environmental determinants of fruit and vegetable consumption among adults	Adults (18–60 yrs). Environment	+
Lachat 2012	International	Eating out of home and its association with dietary intake	Adults and children (5–74 yrs)	+
Power 2005	Canada	Determinants of healthy eating among low-income Canadians	Adults (age not specified). Socioeconomic	-
Cohort Studies				
Yates 2012	US	To examine predictors of change over time in healthy eating behaviours in mid-life and older women in response to a one year health-promoting intervention	Mid-life. Old age. Women	+
Mejean 2011	France	To determine sociodemographic, lifestyle and health characteristics associated with consumption of fatty-sweetened and fatty-salted foods in middle-aged French adults	Mid-life	+
Teixera 2010	Portugal	Weight loss readiness in middle-aged women: Psychosocial predictors of success for behavioural weight reduction	Mid-life. Women	+
Qualitative Studies				
Brown 2012	US	To determine the perception of women of the relationship between recent life events, transitions and diet in midlife (focus groups)	Mid-life. Women	+
Caperchione 2012	Australia	Understanding the challenges and motivations to physical activity participation and healthy eating in middle-aged Australian men (focus groups)	Mid-life. Men	+
DH 2010	England	Insight research conducted in middle-aged adults to inform the Change4Life campaign (a national marketing programme which aims to help people in England change their dietary and physical behaviours) (focus groups)	Mid-life	-
Hammond 2010	US	To determine the perception of women of the relationship between recent life events, transitions and diet in midlife (focus groups)	Mid-life. Women	+
Jilcott 2009	US	Perceptions of the community food environment and related influences on food choice among midlife women residing in rural and urban areas (interviews).	Mid-life. Women. Rural/urban settings	++
Vandelanotte 2013	Australia	What kinds of website and mobile phone-delivered physical activity and nutrition interventions do middle-aged men want? (focus groups)	Mid-life. Men. Technology	+
Vue 2008	US	Need states based on eating occasions experienced by midlife women (focus groups)	Mid-life. Women	+
***Overweight***				
Systematic Reviews				
Giskes 2011	International	Environmental factors and obesogenic dietary intakes among adults	Adults (> 18yrs)	-
Giskes 2010	Europe	Socioeconomic inequalities in dietary intakes associated with weight gain and overweight/obesity conducted among European adults	Adults (>18 yrs)	+
Lovasi 2009	US	Built environments and obesity in disadvantaged populations	Adults and children (age not specified). Disadvantaged communities	-
***Smoking and Smokeless Tobacco***				
Systematic Reviews				
Bader 2007	International & Canada	Smoking cessation among employed and unemployed young adults	Young adults (18–24 yrs). Unemployed	**-**
Kakde 2012	India, Pakistan, Nepal, Bangladesh, UK	Social context of smokeless tobacco use in the South Asian population	Adults and children (8–96 yrs). Ethnic group	**-**
Niederdeppe 2008	Not specified	Media campaigns to promote smoking cessation among socioeconomically disadvantaged populations	Adults (> 18yrs). Socioeconomic status	**-**
Vangeli 2011	International	Predictors of attempts to stop smoking and their success in adult general population samples	Adults (> 18yrs)	**-**
Cohort Studies				
Honjo 2010	Japan	To determine predictive factors for smoking cessation among middle-aged Japanese	Mid-life	+
***Alcohol***				
Systematic Reviews				
Brienza 2002	International	Alcohol use disorders in primary care: do gender-specific differences exist?	Adults (age not specified). Women	-
Bryden 2012	International	Influence on alcohol use of community level availability and marketing of alcohol	Adults and adolescents (age not specified). Community factors	+
Bryden 2013	International	Influence of community level social factors on alcohol use	Adults and adolescents (15–59 yrs). Community factors	+
Cohort Studies				
Caldwell 2008	UK	Lifecourse socioeconomic predictors of midlife drinking patterns, problems and abstention	Mid-life	++
Qualitative studies				
Pettinato 2008	US	Life experience of the misuse of alcohol among midlife and older lesbians (interviews)	Mid-life. Old age. Women. Lesbian	+
***Cardiovascular Health***				
Systematic Reviews				
Bock 2012	UK, US, Can, NZ	Practices and factors associated with behavioural counselling for cardiovascular disease prevention in primary care settings	Adults (mean 41 yrs range 34–45 yrs)	-
Hart 2005	US	Women’s perceptions of coronary heart disease	Adults (> 40 yrs). Women	-
Kurian 2006	US	Racial and ethnic differences in cardiovascular disease risk factors	Adults (> 18 yrs). Ethnic groups	-
Murray 2012	International	Patient reported factors associated with uptake and completion of cardiovascular lifestyle behaviour change	Adults. (> 18 yrs)	-
Qualitative Studies				
Folta 2008	US	Factors related to cardiovascular disease risk reduction in midlife and older women (focus groups)	Mid-life. Old age. Women	+
***Health Promoting Behaviour***				
Systematic Reviews				
Bécares 2012	International	Ethnic density effects on physical morbidity, mortality, and health behaviours	Adults (> 18 yrs). Ethnicity	-
Coles 2012	Developed industrialized countries	Community-based health and health promotion for homeless people	Adults (16–89 yrs). Homelessness	+
Dryden 2012	Western/developed countries	Existing knowledge about who does and does not attend general health checks	Adults (age not specified). Hard to reach populations	-
Jansen 2012	Germany	The influence of social determinants on the use of prevention and health promotion services	Adults (age not specified). Socioeconomic inequity	-
Ryan 2009	UK	Factors associated with self-care activities among adults in the United Kingdom	Adults (age not specified)	+
Yarcheski 2004	US, England, Can	Predictors of positive health practice	Adults. Adolescents (age not specified)	+
Cohort Studies				
Benzies 2008	Sweden	To measure factors that predict change in health-related behaviours among midlife Swedish women	Mid-life. Women	+
King 2007	US	To determine factors related to adopting a healthy lifestyle in a middle-aged cohort	Mid-life	++
Petersson 2008	Sweden	To determine predictors of successful self-reported lifestyle changes in a defined middle-aged population	Mid-life	+
Shi 2004	Japan	Health values and health information seeking in relation to positive change of health practice among middle-aged urban men	Mid-life. Men. Urban setting	**-**
Qualitative Studies				
Enjezab 2012	Iran	Internal motivations and barriers effective on the healthy lifestyle of middle-aged women: A qualitative approach (interviews).	Mid-life. Women	**+**
DH 2010	England	Insight research conducted in middle-aged adults to inform the Change4Life campaign (a national marketing programme which aims to help people in England change their dietary and physical behaviours) (focus groups)	Mid-life	**-**
Gower 2013	US	Barriers to attending an eye examination after vision screening referral within a vulnerable population (telephone based questionnaires/interviews)	Mid-life (mean age 48). Underserved	**+**
Meadows 2001	Canada	Health promotion and preventive measures: Interpreting messages at midlife (interviews)	Mid-life	**++**
Smith-Dijulio 2010	US	The shaping of midlife women’s views of health and health behaviours (interviews)	Mid-life. Women	**+**

Guide

^1^ Description of overall methodological quality ratings

++ All or most of the checklist criteria have been fulfilled; where they have not been fulfilled the conclusions are very unlikely to alter

+ Some of the checklist criteria have been fulfilled; where they have not been fulfilled or adequately described the conclusions are very unlikely to alter

- Few or no checklist criteria have been fulfilled and the conclusions are very likely to alter

Data extraction and evidence synthesis: Study data was extracted on population, setting, study design, outcomes, method of analysis, results and funding (S3 Table) by one reviewer and checked for accuracy by another. Issues relevant to the uptake and maintenance of health behaviours and their context were identified in the literature and analysed thematically to identify common themes, integrating qualitative and quantitative evidence [16].

## Results

In total 81 studies (46 systematic reviews and 39 primary studies specifically in mid-life) are included in the review. The studies’ characteristics and overall quality ratings are shown in Table 1 (Detailed quality assessments are in S2 Table).

Nineteen systematic reviews focused on physical activity, seven on diet, three on overweight, four on smoking, three on alcohol consumption, four on cardiovascular health, and six on health promoting practices more generally. Most reviews focused on the adult population in general with six examining specifically mid-life populations. Of the 46 reviews, seven focused on ethnic groups, nine related to disadvantaged groups, four focused specifically on women.

All 39 primary studies focused on specifically mid-life healthy behaviours. Of these, 28 were qualitative studies and 11 were longitudinal cohort studies. In the qualitative studies, 15 were in women only and five in men only, four studies were relevant to health inequalities focused on ethnic groups or deprived, hard to reach or minority groups. Of the cohort studies, four studies were exclusively in female cohorts and two in male cohorts.

The findings were organised in three levels (Table 2): 1) by health behaviour: evidence was found for physical activity, diet, smoking, smokeless tobacco, alcohol, eye care, health behaviours in general including cardiovascular prevention 2) under broad themes identified: health and quality of life, sociocultural factors, the physical environment, access to facilities and resources, psychological factors, health inequalities 3) barriers and facilitators identified within each broad theme. Some issues could be both barriers and facilitators depending on the context.

**Table 2:** Barriers and Facilitators to the Uptake and Maintenance of Healthy Behaviours by People in Mid-life.

Health behaviour / Theme	Health and quality of life	Sociocultural factors	Physical environment	Access (to facilities and resources)	Psychological factors	Health inequalities
***Physical Activity***						
**Barriers**	Physical ailments or chronic conditions	Lack of time. Lack of knowledge. Self-consciousness or social concerns (in women). Low socioeconomic status. More time at home	Neighbourhood safety. Driving instead of walking^.^ Weather	Financial costs. Transport. Lack of availability or access to community physical activity programmes or facilities^.^ Programmes delivered by mobile phones/social networking	Lack of motivation. Low self-efficacy. Perception of lack of capability (in women). Entrenched attitudes and behaviours in midlife	**Ethnic minority groups** Language barriers. Cultural barriers. **Gender** Female gender and gender roles. Hair maintenance **People with disabilities** Barriers relating to the built and natural environment. Barriers relating to cost. Equipment related barriers. Information-related barriers. Emotional and psychological barriers. Perceptions and attitudes relating to accessibility and disability. Lack of resources. **Low SES (as a barrier)**
**Facilitators**	Enjoyment. Sense of wellbeing/Quality of life. Prevention of illness/Healthy Ageing. Health benefits in general. Previous experience of ill health. Focus on short term benefits. Weight loss/ body image. Specific tools. Integration of physical activity into lifestyle	Support. Being a good role model (men)	None found	Fast, easy websites	None found	**Ethnic minority groups** Type of activity. Having exercise equipment at home **Gender** Physically active, adult, female role models **People with disabilities** Facilitators relating to the built and natural environment. Facilitators relating to cost. Equipment related facilitators. Information-related facilitators. Emotional and psychological facilitators. Perceptions and attitudes relating to accessibility and disability. Resources
***Diet***						
**Barriers**	Misinterpretation of health messages	Social environment around food. Food environment. Eating out of home. Competing priorities. Lack of time. Low socioeconomic status. Unplanned shopping routines. Alcohol consumption. Co-existence of other unhealthy lifestyle behaviours	None found	Financial costs. Food availability. Programmes delivered by mobile phones/social networking. Low SES groups. Access to supermarkets	Lack of motivation. Identity. Perception of lack of capability. Existing entrenched behaviours around eating	**Low SES groups** Access to supermarkets
**Facilitators**	Clear food choices. Health concerns^.^ Previous experience of ill health^.^ Swapping foods. Weight loss^.^ Specific tools	Support. Social environment around food	None found	Accessibility. Fast, easy websites	Identity	**Disadvantaged groups** Access to supermarkets
***Smoking***						
**Barriers**	None found	Low SES. Higher level of current smoking. Younger age of initiation of smoking	None found	None found	Lack of motivation	**Unemployed young adults** Lack of motivation. **Low SES (as a barrier)**
**Facilitators**	Development of disease (including initiation of prescribed medicine). Participation in other health behaviours (including PA)	For media campaigns in low SES populations. High exposure. Combination with community component. Appropriate media use, language preferences, literacy needs, cultural values	None found	Information	None found	**Low SES populations** (as listed for health and quality of life and sociocultural factors)
***Smokeless Tobacco***						
**Barriers**	Misperception of benefits (some perceived health benefits include relief of abdominal problems, enhanced digestion, stress relief, as an aid to oral hygiene, relaxation and concentration). Limited knowledge of harmful health effects	Cultural and social acceptance (associated with socialising and family tradition)	Easy availability	Low cost. Lack of information and resources to aid quitting	Lack of motivation	**Ethnic minority groups** All data relating to smokeless tobacco was from one systematic review in South Asian populations in UK, India, Pakistan, Nepal.
**Facilitators**	None found	Social, physical and emotional support to quit. Advice from doctors or dentists (but devalued when they were users themselves)	None found	None found	None found	As above
***Alcohol***						
**Barriers**	None found	Socioeconomic status. Neighbourhood disorder and crime	Advertising and media. Availability	None found	None found	**Gender** Female **LGBT groups** Disconnection from identity (lesbian women)
**Facilitators**	None found	None found	None found	None found	None found	None found
***Eye Care***						
**Barriers**	Other medical problems prioritised	Lack of understanding of information (e.g. need for follow up examination)	Could not find transportation	Could not afford transportation. Appointment arrangements (e.g. forgetting, attending but not being seen by the clinician, no clinic contact details or location). Long waits	None found	**Low SES** All data reported for eye care was from a population with little or no health insurance in the US.
**Facilitators**	None found	None found	None found	Appointment arrangements (e.g. appointment reminders, same day appointments, decreased wait times, better information about appointment location and contact details, flexible clinic hours)	None found	As above
***General Health Promoting Behaviours***
**Barriers**	None found	Alcohol consumption. Lack of time	Distance	None found	None found	**Gender** Female **Ethnic minority groups**
**Facilitators**	Health check-ups. Knowledge. Physical activity. Experience or fear of ill health	Marital status. Education. Having a child at home	None found	None found	Self-efficacy	None found

### Physical Activity (PA)

#### Health and wellbeing

Barrier: Existing physical ailments or chronic conditions were a barrier to PA participation in four qualitative studies [17–20], one cohort [21], and three systematic reviews [22–24].

Facilitators: 1) Improved sense of wellbeing, energy, positive feelings or self-esteem. Evidence from six qualitative studies [18–20,25–27] indicates that promotion of ‘feel good’ benefits like greater self-esteem and confidence can be a motivator for behaviour change. A cohort study [28] found that females who focused on a sense of wellbeing and/or stress reduction goals participated in significantly more PA than those who focused on weight loss and/or health benefits.

2) Health benefits in general. Eight qualitative studies [17,18,20,25,27,29,30,31] and a cohort study [28] reported improved health benefits as motivators for participation in PA. Another cohort study [32] found that a high value placed on health was positively associated with change in health practices in men. One systematic review [33] found associations between expected health benefits and PA in repeated studies.

3) Fear of illness or ageing and wanting to promote a healthy old age was reported in seven qualitative studies [17,18,20,25,29,31,34] so that people were able to do the things they wanted, for example, travel, hobbies and caring for families in later life.

4) Weight loss, body image, physical appearance was reported in six qualitative studies [17–19,25,27,35]. However, in cohort studies [28,35], participants with weight loss goals participated in significantly less PA than those with sense of wellbeing or stress reduction goals or body shape, toning or losing weight motives.

5) Enjoyment of the activity. There is consistent evidence from three qualitative studies [18,20,30], one systematic review [33] and one cohort study [33,36] in which ‘enjoyment’ was a powerful determinant of later PA.

6) Previous experience of ill health. Two qualitative studies [19,29] reported ill health (high blood pressure, diabetes, obesity, stroke) as a motivator for PA and one reported a family history of disease was important when considering preventive health care [37]. However, evidence was not always consistent. In cohort studies, elevated blood pressure, risk factors for cardiovascular disease or myocardial infarction were associated with positive lifestyle changes in men [38] but a history of hypertension or diabetes was not [21].

7) Integration of PA into lifestyle. In qualitative studies [27] key messages related to positive swapping of behaviours; for example, walking as an easily integrated part of everyday activity to replace sedentary travel [27] and strategies for incorporating PA into daily lifestyle [39]; a systematic review in UK South Asian populations found a need for PA to be incorporated into everyday activities [40].

8) Focus on short-term benefits. One qualitative study found the promotion of short-term benefits that are quickly achievable, potentially leads to longer-term benefits such as prevention of heart disease [27].

9) Supplying the tools to make and sustain behaviour changes. In one qualitative study most people felt they lacked specific information and strategies to make changes in their daily lives [27].

#### Sociocultural factors

Barriers: 1) Lack of time. In seven qualitative primary studies, lack of time was raised as a barrier to participation in mid-life activity [17–20,25,29,41] in particular because of conflicting demands of work, childcare, family and household responsibilities in all genders and ethnic groups represented. Additionally, six systematic reviews also raised relevant barriers relating to lack of time [23,24,33,42]; high job strain [43] and having child care responsibilities [22].

2) Self-consciousness or social concerns. Concerns about social discomfort or self-consciousness about participation in PA programmes or in the gym were raised in three qualitative studies [19,39,44], all in women.

3) Lack of knowledge. This was raised in one qualitative study [31] in middle-aged women in Iran and one systematic review in adults in general [24].

Facilitators: 1) Support. Supportive partners, family or friends, having a companion to do PA, or support from a physician were cited as facilitators of PA in three qualitative studies [17,20,30] and three systematic reviews [22,33,42]. Conversely, lack of support was cited as a barrier in four qualitative studies [17,20,29,41].

2) Being a good role model. Two qualitative studies in men [25,34] reported being a good role model for children and others as being a motivator for PA.

Barrier/Facilitator: Life changes/more time at home. In one qualitative study, middle-aged adults reported spending more time in front of the computer or TV once children had moved out [27]. A review [45] found that changes at work were associated with increased PA in middle-aged women.

#### Physical environment

Barriers: 1) Neighbourhood safety. Two qualitative studies [19,29] and three systematic reviews [22–24] reported concerns about unsafe neighbourhoods.

2) Weather. Two qualitative studies in women reported the weather as a barrier to PA participation [19,39].

3) Driving instead of walking. One qualitative study reported tendency to drive instead of walk, with cars seen as a symbol of status and security [27].

4) Lack of recreational space. One systematic review in mid-life populations reported lack of parks or recreational space and traffic as a barrier [24].

#### Access (to facilities and resources)

Barriers: 1) Financial costs. Three qualitative studies [18,20,34] and one systematic review [24] reported that the costs of organised PA or fitness club membership were a barrier to PA.

2) Transport issues. Inconvenience, for example, having to drive to participate in PA, was cited as a barrier in one qualitative study in men [17]. Lack of transport was a barrier to PA in one qualitative study in women [20] and in one systematic review in adults [22].

3) Lack of availability or access to community programmes and facilities. Two qualitative primary studies [17,19] and two systematic reviews highlighted lack of availability or limited places on PA programmes or access to places to do PA including recreational space, gyms [22,24].

4) Programmes delivered by mobile phones/social networking. One qualitative study [46] found interventions delivered via mobile phone was not of interest to most participants; though if they had a smartphone they were more open to the idea. Social media networking was not a high priority in the mid-life population studied due to lack of time.

Facilitator: Fast, easy to use websites. Middle-aged men preferred websites that were fast, easy to use, clutter-free, and concise with reliable information and interactive features that could give feedback, such as podcasts, step-by-step videos and pictures [46].

#### Psychological factors

Barriers: 1) Lack of motivation. This was reported in three qualitative studies [19,25,34] and one systematic review [24].

2) Low self-efficacy. Three systematic reviews in adults found a relationship between self-efficacy (belief in one’s own ability to complete tasks and reach goals) and PA participation [33,47,48]. Two qualitative studies [20,41] in women found perception of lack of capability often prevented PA participation.

3) Entrenched attitudes and behaviours in mid-life. For example when structured PA was not a part of self-identity or had become associated with a fear of being judged for decreasing abilities [27].

Facilitator: Positive view of ageing. One cohort study [49] found that a positive view of ageing was associated with increased sporting activity in those who were healthy enough to take part.

#### Health inequalities

Barriers or facilitators cited previously were also applicable in broader population groups. Only those more specific to disadvantaged and minority groups are discussed here:

#### Socioeconomic status

Barriers: Six systematic reviews [22,33,50,51–53] in adults in general linked higher SES, education or income with higher levels of PA. Lower occupation status was associated with higher total PA in one review [50] and two reviews [52,53] reported a higher level of occupational PA in lower socioeconomic groups; those with higher socioeconomic position were more active during leisure time.

#### Ethnic minority groups

Barriers: 1) Language barriers. Language was highlighted as a barrier to PA participation in two qualitative studies in US women [19,41], one in an Asian population and one in a Latin-American population.

2) Cultural barriers. Cultural beliefs and traditional roles for women that focus on family and domestic duties were cited in US qualitative studies, in Asian [29,41] and Latin American populations [19]. An emphasis of intellectual activity over physical activity in Asian culture was also a barrier [41]. From systematic reviews [23,40], culturally inappropriate facilities included mixed sex swimming pools and male instructors (for women).

Facilitators: 1) Type of activity. Walking was most commonly recommended PA followed by sports-related activities in one qualitative primary study in mid-life African American men [23,34].

2) Having exercise equipment at home. South Asian populations reported this as a facilitator [23].

#### Gender and gender roles

Barriers: 1) Female gender and gender roles. This was highlighted in four qualitative studies [19,29,30,41] and three systematic reviews [23,40,54], including two specifically in South Asian women. One systematic review [54] reported that women were more likely to begin exercise referral schemes but less likely to maintain participation.

2) Hair maintenance. One systematic review found that hair maintenance was perceived as a barrier to PA in African American women [24].

Facilitator: Physically active female role models. One systematic review in adult women in general found [42] that they would feel more confident about adding PA to their lifestyles if they had female role models.

#### People with disabilities

One qualitative study [55] reported detailed barriers and facilitators to PA participation among people with disabilities. Due to space issues only the main themes are reported in Table 2.

### Diet

#### Health and quality of life

Barrier: Misinterpretation of health messages. In a UK qualitative study [27], misinterpretation of food messages like eating ‘five a day’ could mean fruit and vegetables were being added to existing daily food intake.

Facilitators: 1) Clear and simple food advice. One qualitative study about bone health [56] found that women preferred to have clear and simple food choices for overall health.

2) Health concerns. In one qualitative study in US women [56] food choices were affected by personal health concerns.

3) Previous experience of ill health. In one qualitative study [57], a motivating factor for changes in diet relating to bone health was diagnosis of osteoporosis in a family member.

4) Focus on swapping foods. A UK qualitative study [27] reported that strategies for replacing unhealthy snacks with healthy ones, replacing high calorie meal components with lower calorie ones providing new and interesting ways to add fibre to the diet were behaviour change messages that engaged mid-life adults. Reducing alcohol consumption was less motivating due to the pleasure associated with drinking.

5) Weight loss/short term benefits. Promotion of weight loss was a facilitator for dietary change when it was supported by information on other short-term benefits [27].

6) Supplying the tools to make and sustain behaviour changes. Most people felt they were aware of the changes they needed to make but lacked the specific information and strategies to make the changes in their daily lives [27].

#### Sociocultural factors

Barriers: 1) Eating out of home. In one systematic review [58], eating out of home was associated with higher energy and fat intake and lower micronutrient intake.

2) Competing priorities. One systematic review [59] identified competing food choice priorities as a barrier. These included enjoyment, cost, managing relationships and convenience and may be influenced by personal, social and food context.

3) Lack of time. Insufficient time for healthy eating was highlighted in one systematic review [59] often related to family schedules and work demands.

4) Unplanned shopping routines. In a qualitative study [27] it was found that unplanned shopping trips encouraged impulsive and indulgent purchases.

5) Co-existence of other unhealthy lifestyle behaviours. In a qualitative study, [27], alcohol consumption increased the energy of the overall diet and encouraged unhealthy food choices. In one cohort study, drinkers or smokers were more likely to consume higher amounts of fatty, sweetened or salted foods [60].

Facilitator: Support. Family support was a determinant of the uptake and maintenance of healthy eating behaviour in one cohort study [61] and identified as a factor influencing eating behaviour in one systematic review [59].

Barrier/facilitator: Social environment around food. In one qualitative study conducted in women [56] food choices were influenced by family members and co-workers. In one systematic review [59], social relationships and context influenced how people interpret healthy eating.

#### Physical environment

Barrier/facilitator: Food environment. In one qualitative study conducted in US women [56], food chosen at home and at work was influenced by the surrounding food environment including the type of food available and convenience of access. In two systematic reviews [62,63], greater access to supermarkets or less access to takeaway outlets was associated with lower prevalence of overweight and obesity but mixed associations were found with dietary behaviours [63].

#### Access (to facilities and resources)

Barriers: 1) Financial costs. One systematic review [59] found that people reported that healthy foods were too expensive.

2) Interventions delivered by mobile phones/social networking were considered in one primary qualitative study, as above [46].

Barrier/Facilitator: Food accessibility or availability. Widespread availability of unhealthy food such as junk food and lower availability of healthy food was reported in a systematic review [59]. Another review [64] found some limited evidence that fruit and vegetable consumption was higher when more easily available and having your own vegetable garden or a supermarket in the local area.

Facilitator: Fast, easy to use websites. One primary qualitative study [46], as above.

#### Psychological factors

Barriers: 1) Perception of lack of capability/knowledge/motivation. In one systematic review beliefs about capabilities and knowledge and motivation were consistently associated with fruit and vegetable intake [65].

2) Existing entrenched behaviours around eating. In one qualitative study [27] unhealthy behaviours around eating were deeply embedded, such as snacking, bingeing, junk food, skipping meals, oversized portions, or fussy eating habits retained from childhood.

Barrier/facilitator: Identity. One systematic review [59] reported the influence of a person’s self-concept on how they eat, as some people desire to be healthy eaters whereas others viewed it as weird or picky.

#### Health inequalities

Barriers: 1) Socioeconomic status. Lower household income or SES was associated with lower fruit, vegetable or fibre consumption and higher total fat intake from three systematic reviews [62,64,66]. A further review found that fast food restaurants were more prevalent in low-income areas [67].

2) Food environment. In two systematic reviews [63,68] there was some limited data that the food environment (greater access to supermarket or less access to takeaway outlets) was associated with lower prevalence of overweight and obesity in socioeconomically deprived areas and in US black or Hispanic populations.

### Smoking

#### Health and quality of life

Facilitators: 1) Participation in other healthy behaviours. One systematic review [65] and one cohort study [69] found that those who participated in other healthy behaviours were more likely to quit.

2) Initiation of prescribed drug use or development of disease were identified as facilitators in one cohort study [69].

#### Sociocultural factors

Barriers: 1) Higher level of current smoking. In one cohort study [69] quit attempts were less successful in heavier smokers. In one systematic review, higher number of cigarettes smoked was associated with failed quit attempts [70].

2) Younger age of initiation of smoking. One cohort study found that those who started smoking at a younger age were less likely to stop smoking [69].

#### Psychological factors

Facilitator: Motivational factors. In one systematic review [70], motivation to quit and intention to quit were positively associated with making a quit attempt but not with quit attempt success.

No studies identified barriers or facilitators related to the physical environment or access.

#### Health inequalities

Barriers: 1) Low SES. One systematic review found that media campaigns to promote smoking cessation are often less effective in low SES groups [71]. In another review [70], higher social grade was predictive of quit attempt success in two studies. One Japanese cohort study [69] found that white-collar workers were more likely to quit than blue-collar workers.

2) Unemployed young adults. One review reported a lack of literature relating to smoking cessation among unemployed young adults [72].

### Smokeless Tobacco

All data for smokeless tobacco is from one systematic review that examined the cultural and social acceptance of use in South Asian communities including in the UK, India, Pakistan and Nepal [73] and is summarised in Table 1.

### Alcohol

#### Sociocultural factors

Barrier: Neighbourhood disorder and crime. One systematic review [74] found evidence that alcohol use may be higher in communities with greater social disorders.

#### Physical Environment

Barriers: 1) Availability. One systematic review [75] assessed the relationship between alcohol use and availability from commercial sources at the community level. Results were not significant in the included studies in adults overall but higher outlet density, defined as shops, bars and restaurants in a community, may be associated with an increase in alcohol use.

2) Advertising and media. One systematic review [75] assessed the relationship between alcohol use and advertising at the community level. Only one of the included studies for this exposure was conducted in adults (in women) but that study reported a significant relationship between advertising and alcohol use.

No studies were identified which assessed health and quality of life, access (to facilities and resources), or psychological barriers and facilitators.

#### Health inequalities

Barriers: 1) Socioeconomic status. One UK cohort study [76] found that socioeconomic disadvantage was linked to mid-life moderate-binge, non-/occasional and problem drinking but not low-problem heavy drinking. One systematic review [74] found the association between community-level socio-economic factors (deprivation, income, employment) and alcohol use was inconclusive with some indication that alcohol use may be greater in high-income communities but also in communities with higher unemployment levels.

2) Gender. One review [77] concluded that while women with alcohol use disorders are more likely to seek help, they are less likely to be identified by their physicians. Barriers to seeking help include: fear of abandonment by partner, fear of loss of children and financial dependency.

3) Identity. One qualitative study [78] with lesbian women found that the use of alcohol can be associated with a disconnection from an individual’s identity; in particular with their lesbian identity but also from other roles such as student, partner, employee and parent or from childhood issues.

### Eye Care Behaviours

Only one qualitative primary study was found for eye care in mid-life men and women in the US with little or no health insurance and is summarised in Table 2 [79].

### Health Behaviours in General

#### Health and quality of life

Facilitators: 1) Health check-ups. One cohort study [80] reported that those attending health check-ups were more likely to be engaged with healthy behaviours. In a qualitative study [37] most participants reported going for regular, quick annual check-ups; however women often reported that dismissive statements from healthcare professionals sometimes stopped them seeking preventive check-ups.

2) Knowledge of healthy behaviour. One qualitative study [31] found that health related knowledge, was positively related to health-promoting behaviours.

3) Participation in other healthy behaviours. One cohort study [80] found that participating in physical activity had a positive effect on mental health (which is positively associated with health-related behaviours) and in another cohort study [38] activity was a factor for success in lifestyle change. In a systematic review [81] lack of exercise experience was a barrier to adopting health-promoting behaviours. In one cohort study [38] lower alcohol intake was associated with positive lifestyle changes.

4) Experience or fear of ill health. One qualitative study conducted with women in Iran [31] found that development of, or fear of chronic disease, or disease in relatives, prompted more health-promoting behaviours.

#### Sociocultural factors

Barrier: Lack of time. In a review [81] lack of time was reported to be a barrier to health-promoting behaviours. In one qualitative study [37] lack of time was reported to be a barrier to accessing the healthcare system for rural women.

Facilitators: 1) Being married or cohabiting. Non-attenders for health checks were more likely to be single in one systematic review [82]. Another review [83] found that not being married, not living with a partner or being single could be a barrier to lifestyle change in (in six included studies while eight studies found no association). In one cohort study [80] being married or cohabiting at mid-life was a predictor of a positive change in health behaviour. However, in another cohort study [38] marital status was not significantly associated with successful lifestyle change.

2) Education. In one review [82], those not engaging with preventative health practices were less well educated. In two other reviews, people with higher levels of education tended to be more physically active [84] and engaged in self-care activities [85]. In cohort studies, two cohort studies reported that education was one of the strongest predictors of a positive change in health behaviours [80] [21]. However, education was not associated with positive lifestyle change in another cohort study [38].

3) Support. In one qualitative study [30] women found it difficult to sustain healthy practices if they had no one supportive of their efforts. One systematic review [86] found that physicians felt that their capability in helping patients change their lifestyle was generally low in the areas of smoking, nutrition, exercise, and alcohol consumption.

4) Benefits of health behaviour including the opportunity for socialisation and improved self-esteem was reported as a facilitator for women in one systematic review [81].

Barrier/facilitator: Family responsibilities. One cohort study [80] reported that having a child at home was one of the strongest predictors of a beneficial change in health behaviours. In a review [81] caretaking responsibilities and family obligations were found to be barriers to health-promoting behaviour.

#### Physical Environment

Barrier: Distance. One review [83] found that longer travel time and greater distances from healthcare facilities, or problems with transport, were consistently associated with poorer uptake of lifestyle programmes. In rural mid-life women geographical barriers to accessing healthcare systems were reported [37].

#### Access (to facilities and resources)

Barrier: Lack of time (doctors). One review [86] found that some clinicians lacked time to spend on preventive medicine. In one US qualitative study [37] women reported that their physicians were very busy which prevented access to healthcare.

#### Psychological factors

Facilitators: 1) Self-efficacy. Two reviews found that uptake of lifestyle programmes or lack of engagement with preventive health practices was related to self-efficacy [82,83].

2) Value placed on health. One cohort study [32] found that a high value placed on health was independently associated with positive change of general health practice. In one review [82] those not engaging with preventive health practices were shown to value health less strongly. In one qualitative study [31] women who valued their health were more likely to undertake health-promoting behaviours.

Barriers: 1) Lack of time. Two reviews [81,82] found that lack of time was a barrier to health-promoting behaviour. It was also raised as an issue in two qualitative studies in mid-life women who found family obligations and caretaking responsibilities limited time for healthy behaviours [31,37].

2) Entrenched attitudes and behaviours in mid-life. One qualitative study [27] reported resistance to the idea of change in mid-life, including reluctance to be told what to do and a belief that benefits of behaviour change needed to be experienced before adoption of the changes long-term.

#### Health inequalities

Barrier: 1) Low SES. One review found that men on low incomes, low SES, and unemployed or less well educated were less likely than others to attend health check-ups [82]. Lack of money was reported in one systematic review [81].

2) Gender. One review [82] and one cohort study [21] found that men were less likely to attend health checks or adopt a healthy lifestyle. In one qualitative study [37] in US women, various roles (caring for homes, jobs, children, grandchildren, and parents) women fulfilled limited time and access to the healthcare system. One qualitative study [31] found that social responsibilities inside and outside home interfered with participation in health behaviours. One systematic review [81] found that caretaking responsibilities and family obligations for women were a barrier to health behaviours relating to CHD.

3) Ethnicity. In a cohort study [21] conducted in the US, those from African American, or BME communities were less likely to adopt a healthy lifestyle.

## Discussion

This review found a broad range of barriers and facilitators that either prevent or limit, or which help or motivate individuals to take up and maintain healthy behaviours in mid-life. Evidence was found relating to barriers and facilitators to physical activity, diet and eating, smoking, smokeless tobacco, alcohol, eye care and health behaviours in general, in particular in relation to prevention of cardiovascular disease. Evidence was sought, but not found, for barriers and facilitators to other health behaviours, including mid-life social activity and prevention of hearing loss.

Most of the available evidence is from developed countries (European nations, USA, Canada, Australia and New Zealand). In relation to health inequalities, substantial evidence was found that low SES is a barrier to healthy behaviour. Evidence for both men and women at mid-life was found and the perspectives of a range of ethnic minority groups across different health behaviours were represented. There is a comprehensive study of barriers and facilitators to PA for disabled people in the US [55], and a qualitative study relating to alcohol use in lesbian women. However, there is little evidence relevant to other minority or disadvantaged groups or for different health behaviours.

The review had a wide scope as it sought information relating to a wide range of health behaviours in mid-life. In order to provide timely evidence and obtain a manageable number of search hits, the searches were limited to studies that included terms related to mid-life in the title, abstract or indexing terms. There may be other studies that include a predominantly mid-life population that have not been indexed in this way. While this was a rapid review, established, rigorous systematic review methods have been used and reported in the methods, inclusions, quality assessment and synthesis of studies [87,88].

Remarkably, we found no overlap between studies in systematic reviews and primary studies identified perhaps reflecting differing inclusion criteria and dates for systematic reviews and the challenges of searching the large volume of work that has been published in this area. This review has only included studies published from 2000 onwards so it may be that some of the available data was published before then.

Some systematic and narrative reviews included in this review contained both qualitative and quantitative studies, including cross-sectional studies. To compensate for this we extracted and focused on longitudinal data as much as possible, without going back to individual primary studies. Most of the data in included quantitative studies was self-reported (S3 Table), and few studies used objective measures.

Many of the barriers and facilitators identified were found in a number of different studies, across different study designs, ethnic groups, in men and women. Some of the same issues were also identified in disadvantaged and minority groups. Therefore there is a fairly high degree of confidence in the crosscutting barriers and facilitators identified. Additionally, some of the same barriers and facilitators were found across studies that examined different health behaviours.

Key barriers that recur across different health behaviours include lack of time (in particular in relation to family, childcare, household and occupational responsibilities), access issues (transport, facilities and resources), financial costs, personal attitudes and behaviours (including lack of motivation), personal identity, restrictions in the physical environment, low socioeconomic status and lack of knowledge. Key facilitators include a focus on enjoyment of the healthy behaviour, doing other healthy behaviours, health benefits, prevention of illness, the potential benefits for healthy ageing and wellbeing as motivation; social support and encouragement; clear, accurate health messages, and integration of behaviours into lifestyle and routine. Specific issues relating to population and culture were identified for disadvantaged and minority groups.

While a number of studies have shown that mid-life interventions can be effective in changing health behaviours for those that participate [89], few existing mid-life interventions have addressed issues that may affect uptake and participation in programmes or individual participation in the first place. There is scope to design tailored interventions at both population and individual levels that address the mid-life barriers and facilitators identified in terms of intervention design to address capability, opportunity and motivation [90], including ease of access to and take up of programmes.

### Conclusion and Recommendations

The barriers and facilitators to mid-life health behaviours identified in this review are important considerations that can inform the design of mid-life interventions. While change in health behaviour can occur in mid-life, health behaviours may be more entrenched. Yet, there are opportunities to change behaviour as people start to think more about the need to maintain health in later life in order to make the most of retirement, family and grandchildren for example.

While interventions to change health-related behaviour in mid-life can be effective for people that participate in them, issues around access, including availability and affordability also need to be considered as part of the design of health promoting strategies to address time constraints and competing priorities, ease of access and financial issues for people in mid-life.

Strategies to promote mid-life healthy behaviours that could be considered include: The provision of locally available, affordable programmes that allow quick and easy access such as shorter, bite-sized interventions; strategies to enable people to integrate healthy behaviours into their daily lives, such as promotion of home-based interventions, for example quick home preparation of healthy food, home physical activity programmes or improving the local environment to encourage walking, cycling, active transport or healthier food outlets; promote programmes as an opportunity for socialisation, support and enjoyment; highlight the short-term and long-term benefits of healthy behaviour such as promotion of wellbeing and healthy ageing; ensure websites with information or programmes are fast and easy to use; develop interventions and strategies around children and family, using local community resources, including those that their children also use, such as schools and recreational facilities; provide interventions at convenient times and in easily accessible places, for example outside office hours, in workplaces or local community settings; provide programmes and information in a range of appropriate languages and culturally acceptable styles and facilities; target health promotion at times in people’s lives when substantial change occurs such as retirement or when children leave home; target lower socioeconomic status groups and consideration of specific strategies to encourage women to take up activity programmes.

Addressing barriers and facilitators of behaviour change in the design and implementation of public health interventions in mid-life, can inform the delivery of tailored, evidence-based strategies towards maintaining health in later life.

## Supporting Information

S1 PRISMA Checklist PRISMA Checklist. (DOC)

S1 Table Excluded Studies. (DOCX)

S2 Table QA Tables. (DOCX)

S3 Table Evidence Tables. (DOCX)

S1 Text Search Strategies. (DOCX)

S2 Text Websites Searched. (DOCX)

S3 Text Review Protocol. (DOCX)
