# Supplementary material for: Barriers and Facilitators to the Uptake and Maintenance of Healthy Behaviours by People at Mid-Life: A Rapid Systematic Review
Source: PLoS One. 2016 Jan 27;11(1):e0145074. doi: 10.1371/journal.pone.0145074 (PMC4731386; doi:10.1371/journal.pone.0145074)
Supplement: S1 Table — (DOCX) [file pone.0145074.s002.docx]

**Excluded Studies and Reason for Exclusion**

**(Systematic Reviews and Primary Studies)**

## Systematic Reviews

| **Study** | **Reason excluded** |
| --- | --- |
| Aalbers T, Baars MA, Rikkert MG. (2011) Characteristics of effective Internet-mediated interventions to change lifestyle in people aged 50 and older: a systematic review. Ageing Research Reviews 10(4): 487-497. | SR - Review 3 |
| Aarsland D, Sardahaee FS, Anderssen S et al. (2010) Is physical activity a potential preventive factor for vascular dementia? A systematic review. Aging & Mental Health 14(4): 386-395. | Review 2? |
| Abioye AI, Hajifathalian K, Danaei G. (2013) Do mass media campaigns improve physical activity? A systematic review and meta-analysis. Archives of Public Health 71(1): 20. | SR - Review 3 |
| Ackermann RT, Marrero DG, Hicks KA et al. (2006) An evaluation of cost sharing to finance a diet and physical activity intervention to prevent diabetes. Diabetes Care 29(6): 1237-1241. | Not SR - about financing interventions |
| Aggarwal B, Liao M, Mosca L. (2010) Predictors of physical activity at 1 year in a randomized controlled trial of family members of patients with cardiovascular disease. Journal of Cardiovascular Nursing 25(6): 444-449. | Not SR. Review 3? - primary paper |
| Ahmad N, Boutron I, Dechartres A et al. (2010) Applicability and generalisability of the results of systematic reviews to public health practice and policy: a systematic review. Trials 26:11:20. | SR of SRs about external generalisability |
| Ahmadi P, Kiyani R. (2011) Investigating the relationship of weight gain and obesity with minimal cognitive impairment among middle-aged people. Procedia-Social and Behavioral Sciences 30: 1849-1851. | Out of scope |
| Ahrens JN. (2009) Correlates of physical activity and wellness program completion among Mexican-American women. Dissertation Abstracts International: Section B: The Sciences and Engineering. 70:11-B. | PhD dissertation - not peer-reviewed publication |
| Akers JD, Estabrooks PA, Davy BM. (2010) Translational research: bridging the gap between long-term weight loss maintenance research and practice. Journal of the American Dietetic Association 110(10): 1511-1522. | SR about translation from research to practice |
| Akvardar Y. (2010) How are alcohol related problems prevented? Brief intervention approach in the treatment of alcohol use disorders. | Turkish language |
| Alam S, Johnson AG. (1999) A meta-analysis of randomised controlled trials (RCT) among healthy normotensive and essential hypertensive elderly patients to determine the effect of high salt (NaC1) diet on blood pressure Journal of Human Hypertension 13(6): 367-374 | Pre 2000 (but relevant to review 3) |
| Albright C, Thompson DL. (2006) The effectiveness of walking in preventing cardiovascular disease in women: A review of the current literature. Journal of Womens Health 15(3): 271-280. | Out of scope |
| Alfermann D, Stoll O. (2000) Effects of physical exercise on self-concept and well-being. International Journal of Sport Psychology 31(1): 47-65. | Out of scope |
| Ali MK, Echouffo-Tcheugui J, Williamson DF. (2012) How effective were lifestyle interventions in real-world settings that were modeled on the Diabetes Prevention Program? Health Affairs 31(1): 67-75. | SR - Review 3 |
| Allen JC, Lewis JB, Tagliaferro AR. (2012) Cost-effectiveness of health risk reduction after lifestyle education in the small workplace. Preventing Chronic Disease 9: E96. | Cost-effectiveness study Review 3 - primary paper? |
| Allman RM, Baker PS, Maisiak RM et al. (2004) Racial similarities and differences in predictors of mobility change over eighteen months. Journal of General Internal Medicine 19(11): 1118-1126. | Not SR, all data older adults >65 |
| Alterman AI, Gariti P, Mulvaney F. (2001) Short- and long-term smoking cessation for three levels of intensity of behavioral treatment, Psychology of Addictive Behaviors 15(3): 261-264. | Cost-effectiveness study Review 3 - primary paper? |
| Ammerman AS, Lindquist CH, Lohr KN et al. (2002) The efficacy of behavioral interventions to modify dietary fat and fruit and vegetable intake: a review of the evidence. Preventive Medicine 35(1): 25-41. | SR (Review 3) |
| An LC, Schillo BA, Kavanaugh AM et al. (2006) Increased reach and effectiveness of a statewide tobacco quitline after the addition of access to free nicotine replacement therapy. Tobacco Control 15(4): 286-293 | Before and after study - primary paper, not midlife |
| An RP. (2013) Effectiveness of subsidies in promoting healthy food purchases and consumption: a review of field experiments. Public Health Nutrition 16(7): 1215-1228. | SR BUT also children included depends if can separate adult conclusions. Rev 3? |
| Andersen RE, Jakicic JM. (2009) Interpreting the physical activity guidelines for health and weight management. Journal of Physical Activity & Health 6(5): 651-656. | Out of scope |
| Anderson JW, Konz EC, Frederich RC et al. (2001) Long-term weight-loss maintenance: a meta-analysis of US studies. American Journal of Clinical Nutrition 74(5): 579-584. | SR (Review 3) |
| Anderson LM, Quinn TA, Glanz K et al. (2009) The effectiveness of worksite nutrition and physical activity interventions for controlling employee overweight and obesity: a systematic review. American Journal of Preventive Medicine 37(4): 340-357. | SR (Review 3) |
| Angevaren M, Aufdemkampe G, Verhaar HJ et al. (2008) Physical activity and enhanced fitness to improve cognitive function in older people without known cognitive impairment. Cochrane Database of Systematic Reviews 16(3): CD005381. | Participants >55 included but vast majority of studies, participants >65 |
| Annemans L, Lamotte M, Clarys P et al. (2007) Health economic evaluation of controlled and maintained physical exercise in the prevention of cardiovascular and other prosperity diseases. European Journal of Cardiovascular Prevention and Rehabilitation 14(6): 815-824. | Cost-effectiveness study Review 3 - primary paper? |
| Anokye NK, Trueman P, Green C et al. (2011) The cost-effectiveness of exercise referral schemes. BMC Public Health 16;11:954. | Cost-effectiveness study Review 3 - primary paper? |
| Antikainen I, Ellis R. (2011) A RE-AIM evaluation of theory-based physical activity interventions. Journal of Sport & Exercise Psychology 33(2): 198-214. | Out of scope |
| Arab L, Liu W, Elashoff D. (2009) Green and black tea consumption and risk of stroke: a meta-analysis. Stroke 40(5): 1786-1792. | Review 2? |
| Archer E, Groessl EJ, Sui X et al. (2012) An economic analysis of traditional and technology-based approaches to weight loss. American Journal of Preventive Medicine 43(2): 176-182. | Cost-effectiveness study Review 3 - primary paper? |
| Arcury TA, Quandt SA, Bell RA. (2001) Staying healthy: the salience and meaning of health maintenance behaviors among rural older adults in North Carolina. Social Science & Medicine 53(11): 1541-1556. | Out of scope, older adults |
| Arent SM, Landers DM, Etnier JL. (2000) The effects of exercise on mood in older adults: A meta-analytic review. Journal of Aging and Physical Activity 8(4): 407-430. | Older adults only >65 |
| Arlinger S. (2003) Negative consequences of uncorrected hearing loss - a review. International Journal of Audiology 42: S17-S20. | Consequences of hearing loss, not barriers/facilitators. |
| Arnold CM, Sran MM, Harrison EL. (2008) Exercise for fall risk reduction in community-dwelling older adults: a systematic review. Physiotherapy Canada 60(4): 358-372. | Inclusion criteria > 50 years but only 4 of 35 studies included adults >50, rest all >65. |
| Arcury TA, Quandt SA, Bell RB. (2001) Staying healthy: the salience and meaning of health maintenance behaviors among rural older adults in North Carolina. Social Science & Medicine 53: 1541–1556. | Qualitative study, older adults only all participants >70 |
| Asher RC, Burrows TL, Collins CE. (2013) Very low-energy diets for weight loss in adults: A review. Nutrition & Dietetics 70(2): 101-112. | Review 3 SR |
| Ashford S, Edmunds J, French DP. (2010) What is the best way to change self-efficacy to promote lifestyle and recreational physical activity? A systematic review with meta-analysis. British Journal of Health Psychology 15(Pt 2): 265-288. | SR (Review 3) |
| Ashworth NL, Chad KE, Harrison EL et al. (2005) Home versus center based physical activity programs in older adults. Cochrane Database of Systematic Reviews 25(1): CD004017. | Most included studies in people with established CVD or COPD but some in those with risk factors |
| Asikainen TM, Kukkonen-Harjula K, Miilunpalo S. (2004) Exercise for health for early postmenopausal women: a systematic review of randomised controlled trials. Sports Medicine 34(11): 753-778. | Review 3? |
| Atienza AA. (2001) Home-based physical activity programs for middle-aged and older adults: Summary of empirical research. Journal of Aging and Physical Activity 9: S38-S58. | Not a SR, may have useful primary studies for review 3 |
| Aucott L, Gray D, Rothnie H et al. (2011) Effects of lifestyle interventions and long-term weight loss on lipid outcomes - a systematic review. Obesity Reviews 12(5): e412-425. | Review 3? |
| Aucott LS. (2008) Influences of weight loss on long-term diabetes outcomes. Proceedings of the Nutrition Society 67(1): 54-59. | About links between obesity and diabetes |
| Aucott L, Rothnie H, McIntyre L et al. (2009) Long-term weight loss from lifestyle intervention benefits blood pressure: a systematic review. Hypertension 54(4): 756-762 | For review 2 and 3? |
| Aucott L, Gray D, Rothnie H et al. (2011) Effects of lifestyle interventions and long-term weight loss on lipid outcomes - a systematic review. Obesity Reviews 12(5): e412-25. | Review 3? |
| Avery KN, Donovan JL, Horwood J et al. (2013) Behavior theory for dietary interventions for cancer prevention: A systematic review of utilization and effectiveness in creating behavior change. Cancer Causes and Control 24(3): 409-420. | Out of scope |
| Babcock Irvin C, Wyer PC et al. (2000) Preventive care in the emergency department, Part II: Clinical preventive services--an emergency medicine evidence-based review. Society for Academic Emergency Medicine Public Health and Education Task Force Preventive Services Work Group. Academic Emergency Medicine 7(9): 1042-1054. | Include for tobacco cessation counselling only |
| Bacigalupo R, Cudd P, Littlewood C et al. (2013) Interventions employing mobile technology for overweight and obesity: an early systematic review of randomized controlled trials. Obesity Reviews 14(4): 279-291. | Out of scope |
| Bader P, Boisclair D, Ferrence R. (2011) Effects of tobacco taxation and pricing on smoking behavior in high risk populations: a knowledge synthesis. International Journal of Environmental Research & Public Health 8(11): 4118-4139. | About tobacco taxation and pricing |
| Baker PR, Francis DP, Soares J et al. (2011) Community wide interventions for increasing physical activity. Cochrane Database of Systematic Reviews 13(4): CD008366. | Out of scope |
| Baker MK, Simpson K, Lloyd B et al. (2011) Behavioral strategies in diabetes prevention programs: A systematic review of randomized controlled trials. Diabetes Research & Clinical Practice 91(1): 1-12. | Out of scope |
| Baker AL, Thornton LK, Hiles S et al. (2012) Psychological interventions for alcohol misuse among people with co-occurring depression or anxiety disorders: a systematic review. Journal of Affective Disorders 139(3): 217-229. | Alcohol misuse in people with existing MH problems |
| Bala MM, Lesniak W. (2007) Efficacy of non-pharmacological methods used for treating tobacco dependence: meta-analysis. Polskie Archiwum Medycyny Wewnetrznej 117(11-12): 504-511. | Out of scope |
| Bala M, Strzeszynski L, Cahill K. (2008) Mass media interventions for smoking cessation in adults. Cochrane Database of Systematic Reviews 23;(1): CD004704. | Updated in 2013 |
| Bala MM, Strzeszynski L, Topor-Madry R et al. (2013) Mass media interventions for smoking cessation in adults. Cochrane Database of Systematic Reviews 6;6: CD004704. | Out of scope |
| Baldwin RC. (2010) Preventing late-life depression: a clinical update. International Psychogeriatrics 22(8): 1216-1224. | Not a SR |
| Ballesteros J, Duffy JC, Querejeta I et al. (2004) Efficacy of brief interventions for hazardous drinkers in primary care: Systematic review and meta-analyses. Alcoholism-Clinical and Experimental Research 28(4): 608-618 | Brief int for hazardous drinkers in primary care - male vs female comparison |
| Ballesteros J, González-Pinto A, Querejeta I et al. (2004) Brief interventions for hazardous drinkers delivered in primary care are equally effective in men and women. Addiction 99(1): 103-108 | Out of scope |
| Banham L, Gilbody S. (2010) Smoking cessation in severe mental illness: what works? Addiction 105(7): 1176-1189. | Out of scope |
| Banks-Wallace J, Conn V. (2002) Interventions to promote physical activity among African American women. Public Health Nursing 19(5): 321-335 | Not much HB reported but perhaps should include to show it has been considered |
| Barbeau EM, Leavy-Sperounis A, Balbach ED. (2004) Smoking, social class, and gender: what can public health learn from the tobacco industry about disparities in smoking? Tobacco Control 13(2): 115-120. | Consider as primary study for review 1, not midlife |
| Barber SE, Clegg AP, Young JB. (2012) Is there a role for physical activity in preventing cognitive decline in people with mild cognitive impairment? Age and Ageing 41(1): 5-8. | Not a SR |
| Barker F, Mackenzie E, Elliott L et al. (2013) Interventions to improve hearing aid use in adult auditory rehabilitation. Cochrane Database of Systematic Reviews: CD010342 | Protocol only in people with existing hearing loss |
| Barnes J, Dong CY, McRobbie H et al. (2010) Hypnotherapy for smoking cessation. Cochrane Database of Systematic Reviews 10: CD001008. | In for review 3 |
| Barnes D, Yaffe K. (2011) The projected impact of risk factor reduction on alzheimer's disease prevalence. Alzheimer’s and Dementia 1): S511. | Not a SR but v relevant for review 2 |
| Barnes D. (2012) Risk factor reduction and Alzheimer's disease prevalence: Projected effect and practical implications. Alzheimer's and Dementia 8(4): P605. | Abstract only |
| Barr SI. (2003) Increased dairy product or calcium intake: Is body weight or composition affected in humans? Journal of Nutrition 133(1): 245S-248S. | Consider refs for review 3 but does not meet SR criteria |
| Barrera M Jr, Castro FG, Strycker LA et al. (2013) Cultural adaptations of behavioral health interventions: a progress report. Journal of Consulting and Clinical Psychology 81(2): 196-205. | May be useful refs for primary studies |
| Barte JC, ter Bogt NC, Bogers RP et al. (2010) Maintenance of weight loss after lifestyle interventions for overweight and obesity, a systematic review. Obesity Reviews 11(12): 899-906. | Review 3? |
| Bar Barton P, Andronis L, Briggs A et al. (2011) Effectiveness and cost effectiveness of cardiovascular disease prevention in whole populations: modelling study. BMJ 28;343:d4044. | Review 3? |
| Barton GR, Goodall M, Bower P et al. (2012) Increasing heart-health lifestyles in deprived communities: economic evaluation of lay health trainers. Journal of Evaluation in Clinical Practice 18(4): 835-840. | Review 3? |
| Bath-Hextall F, Leonardi-Bee J, Somchand N et al. (2007) Interventions for preventing non-melanoma skin cancers in high-risk groups. Cochrane Database of Systematic Reviews 17(4): CD005414. | Rev 3. Check if we are including sun exposure? |
| Batty GD. (2002) Physical activity and coronary heart disease in older adults. A systematic review of epidemiological studies. European Journal of Public Health 12(3): 171-176. | Review 2? Older adults |
| Batty GD, Shipley MJ, Gunnell D et al. (2009) Height, wealth, and health: An overview with new data from three longitudinal studies. Economics & Human Biology 7(2): 137-152. | Health consequences of height - not lifestyle |
| Batty GD, Shipley MJ, Kivimaki M et al. (2010) Walking pace, leisure time physical activity, and resting heart rate in relation to disease-specific mortality in London: 40 years follow-up of the original Whitehall study. An update of our work with Professor Jerry N. Morris (1910-2009). Annals of Epidemiology 20(9): 661-669. | Review 2? |
| Beauchamp A, Peeters A, Tonkin A. (2010) Inequalities in cardiovascular disease mortality: the role of behavioural, physiological and social risk factors. Journal of Epidemiology and Community Health 64(6): 542-548. | Review 2? |
| Beemsterboer W, Stewart R, Groothoff J et al. (2008) The influence of sick leave frequency determinants on homogeneous groups in two socio-economically comparable, but socio-culturally different regions in The Netherlands. Central European Journal of Public Health 16(4): 151-160. | Out of scope |
| Beydoun MA, Wang FA. (2010) Pathways linking socioeconomic status to obesity through depression and lifestyle factors among young US adults. Journal of Affective Disorders 123(1-3): 52-63. | Consider as primary study review 2 |
| Bezerra IN, Curioni C, Sichieri R. (2012) Association between eating out of home and body weight. Nutrition Reviews 70(2): 65-79. | Include for review 2 SR |
| Birnie K, Cooper R, Martin RM et al. (2011) Childhood socioeconomic position and objectively measured physical capability levels in adulthood: A systematic review and meta-analysis. PLoS One 6(1): e15564. | Out of scope |
| Black AP, Brimblecombe J, Eyles H et al. (2012) Food subsidy programs and the health and nutritional status of disadvantaged families in high income countries: a systematic review. BMC Public Health 21;12: 1099. | SR consider review 3 |
| Blagojevic M, Jinks C, Jeffery A et al. (2010) Risk factors for onset of osteoarthritis of the knee in older adults: a systematic review and meta-analysis. Osteoarthritis & Cartilage 18(1): 24-33. | SR consider review 2 |
| Blaine BE, Rodman J, Newman JM. (2007) Weight loss treatment and psychological well-being: a review and meta-analysis. Journal of Health Psychology 12(1): 66-82. | Links between weight loss and depression |
| Blair SN, Wei M. (2000) Sedentary habits, health, and function in older women and men. American Journal of Health Promotion 15(1): 1-8. | Out of scope |
| Blair SN, Cheng Y, Holder JS. (2001) Is physical activity or physical fitness more important in defining health benefits? Medicine and Science in Sports and Exercise 33(6): S379-S399. | Consider for review 2 |
| Blamey A, Nutrie N. (2004) Changing the individual to promote health-enhancing physical activity: the difficulties of producing evidence and translating it into practice. Journal of Sports Sciences 22(8): 741-754. | Out of scope |
| Blankers M, Nabitz U, Smit F et al. (2012) Economic evaluation of internet-based interventions for harmful alcohol use alongside a pragmatic randomized controlled trial. Journal of Medical Internet Research 14(5): 71-83. | Consider as primary study review 3 |
| Blosnich JR. (2012) Deconstructing disparity: Examining risk factors related to smoking among sexual minority populations. Dissertation Abstracts International: Section B: The Sciences and Engineering 73(3-B). | Risk factors for smoking in sexual minorities |
| Bodner ME, Dean E. (2009) Advice as a smoking cessation strategy: a systematic review and implications for physical therapists. Physiotherapy Theory & Practice 25(5-6): 369-407. | SR - For review 3? |
| Boehm J, Franklin RC, Newitt R et al. (2013) Barriers and motivators to exercise for older adults: A focus on those living in rural and remote areas of Australia. Australian Journal of Rural Health 21(3): 141-149. | SR, older people >50. Not sure about quality assessment - qualitative studies. |
| Bolam KA, van Uffelen JG, Taaffe DR. (2013) The effect of physical exercise on bone density in middle-aged and older men: a systematic review. Osteoporos International 24(11): 2749-62. | Consider as SR for review 3 |
| Bolego C, Poli A, Paoletti R.  (2002) Smoking and gender. Cardiovascular Research 53(3): 568-576. | Not a SR. Not a relevant primary study. |
| Bollenberg BW. (2004) Smoking cessation experience survey. Dissertation Abstracts International: Section B: The Sciences and Engineering 65(3-B). | Consider as primary study review 1, not midlife |
| Boon B, Risselada A, Huiberts A et al. (2011) Curbing alcohol use in male adults through computer generated personalized advice: randomized controlled trial. Journal of Medical Internet Research 13(2): e43. | Consider as primary study review 3, not midlife |
| Boone-Heinonen J, Evenson KR et al. (2009) Walking for prevention of cardiovascular disease in men and women: A systematic review of observational studies: Obesity Prevention. Obesity Reviews 10(2): 204-217. | Consider for review 2 |
| Borer KT. (2008) How effective is exercise in producing fat loss? Kinesiology 40(2): 126-137 | Out of scope |
| Borok J, Galier P, Dinolfo M et al. (2013) Why do older unhealthy drinkers decide to make changes or not in their alcohol consumption? Data from the Healthy Living As You Age study. Journal of the American Geriatrics Society 61(8): 1296-1302. | Out of scope |
| Bós AM, Howard BV, Beresford SA et al. (2011) Cost-effectiveness analysis of a low-fat diet in the prevention of breast and ovarian cancer. Journal of the American Dietetic Association 111(1): 56-66. | Consider as primary study review 3 |
| Boyette LW, Lloyd A, Manuel S et al. (2001) Development of an exercise expert system for older adults. Journal of Rehabilitation Research and Development 38(1): 79-91. | Not a SR. Consider as primary study for review 3. |
| Boyette LW, Lloyd A, Boyette JE et al. (2002) Personal characteristics that influence exercise behavior of older adults. Journal of Rehabilitation Research & Development 39(1): 95-103. | Not a SR. Older adults>60 |
| Boylan S, Louie JC, Gill TP. (2012) Consumer response to healthy eating, physical activity and weight-related recommendations: a systematic review. Obesity Reviews 13(7): 606-617. | Not sure about study quality |
| Boyle RG, Solberg LI, Fiore MC. (2010) Electronic medical records to increase the clinical treatment of tobacco dependence: a systematic review. American Journal of Preventive Medicine 39(6 Suppl 1): S77-82. | Clinician behaviour |
| Brambila-Macias J, Shankar B, Capacci S et al. (2011) Policy interventions to promote healthy eating: a review of what works, what does not, and what is promising. Food & Nutrition Bulletin 32(4): 365-375. | Out of scope |
| Brand DJ, Alston RJ, Harley DA. (2012) Disability and race: a comparative analysis of physical activity patterns and health status. Disability and Rehabilitation 34(10): 795-801. | Out of scope |
| Bravata DM, Sanders L, Huang J et al. (2003) Efficacy and safety of low-carbohydrate diets: a systematic review. JAMA: Journal of the American Medical Association 289(14): 1837-1850. | Consider for review 3 |
| Brawley LR, Rejeski WJ, King AC. (2003) Promoting physical activity for older adults - The challenges for changing behavior. American Journal of Preventive Medicine 25(3): 172-183. | Out of scope |
| Bridle C, Spanjers K, Patel S et al. (2012) Effect of exercise on depression severity in older people: systematic review and meta-analysis of randomised controlled trials. British Journal of Psychiatry 201(3): 180-185 | Out of scope |
| Brindal E, Hendrie G, Freyne J et al. (2013) Design and pilot results of a mobile phone weight-loss application for women starting a meal replacement programme. Journal of Telemedicine and Telecare 19(3): 166-174. | Out of scope |
| Brindle P, Beswick A, Fahey T et al. (2006) Accuracy and impact of risk assessment in the primary prevention of cardiovascular disease: a systematic review. Heart 92(12): 1752-1759. | Out of scope |
| Brody JG, Rudel RA, Michels KB et al. (2007) Environmental pollutants, diet, physical activity, body size, and breast cancer: Where do we stand in research to identify opportunities for prevention? Cancer 109(12): 2627-2634. | Consider review 2 |
| Brouwer W, Oenema A, Crutzen R et al. (2008) An exploration of factors related to dissemination of and exposure to internet-delivered behavior change interventions aimed at adults: A Delphi Study Approach. Journal of Medical Internet Research 10(2): e10. | Consider as a primary study? |
| Brown WJ, Mishra G, Lee C et al. (2000) Leisure time physical activity in Australian women: Relationship with well being and symptoms. Research Quarterly for Exercise and Sport 71(3): 206-216. | Consider as primary study for review 2. |
| Brown T, Avenell A, Edmunds LD et al. (2009) Systematic review of long-term lifestyle interventions to prevent weight gain and morbidity in adults. Obesity Reviews 10(6): 627-638. | SR, Consider for review 3. |
| Brown J, Michie S, Geraghty AW et al. (2012) A pilot study of StopAdvisor: A theory-based interactive internet-based smoking cessation intervention aimed across the social spectrum. Addictive Behaviors 37(12): 1365-1370. | Not a SR. Consider as primary study for review 3. |
| Brown WJ, McLaughlin D, Leung J et al. (2012) Physical activity and all-cause mortality in older women and men. British Journal of Sports Medicine 46(9): 664-668. | Abstract only |
| Brunner EJ, Thorogood M, Rees K et al. (2005) Dietary advice for reducing cardiovascular risk. Cochrane Database of Systematic Reviews (4): CD002128 | Updated 2007 |
| Brunner EJ, Thorogood M, Rees K et al. (2007) Dietary advice for reducing cardiovascular risk. Cochrane Database of Systematic Reviews (4): CD002128. | Consider for review 3 |
| Bucher HC, Hengstler P, Schindler C et al. (2002) N-3 polyunsaturated fatty acids in coronary heart disease: a meta-analysis of randomized controlled trials. American Journal of Medicine 112(4): 298-304. | Consider for review 3 |
| Buchholz SW, Huffman D, McKenna JC. (2012) Overweight and obese low-income women: restorative health behaviors under overwhelming conditions. Health Care for Women International 33(2): 182-197. | Consider as primary study review 1 |
| Buchholz SW, Wilbur J, Ingram D et al. (2013) Physical activity text messaging interventions in adults: a systematic review. Worldviews on Evidence-Based Nursing 10(3): 163-173. | Text messaging - Review 3? |
| Buchthal OV. (2013) The role of social capital in changing dietary behavior in a low-income multi-ethnic community. Dissertation Abstracts International: Section B: The Sciences and Engineering 74 1-B E. | Out of scope |
| Buckner JD, Heimberg RG, Ecker AH et al. (2013) A biopsychosocial model of social anxiety and substance use. Depression and Anxiety 30(3): 276-284. | Out of scope |
| Buechter RB, Fechtelpeter D. (2011) Climbing for preventing and treating health problems: a systematic review of randomized controlled trials. German Medical Science 9: Doc19. | Not relevant populations - with existing conditions or children |
| Büla CJ, Monod S, Hoskovec C et al. (2011) Interventions aiming at balance confidence improvement in older adults: an updated review. Gerontology 57(3): 276-286. | Out of scope |
| Bull FC, Holt CL, Kreuter MW et al. (2001) Understanding the effects of printed health education materials: Which features lead to which outcomes? Journal of Health Communication 6(3): 265-279. | Not a SR - Consider as primary study for review 1 |
| Bullen C, Howe C, Lin RB et al. (2010) Pre-cessation nicotine replacement therapy: pragmatic randomized trial. Addiction 105(8): 1474-1483. | Not a SR - Consider as primary study for review 3 |
| Bulut S. (2009) Late life depression: A literature review of late-life depression and contributing factors. Anales De Psicologia 25(1): 21-26. | Not a SR |
| Bunn F, Dickinson A, Barnett-Page E et al. (2008) A systematic review of older people's perceptions of facilitators and barriers to participation in falls-prevention interventions. Ageing & Society 28(4): 449-472. | SR but in older people only >65 y |
| Burns RA, Birrell CL, Steel D et al. (2013) Alcohol and smoking consumption behaviours in older Australian adults: prevalence, period and socio-demographic differentials in the DYNOPTA sample. Social Psychiatry and Psychiatric Epidemiology 48(3): 493-502. | Not a SR or primary study |
| Burrows J, Carlisle J. (2010) They don’t want it ramming down their throats. Learning from the perspectives of current and ex-smokers with smoking-related illness to improve communication in primary care: A qualitative study. Primary Health Care Research and Development 11(3): 206-214. | Not a SR or relevant primary study - people with existing COPD, aged >65 mainly |
| Burton NW, Oldenburg B, Sallis JF et al. (2007) Measuring psychological, social, and environmental influences on leisure-time physical activity among adults. Australian and New Zealand Journal of Public Health 31(1): 36-43. | Not a SR or relevant topic |
| Cahill K, Lancaster T, Green N. (2010) Stage-based interventions for smoking cessation. Cochrane Database of Systematic Reviews 10;(11): CD004492. | Cochrane SR, stages of change |
| Campos S, Doxey J, Hammond D. (2011) Nutrition labels on pre-packaged foods: a systematic review. Public Health Nutrition 14(8) 1496-506. | Out of scope |
| Cardona-Morrell M, Rychetnik L, Morrell SL et al. (2010) Reduction of diabetes risk in routine clinical practice: are physical activity and nutrition interventions feasible and are the outcomes from reference trials replicable? A systematic review and meta-analysis. BMC Public Health 29;10:653. | Translation - external generalisability, consider for review 3 |
| Caro JJ, Getsios D, Caro I et al. (2004) Economic evaluation of therapeutic interventions to prevent Type 2 diabetes in Canada. Diabetic Medicine 11: 1229-1236. | Out of scope |
| Carter P, Gray LJ, Troughton J et al. (2010) Fruit and vegetable intake and incidence of type 2 diabetes mellitus: systematic review and meta-analysis. BMJ 18:341:c4229. | Include for review 2 |
| Cavill JL, Jancey JM, Howat P. (2012) Review and recommendations for online physical activity and nutrition programmes targeted at over 40s. Global Health Promotion 19(2): 44-53. | Out of scope |
| Cecchini M, Sassi F, Lauer JA et al. (2010) Tackling of unhealthy diets, physical inactivity, and obesity: health effects and cost-effectiveness. Lancet 20;376(9754):1775-8. | Not a SR, for review 3? |
| Chan MF, Ko CY. (2006) Osteoporosis prevention education programme for women. Journal of Advanced Nursing 54(2): 159-170. | Consider as primary study review 3 |
| Chan CB, Ryan DA. (2009) Assessing the effects of weather conditions on physical activity participation using objective measures. International Journal of Environmental Research and Public Health 6(10): 2639-2654. | Not a SR, Older people only |
| Chan CW, Chan LP. (2012) Lifestyle health promotion interventions for the nursing workforce: A systematic review. Journal of Clinical Nursing 21(15-16): 2247-2261. | SR lack of consideration of nurses for intervention could be a barrier? |
| Chang YK, Labban JD, Gapin JI et al. (2012) The effects of acute exercise on cognitive performance: A meta-analysis. Brain Research 1453: 87-101. | Effects of acute exercise on cog perf. |
| Chapman J, Qureshi N, Kai J. (2013) Effectiveness of physical activity and dietary interventions in South Asian populations: a systematic review. British Journal of General Practice 63:607: e104-14. | Out of scope |
| Charlton KE. (2002) Eating well: ageing gracefully! Asia Pacific Journal of Clinical Nutrition 11: S607-S617. | Out of scope |
| Chen KM, Tseng WS, Ting LF et al. (2007) Development and evaluation of a yoga exercise programme for older adults. Journal of Advanced Nursing 57(4): 432-441. | Not a SR, older adults only |
| Chen KM, Chen MH, Hong SM et al. (2008) Physical fitness of older adults in senior activity centres after 24-week silver yoga exercises. Journal of Clinical Nursing 17(19): 2634-2646. | Older adults only |
| Chen MU, Pan AN, Malik VS et al. (2012) Effects of dairy intake on body weight and fat: a meta-analysis of randomized controlled trials. American Journal of Clinical Nutrition 96(4): 735-47. | Consider for review 3 |
| Chen, YF, Madan J, Welton N et al. (2012) Effectiveness and cost-effectiveness of computer and other electronic aids for smoking cessation: a systematic review and network meta-analysis. Health Technology Assessment 16(38): 1-205, iii-v. | SR - consider for review 3? |
| Chiu M, Austin PC, Manuel DG et al. (2010) Comparison of cardiovascular risk profiles among ethnic groups using population health surveys between 1996 and 2007. CMAJ Canadian Medical Association Journal 182(8): E301-310. | Not a SR, pooled x-sectional studies |
| Chodzko-Zajko W, Sheppard L, Senior J et al. (2005) The national blueprint for promoting physical activity in the mid-life and older adult population. Quest 57(1): 2-11. | Not a SR, follow up of Atienza 2001 |
| Ciliska D, Robinson P, Horsley T et al. (2006) Diffusion and dissemination of evidence-based dietary strategies for the prevention of cancer. Current Oncology 13(4): 130-40. | SR - consider for review 3 |
| Civljak M, Sheikh A, Stead LF et al. (2010) Internet-based interventions for smoking cessation. Cochrane Database of Systematic Reviews 9: CD007078. | SR - consider for review 3 |
| Clark DO, Frankel RM, Morgan DL et al. (2008) The meaning and significance of self-management among socioeconomically vulnerable older adults. Journals of Gerontology Series B-Psychological Sciences and Social Sciences 63(5): S312-S319. | Not a SR, older adults only |
| Clark F, Jackson J, Carlson M et al. (2012) Effectiveness of a lifestyle intervention in promoting the well-being of independently living older people: results of the Well Elderly 2 Randomised Controlled Trial. Journal of Epidemiology and Community Health 66(9): 782-90. | Not a SR, older adults only |
| Clark IN, Taylor NF, Baker F. (2012) Music interventions and physical activity in older adults: a systematic literature review and meta-analysis. Journal of Rehabilitation Medicine 44(9): 710-719. | Not a SR, older adults only |
| Clarke P, Nieuwenhuijsen EF. (2009) Environments for healthy ageing: A critical review. Maturitas 64(1): 14-19. | Not a SR, older adults only |
| Cleland CL, Tully MA, Kee F et al. (2012) The effectiveness of physical activity interventions in socio-economically disadvantaged communities: a systematic review. Preventive Medicine 54(6): 371-380. | SR - consider for review 3? |
| Cleland, V, Granados A, Crawford D et al. (2013) Effectiveness of interventions to promote physical activity among socioeconomically disadvantaged women: a systematic review and meta-analysis. Obesity Reviews 14:3 p197-212. | SR - consider for review 3? |
| Clemens SL, Grant BM, Matthews SL. (2009) A review of the impacts of health and health behaviors on women's alcohol use. American Journal of Health Behavior 33(4): 400-415. | Quality of review? |
| Clement S, Ibrahim S, Crichton N et al. (2009) Complex interventions to improve the health of people with limited literacy: A systematic review. Patient Education & Counseling 75(3): 340-351. | SR - consider for review 3? |
| Clifton PM, Bastiaans K, Keogh JB. (2009) High protein diets decrease total and abdominal fat and improve CVD risk profile in overweight and obese men and women with elevated triacylglycerol. Nutrition Metabolism & Cardiovascular Diseases 19(8): 548-554 | Not SR - consider review 3 |
| Coday M, Klesges LM, Garrison RJ et al. (2002) Health Opportunities with Physical Exercise (HOPE): social contextual interventions to reduce sedentary behavior in urban settings. Health Education Research 17(5): 637-647. | Not SR, no results |
| Cohen DA, Lapham S, Evenson KR et al. (2013) Use of neighbourhood parks: does socio-economic status matter? A four-city study. Public Health 127(4): 325-332. | x-sectional |
| Colcombe S, Kramer AF. (2003) Fitness effects on the cognitive function of older adults: a meta-analytic study. Psychological Science 14(2): 125-130. | SR - consider for review 3? |
| Collaboration, Prospective Studies. (2007) Blood cholesterol and vascular mortality by age, sex, and blood pressure: a meta-analysis of individual data from 61 prospective studies with 55 000 vascular deaths. The Lancet 370: 1829-1839. | Out of scope |
| Conklin AI, Maguire ER, Monsivais P. (2013) Economic determinants of diet in older adults: systematic review. Journal of Epidemiology & Community Health 67(9): 721-727. | SR - older adults only |
| Conn VS, Valentine JC, Cooper HM. (2002) Interventions to increase physical activity among aging adults: a meta-analysis. Annals of Behavioral Medicine 24(3): 190-200. | SR - older adults only |
| Conn VS, Minor MA, Burks KJ et al. (2003) Integrative review of physical activity intervention research with aging adults. Journal of the American Geriatrics Society 51(8): 1159-1168. | SR - older adults only |
| Conn VS, Hafdahl AR, Cooper AS et al. (2009) Meta-analysis of workplace physical activity interventions. American Journal of Preventive Medicine 37(4): 330-9. | SR - review 3 worksite interventions |
| Conn VS. (2010) Depressive symptom outcomes of physical activity interventions: meta-analysis findings. Annals of Behavioral Medicine 39(2): 128-138. | SR - review 2? |
| Conn VS. (2010) Anxiety outcomes after physical activity interventions: meta-analysis findings. Nursing Research 59(3): 224-231. | SR - review 3? |
| Conn VS, Hafdahl AR, Mehr DR. (2011) Interventions to increase physical activity among healthy adults: meta-analysis of outcomes. American Journal of Public Health 101(4): 751-758. | SR –rev 3 |
| Conn VS, Phillips LJ, Ruppar TM et al. (2012) Physical activity interventions with healthy minority adults: meta-analysis of behavior and health outcomes. Journal of Health Care for the Poor & Underserved 23(1): 59-80. | SR –rev 3 |
| Connell P, Wolfe C, McKevitt C. (2008) Preventing stroke: a narrative review of community interventions for improving hypertension control in black adults. Health & Social Care in the Community 16(2): 165-187. | SR, includes studies in people with existing hypertension, not much about HB |
| Conti A, Voelkl JE. (2009) The potential role of leisure in the prevention of dementia. Annual in Therapeutic Recreation 17: 31-45. | Out of scope |
| Cornelissen VA, Fagard RH. (2005) Effect of resistance training on resting blood pressure: a meta-analysis of randomized controlled trials. Journal of Hypertension 23(2): 251-259. | SR - review 3? |
| Cornelissen VA, Fagard RH, Coeckelberghs E et al. (2010) Resistance training and blood pressure: A meta-analysis of randomized controlled trials. European Journal of Cardiovascular Prevention and Rehabilitation 17: S32. | SR - review 3? |
| Cornelissen VA, Smart NA. (2013) Exercise training for blood pressure: a systematic review and meta-analysis. Journal of the American Heart Association 2(1): e004473. | SR - review 3? |
| Cousins SO. (2003) Seniors say the "darndest" things about exercise: Quotable quotes that stimulate applied gerontology. Journal of Applied Gerontology 22(3): 359-378. | Not SR, older adults >70 |
| Covas MI, Marrugat J, Fitó M et al. (2002) Scientific aspects that justify the benefits of the Mediterranean diet - mild-to-moderate versus heavy drinking. Alcohol and Wine in Health and Disease 957: 162-173. | Not SR |
| Cox RH, White AH, Gaylord CK. (2003) A video lesson series is effective in changing the dietary intakes and food-related behaviors of low-income homemakers.Journal of the American Dietetic Association 103(11): 1488-1493. | Not SR, consider review 3 primary |
| Crichton GE, Bryan J, Murphy KJ et al. (2010) Review of dairy consumption and cognitive performance in adults: findings and methodological issues. Dementia & Geriatric Cognitive Disorders 30(4): 352-361. | SR - review 2? |
| Cromwell SL, Berg JA. (2006) Lifelong physical activity patterns of sedentary Mexican American women. Geriatric Nursing 27(4): 209-213. | Not SR, not midlife, patterns rather than behaviours. |
| Crouch R, Wilson A, Newbury J. (2011) A systematic review of the effectiveness of primary health education or intervention programs in improving rural women's knowledge of heart disease risk factors and changing lifestyle behaviours. International Journal of Evidence-Based Healthcare 9(3): 236-245. | SR - review 3? |
| Cugelman B, Thelwall M, Dawes P. (2011) Online interventions for social marketing health behavior change campaigns: a meta-analysis of psychological architectures and adherence factors. Journal of Medical Internet Research 13(1): e17. | SR - review 3? |
| Cummings SM, Cooper RL, Cassie KM. (2009) Motivational interviewing to affect behavioral change in older adults. Research on Social Work Practice 19(2): 195-204. | SR, adults > 50 with acute and chronic illness |
| Cundick KE. (2004) The effects of long-term smoking on the cognitive function of older adults. Dissertation Abstracts International: Section B: The Sciences and Engineering 64(9-B). | Out of scope |
| Cunningham GO, Michael YL. (2004) Concepts guiding the study of the impact of the built environment on physical activity for older adults: A review of the literature. American Journal of Health Promotion 18(6): 435-443. | Out of scope |
| Curioni CC, Lourenco PM. (2005) Long-term weight loss after diet and exercise: A systematic review. International Journal of Obesity 29(10): 1168-1174. | SR - review 3? |
| Curioni C, André C, Veras R. (2006) Weight reduction for primary prevention of stroke in adults with overweight or obesity. Cochrane Database of Systematic Review 4. | SR - review 3? |
| Curry SJ, Grothaus LC, McAfee T et al. (1998) Use and cost effectiveness of smoking-cessation services under four insurance plans in a health maintenance organisation. New England Journal of Medicine 3;339(10): 673-679. | Out of scope |
| Cutt H, Giles-Corti B, Knuiman M et al. (2007) Dog ownership, health and physical activity: A critical review of the literature. Health & Place 13(1): 261-272. | SR - but mainly X-sectional studies |
| Dalziel K, Segal L, Elley CR. (2006) Cost utility analysis of physical activity counselling in general practice. Australian and New Zealand Journal of Public Health 30(1): 57-63. | Not SR - review 3? |
| Damschroder LJ, Lutes LD, Goodrich DE et al. (2010) A small-change approach delivered via telephone promotes weight loss in veterans: Results from the ASPIRE-VA pilot study. Patient Education and Counseling 79(2): 262-266. | Not SR - review 3? |
| Danaei G, Pan A, Hu FB et al. (2013) Hypothetical midlife interventions in women and risk of type 2 diabetes. Epidemiology 24(1): 122-128. | Not SR |
| Daniels R, van Rossum E, de Witte L et al. (2008) Interventions to prevent disability in frail community-dwelling elderly: a systematic review. BMC Health Services Research 30;8:278. | SR, older people only |
| Darmon N, Drewnowski A. (2008) Does social class predict diet quality? American Journal of Clinical Nutrition 87(5): 1107-1117. | Not SR, may be useful primary studies? |
| Dauchet L, Amouyel P, Dallongeville J. (2005) Fruit and vegetable consumption and risk of stroke: a meta-analysis of cohort studies. Neurology 65(8): 1193-1197. | SR - review 2? |
| Davidson PL, Andersen RM, Wyn R et al. (2004) A framework for evaluating safety-net and other community-level factors on access for low-income populations. Inquiry-the Journal of Health Care Organization Provision and Financing 41(1): 21-38 | Not SR, not midlife primary |
| Daviglus ML, Lloyd-Jones DM, Pirzada A. (2006) Preventing cardiovascular disease in the 21st century: Therapeutic and preventive implications of current evidence. American Journal of Cardiovascular Drugs 6(2): 87-101. | SR - review 3? |
| Daviglus ML, Plassman BL, Pirzada A et al. (2011) Risk factors and preventive interventions for alzheimer disease: State of the science. Archives of Neurology 68(9): 1185-1190. | SR - review 2? |
| Dawel A, Anstey KJ. (2011) Interventions for midlife smoking cessation: a literature review. Australian Psychologist 46(3): 190-195. | Not a SR, source of primary studies review 1? |
| de Sherbinin A, Vanwey L, McSweeney K et al. (2008) Rural household demographics, livelihoods and the environment. Global Environmental Change-Human and Policy Dimensions 18(1): 38-53. | Out of scope |
| de Viron S, Van der Heyden J, Ambrosino E et al. (2012) Impact of genetic notification on smoking cessation: Systematic review and pooled-analysis. PLoS ONE 7(7): e40230. | SR review 1 and 3 |
| Delavari M, Sønderlund AL, Swinburn B et al. (2013) Acculturation and obesity among migrant populations in high income countries--a systematic review. BMC Public Health 10;13:458. | SR review 1 |
| Dennis S, Williams A, Taggart J et al. (2012) Which providers can bridge the health literacy gap in lifestyle risk factor modification education: a systematic review and narrative synthesis. BMC Family Practice 18;13:4. | SR, review 1 |
| Desroches S, Lapointe A, Ratté S et al. (2013) Interventions to enhance adherence to dietary advice for preventing and managing chronic diseases in adults. Cochrane Database of Systematic Reviews 2 28;2:CD008722. | SR interventions for diet adherence but includes people with existing chronic disease |
| DiClemente CC, Delahanty JC, Fiedler RM. (2010) The journey to the end of smoking a personal and population perspective. American Journal of Preventive Medicine 38:3: S418-S428. | Consider rev 1 primary, not midlife |
| Di Noia J, Prochaska JO. (2010) Dietary stages of change and decisional balance: a meta-analytic review. American Journal of Health Behavior 34(5): 618-632. | Not SR, application of model |
| Dickens AP, Richards SH, Greaves CJ et al. (2011) Interventions targeting social isolation in older people: a systematic review. BMC Public Health 15;11:647. | Review 3? |
| Dickson-Spillmann M, Siegrist M. (2011) Consumers' knowledge of healthy diets and its correlation with dietary behaviour. Journal of Human Nutrition and Dietetics 24(1): 54-60. | Not a SR, primary study review 1, not midlife? |
| Diedrick MJ, Greaves K, Slavin J.  (2011) The effect of fiber ingredients on satiety and food intake: A systematic review. FASEB Journal 25, 588-12. | Out of scope |
| Diep L, Kwagyan J, Kurantsin-Mills J et al. (2010) Association of physical activity level and stroke outcomes in men and women: a meta-analysis. Journal of Women’s Health 19(10): 1815-1822. | Review 2? |
| Dimech AS, Seiler R. (2011) Extra-curricular sport participation: A potential buffer against social anxiety symptoms in primary school children. Psychology of Sport and Exercise 12(4): 347-354. | Children |
| Dimich-Ward H, Beking K, DyBuncio A et al. (2012) Occupational exposure influences on gender differences in respiratory health. Lung 190(2): 147-154. | Not a SR |
| Dinas PC, Koutedakis Y, Flouris AD. (2011) Effects of exercise and physical activity on depression. Irish Journal of Medical Science 180(2): 319-325. | In people with existing depression |
| Dinour LM, Bergen D, Yeh MC. (2007) The food insecurity-obesity paradox: A review of the literature and the role food stamps may play. Journal of the American Dietetic Association 107(11): 1952-1961. | Not SR, X-sectional |
| DiPietro L. (2001) Physical activity in aging: Changes in patterns and their relationship to health and function. Journals of Gerontology Series a-Biological Sciences and Medical Sciences 56: 13-22. | Not SR, primary for rev 1? Not midlife |
| Dishman RK, Vandenberg RJ, Motl RW et al. (2010) Using constructs of the transtheoretical model to predict classes of change in regular physical activity: A multi-ethnic longitudinal cohort study. Annals of Behavioral Medicine 40(2): 150-163. | Primary rev 1? Not midlife |
| Doman LCH, Roux A. (2010). The causes of loneliness and the factors that contribute towards it - a literature review. Tydskrif Vir Geesteswetenskappe 50(2): 216-228. | Afrikaans language |
| Doolan DM, Froelicher ES. (2006) Efficacy of smoking cessation intervention among special populations - Review of the Literature From 2000 to 2005. Nursing Research 55(4): S29-S37. | Not a SR, rev 3? |
| Drewnowski A, Monsen E, Birkett D et al. (2003) Health screening and health promotion programs for the elderly. Disease Management & Health Outcomes 11(5): 299-309. | Not SR, elderly only |
| Drogan D, Sheldrick AJ, Schütze M et al. (2012) Alcohol consumption, genetic variants in alcohol deydrogenases, and risk of cardiovascular diseases: a prospective study and meta-analysis. PLoS One 7(2): e32176. | SR - rev 2? |
| Du H, van der A DL, van Bakel MM et al. (2009) Dietary glycaemic index, glycaemic load and subsequent changes of weight and waist circumference in European men and women. International Journal of Obesity 33(11): 1280-1288. | Not SR, cohort study rev 2 |
| Dubbert PM, Carithers T, Sumner AE et al. (2002) Obesity, physical inactivity, and risk for cardiovascular disease. American Journal of the Medical Sciences 324(3): 116-126. | Out of scope |
| Dunn C, Deroo L, Rivara FP. (2001) The use of brief interventions adapted from motivational interviewing across behavioral domains: a systematic review. Addiction 96(12): 1725-1742. | Out of scope |
| Dunn AL, Trivedi MH, O'Neal HA. (2001) Physical activity dose-response effects on outcomes of depression and anxiety. Medicine and Science in Sports and Exercise 33(6 Suppl): S587-s597. | Out of scope |
| Dunsky A, Netz Y. (2012) Physical activity and sport in advanced age: is it risky? a summary of data from articles published between 2000-2009. Current Aging Science 5(1): 66-71. | SR but sports injuries |
| Durkin S, Brennan E, Wakefield M (2012) Mass media campaigns to promote smoking cessation among adults: an integrative review. Tobacco Control 21(3): 127-138. | SR but mass media campaigns - review 3? |
| Eakin E. (2001) Promoting physical activity among middle-aged and older adults in health care settings. Journal of Aging and Physical Activity 9: S29-S37. | Source of primary studies rev 3? |
| Eakin EG, Lawler SP, Vandelanotte C et al. (2007) Telephone interventions for physical activity and dietary behavior change: a systematic review. American Journal of Preventive Medicine 32(5): 419-34. | SR rev 3? |
| Ebrahim S, Taylor F, Ward K et al. (2011) Multiple risk factor interventions for primary prevention of coronary heart disease. Cochrane Database of Systematic Reviews 1: CD001561. | Cochrane SR, health promotion limited use in gen pop? |
| Edwardson CL, Gorely T, Davies MJ et al. (2012) Association of sedentary behaviour with metabolic syndrome: a meta-analysis. PLoS One 7(4): e34916. | SR Rev 2? |
| Einecke D. (2005) Explosion of cardiovascular risks in family practice. [German] Explosion kardiovaskularer Risiken in der Hausarztpraxis. MMW Fortschritte der Medizin 147(42): 1. | Out of scope |
| Ekelund U, Besson H, Luan J et al. (2011) Physical activity and gain in abdominal adiposity and body weight: prospective cohort study in 288,498 men and women. American Journal of Clinical Nutrition 93(4): 826-835. | Primary rev 2? |
| Ekkekakis P. (2009) Let them roam free? Physiological and psychological evidence for the potential of self-selected exercise intensity in public health. Sports Medicine 39(10): 857-888. | Out of scope |
| Elder SJ, Roberts SB. (2007) The effects of exercise on food intake and body fatness: A summary of published studies. Nutrition Reviews 65(1): 1-19 | Rev 2? |
| Elfeddali I, Bolman C, Candel MJ et al. (2012) The role of self-efficacy, recovery self-efficacy, and preparatory planning in predicting short-term smoking relapse. British Journal of Health Psychology 17(1): 185-201. | Review 1 primary study, not midlife? |
| Elo IT. (2009) Social class differentials in health and mortality: patterns and explanations in comparative perspective. Annual Review of Sociology 35: 553-572. | Out of scope |
| El-Shikh H, Fahmy E, Michael VS et al. (2004) Life events and addiction: A review of literature. European Journal of Psychiatry 18(3): 163-170. | Out of scope |
| Emberson JR, Shaper AG, Wannamethee SG et al. (2005).Alcohol intake in middle age and risk of cardiovascular disease and mortality: Accounting for intake variation over time. American Journal of Epidemiology 161(9): 56-863. | Rev 2 primary? |
| Esposito K, Kastorini CM, Panagiotakos DB et al. (2011) Mediterranean diet and weight loss: meta-analysis of randomized controlled trials. Metabolic Syndrome & Related Disorders 9(1): 1-12. | SR rev 3? |
| Estabrooks PA, Gyurcsik NC. (2003) Evaluating the impact of behavioral interventions that target physical activity: issues of generalizability and public health. Psychology of Sport and Exercise 4(1): 41-55. | Not SR, translation of research to pract |
| Estabrooks PA, Glasgow RE. (2006) Translating effective clinic-based physical activity interventions into practice. American Journal of Preventive Medicine 31(4): S45-S56. | Not SR, translation of research to pract |
| Etter JF, Stapleton JA. (2006) Nicotine replacement therapy for long-term smoking cessation: A meta-analysis. Tobacco Control 15(4): 280-285. | Rev 3? |
| Etter JF. (2009) Comparing computer-tailored, internet-based smoking cessation counseling reports with generic, untailored reports: a randomized trial. Journal of Health Communication 14(7): 646-657. | Out of scope |
| Evenhuis H, Henderson CM, Beange H et al. (2000) Healthy ageing - adults with intellectual disabilities: physical health issues. Journal of Applied Research in Intellectual Disabilities 14(3): 175-194. | Out of scope |
| Evers A, Klusmann V, Schwarzer R et al. (2011) Improving cognition by adherence to physical or mental exercise: A moderated mediation analysis. Aging & Mental Health 15(4): 446-455. | Not SR, rev 3? |
| Evers A, Klusmann V, Schwarzer R et al. (2012) Adherence to physical and mental activity interventions: Coping plans as a mediator and prior adherence as a moderator. British Journal of Health Psychology 17(3): 477-491. | Not SR, primary study review 1? Not midlife |
| Eyles HC, Mhurchu CN. (2009) Does tailoring make a difference? A systematic review of the long-term effectiveness of tailored nutrition education for adults. Nutrition Reviews 67(8): 464-480. | SR but is it HB? |
| Fagard RH. (2001) Exercise characteristics and the blood pressure response to dynamic physical training. Medicine & Science in Sports & Exercise 33(6 Suppl): S484-492; discussion S493-484. | SR - rev 3? |
| Fagard RH. (2005) Effects of exercise, diet and their combination on blood pressure. Journal of Human Hypertension 19(Suppl 3): S20-24. | SR- rev 3? |
| Fagard RH. (2006) Exercise is good for your blood pressure: Effects of endurance training and resistance training. Clinical and Experimental Pharmacology and Physiology 33(9): 853-856. | SR - rev 3? |
| Fallon EA, Hausenblas HA, Nigg CR. (2005) The transtheoretical model and exercise adherence: examining construct associations in later stages of change. Psychology of Sport and Exercise 6(6): 629-641. | Out of scope |
| Faulkner GE, Grootendorst P, Nguyen VH et al. (2011) Economic instruments for obesity prevention: Results of a scoping review and modified delphi survey. The International Journal of Behavioral Nutrition and Physical Activity 6(8): 109. | SR? Food taxes and subsidies effect on obesity/PA/diet |
| Faulkner GP, Pourshahidi LK, Wallace JM et al. (2012) Serving size guidance for consumers: is it effective? Proceedings of the Nutrition Society 71(4): 610-621. | Not a SR |
| Faulkner M. (2013) A systematic review of aerobic exercise interventions to prevent the development of Type 2 diabetes in adults with intermediate hyperglycaemia. Diabetic Medicine 30: 108. | SR Preconditions hyperglycaemia to diabetes |
| Feinstein RE, Feinstein MS. (2001) Psychotherapy for health and lifestyle change. Journal of Clinical Psychology 57(11): 1263-1275. | Not a SR |
| Ferreira ML, Sherrington C, Smith K et al. (2012) Physical activity improves strength, balance and endurance in adults aged 40-65 years: a systematic review. Journal of Physiotherapy 58(3): 145-56. | Rev 3? |
| Ferri M, Amato L, Davoli M. (2006) Alcoholics Anonymous and other 12-step programmes for alcohol dependence. Cochrane Database of Systematic Reviews 19(3): CD005032. | Cochrane SR - review 3 |
| Fichtenberg CM, Glantz SA. (2002) Effect of smoke-free workplaces on smoking behavior: systematic review. BMJ 325(7357): 188-191. | SR smoke free workplaces, includes X-sectional studies |
| Finkler E, Heymsfield SB, St-Onge PM. (2012) Rate of weight loss can be predicted by patient characteristics and intervention strategies. Journal of the Academy of Nutrition & Dietetics 112(1): 75-80. | Review but not systematic? Factors associated with weight loss in interventions |
| Finlayson G, King N, Blundell J. (2008) The role of implicit wanting in relation to explicit liking and wanting for food: Implications for appetite control. Appetite 50(1): 120-127. | Not SR, not primary study - not pop level? |
| Fischbacher CM, Hunt S, Alexander L. (2004) How physically active are South Asians in the United Kingdom? A literature review. Journal of Public Health 26(3): 250-258. | Out of scope |
| Fitzgibbon ML, Tussing-Humphreys LM, Porter JS et al. (2012) Weight loss and African-American women: a systematic review of the behavioural weight loss intervention literature. Obesity Reviews 13(3): 193-213. | SR, behavioural int for weight loss in African American women |
| Fjeldsoe B, Neuhaus M, Winkler E et al. (2011) Systematic review of maintenance of behavior change following physical activity and dietary interventions. Health Psychology 30(1): 99-109. | SR maintenance of behaviour change (from interventions) |
| Fleming MF, Mundt MP, French MT et al. (2002) Brief physician advice for problem drinkers: long-term efficacy and benefit-cost analysis. Alcoholism: Clinical and Experimental Research 26(1): 36-43. | Not a SR, RCT of brief phys advice, primary study review 1? Not midlife |
| Fleming P, Godwin M. (2008) Lifestyle interventions in primary care: systematic review of randomized controlled trials. Canadian Family Physician 54(12): 1706-1713. | SR - Review 3, lifestyle interventions in primary care, compares providers |
| Fleury J, Keller C, Perez A et al. (2009) The role of lay health advisors in cardiovascular risk reduction: a review. American Journal of Community Psychology 44(1-2): 28-42. | SR? Quality of review, lay health advisors? |
| Flodgren G, Deane K, Dickinson HO et al. (2010) Interventions to change the behaviour of health professionals and the organisation of care to promote weight reduction in overweight and obese adults. Cochrane Database of Systematic Reviews 17(3): CD000984. | Cochrane SR - review 3? Ints to change behaviour of health profs. |
| Floyd MF, Spengler JO, Maddock JE et al. (2008) Park-based physical activity in diverse communities of two US cities - An observational study. American Journal of Preventive Medicine 34(4): 299-305. | Primary study review 1? Not midlife |
| Fogelholm M, Kukkonen-Harjula K. (2000) Does physical activity prevent weight gain - a systematic review. Obesity Reviews 1(2): 95-111. | SR - rev 3? |
| Foley L, Maddison R, Jones Z et al. (2011) Comparison of two modes of delivery of an exercise prescription scheme. New Zealand Medical Journal 1338: 44-54. | Not SR, primary study rev 3? Not midlife |
| Forouhi NG, Sharp SJ, Du H et al. (2009) Dietary fat intake and subsequent weight change in adults: results from the European Prospective Investigation into Cancer and Nutrition cohorts. American Journal of Clinical Nutrition 90(6): 1632-1641. | Review 2? |
| Forsman AK, Schierenbeck I, Wahlbeck K. (2011) Psychosocial interventions for the prevention of depression in older adults: Systematic review and meta-analysis. Journal of Aging and Health 23(3): 387-416. | SR - rev 3? |
| Foster C, Hillsdon M, Thorogood M et al. (2005) Interventions for promoting physical activity. Cochrane Database of Systematic Reviews 1. | SR - rev 3? |
| Franco, M, Ordunez P, Caballero B et al. (2007) Impact of energy intake, physical activity, and population-wide weight loss on cardiovascular disease and diabetes mortality in Cuba, 1980-2005. American Journal of Epidemiology 166(12): 1374-1380. | Not SR, primary for rev 2? |
| Fratiglioni L, Paillard-Borg S, Winblad B. (2004) An active and socially integrated lifestyle in late life might protect against dementia. Lancet Neurology 3(6): 343-353. | Not sure if SR - lit search? Meta-analysis and relevant HB |
| Freak-Poli RL, Cumpston M, Peeters A et al. (2013) Workplace pedometer interventions for increasing physical activity. Cochrane Database of Systematic Reviews 30(4): CD009209. | Rev 3? |
| French DP, Stevenson A, Michie S. (2012) An intervention to increase walking requires both motivational and volitional components: A replication and extension. Psychology Health & Medicine 17(2): 127-135. | Not SR, primary rev 1? |
| Frerichs W, Kaltenbacher E, van de Leur JP et al. (2012) Can physical therapists counsel patients with lifestyle-related health conditions effectively? A systematic review and implications. Physiotherapy Theory & Practice 28(8): 571-587. | SR, physical therapists supplying counselling |
| Frost H, Haw S, Frank J. (2012) Interventions in community settings that prevent or delay disablement in later life: an overview of the evidence." Quality in Ageing and Older Adults 13(3): 212-230. | SR, older people > 50, about interventions not barriers facilitators. |
| Frost SS, Goins RT, Hunter RH et al. (2010) Effects of the built environment on physical activity of adults living in rural settings. American Journal of Health Promotion 24(4): 267-83. | SR, built environment |
| Furlow EA, Anderson JW. (2009) A systematic review of targeted outcomes associated with a medically supervised commercial weight-loss program. Journal of the American Dietetic Association 109(8): 1417-1421 | SR - rev 3? |
| Galani C, Schneider H. (2007) Prevention and treatment of obesity with lifestyle interventions: review and meta-analysis. International Journal of Public Health 52(6): 348-359. | Not a SR - rev 3 |
| Galani C, Schneider H, Rutten FF. (2007) Modelling the lifetime costs and health effects of lifestyle intervention in the prevention and treatment of obesity in Switzerland. International Journal of Public Health 52(6): 372-382 | SR - rev 3? |
| Gale CR, Sayer AA, Cooper C et al. (2011) Factors associated with symptoms of anxiety and depression in five cohorts of community-based older people: the HALCyon (Healthy Ageing across the Life Course) Programme. Psychological Medicine 41(10): 2057-2073. | Possible primary for rev2 but x-sectional |
| Gallicchio L, Matanoski G, Tao XG et al. (2006) Adulthood consumption of preserved and nonpreserved vegetables and the risk of nasopharyngeal carcinoma: a systematic review. International Journal of Cancer 119(5): 1125-1135. | Out of scope |
| Gao Y, Griffiths S, Chan EY. (2007) Community-based interventions to reduce overweight and obesity in China: a systematic review of the Chinese and English literature. Journal of Public Health 30(4): 436-448. | SR - rev 3? |
| Gardner MM, Robertson MC, Campbell AJ. (2000) Exercise in preventing falls and fall related injuries in older people: a review of randomised controlled trials. British Journal of Sports Medicine 34(1): 7-17. | Out of scope |
| Gates N, Valenzuela M. (2010) Cognitive exercise and its role in cognitive function in older adults. Current Psychiatry Reports 12(1): 20-27. | Not a SR , review, |
| Geleijnse JM, Kok FJ, Grobbee DE. (2004) Impact of dietary and lifestyle factors on the prevalence of hypertension in Western populations. European Journal of Public Health 14(3): 235-239. | Review 2? |
| George A, Fleming P. (2004) Factors affecting men’s help-seeking in the early detection of prostate cancer: Implications for health promotion. Journal of Men’s Health & Gender 1(4): 345-352. | Out of scope |
| George ES, Kolt GS, Duncan MJ et al. (2012) A review of the effectiveness of physical activity interventions for adult males. Sports Medicine 42(4): 281-300. | SR, rev 3? |
| Gething L, Gridley H, Browning C et al. (2003) The role of psychologists in fostering the wellbeing of older Australians. Australian Psychologist 38(1): 1-10. | Out of scope |
| Gibson S. (2008) Sugar-sweetened soft drinks and obesity: A systematic review of the evidence from observational studies and interventions. Nutrition Research Reviews 21(2): 134-147. | SR, rev 2? |
| Gillies CL, Lambert PC, Abrams KR et al. (2008) Different strategies for screening and prevention of type 2 diabetes in adults: cost effectlveness analysis. BMJ 24;336(7654): 1180-1185. | Out of scope |
| Gillison FB, Skevington SM, Sato A et al. (2009) The effects of exercise interventions on quality of life in clinical and healthy populations; a meta-analysis. Social Science & Medicine 68(9): 1700-1710. | Out of scope |
| Gittelsohn J, Kim EM, He S et al. (2013) A food store-based environmental intervention is associated with reduced BMI and improved psychosocial factors and food-related behaviors on the Navajo nation." Journal of Nutrition 143(9): 1494-1500 | Not SR primary study for review 1? Not midlife |
| Gokah TK, Gumpo R. (2010) Enabling and empowering-the need for an integrated approach to address hypertension among African adults. Health Education Research 25(3): 510-518. | Not a SR |
| Goldsby TU. (2013) The immediate and long lasting effects of aerobic exercise: A meta-analysis among ethnically diverse adults. Dissertation Abstracts International: Section B: The Sciences and Engineering 74(1-B E). | Out of scope |
| González CA, Pera G, Agudo A et al. (2006) Fruit and vegetable intake and the risk of stomach and oesophagus adenocarcinoma in the European Prospective Investigation into Cancer and Nutrition (EPIC-EURGAST). International Journal of Cancer 118(10): 2559-2566. | Primary rev 1? Not midlife |
| González D, Nazmi A, Victora CG. (2009) Childhood poverty and abdominal obesity in adulthood: a systematic review. Cadernos De Saude Publica 25: S427-S440. | Out of scope |
| Goode AD, Owen N, Reeves MM et al. (2012) Translation from research to practice: community dissemination of a telephone-delivered physical zctivity and dietary behavior change intervention. American Journal of Health Promotion: 26(4) 253-259. | Case study, translation from research to practice |
| Goode, AD, Reeves MM, Eakin EG. (2012) Telephone-delivered interventions for physical activity and dietary behavior change: an updated systematic review. American Journal of Preventive Medicine 42(1): 81-8. | SR, telephone ints |
| Gould DJ, Pearce C, James T. (2000) The role of the practice nurse in smoking cessation. Clinical Effectiveness in Nursing, Edinburgh 4(4): 152-160. | Not SR, 1y study |
| Gourlan MJ, Trouilloud DO, Sarrazin PG. (2011) Interventions promoting physical activity among obese populations: a meta-analysis considering global effect, long-term maintenance, physical activity indicators and dose characteristics. Obesity Reviews 12(7): E633-E645. | SR, some info about HB, intervention |
| Grace C, Begum R, Subhani S et al. (2008) Prevention of type 2 diabetes in British Bangladeshis: Qualitative study of community, religious, and professional perspectives. BMJ 4:337:a1931 | Primary study review 1?Not midlife |
| Gray L, Hart CL, Smith GD et al. (2010) What is the predictive value of established risk factors for total and cardiovascular disease mortality when measured before middle age? Pooled analyses of two prospective cohort studies from Scotland. European Journal of Cardiovascular Prevention & Rehabilitation 17(1): 106-112. | Primary study for review 2? |
| Greaves CJ, Sheppard KE, Abraham C et al. (2011) Systematic review of reviews of intervention components associated with increased effectiveness in dietary and physical activity interventions. BMC Public Health 18;11:119. | Primary study for 3? |
| Grzywacz JG, Marks NF. (2000) Family, work, work-family spillover, and problem drinking during midlife. Journal of Marriage and the Family 62(2): 336-348. | Not SR, relevant primary study for review 1? Not midlife |
| Gudzune K, Hutfless S, Maruthur N et al. (2013) Strategies to prevent weight gain in workplace and college settings: A systematic review. Preventive Medicine 57(4): 268-277. | SR, settings? Interventions |
| Guérin E, Bales E, Sweet S et al. (2012) A meta-analysis of the influence of gender on self-determination theory's motivational regulations for physical activity. Canadian Psychology/Psychologie canadienne 53(4): 291-300. | Out of scope |
| Guerin E. (2012) Disentangling vitality, well-being, and quality of life: a conceptual examination emphasizing their similarities and differences with special application in the physical sctivity domain. Journal of Physical Activity & Health 9(6): 896-908. | Out of scope |
| Guralnik JM, Kritchevsky SB. (2010) Translating research to promote healthy aging: The complementary role of longitudinal studies and clinical trials. Journal of the American Geriatrics Society 58(Suppl 2): S337-S342. | Out of scope |
| Hackam DG, Khan NA, Hemmelgarn BR et al. (2010) The 2010 Canadian Hypertension Education Program recommendations for the management of hypertension: part 2 - therapy. Canadian Journal of Cardiology 26(5): 249-258. | Out of scope |
| Hagenfeldt K, Johansson C, Johnell O. (2003) Osteoporosis – prevention, diagnosis and treatment. The Swedish Council on Technology Assessment in Health Care. | SR - review 2? |
| Hagger MS, Chatzisarantis NLD. (2009) Integrating the theory of planned behaviour and self-determination theory in health behaviour: a meta-analysis. British Journal of Health Psychology 14(Pt 2): 275-302. | Out of scope |
| Hagger-Johnson GE, Shickle DA, Deary IJ et al. (2010) Direct and indirect pathways connecting cognitive ability with cardiovascular disease risk: Socioeconomic status and multiple health behaviors. Psychosomatic Medicine 72(8): 777-785. | Not SR, 1y ? |
| Halcomb E, Moujalli S, Griffiths R et al. (2007) Effectiveness of general practice nurse interventions in cardiac risk factor reduction among adults. International Journal of Evidence Based Healthcare 5(3): 269-295. | SR, nurse delivery review 3 |
| Hall NJ, Rubin G, Charnock A. (2009) Systematic review: adherence to a gluten-free diet in adult patients with coeliac disease. Alimentary Pharmacology & Therapeutics 30(4): 315-330. | Out of scope |
| Halpin HA, McMenamin SB, Rideout J et al. (2006) The costs and effectiveness of different benefit designs for treating tobacco dependence: results from a randomized trial. Inquiry 43(1): 54-65. | Not a SR, primary rev 3? |
| Hamer M, Chida Y. (2009) Physical activity and risk of neurodegenerative disease: a systematic review of prospective evidence. Psychological Medicine 39(1): 3-11. | Out of scope |
| Hardcastle SJ, Taylor AH, Bailey MP et al. (2013) Effectiveness of a motivational interviewing intervention on weight loss, physical activity and cardiovascular disease risk factors: a randomised controlled trial with a 12-month post-intervention follow-up. International Journal of Behavioral Nutrition and Physical Activity 28(10): 40. | Not a SR, primary rev 3? |
| Hardeman W, Kinmonth AL, Michie S et al. (2011) Theory of planned behaviour cognitions do not predict self-reported or objective physical activity levels or change in the ProActive trial. British Journal of Health Psychology 16(Pt 1): 135-150. | Not a SR, primary rev 1? |
| Harris R, Gamboa A, Dailey Y et al. (2012) One-to-one dietary interventions undertaken in a dental setting to change dietary behaviour. Cochrane Database of Systematic Reviews 3: CD006540. | Cochrane SR, dental setting |
| Hartley L, Igbinedion E, Holmes J et al. (2013) Increased consumption of fruit and vegetables for the primary prevention of cardiovascular diseases. Cochrane Database of Systematic Reviews 4(6): CD009874. | Out of scope |
| Haruyama Y. (2011) Outcomes of lifestyle improvement programs in the last ten years in Asia. Asian Perspectives and Evidence on Health Promotion and Education: 214-222. | Out of scope |
| Havranek EP. (2011) A Mediterranean diet reduces cardiovascular risk factors in overweight patients compared with a low-fat diet. ACP Journal Club 155(6): 2-2. | Out of scope |
| Heikkilä K, Nyberg ST, Fransson EI et al. (2012) Job strain and tobacco smoking: an individual-participant data meta-analysis of 166,130 adults in 15 European studies. PLoS One 7(7): e35463. | Not a SR, but relevant primary study for review 1? Not midlife |
| Herbert K, Plugge E, Foster C et al. (2012) Prevalence of risk factors for non-communicable diseases in prison populations worldwide: a systematic review. Lancet 379(9830): 1975-1982. | All x-sectional? |
| Hersey JC, Khavjou O, Strange LB et al. (2012) The efficacy and cost-effectiveness of a community weight management intervention: a randomized controlled trial of the health weight management demonstration. Preventive Medicine 54(1): 42-49. | Not a SR, primary rev 3? |
| Heymsfield SB, van Mierlo CA, van der Knaap HC et al. (2003) Weight management using a meal replacement strategy: meta and pooling analysis from six studies. International Journal of Obesity & Related Metabolic Disorders 27(5): 537-549. | SR, rev 3 |
| Hilbert A, Ried J, Schneider D et al. (2007) Primary prevention of adult obesity. An interdisciplinary analysis. Herz 32(7): 542-52. | German language |
| Hillsdon M, Thorogood M. (1996) A systematic review of physical activity promotion strategies. British Journal of Sports Medicine 30(2): 84-89. | Out of scope |
| Hillsdon M, Foster C, Thorogood M et al. (2005) Interventions for promoting physical activity. Cochrane Database of Systematic Reviews (1). | SR, effectivenes of individual components of interventions |
| Hind D, Scott EJ, Copeland R et al. (2010) A randomised controlled trial and cost-effectiveness evaluation of "booster" interventions to sustain increases in physical activity in middle-aged adults in deprived urban neighbourhoods. BMC Public Health 4(10): 3. | Protocol only |
| Hobbs N, Godfrey A, Lara J et al. (2013) Are behavioral interventions effective in increasing physical activity at 12 to 36 months in adults aged 55 to 70 years? A systematic review and meta-analysis. BMC Medicine 19(11): 75. | Not SR, RCT rev 3 |
| Hodge A, Almeida OP, English DR et al. (2013) Patterns of dietary intake and psychological distress in older Australians: benefits not just from a Mediterranean diet. International Psychogeriatrics 25(3): 456-466. | Not SR, primary rev 2 |
| Hoevenaar-Blom MP, Nooyens AC, Kromhout D et al. (2012) Mediterranean style diet and 12-year incidence of cardiovascular diseases: the EPIC-NL cohort study. PLoS One 7(9): e45458. | Out of scope |
| Hogg J, Lucchino R, Wang K et al. (2001) Healthy ageing - Adults with intellectual disabilities: Ageing and social policy. Journal of Applied Research in Intellectual Disabilities 14(3): 229-255. | Adults with intellectual disabilities - out of scope |
| Hollis JF, McAfee TA, Fellows JL et al. (2007) The effectiveness and cost effectiveness of telephone counselling and the nicotine patch in a state tobacco quitline. Tobacco Control 16 Suppl1:i53-i59 | Not SR, primary rev 3? |
| Hollis JL, Williams LT, Collins CE et al. (2013) Effectiveness of interventions using Motivational interviewing for dietary and physical activity modification in adults: A systematic review. JBI Database of Systematic Reviews and Implementation Reports 11(5): 1-27. | SR, rev 3? Motivational interviewing |
| Hooper R, Heinrich J, Omenaas E et al. (2010) Dietary patterns and risk of asthma: results from three countries in European Community Respiratory Health Survey-II. The British Journal of Nutrition 103(9): 1354-1365. | Asthma |
| Hooper L, Abdelhamid A, Moore HJ et al. (2012) Effect of reducing total fat intake on body weight: systematic review and meta-analysis of randomised controlled trials and cohort studies. BMJ 6;345: e7666. | SR, rev 3, athsma |
| Hopkins ME, Davis FC, Vantieghem MR et al. (2012) Differential effects of acute and regular physical exercise on cognition and affect. Neuroscience 215: 59-68. | Out of scope |
| Horne M, Tierney S. (2012) What are the barriers and facilitators to exercise and physical activity uptake and adherence among South Asian older adults: a systematic review of qualitative studies. Preventive Medicine 55(4): 276-284. | SR, rev 1 |
| Hosseinpoor AR, Bergen N, Kunst A et al. (2012) Socioeconomic inequalities in risk factors for non-communicable diseases in low-income and middle-income countries: results from the World Health Survey. BMC Public Health 28(12): 912. | Out of scope |
| Housman J, Dorman S. (2005) The Alameda County study: a systematic, chronological review. American Journal of Health Education 36(5): 302-308. | Not a formal systematic review. Not about health behaviours but more about risks - Review 2? |
| Hovick SR, Freimuth VS, Johnson-Turbes A et al. (2011) Multiple health risk perception and information processing among African Americans and Whites living in poverty. Risk Analysis 31(11): 1789-1799. | Out of scope |
| Hruschka DJ, Brewis AA. (2013) Absolute wealth and world region strongly predict overweight among women (ages 18-49) in 360 populations across 36 developing countries. Economics & Human Biology 11(3): 337-344. | Out of scope |
| Hughes MC, Girolami TM, Cheadle AD et al. (2007) A lifestyle-based weight management program delivered to employees: examination of health and economic outcomes. Journal of Occupational and Environmental Medicine 49(11): 1212-1217. | Not SR, rev 3? |
| Hutchesson MJ, Hulst J, Collins CE. (2013) Weight management interventions targeting young women: a systematic review.Journal of the Academy of Nutrition & Dietetics 113(6): 795-802. | SR, young women |
| Huxley R, Woodward M, Barzi F et al. (2005) Does sex matter in the associations between classic risk factors and fatal coronary heart disease in populations from the Asia-Pacific region? Journal of Women's Health 14(9): 820-828. | Out of scope |
| Ickes MJ, Sharma M. (2012) A systematic review of physical activity interventions in Hispanic adults. Journal Of Environmental & Public Health 2012: 156435. | SR, some HB, ethnicity |
| Ikeda N, Inoue M, Iso H et al. (2012) Adult mortality attributable to preventable risk factors for non-communicable diseases and injuries in Japan: a comparative risk assessment." PLoS Medicine 9(1):e1001160. | Out of scope |
| Im EO, Stuifbergen AK, Walker L. (2010) A situation-specific theory of Midlife Women's Attitudes Toward Physical Activity (MAPA). Nursing Outlook 58(1): 52-58. | Not SR, relevant primary study rev 1 |
| InterAct consortium. (2013) The link between family history and risk of type 2 diabetes is not explained by anthropometric, lifestyle or genetic risk factors: the EPIC-InterAct study. Diabetologia 56(1): 60–69. | Not SR, rev 2 primary |
| Jakicic JM, Tate DF, Lang W et al. (2012) Effect of a stepped-care intervention approach on weight loss in adults: a randomized clinical trial. JAMA 307(24):2617-26. | Not SR, rev 3? |
| Jané-Llopis E, Hosman C, Jenkins R et al. (2003) Predictors of efficacy in depression prevention programmes: meta-analysis. British Journal of Psychiatry 183: 384-397. | SR, not lifestyle |
| Janßen C, Sauter S, Kowalski C. (2012) The influence of social determinants on the use of prevention and health promotion services: Results of a systematic literature review. GMS Psycho-Social-Medicine 9. | Out of scope |
| Janssen KW, van der Wees PJ, Rowe BH et al. (2011) Interventions for preventing ankle ligament injuries. Cochrane Database of Systematic Reviews: CD009512. | Out of scope |
| Jebb SA. (2005) Dietary strategies for the prevention of obesity. Proceedings of the Nutrition Society 64(2): 217-227. | Not SR, primary sources rev 3 |
| Jefferson VW, Melkus GD, Spollett GR. (2000) Health-promotion practices of young black women at risk for diabetes. Diabetes Educator 26(2): 295-302. | Out of scope |
| Jenkins A, Christensen H, Walker JG et al. (2009) The effectiveness of distance interventions for increasing physical activity: a review. American Journal of Health Promotion 24(2): 102-117. | Out of scope |
| Jepson R, Clegg A, Forbes C et al. (2000) The determinants of screening uptake and interventions for increasing uptake: a systematic review. Health Technology Assessment 4(14): i-vii, 1-133. | Out of scope |
| Jeste DV, Depp CA, Vahia IV. (2010) Successful cognitive and emotional aging. World Psychiatry 9(2): 78-84. | Out of scope |
| Joffe M. (2007) Health, livelihoods, and nutrition in low-income rural systems. Food and Nutrition Bulletin 28(2): S227-S236. | Out of scope |
| Johnson CE, Danhauer JL, Bennett M et al. (2009) Systematic review of physicians' knowledge of, participation in, and attitudes toward hearing and balance screening in the elderly population. Seminars in Hearing 30(3): 193-206. | SR, screening excluded? But not much else on this |
| Johnson BT, Scott-Sheldon LA, Carey MP. (2010) Meta-synthesis of health behavior change meta-analyses. American Journal of Public Health 100(11): 2193-2198. | SR, relevant HB? |
| Johnson M, Jackson R, Guillaume L et al. (2011) Barriers and facilitators to implementing screening and brief intervention for alcohol misuse: a systematic review of qualitative evidence. Journal of Public Health 33(3): 412-421. | SR, screening? |
| Johnson F, Pratt M, Wardle J. (2012) Dietary restraint and self-regulation in eating behavior. International Journal of Obesity 36(5): 665-674. | Not a SR |
| Johnson M, Jones R, Freeman C et al. (2013) Can diabetes prevention programmes be translated effectively into real-world settings and still deliver improved outcomes? A synthesis of evidence. Diabetic Medicine 30(1): 3-15. | SR, translation res pract |
| Jokela M, Batty GD, Nyberg ST et al. (2013) Personality and all-cause mortality: Individual-participant meta-analysis of 3,947 deaths in 76,150 adults. American Journal of Epidemiology 178(5): 667-675. | Out of scope |
| Jonas DE, Garbutt JC, Amick HR et al. (2012) Behavioral Counseling After Screening for Alcohol Misuse in Primary Care: A Systematic Review and Meta-analysis for the US Preventive Services Task Force. Annals of Internal Medicine 157(9): 645-54. | SR, rev 3 |
| Kang M, Marshall SJ, Barreira TV et al. (2009) Effect of pedometer-based physical activity interventions: a meta-analysis. Research Quarterly for Exercise & Sport 80(3): 648-655. | SR - rev 3? |
| Kasparian NA, McLoone JK, Meiser B. (2009) Skin cancer-related prevention and screening behaviors: a review of the literature. Journal of Behavioral Medicine 32(5): 406-428. | SR, a lot of cross-sectional studies, quality assessment? |
| Katz DL, O'Connell M, Yeh MC et al. (2005) Public health strategies for preventing and controlling overweight and obesity in school and worksite settings: a report on recommendations of the Task Force on Community Preventive Services. Morbidity & Mortality Weekly Report. Recommendations & Reports 54(RR-10): 1-12. | SR, worksite outcomes with relevant HB separate |
| Kavanagh DJ. (2012) Online treatment for depressed drinkers: Is a therapist needed? Alcoholism: Clinical and Experimental Research 36: 79A. | Not SR, existing depression |
| Kelley GA, Kelley KS, Tran ZV. (2001) Walking and resting blood pressure in adults: a meta-analysis. Preventive Medicine 33(2 Pt 1): 120-127. | SR, rev 3? |
| Kelley GA, Kelley KS, Roberts S et al. (2011) Efficacy of aerobic exercise and a prudent diet for improving selected lipids and lipoproteins in adults: a meta-analysis of randomized controlled trials. BMC Medicine 15;9:74. | SR, rev 3? |
| Ketola E, Sipilä R, Mäkelä M. (2000) Effectiveness of individual lifestyle interventions in reducing cardiovascular disease and risk factors. Annals of Medicine 32(4): 239-251. | SR, rev 3? |
| Keyes KM, Hatzenbuehler ML, Hasin DS. (2011) Stressful life experiences, alcohol consumption, and alcohol use disorders: the epidemiologic evidence for four main types of stressors. Psychopharmacology 218(1): 1-17. | Not a SR, but v useful for primary refs |
| Khan NA, Hemmelgarn B, Padwal R et al. (2007) The 2007 Canadian Hypertension Education Program recommendations for the management of hypertension: Part 2 - Therapy. Canadian Journal of Cardiology 23(7): 539-550. | Out of scope |
| Kidd T, Peters PK. (2010) Decisional balance for health and weight is associated with whole-fruit intake in low-income young adults. Nutrition Research 30(7): 477-482. | Not SR, include as primary rev 1 |
| King DK. (2006) Individual and neighborhood effects on active lifestyles and social isolation in a sample of community-dwelling elderly: A socio-ecological study. Dissertation Abstracts International: Section B: The Sciences and Engineering 67(5-B). | Not SR, older >65y |
| King AC, Ahn DK, Oliveira BM et al. (2008) Promoting physical activity through hand-held computer technology. American Journal of Preventive Medicine 34(2): 138-142. | Not SR, primary rev 3? |
| King AC, Hekler EB, Grieco LA et al. (2013) Harnessing different motivational frames via mobile phones to promote daily physical activity and reduce sedentary behavior in aging adults.PLoS One 8(4): e2613. | Not SR, rev 3 primary? |
| Kirk JK, Bell RA, Bertoni AG et al. (2005) A qualitative review of studies of diabetes preventive care among minority patients, in the United States, 1993-2003. American Journal of Managed Care 11(6): 349-360. | SR? Lifestyle? |
| Kivimäki M, Nyberg ST, Batty GD et al. (2012) Job strain as a risk factor for coronary heart disease: A collaborative meta-analysis of individual participant data. Lancet 380 (9852): 1491-1497. | Not SR, consider for rev 2 |
| Klavestrand J, Vingard E. (2009) The relationship between physical activity and health-related quality of life: a systematic review of current evidence. Scandinavian Journal of Medicine & Science in Sports 19(3): 300-312. | Retracted |
| Klimas J, Field CA, Cullen W et al. (2012) Psychosocial interventions to reduce alcohol consumption in concurrent problem alcohol and illicit drug users. Cochrane Database of Systematic Reviews 12;2:3. | SR, also drugs |
| Knop J, Penick EC, Jensen P et al. (2003) Risk factors that predicted problem drinking in Danish men at age thirty. Journal of Studies on Alcohol 64(6): 745-755. | Not SR, outcomes age 30 |
| Koba S, Tanaka H, Maruyama C et al. (2011) Physical activity in the Japan population: association with blood lipid levels and effects in reducing cardiovascular and all-cause mortality. Journal of Atherosclerosis & Thrombosis 18(10): 833-845. | Out of scope |
| Kodama S, Tanaka S, Saito K et al. (2007) Effect of aerobic exercise training on serum levels of high-density lipoprotein cholesterol: a meta-analysis. Archives of Internal Medicine 167(10): 999-1008. | SR? rev 3? |
| Koelewijn-van Loon MS, van Steenkiste B, Ronda G et al. (2008) Improving patient adherence to lifestyle advice (IMPALA): a cluster-randomised controlled trial on the implementation of a nurse-led intervention for cardiovascular risk management in primary care. BMC Health Services Research 14;8:9. | Not SR, rev 3? |
| Koeneman MA, Verheijden MW, Chinapaw MJ et al. (2011) Determinants of physical activity and exercise in healthy older adults: A systematic review. The International Journal of Behavioral Nutrition and Physical Activity 28(8): 142. | SR< older adults > 55 but most studies >65 |
| Kondo T, Kimata A, Yamamoto K et al. (2009) Effects of Short-term Variation in Body Mass Index on Blood Pressure in Middle-aged Japanese Male Workers. Journal of Health Science 55(1): 62-71. | Out of scope |
| Koopmans B, Nielen MM, Schellevis FG et al. (2012) Non-participation in population-based disease prevention programs in general practice. BMC Public health 9;12: 856. | SR, mainly non-relevant outcomes but a few studies in dementia, diabetes. |
| Kremers S, Reubsaet A, Martens M et al. (2010) Systematic prevention of overweight and obesity in adults: a qualitative and quantitative literature analysis. Obesity Reviews 11(5): 371-379. | SR, qualitative and quant studies |
| Krishna S, Boren SA, Balas EA. (2009) Healthcare via cell phones: a systematic review. Telemedicine Journal & E-Health 15(3): 231-240. | SR, cell phones, delivery |
| Krist AH, Peele E, Woolf SH et al. (2011) Designing a patient-centered personal health record to promote preventive care. BMC Medical Informatics and Decision Making 24;11: 73. | Not SR, not primary |
| Kruger J, Buchner DM, Prohaska TR. (2009) The prescribed amount of physical activity in randomized clinical trials in older adults. The Gerontologist 49(Suppl1): S100-S107. | SR? not relevant outcomes. |
| Krummel DA, Koffman DM, Bronner Y et al. (2001) Cardiovascular health interventions in women: What works? Journal of Womens Health & Gender-Based Medicine 10(2): 117-136. | SR? rev 3? |
| Kulwicki A, Smiley K, Devine S. (2007) Smoking behavior in pregnant Arab Americans. MCN: The American Journal of Maternal/Child Nursing 32(6): 363-367. | Not SR, not midlife |
| Kumanyika S. (2008) Ethnic minorities and weight control research priorities: Where are we now and where do we need to be?"Preventive Medicine 47(6): 583-586. | Not SR, |
| Kurian AK, Cardarelli KM. (2007) Racial and ethnic differences in cardiovascular disease risk factors: a systematic review. Ethnicity & Disease 17(1): 143-152. | SR, rev 1 |
| Lakerveld J, Bot SD, Chinapaw MJ et al. (2013) Motivational interviewing and problem solving treatment to reduce type 2 diabetes and cardiovascular disease risk in real life: a randomized controlled trial. International Journal of Behavioral Nutrition and Physical Activity 19;10:47. | Primary review 3 |
| Lantz PM, Golberstein E, House JS et al. (2010) Socioeconomic and behavioral risk factors for mortality in a national 19-year prospective study of US adults. Social Science & Medicine 70(10): 1558-1566. | Out of scope |
| Latimer AE, Brawley LR, Bassett RL. (2010) A systematic review of three approaches for constructing physical activity messages: What messages work and what improvements are needed? The International Journal of Behavioral Nutrition and Physical Activity 11;7: 36. | Out of scope |
| Latimer-Cheung AE, Rhodes RE et al.  (2013) Evidence-informed recommendations for constructing and disseminating messages supplementing the new Canadian Physical Activity Guidelines. BMC Public Health 1;13: 419. | Out of scope |
| Lawlor DA, Taylor M, Bedford C et al. (2002) Is housework good for health? Levels of physical activity and factors associated with activity in elderly women. Results from the British Women's Heart and Health Study. Journal of Epidemiology and Community Health 56(6): 473-478. | C-sectional survey, not SR |
| Laws RA, St George AB, Rychetnik L et al. (2012) Diabetes prevention research: a systematic review of external validity in lifestyle interventions. American Journal of Preventive Medicine 43(2): 205-214. | *Out of scope* |
| Le C, Chongsuvivatwong V, Geater A. (2007) Contextual socioeconomic determinants of cardiovascular risk factors in rural south-west China: a multilevel analysis. BMC Public Health 5;7: 72. | Out of scope |
| Leavy JE, Bull FC, Rosenberg M et al. (2011) Physical activity mass media campaigns and their evaluation: a systematic review of the literature 2003-2010. Health Education Research 26(6): 1060-1085. | Out of scope |
| Lee Y, Back JH, Kim J et al. (2010) Systematic review of health behavioral risks and cognitive health in older adults. International psychogeriatrics 22(2): 174-187. | Rev 2 |
| Leischow SJ, Ranger-Moore J, Lawrence D. (2000) Addressing social and cultural disparities in tobacco use. Addictive Behaviors 25(6): 821-831. | Out of scope |
| Lemmens V, Oenema A, Knut IK et al. (2008) Effectiveness of smoking cessation interventions among adults: a systematic review of reviews. European Journal of Cancer Prevention 17(6): 535-544. | Out of scope |
| Lemmens VE, Oenema A, Klepp KI et al. (2008) A systematic review of the evidence regarding efficacy of obesity prevention interventions among adults. Obesity Reviews 9(5): 446-455. | Out of scope |
| Lemura LM, von Duvillard SP, Mookerjee S. (2000) The effects of physical training of functional capacity in adults: ages 46 to 90: a meta-analysis. Journal of Sports Medicine & Physical Fitness 40(1): 1-10. | Out of scope |
| Lewin SA, Babigumira SM, Bosch-Capblanch X et al. (2005) Lay health workers in primary and community health care. Cochrane Database of Systematic Reviews (1): CD004015. | SR, rev 3 |
| Li F, Fisher KJ, Bauman A et al. (2005) Neighborhood influences on physical activity in middle-aged and older adults: A multilevel perspective. Journal of Aging and Physical Activity 13(1): 87-114. | Out of scope |
| Liddell C, Morris C. (2010) Fuel poverty and human health: A review of recent evidence. Energy Policy 38(6): 2987-2997. | Out of scope |
| Lim KC, Kayser-Jones JS, Waters C et al. (2007) Aging, health, and physical activity in Korean Americans. Geriatric Nursing 28(2): 112-119. | Out of scope |
| Lindhjem H, Navrud S, Braathen NA et al. (2011) Valuing mortality risk reductions from environmental, transport, and health policies: a global meta-analysis of stated preference studies. Risk Analysis 31(9): 1381-1407. | Out of scope |
| Lindholm L. (1998) Alcohol advice in primary health care: is it a wise use of resources? Health Policy 45.1 (1998): 47-56. | Out of scope |
| Lira MT, Kunstmann S, Caballero E et al. (2006) Cardiovascular prevention and attitude of people towards behavior changes: state of the art. Revista Medica De Chile 134(2): 223-230. | Spanish |
| Lock CA, Kaner E, Heather N et al. (2006) Effectiveness of nurse-led brief alcohol intervention: a cluster randomized controlled trial. Journal of Advanced Nursing 54(4): 426-439. | Out of scope |
| Loef M, Walach H. (2013) Midlife obesity and dementia: Meta‐analysis and adjusted forecast of dementia prevalence in the United States and China.Obesity, 21(1): E51-E55. | Out of scope |
| Lombard CB, Deeks AA, Teede HJ. (2009) A systematic review of interventions aimed at the prevention of weight gain in adults. Public Health Nutrition 12(11): 2236-2246. | Out of scope |
| Loveman E, Frampton GK, Shepherd J et al. (2011) The clinical effectiveness and cost-effectiveness of long-term weight management schemes for adults: a systematic review. Health Technology Assessment 15(2): 1-182. | Out of scope |
| Luhmann M, Hofmann W, Eid M et al. (2012) Subjective well-being and adaptation to life events: a meta-analysis. Journal of Personality & Social Psychology 102(3): 592-615. | Out of scope |
| MacDonald LA, Cohen A, Baron S, Burchfiel CM. (2009) Occupation as socioeconomic status or environmental exposure? A aurvey of practice among population-based cardiovascular studies in the United States. American Journal of Epidemiology 169(12): 1411-1421 | Out of scope |
| Maes L, Van Cauwenberghe E, Van Lippevelde W et al. (2012) Effectiveness of workplace interventions in Europe promoting healthy eating: a systematic review. European Journal of Public Health 22(5): 677-683. | Out of scope |
| Makela P, Osterberg E. (2009) Weakening of one more alcohol control pillar: a review of the effects of the alcohol tax cuts in Finland in 2004. Addiction 104(4): 554-563. | Out of scope |
| Marshall T, Rouse A. (2002) Resource implications and health benefits of primary prevention strategies for cardiovascular disease in people aged 30 to 74: mathematical modelling study. BMJ 325(7357): 197. | Out of scope |
| Martin A, Sanderson K, Cocker F. (2009) Meta-analysis of the effects of health promotion intervention in the workplace on depression and anxiety symptomS. Scandinavian Journal of Work, Environment and Health 35(1): 7-18 | Out of scope |
| Masi CM, Chen HY, Hawkley LC et al. (2011) A meta-analysis of interventions to reduce loneliness. Personality & Social Psychology Review 15(3): 219-266. | Out of scope |
| McCreary DR, Sadava SW. (2000) Stress, alcohol use and alcohol-related problems: The influence of negative and positive affect in two cohorts of young adults. Journal of Studies on Alcohol 61(3): 466-474. | Out of scope |
| McEvoy CT, Temple N, Woodside JV. (2012) Vegetarian diets, low-meat diets and health: a review. Public Health Nutrition 15(12): 2287-2294. | Out of scope |
| McGuinness B, Todd S, Passmore P et al. (2009) Blood pressure lowering in patients without prior cerebrovascular disease for prevention of cognitive impairment and dementia. Cochrane Database of Systematic Reviews 7(4): CD004034. | Out of scope |
| McKenzie SK, Carter KN, Blakely T et al. (2011) Effects of childhood socioeconomic position on subjective health and health behaviours in adulthood: how much is mediated by adult socioeconomic position? BMC Public Health 29;11:269. | Out of scope |
| McLean N, Griffin S, Toney K et al. (2003) Family involvement in weight control, weight maintenance and weight-loss interventions: a systematic review of randomised trials. International Journal of Obesity & Related Metabolic Disorders 27(9): 987-1005. | Out of scope |
| McMahon S, Fleury J. (2010) Physical activity to reduce frailty risk and falls: a review of intervention research. Communicating Nursing Research 43: 494-494. | Out of scope |
| Mehta S, Dimsdale J, Nagle B et al. (2013) Worksite interventions improving lifestyle habits among Latin American adults. American Journal of Preventive Medicine 44(5): 538-542. | Out of scope |
| Melia J, Pendry L, Eiser JR et al. (2000) Evaluation of primary prevention initiatives for skin cancer: a review from a UK perspective. British Journal of Dermatology 143(4): 701-708. | Out of scope |
| Michie S, Abraham C, Whittington C et al. (2009) Effective techniques in healthy eating and physical activity interventions: A meta-regression. Health Psychology 28(6): 690-701. | Out of scope |
| Michie S, Jochelson K, Markham WA et al. (2009) Low-income groups and behaviour change interventions: a review of intervention content, effectiveness and theoretical frameworks. Journal of Epidemiology and Community Health 63(8): 610-622. | Out of scope |
| Mills AL, Messer K, Gilpin EA et al. (2009) The effect of smoke-free homes on adult smoking behavior: A review. Nicotine & Tobacco Research 11(10): 1131-1141. | Out of scope |
| Mills M, Loney P, Jamieson E et al. (2010) A primary care cardiovascular risk reduction clinic in Canada was more effective and no more expensive than usual on-demand primary care: a randomised controlled trial. Health and Social Care in the Community 18(1): 30-40. | Out of scope |
| Mills SD, Tanner LM, Adams J. (2013) Systematic literature review of the effects of food and drink advertising on food and drink-related behaviour, attitudes and beliefs in adult populations. Obesity Reviews 14(4): 303-314. | Out of scope |
| Moore CJ, Cunningham SA. (2012) Social position, psychological stress, and obesity: a systematic review. Journal of the Academy of Nutrition & Dietetics 112(4): 518-526. | Out of scope |
| Morales LS, Lara M, Kington RS et al. (2002) Socioeconomic, cultural, and behavioral factors affecting Hispanic health outcomes. Journal of Health Care for the Poor & Underserved 13(4): 477-503. | Out of scope |
| Morris BH, Bylsma LM, Rottenberg J. (2009) Does emotion predict the course of major depressive disorder? A review of prospective studies. British Journal of Clinical Psychology 48(3): 255-273. | Out of scope |
| Mountain G, Mozley C, Craig C et al. (2008) Occupational therapy led health promotion for older people: Feasibility of the lifestyle matters programme. British Journal of Occupational Therapy 71(10): 406-413. | Out of scope |
| Moyer A, Finney JW, Swearingen CE et al. (2002) Brief interventions for alcohol problems: A meta-analytic review of controlled investigations in treatment-seeking and non-treatment-seeking populations. Addiction 97(3): 279-292. | Out of scope |
| Mozaffarian D, Hao T, Rimm EB et al. (2011) Changes in diet and lifestyle and long-term weight gain in women and men. New England Journal of Medicine 364(25): 2392-2404. | Out of scope |
| Müller-Riemenschneider F, Andersohn F, Ernst S et al. (2012) Association of physical activity and atrial fibrillation. Journal of Physical Activity & Health 9(5): 605-616 | Out of scope |
| Murray J, Saxena S, Millett C et al. (2010) Reductions in risk factors for secondary prevention of coronary heart disease by ethnic group in south-west London: 10-year longitudinal study (1998-2007). Family Practice 27(4): 430-438 | Out of scope |
| Myers EF, Spence LA, Leslie B et al. (2010) Nutrition and telephone counseling: future implications for dietitians and teledietetics. Topics in Clinical Nutrition 25(2): 88-108. | Out of scope |
| Neafsey EJ, Collins MA (2011) Moderate alcohol consumption and cognitive risk. Neuropsychiatric Disease and Treatment 7(1): 465-484. | Out of scope |
| Neville LM, O'Hara B, Milat A. (2009) Computer-tailored physical activity behavior change interventions targeting adults: a systematic review. International Journal of Behavioral Nutrition & Physical Activity 3;6: 30. | Out of scope |
| Newton TL, Woodruff-Borden J, Stetson BA. (2006) Integrating mind and body: Graduate psychology education in primary behavioral health care. Journal of Clinical Psychology in Medical Settings 13(1): 3-11. | Out of scope |
| Nicholson NR. (2012) A review of social isolation: An important but underassessed condition in older adults. The Journal of Primary Prevention 33(2-3): 137-152. | Out of scope |
| Niedzwiedz CL, Katikireddi SV, Pell JP et al. (2012) Life course socio-economic position and quality of life in adulthood: a systematic review of life course models. BMC Public Health 9;12: 628. | Out of scope |
| Nordmann AJ, Suter-Zimmermann K, Bucher HC et al. (2011) Meta-analysis comparing Mediterranean to low-fat diets for modification of cardiovascular risk factors. American Journal of Medicine 124(9): 841-851. | Out of scope |
| Nyman SR, Yardley L. (2009) Web-site-based tailored advice to promote strength and balance training: an experimental evaluation. Journal of Aging & Physical Activity 17(2): 210-222. | Out of scope |
| O Flaherty M, Flores-Mateo G, Nnoaham K et al. (2012) Potential cardiovascular mortality reductions with stricter food policies in the United Kingdom of Great Britain and Northern Ireland. Bulletin of the World Health Organization 90(7): 522-531. | Out of scope |
| O'Connor R, Fix B, Celestino P et al. (2006) Financial incentives to promote smoking cessation: evidence from 11 quit and win contests. Journal of Public Health Management and Practice 12(1): 44-51. | Out of scope |
| Oguma Y, Shinoda-Tagawa T. (2004) Physical activity decreases cardiovascular disease risk in women: review and meta-analysis. American Journal of Preventive Medicine 26(5):407-18. | Out of scope |
| Oh EG, Bang SY, Hyun SS et al. (2010) Effects of a 6-month lifestyle modification intervention on the cardiometabolic risk factors and health-related qualities of life in women with metabolic syndrome. Metabolism-Clinical and Experimental 59(7): 1035-1043. | Out of scope |
| Oja P, Titze S, Bauman A et al. (2011) Health benefits of cycling: a systematic review. Scandinavian Journal of Medicine & Science in Sports 21(4): 496-509. | Out of scope |
| Oldroyd J, Burns C, Lucas P et al. (2008) The effectiveness of nutrition interventions on dietary outcomes by relative social disadvantage: a systematic review. Journal of Epidemiology and Community Health 62(7): 573-579. | Out of scope |
| Oliveira AJ, Lopes CS, de Leon AC et al. (2011) Social support and leisure-time physical activity: longitudinal evidence from the Brazilian Pro-Saude cohort study. International Journal of Behavioral Nutrition and Physical Activity 26;8:77. | Out of scope |
| Organization WH. (2001) Healthy ageing - Adults with intellectual disabilities: Summative report. Journal of Applied Research in Intellectual Disabilities 14(3): 256-275. | Out of scope |
| Osei-Assibey G, Kyrou I, Adi Y et al. (2010) Dietary and lifestyle interventions for weight management in adults from minority ethnic/non-White groups: a systematic review. Obesity Reviews 11(11): 769-776. | Out of scope |
| OShea E. (2006) Developing a healthy ageing policy for Ireland: The view from below. Health Policy 76(1): 93-105. | Out of scope |
| Ota A, Masue T, Yasuda N et al. (2010) Psychosocial job characteristics and smoking cessation: A prospective cohort study using the Demand-Control-Support and Effort-Reward Imbalance job stress models. Nicotine & Tobacco Research 12(3): 287-293 | Out of scope |
| Pampel FC, Krueger PM, Denney JT. (2010) Socioeconomic disparities in health behaviors. Annual Review of Sociology 36: 349-370. | Out of scope |
| Papaioannou A, Kennedy CC, Cranney A et al. (2009) Risk factors for low BMD in healthy men age 50 years or older: a systematic review. Osteoporosis International 20(4): 507-518. | Not SR, but v relevant for primary studies |
| Parente RC, Faerstein E, Celeste RK et al. (2008) The relationship between smoking and age at the menopause: A systematic review. Maturitas 61(4): 287-298. | Out of scope |
| Park CH. (2008) Assessing the impact of the national blueprint: Increasing physical activity among adults age 50 and older. Dissertation Abstracts International Section A: Humanities and Social Sciences 68(7-A). | Out of scope |
| Patil SR, Cates S, Morales R. (2005) Consumer food safety knowledge, practices, and demographic differences: findings from a meta-analysis. Journal of Food Protection 68(9): 1884-1894. | Out of scope |
| Pavey TG, Anokye N, Taylor AH et al. (2011) The clinical effectiveness and cost-effectiveness of exercise referral schemes: a systematic review and economic evaluation. Health Technology Assessment 15(44): 1-254. | Out of scope |
| Pavia M, Pileggi C, Nobile CG et al. (2006) Association between fruit and vegetable consumption and oral cancer: a meta-analysis of observational studies. American Journal of Clinical Nutrition 83(5): 1126-1134. | Out of scope |
| Peels DA, van Stralen MM, Bolman C et al. (2012) Development of web-based computer-tailored advice to promote physical activity among people older than 50 years. Journal of Medical Internet Research 14(2). | Out of scope |
| Perez A, Fleury J, Keller C. (2010) Review of intervention studies promoting physical activity in Hispanic women. Western Journal of Nursing Research 32(3): 341-362. | Out of scope |
| Pérez-Escamilla R, Hromi-Fiedler A, Vega-López S et al. (2008) Impact of peer nutrition education on dietary behaviors and health outcomes among Latinos: a systematic literature review. Journal of Nutrition Education & Behavior 40(4): 208-225. | Out of scope |
| Perkins KA, Scott J. (2008) Sex differences in long-term smoking cessation rates due to nicotine patch. Nicotine & Tobacco Research 10(7): 1245-1250. | Out of scope |
| Perry KJ, Hickson M, Thomas J. (2011) Factors enabling success in weight management programmes: systematic review and phenomenological approach. Journal of Human Nutrition & Dietetics 24(3): 301-302. | Out of scope |
| Peterson J, Atwood JR, Yates B. (2002) Key elements for church-based health promotion programs: outcome-based literature review. Public Health Nursing 19(6): 401-411. | Out of scope |
| Pierce JP, Gilpin EA. (2001) News media coverage of smoking and health is associated with changes in population rates of smoking cessation but not initiation. Tobacco Control 10(2): 145-153. | Not SR |
| Pinquart M, Sorensen S. (2000) Influences of socioeconomic status, social network, and competence on subjective well-being in later life: a meta-analysis. Psychology & Aging 15(2): 187-224. | Out of scope |
| Pinquart M, Sorensen S. (2001) Influences on loneliness in older adults: A meta-Analysis. Basic and Applied Social Psychology 23(4): 245-266. | Out of scope |
| Pinquart M, Sorensen S. (2001) Gender differences in self-concept and psychological well-being in old age: A meta-analysis. Journals of Gerontology - Series B Psychological Sciences and Social Sciences 56(4): 195-213. | Not a SR but useful for primary studies |
| Pinquart M, Sorensen S. (2003) Risk factors for loneliness in adulthood and old age - a meta-analysis. Nova Science Publishers 111-143. | Out of scope |
| Pirozzo S, Summerbell C, Cameron C et al. (2002) Advice on low-fat diets for obesity. Obesity Reviews 4(2): 83-90. | Out of scope |
| Poikolainen K. (1999) Effectiveness of brief interventions to reduce alcohol intake in primary health care populations: a meta-analysis. Preventive Medicine 28(5): 503-509. | Out of scope |
| Pollitt RA, Rose KM, Kaufman JS. (2005) Evaluating the evidence for models of life course socioeconomic factors and cardiovascular outcomes: a systematic review. BMC Public Health 20;5:7. | Out of scope |
| Popkin BM, Gordon-Larsen P. (2004) The nutrition transition: worldwide obesity dynamics and their determinants. International Journal of Obesity (2004) 28, S2–S9. | Out of scope |
| Popkin BM, Duffey K, Gordon-Larsen P. (2005) Environmental influences on food choice, physical activity and energy balance. Physiology & Behavior 86(5): 603-613. | Not SR |
| Popkin BM, Kim S, Rusev ER et al. (2006) Measuring the full economic costs of diet, physical activity and obesity-related chronic diseases. Obesity Reviews 7(3): 271-293. | Not a SR |
| Powell LM, Chriqui JF, Khan T et al. (2013) Assessing the potential effectiveness of food and beverage taxes and subsidies for improving public health: a systematic review of prices, demand and body weight outcomes. Obesity Reviews 14(2): 110-128. | Out of scope |
| Primack BA, Carroll MV, McNamara M et al. (2012) Role of video games in improving health-related outcomes: a systematic review. American Journal of Preventive Medicine 42(6): 630-638. | Quality of SR? |
| Pronk NP, Boucher JL, Gehling E et al. (2002) A platform for population-based weight management: Description of a health plan-based integrated systems approach. American Journal of Managed Care 8(10): 847-857. | Out of scope |
| Prospective Studies Collaboration. (2007) Blood cholesterol and vascular mortality by age, sex, and blood pressure: a meta-analysis of individual data from 61 prospective studies with 55 000 vascular deaths. Lancet 370: 1829–39. | Out of scope |
| Puetz TW, O'Connor PJ, Dishman RK. (2006) Effects of chronic exercise on feelings of energy and fatigue: a quantitative synthesis. Psychological Bulletin 132(6): 866-876. | Out of scope |
| Puhl R, Luedicke J, Peterson JL. (2013) Public reactions to obesity-related health campaigns: a randomized controlled trial. American Journal of Preventive Medicine 45(1): 36-48. | Out of scope |
| Purshouse RC, Meier PS, Brennan A et al. (2010) Estimated effect of alcohol pricing policies on health and health economic outcomes in England: An epidemiological model. The Lancet 375(9723): 1355-1364 | Out of scope |
| Quintiliani LM, Carbone ET. (2005) Impact of diet-related cancer prevention messages written with cognitive and affective arguments on message characteristics, stage of change, and self-efficacy." ournal of Nutrition Education & Behavior 37(1): 12-19. | Out of scope |
| Rabin BA, Glasgow RE, Kerner JF et al. (2010) Dissemination and implementation research on community-based cancer prevention: a systematic review. American Journal of Preventive Medicine 38(4): 443-456. | Out of scope |
| Ramond A, Bouton C, Richard I et al. (2011) Psychosocial risk factors for chronic low back pain in primary care-a systematic review. Family Practice 28(1): 12-21. | Out of scope |
| Ranney L, Melvin C, Lux L et al. (2006) Systematic review: smoking cessation intervention strategies for adults and adults in special populations. Annals of Internal Medicine 145(11): 845-856. | Out of scope |
| Ratey JJ, Loehr JE. (2011) The positive impact of physical activity on cognition during adulthood: A review of underlying mechanisms, evidence and recommendations. Reviews in the Neurosciences 22(2): 171-185. | Out of scope |
| Rauner A, Mess F, Woll A. (2013) The relationship between physical activity, physical fitness and overweight in adolescents: a systematic review of studies published in or after 2000. BMC Pediatrics 1;13:19. | Out of scope |
| Reed SD, Li Y, Oddone EZ et al. (2010) Economic evaluation of home blood pressure monitoring with or without telephonic behavioral self-management in patients with hypertension. American Journal of Hypertension 23(2): 142-148. | Adolescents |
| Rees K, Dyakova M, Ward K et al. (2013) Dietary advice for reducing cardiovascular risk. Cochrane Database of Systematic Reviews 28(3): CD002128. | Out of scope |
| Rees K, Hartley L, Flowers N et al. (2013) Mediterranean dietary pattern for the primary prevention of cardiovascular disease. Cochrane Database of Systematic Reviews 12(8): CD009825. | Out of scope |
| Reichstadt J, Sengupta G, Depp CA et al. (2010) Older adults' perspectives on successful aging: Qualitative interviews. The American Journal of Geriatric Psychiatry 18(7): 567-575. | Adolescents |
| Rhodes SD, Foley KL, Zometa CS et al. (2007) Lay health advisor interventions among Hispanics/Latinos: a qualitative systematic review. American Journal of Preventive Medicine 33(5): 418-27. | Not SR |
| Rhodes RE, Blanchard CM, Bellows KH. (2008) Exploring cues to sedentary behaviour as processes of physical activity action control. Psychology of Sport and Exercise 9(2): 211-224. | Out of scope |
| Rhodes RE, Fiala B, Conner M. (2009) A review and meta-analysis of affective judgments and physical activity in adult populations. Annals of Behavioral Medicine 38(3): 180-204. | Out of scope |
| Rhodes RE, Warburton DE, Murray H. (2009) Characteristics of physical activity guidelines and their effect on adherence: a review of randomized trials. Sports Medicine 30(5): 355-375. | Out of scope |
| Rhodes RE, Pfaeffli LA. (2010) Mediators of physical activity behaviour change among adult non-clinical populations: a review update. International Journal of Behavioral Nutrition and Physical Activity 7: 37-48. | Out of scope |
| Rhodes RE, Temple VA, Tuokko HA. (2011) Evidence-based risk assessment and recommendations for physical activity clearance: cognitive and psychological conditions. Applied Physiology Nutrition and Metabolism 36 Suppl1: S113-S153. | Out of scope |
| Rhodes RE, Nasuti G. (2011) Trends and changes in research on the psychology of physical activity across 20 years: A quantitative analysis of 10 journals. Preventive Medicine 53(1-2): 17-23. | Out of scope |
| Rhodes RE, Dickau L. (2013) Moderators of the intention-behaviour relationship in the physical activity domain: a systematic review. British Journal of Sports Medicine 47(4): 215-225. | Out of scope |
| Rhodes SD, Foley KL, Zometa CS et al. (2007) Lay health advisor interventions among Hispanics/Latinos: a qualitative systematic review. American Journal of Preventive Medicine 33(5): 418-427. | Out of scope |
| Richards J, Hillsdon M, Thorogood M et al. (2013) Face-to-face interventions for promoting physical activity. Cochrane Database of Systematic Reviews 30(9): CD010392. | SR but lay health - delivery |
| Riper H, Spek V, Boon B et al. (2011) Effectiveness of E-self-help interventions for curbing adult problem drinking: a meta-analysis. Journal of Medical Internet Research 13(2): e42. | Out of scope |
| Roberts A, Noyes J. (2009). Contraception and women over 40 years of age: Mixed-method systematic review. Journal of Advanced Nursing 65(6): 1155-1170. | Out of scope |
| Rodgers WM, Conner M, Murray TC. (2008) Distinguishing among perceived control, perceived difficulty, and self-efficacy as determinants of intentions and behaviours. British Journal of Social Psychology 47(Pt 4): 607-630. | Out of scope |
| Romelsjö A, Allebeck P, Andréasson S et al. (2012) Alcohol, mortality and cardiovascular events in a 35 year follow-up of a nationwide representative cohort of 50,000 Swedish conscripts up to age 55. Alcohol and Alcoholism 47(3): 322-327. | Not SR |
| Rubinstein A, Colantonio L, Bardach A et al. (2010) Estimation of the burden of cardiovascular disease attributable to modifiable risk factors and cost-effectiveness analysis of preventative interventions to reduce this burden in Argentina. BMC Public Health 20;10:627. | Out of scope |
| Sabia S, Guéguen A, Berr C et al. (2011) High alcohol consumption in middle-aged adults is associated with poorer cognitive performance only in the low socio-economic group. Results from the GAZEL cohort study. Addiction 106(1): 93-101 | Out of scope |
| Sacerdote C, Ricceri F, Rolandsson O et al. (2012) Lower educational level is a predictor of incident type 2 diabetes in European countries: The EPIC-interact study. International Journal of Epidemiology 41(4): 1162-1173. | Out of scope |
| Sahyoun NR, Pratt CA, Anderson A. (2004) Evaluation of nutrition education interventions for older adults: a proposed framework. Journal of the American Dietetic Association 104(1): 58-69. | Out of scope |
| Sanchez-Villegas A, Martínez JA et al. (2003) A systematic review of socioeconomic differences in food habits in Europe: Consumption of cheese and milk. European Journal of Clinical Nutrition 57(8): 917-929. | Out of scope |
| Sandercock GR, Bromley PD, Brodie DA. (2005) Effects of exercise on heart rate variability: inferences from meta-analysis. Medicine & Science in Sports & Exercise 37(3): 433-439. | Out of scope |
| Saraiya M, Glanz K, Briss PA et al. (2004) Interventions to prevent skin cancer by reducing exposure to ultraviolet radiation: a systematic review American Journal of Preventive Medicine 27(5): 422-466. | Out of scope |
| Sargent GM, Forrest LE, Parker RM. (2012) Nurse delivered lifestyle interventions in primary health care to treat chronic disease risk factors associated with obesity: a systematic review. Obesity Reviews 13(12): 1148-1171. | Out of scope |
| Sawaya AL, Sesso R, Florêncio TM et al. (2005) Association between chronic undernutrition and hypertension. Maternal and Child Nutrition 1(3): 155-163. | Out of scope |
| Scharf D, Shiffman S. (2004) Are there gender differences in smoking cessation, with and without bupropion? Pooled- and meta-analyses of clinical trials of Bupropion SR. Addiction 99(11): 1462-1469. | Out of scope |
| Schmid M, Egli K, Martin BW et al. (2009) Health promotion in primary care: evaluation of a systematic procedure and stage specific information for physical activity counselling. Swiss Medical Weekly 14:139(45-46): 665-671. | Out of scope |
| Schutgens CA, Schuring M, Voorham TA et al. (2009) Changes in physical health among participants in a multidisciplinary health programme for long-term unemployed persons. BMC Public Health 19;9:197. | Out of scope |
| Schwartz A, Hazen G, Leifer A et al. (2008). Life goals and health decisions: What will people live (or die) for? Medical Decision Making 28(2): 209-219. | Out of scope |
| Scott EJ, Dimairo M, Hind D et al. (2011) "Booster" interventions to sustain increases in physical activity in middle-aged adults in deprived urban neighbourhoods: internal pilot and feasibility study. BMC Public Health 23;11:129. | Out of scope |
| Secker-Walker RH, Gnich W et al. (2002) Community interventions for reducing smoking among adults. Cochrane Database of Systematic Reviews (3): CD001745. | Out of scope |
| Seefeldt V, Malina RM, Clark MA. (2002) Factors affecting levels of physical activity in adults. Sports Medicine 32(3): 143-168. | Out of scope |
| Seeman TE. (2000) Health promoting effects of friends and family on health outcomes in older adults. American Journal of Health Promotion 14(6): 362-370. | SR? |
| Shaw R, Fenwick E, Baker G et al. (2011) 'Pedometers cost buttons': the feasibility of implementing a pedometer based walking programme within the community. BMC Public Health 31;11:200. | Out of scope |
| Sheeran P, Harris P, Vaughan J et al. (2013) Gone Exercising: Mental contrasting promotes physical activity among overweight, middle-aged, low-SES fishermen. Health Psychology 32(7): 802-809. | Out of scope |
| Sherwood NE, Jeffery RW, Pronk NP et al. (2006) Mail and phone interventions for weight loss in a managed-care setting: weigh-to-be 2-year outcomes. International Journal of Obesity 30(10): 1565-1573. | Out of scope |
| Sherzai A, Heim LT, Boothby C et al. (2012) Stroke, food groups, and dietary patterns: a systematic review. Nutrition Reviews 70(8): 423-435. | Out of scope |
| Siervo M, Arnold R, Wells JC et al. (2011) Intentional weight loss in overweight and obese individuals and cognitive function: a systematic review and meta-analysis. Obesity Reviews 12(11): 968-983 | Out of scope |
| Singh, MAF. (2002) Benefits of exercise and dietary measures to optimize shifts in body composition with age. Asia Pacific Journal of Clinical Nutrition 11: S642-S652. | Out of scope |
| Skelton DA, Howe TE, Ballinger C et al. (2013) Environmental and behavioural interventions for reducing physical activity limitation in community-dwelling visually impaired older people. Cochrane Database of Systematic Reviews 5(6): CD009233. | Out of scope |
| Sloan F, Platt A. (2011) Information, risk perceptions, and smoking choices of youth. Journal of Risk and Uncertainty 42(2): 161-193. | Out of scope |
| Smedslund G, Fisher KJ, Boles SM et al. (2004) The effectiveness of workplace smoking cessation programmes: a meta-analysis of recent studies. Tobacco Control 13(2): 197-204. | Out of scope |
| Smeeth L, Iliffe S. (2006) Community screening for visual impairment in the elderly. Cochrane Database of Systematic Reviews 3: CD001054. | Out of scope |
| Smerecnik C, Grispen JE, Quaak M. (2012) Effectiveness of testing for genetic susceptibility to smoking-related diseases on smoking cessation outcomes: a systematic review and meta-analysis. Tobacco Control 21(3): 347-354. | Out of scope |
| Sodergren M. (2013) Lifestyle predictors of healthy ageing in men. Maturitas 75(2): 113-117. | Out of scope |
| Solomon LJ, Marcy TW, Howe KD et al. (2005) Does extended proactive telephone support increase smoking cessation among low-income women using nicotine patches? Preventive Medicine 40(3): 306-313. | Out of scope |
| Somerset SM, Markwell K, Al-Foraih M. (2013) A systematic review of baseline psychosocial characterisation in dietary randomised controlled trials for weight loss. European Journal of Clinical Nutrition 67(7): 697-702. | Out of scope |
| Spring B, Howe D, Berendsen M et al. (2009) Behavioral intervention to promote smoking cessation and prevent weight gain: a systematic review and meta-analysis. Addiction 104(9): 1472-1486. | Out of scope |
| Spring B, Moller AC, Coons MJ. (2012) Multiple health behaviours: overview and implications. Journal of Public Health 34: I3-I10. | Out of scope |
| Stalsberg R, Pedersen AV. (2010) Effects of socioeconomic status on the physical activity in adolescents: a systematic review of the evidence. Scandinavian Journal of Medicine & Science in Sports 20(3): 368-383. | Out of scope |
| Steffen PR, Smith TB, Larson M et al. (2006) Acculturation to Western society as a risk factor for high blood pressure: a meta-analytic review. Psychosomatic Medicine 68(3): 386-397. | Young people |
| Stern C, Konno R. (2009) Physical leisure activities and their role in preventing dementia: a systematic review. International Journal of Evidence-Based Healthcare 7(4): 270-282. | Out of scope |
| Strand BH, Langballe EM, Hjellvik V et al. (2013) Midlife vascular risk factors and their association with dementia deaths: Results from a Norwegian prospective study followed up for 35 years. Journal of the Neurological Sciences 324(1-2): 124-130. | Out of scope |
| Stroth S, Hille K, Spitzer M et al. (2009) Aerobic endurance exercise benefits memory and affect in young adults. Neuropsychological Rehabilitation 19(2): 223-243. | Out of scope |
| Suls J, Bunde J. (2005) Anger, anxiety, and depression as risk factors for cardiovascular disease: The problems and implications of overlapping affective dispositions. Psychological Bulletin 131(2): 260-300. | Out of scope |
| Summerbell CD, Cameron C, Glasziou PP. (2008) Advice on low-fat diets for obesity. Cochrane Database of Systematic Reviews 3: CD003640. | Out of scope |
| Sutherland K, Christianson JB, Leatherman S. (2008) Impact of targeted financial incentives on personal health behaviour: A review of the literature. Medical Care Research and Review 65(6): 36S-78S. | Out of scope |
| Szaflarski M, Cubbins LA. (2004) Self-reported health in Poland and the United States: a comparative analysis of demographic, family and socioeconomic influences. Health: An Interdisciplinary Journal for the Social Study of Health, Illness & Medicine 8(1): 5-31. | Quality of SR? |
| Taggart J, Williams A, Dennis S et al. (2012) A systematic review of interventions in primary care to improve health literacy for chronic disease behavioral risk factors. BMC Family Practice 1:13:49. | Out of scope |
| Tamayo T, Christian H, Rathmann W. (2010) Impact of early psychosocial factors (childhood socioeconomic factors and adversities) on future risk of type 2 diabetes, metabolic disturbances and obesity: a systematic review. BMC Public Health 1;10:525. | Review 3 |
| Taylor CA, Shaw RL, Dale J et al. (2011) Enhancing delivery of health behaviour change interventions in primary care: A meta-synthesis of views and experiences of primary care nurses. Patient Education and Counseling 85(2): 315-322. | Out of scope |
| Taylor J, Cottrell C, Chatterton H et al. (2013) Identifying risk and preventing progression to Type 2 diabetes in vulnerable and disadvantaged adults: a pragmatic review. Diabetic Medicine 30(1): 16-25. | SR about delivery |
| Taylor MJ, McCormick D, Shawis T et al. (2011) Activity-promoting gaming systems in exercise and rehabilitation. Journal of Rehabilitation Research & Development 48(10): 1171-1186. | Loneliness, interventions rev 3 |
| Thomas S, Fayter D, Misso K et al. (2008) Population tobacco control interventions and their effects on social inequalities in smoking: systematic review. Tobacco Control 17(4): 230-237. | Out of scope |
| Thompson B, Coronado G, Snipes SA et al. (2003) Methodologic advances and ongoing challenges in designing community-based health promotion programs. Annual Review of Public Health 24: 315-340. | Policy based |
| Thompson RL, Summerbell CD, Hooper L et al. (2003) Dietary advice given by a dietitian versus other health professional or self-help resources to reduce blood cholesterol. Cochrane Database of Systematic Reviews (3): CD001366. | Out of scope |
| Thompson VJ, Baranowski T, Cullen KW et al. (2003) Influences on diet and physical activity among middle-class African American 8-to 10-year-old girls at risk of becoming obese. Journal of Nutrition Education and Behavior 35(3): 115-123. | Out of scope |
| Thompson P, Lang L, Annells M. (2008) A systematic review of the effectiveness of in-home community nurse led interventions for the mental health of older persons. Journal of Clinical Nursing 17(11): 1419-1427. | Children |
| Thompson Coon J, Boddy K, Stein K et al. (2011) Does participating in physical activity in outdoor natural environments have a greater effect on physical and mental wellbeing than physical activity indoors? A systematic review. Environmental Science & Technology 45(5): 1761-1772. | J Clin Nurs Check ref |
| Thomson CA, Ravia J. (2011) A systematic review of behavioral interventions to promote intake of fruit and vegetables. Journal of the American Dietetic Association 111(10): 1523-1535. | Out of scope |
| Thorndike AN, Biener L, Rigotti NA. (2002) Effect on smoking cessation of switching nicotine replacement therapy to over-the-counter status. American Journal of Public Health 92(3): 437-442. | SR? Behavioural ints not HB |
| Thorpe L, Davidson P, Janicki M. (2001) Healthy ageing - Adults with intellectual disabilities: Biobehavioural issues. Journal of Applied Research in Intellectual Disabilities 14(3): 218-228. | Out of scope |
| Titze S, Stronegger W, Owen N. (2005) Prospective study of individual, social, and environmental predictors of physical activity: women's leisure running. Psychology of Sport and Exercise 6(3): 363-376. | Out of scope |
| Tobacco use: NIH State-of-the-Science Conference Statement on Tobacco Use: Prevention, Cessation, and Control. Annals of Internal Medicine 145: 839-844. | Out of scope |
| Tsai SF, Cheney D. (2012) The Impact of the Adult-Child Relationship on School Adjustment for Children at Risk of Serious Behavior Problems. Journal of Emotional and Behavioral Disorders 20(2): 105-114. | Cross-sectional? |
| Tzelepis F, Paul CL, Duncan SL et al. (2012) Increasing the reach of quitlines through active telephone recruitment: Do cold-called smokers differ from quitline callers? Nicotine & Tobacco Research 14(12): 1488-1493. | Out of scope |
| Ussher MH, Taylor AH, West R et al. (2000) Does exercise aid smoking cessation? A systematic review. Addiction 95(2): 199-208. | Out of scope |
| Van Cauwenberg J, De Bourdeaudhuij I, De Meester F et al. (2011) Relationship between the physical environment and physical activity in older adults: A systematic review. Health & Place 17(2): 458-469. | Out of scope |
| van Genugten L, van Empelen P, Flink I et al. (2010) Systematic development of a self-regulation weight-management intervention for overweight adults. BMC Public Health 27;10:649. | Out of scope |
| Vanwormer JJ, Boucher JL, Pronk NP. (2006) Telephone-based counseling improves dietary fat, fruit, and vegetable consumption: a best-evidence synthesis. Journal of the American Dietetic Association 106(9): 1434-1444. | Out of scope |
| Vasilaki EI, Hosier SG, Cox WM. (2006) The efficacy of motivational interviewing as a brief intervention for excessive drinking: a meta-analytic review. Alcohol & Alcoholism 41(3): 328-335. | Out of scope |
| Volkert D. (2005) Nutrition and lifestyle of the elderly in Europe. Journal of Public Health 13(2): 56-61. | Out of scope |
| Walsh PN, Heller T, Schupf N et al. (2001) Healthy ageing - Adults with intellectual disabilities: Women's health and related issues. Journal of Applied Research in Intellectual Disabilities 14(3): 195-217. | Out of scope |
| Walters ST, Wright JA, Shegog R. (2006) A review of computer and Internet-based interventions for smoking behaviour. Addictive Behaviors 264-277. | Out of scope |
| Wang D, Connock M, Barton P et al. (2008) 'Cut down to quit' with nicotine replacement therapies in smoking cessation: a systematic review of effectiveness and economic analysis. Health Technology Assessment (Winchester, England) 12(2): iii-iv, ix-xi, 1-135. | Out of scope |
| Wang HHX. (2011) Effectiveness of lifestyle interventions in reducing cardiovascular risk factors among Chinese subjects in primary care setting: A systematic review. International Journal of Cardiology 147: S32. | Out of scope |
| Warburton D, Charlesworth S, Ivey A et al. (2010) A systematic review of the evidence for Canada's Physical Activity Guidelines for Adults. International Journal of Behavioral Nutrition and Physical Activity 7(1); 39. | Risks Rev 2 |
| Waugh EJ, Lam MA, Hawker GA et al. (2009) Risk factors for low bone mass in healthy 40-60 year old women: A systematic review of the literature."Osteoporosis International 20(1): 1-21. | Out of scope |
| Weinstein PK. (2006) A review of weight loss programs delivered via the Internet. Journal of Cardiovascular Nursing 21(4):251-8. | Out of scope |
| Westerhof GJ, Dittmann-Kohli F, Thissen T. (2001) Beyond life satisfaction: Lay conceptions of well-being among middle-aged and elderly adults. Social Indicators Research 56(2): 179-203. | Out of scope |
| White IR, Altmann DR, Nanchahal K. (2002) Alcohol consumption and mortality: modelling risks for men and women at different ages. BMJ 325;7357:191. | Out of scope |
| White A, Kavanagh D, Stallman H et al. (2010) Online alcohol interventions: a systematic review. Journal of Medical Internet Research 12(5): e62. | Out of scope |
| Whitlock EP, Williams SB. (2003) The primary prevention of heart disease in women through health behavior change promotion in primary care. Women's Health Issues 13(4): 122-141. | Rev 3? |
| Whitlock EP, Polen MR, Green CA et al. (2004) Behavioral Counseling Interventions in Primary Care to Reduce Risky/ Harmful Alcohol Use by Adults: A Summary of the Evidence for the U.S. Preventive Services Task Force." Annals of Internal Medicine 140(7): 557-568+I564. | Out of scope |
| Whittaker R, McRobbie H, Bullen C et al. (2010) Mobile phone-based interventions for smoking cessation. Sao Paulo Medical Journal 128(2): 106-107. | Out of scope |
| Whitt-Glover MC, Kumanyika SK. (2009) Systematic review of interventions to increase physical activity and physical fitness in African-Americans. American Journal of Health Promotion 23(6): S33-56. | Out of scope |
| Wieland LS, Falzon L, Sciamanna CN et al. (2012) Interactive computer-based interventions for weight loss or weight maintenance in overweight or obese people. Cochrane Database of Systematic Reviews 8: CD007675. | Out of scope |
| Wielgosz AT, Nolan RP. (2000) Biobehavioral factors in the context of ischemic cardiovascular diseases. Journal of Psychosomatic Research 48(4-5): 339-345. | Out of scope |
| Wikström K, Lindström J, Tuomilehto J et al. (2011) Socio-economic differences in dysglycemia and lifestyle-related risk factors in the Finnish middle-aged population. European Journal of Public Health 21(6): 768-774. | Out of scope |
| Wilcox S, Parra-Medina D, Thompson-Robinson M et al. (2001) Nutrition and physical activity interventions to reduce cardiovascular disease risk in health care settings: a quantitative review with a focus on women. Nutrition Reviews 59(7): 197-214. | Only abstract - not sure. No full paper |
| Wilcox S. (2002) Physical activity in older women of color. Topics in Geriatric Rehabilitation 18(1): 21-33. | Out of scope |
| Willeit P, Thompson A, Aspelund T et al. (2013) Hemostatic factors and risk of coronary heart disease in general populations: New prospective atudy and updated meta-Analyses." PLoS One 8(2):e55175. | Out of scope |
| Williams PT. (2001) Physical fitness and activity as separate heart disease risk factors: a meta-analysis. Medicine & Science in Sports & Exercise 33(5): 754-761. | Out of scope |
| Williams NH, Hendry M, France B et al. (2007) Effectiveness of exercise-referral schemes to promote physical activity in adults: systematic review. British Journal of General Practice 57(545): 979-986. | Out of scope |
| Williams AD. (2012) Use of a Text Messaging Program to Promote Adherence to Daily Physical Activity Guidelines: A Review of the Literature. Bariatric Nursing and Surgical Patient Care 7(1): 13-16. | Out of scope |
| Wilmot EG, Edwardson CL, Achana FA et al. (2012) Sedentary time in adults and the association with diabetes, cardiovascular disease and death: systematic review and meta-analysis. Diabetologia 55(11): 2895-2905. | Out of scope |
| Wolfe BL, LeMura LM, Cole PJ. (2004) Quantitative analysis of single- vs. multiple-set programs in resistance training. Journal of Strength & Conditioning Research 18(1): 35-47. | Out of scope |
| Wong JY, Gilson ND, van Uffelen JG et al. (2012) The effects of workplace physical activity interventions in men: a systematic review. Database of Abstracts of Reviews of Effects 303-313. | Out of scope |
| Worrall-Carter L, Edward KL, Page K. (2012) Women and cardiovascular disease: at a social disadvantage? Collegian 19(1): 33-37. | Out of scope |
| Wu S, Cohen D, Shi Y, Pearson M et al. (2011) Economic Analysis of Physical Activity Interventions. American Journal of Preventive Medicine 40(2): 149-158. | Out of scope |
| Yamaoka K, Tango T. (2005) Efficacy of lifestyle education to prevent type 2 diabetes: a meta-analysis of randomized controlled trials. Diabetes Care 28(11): 2780-2786. | Out of scope |
| Yeh MC, Ickes SB, Lowenstein LM et al. (2008) Understanding barriers and facilitators of fruit and vegetable consumption among a diverse multi-ethnic population in the USA. Health Promotion International 23(1): 42-51. | Not SR |
| Yen IH, Michael YL, Perdue L. (2009) Neighborhood environment in studies of health of older adults: a systematic review. American Journal of Preventive Medicine 37(5): 455-463. | Age >55, 25 of 33 studies X-sectional |
| Zerger S, Strehlow AJ, Gundlapalli AV. (2008) Homeless young adults and behavioral health - An overview. American Behavioral Scientist 51(6): 824-841. | Out of scope |
| Zhang J, Wang Z. (2008) Factors associated with smoking in Asian American adults: A systematic review. Nicotine & Tobacco Research 10(5): 791-801. | Out of scope |
| Zhou BF. (2002) Effect of body mass index on all-cause mortality and incidence of cardiovascular diseases--report for meta-analysis of prospective studies open optimal cut-off points of body mass index in Chinese adults. Biomedical & Environmental Sciences 15(3): 245-252. | Out of scope |
| Zijlstra GA, van Haastregt JC, van Rossum E et al. (2007) Interventions to reduce fear of falling in community-living older people: a systematic review. Journal of the American Geriatric Society 55(4): 603-15. | Out of scope |

**2. Primary Studies**

| **Study** | **Reason excluded** |
| --- | --- |
| Aarsland D, Sardahaee FS, Anderssen S et al. (2010) Is physical activity a potential preventive factor for vascular dementia? A systematic review. Aging & Mental Health 14(4): 386-395. | SR review 2 |
| Adams J, White M, Pearce MS et al. (2004) Life course measures of socioeconomic position and self-reported health at age 50: prospective cohort study. Journal of Epidemiolgy and Community Health 58(12): 1028-1029. | Not HB |
| Aggarwal A, Monsivais P, Cook AJ et al. (2011) Does diet cost mediate the relation between socioeconomic position and diet quality? European Journal of Clinical Nutrition 65(9): 1059-1066. | But x-sectional |
| Ahmad B. (2008) Life events and change in health behaviours at midlife: an analysis of data from the National Survey of Health and Development. Doctoral dissertation, UCL (University College London). | Mainly medical condition - need full paper? Thesis only |
| Allen KV, Frier BM, Strachan MW. (2004) The relationship between type 2 diabetes and cognitive dysfunction: longitudinal studies and their methodological limitations. European Journal of Pharmacology 490(1): 169-175. | Not HB |
| Almeida OP, Hulse GK, Lawrence D et al. (2002) Smoking as a risk factor for Alzheimer’s disease: contrasting evidence from a systematic review of case–control and cohort studies. Addiction, 97(1): 15-28. | Primary review 2? |
| Anderson R, Anderson D, Hurst C. (2010) Modeling factors that influence exercise and dietary change among midlife Australian women: results from the Healthy Aging of Women Study. Maturitas 67(2): 151-158. | Not HB |
| Anderson FE. (2010) Being 50: A psycho-social study of a cohort of women in contemporary society from a life course perspective. Thesis. | Thesis, not HB |
| Andresen EM, Wolinsky FD, Miller JP et al. (2006) Cross-sectional and longitudinal risk factors for falls, fear of falling, and falls efficacy in a cohort of middle-aged African Americans. Gerontologist 46(2): 249-257. | SR, rev 3 |
| Angevaren M, Aufdemkampe G, Verhaar HJ et al. (2008) Physical activity and enhanced fitness to improve cognitive function in older people without known cognitive impairment. The Cochrane Database of Systematic Reviews 3(3). | Rev 2/3? |
| Anon. (2001) Eating for health? A survey of attitudes, awareness and eating habits among adults in Northern Ireland. Health Promotion Agency | Not a primary study, no methodology |
| Anon. (2003) Improving the health and wellbeing of people in mid-life and beyond: making the case for local authorities. NHS Health Development Agency. | Not a primary study, no methodology |
| Anon. (2004) Taking action: improving the health and wellbeing of people in mid-life and beyond. NHA Health Development Agency. | Not a primary study, no methodology |
| Anon. (2011) Small area indicators for joint strategic needs assessment: Developed by PHOs on behalf of the Department of Health. EMPHO. | Not relevant |
| Anstey KJ, von Sanden C, Salim A et al. (2007) Smoking as a risk factor for dementia and cognitive decline: A meta-analysis of prospective studies. American Journal of Epidemiology 166(4): 367-78. | SR, rev 2 |
| Anstey KJ. (2008) Alcohol exposure and cognitive development: An example of why we need a contextualized, dynamic life course approach to cognitive ageing - a mini-review. Gerontology 54(5): 283-291. | SR, rev 2 |
| Anstey KJ, Cherbuin N, Budge M et al. (2011) Body mass index in midlife and late-life as a risk factor for dementia: a meta-analysis of prospective studies. Obesity Reviews 12: e426-e437. | SR, rev 2 |
| Anthony D, Baggott R, Tanner J et al. (2012) Health, lifestyle, belief and knowledge differences between two ethnic groups with specific reference to tobacco, diet and physical activity. Journal of Advanced Nursing 68(11): 2496-2503. | Not mid-life, X-sectional primary study |
| Araújo Filho A, Salomão SR, Berezovsky A et al. (2008) Prevalence of visual impairment, blindness, ocular disorders and cataract surgery outcomes in low-income elderly from a metropolitan region of Sao Paulo--Brazil. Arquivos Brasileiros de Oftalmologia 71(2): 246-253. | Not HB |
| Areosa SA, Grimley EV. (2002) Effect of the treatment of Type II diabetes mellitus on the development of cognitive impairment and dementia. The Cochrane Library (4): CD003804. | Existing diabetes |
| Arpanantikul M. (2006) Self-care process as experienced by middle-aged Thai women. Health Care for Women International 27(10): 893-907. | Not about HB, Thailand, v small n=15 |
| Arvanitakis Z, Wilson RS, Bienias JL et al. (2006) Diabetes mellitus and risk of Alzheimer disease and decline in cognitive function. Archives of Neurology 61(5): 661-6. | Not midlife, rev 2? |
| Ashcroft RE, Marteau TM, Oliver A. (2008) Incentive mechanisms require deeper understanding. BMJ 337;7665:311. | Not a primary, financial incentives review, not mid-life |
| Ashcroft RE, Marteau TM, Oliver A. (2008) Payment to look after health: Incentive mechanisms require deeper understanding. BMJ 6;337:a1135. | Not full paper |
| Ashford S, Edmunds J, French DP. (2010) What is the best way to change self-efficacy to promote lifestyle and recreational physical activity? A systematic review with meta-analysis. British Journal of Health Psychology 15(Pt 2): 265-288. | SR, rev 3 |
| August KJ, Sorkin DH. (2011) Racial/ethnic disparities in exercise and dietary behaviors of middle-aged and older adults. Journal of General Internal Medicine 26(3): 245-250. | X-sectional |
| Avis NE, Ory M, Matthews KA et al. (2003) Health-related quality of life in a multiethnic sample of middle-aged women: Study of Women's Health Across the Nation (SWAN). Medical Care 41(11): 1262-1276. | Not HB (HRQoL) |
| Ayotte BJ, Margrett JA, Hicks-Patrick J. (2010) Physical activity in middle-aged and young-old adults: the roles of self-efficacy, barriers, outcome expectancies, self-regulatory behaviors and social support. Journal of Health Psychology 15(2): 173-185. | X-sectional |
| Ayotte BJ, Margrett JA, Patrick JH. (2013) Dyadic analysis of self-efficacy and perceived support: the relationship of individual and spousal characteristics with physical activity among middle-aged and young-older adults. Psychology & Aging 28(2): 555-563. | X-sectional |
| Baker RS, Bazargan M, Bazargan-Hejazi S et al. (2005) Access to vision care in an urban low-income multiethnic population. Ophthalmic Epidemiology 12(1): 1-12. | X-sectional |
| Barg CJ, Latimer AE, Pomery EA et al. (2012) Examining predictors of physical activity among inactive middle-aged women: an application of the health action process approach. Psychology & Health 27(7): 829-845. | Rev 2? |
| Beenackers MA, Kamphuis CB, Giskes K et al. (2012) Socioeconomic inequalities in occupational, leisure-time, and transport related physical activity among European adults: A systematic review. The International Journal of Behavioral Nutrition and Physical Activity 19;9:116. | Not primary, already IN as SR |
| Beeri MS, Rapp M, Silverman JM et al. (2006) Coronary artery disease is associated with Alzheimer disease neuropathology in APOE4 carriers. Neurology 66(9): 1399-1404. | Not HB |
| Benjamins MR. (2006) Religious influences on preventive health care use in a nationally representative sample of middle-age women. Journal of Behavioral Medicine 29(1): 1-16 | X-sect |
| Benyamini Y, Blumstein T, Boyko V et al. (2008) Cultural and educational disparities in the use of primary and preventive health care services among midlife women in Israel. Womens Health Issues 18(4): 257-266. | X-sect |
| Bertrais S, Preziosi P, Mennen L et al. (2004) Sociodemographic and geographic correlates of meeting current recommendations for physical activity in middle-aged French adults: the Supplementation en Vitamines et Mineraux Antioxydants (SUVIMAX) Study. American Journal of Public Health 94(9): 1560-1566. | X-sect |
| Betschild MJ. (1998) Midlife women’s lived experience: their patterns of health, leisure and enjoyment. Thesis. | Thesis |
| Beydoun MA, Beydoun HA, Wang Y. (2008) Obesity and central obesity as risk factors for incident dementia and its subtypes: a systematic review and meta-analysis. Obesity Reviews 9: 204-218. | Not HB |
| Beydoun MA, Kuczmarski MTF, Mason MA et al. (2009) Role of depressive symptoms in explaining socioeconomic status disparities in dietary quality and central adiposity among US adults: a structural equation modeling approach. American Journal of Clinical Nutrition 90(4): 1084-95. | Not sure -depression as mediator of link |
| Biessels GJ, Staekenborg S, Brunner E et al. (2006) Risk of dementia in diabetes mellitus: a systematic review. Lancet Neurology 5(1): 64-74. | Not HB |
| Bishop AJ, Marteau TM, Hall S et al. (2005) Increasing women’s intentions to stop smoking following an abnormal cervical smear test result. Preventive Medicine 41(1), 179-185. | X-sect |
| Bjørk C, Thygesen LC, Vinther-Larsen M et al. (2008) Time trends in heavy drinking among middle-aged and older adults in Denmark. Alcoholism: Clinical & Experimental Research 32(1): 120-127. | More a prevalence study |
| Blank TO. (2007) Review of Midlife and older LGBT adults: Knowledge and affirmative practice for the social services. Educational Gerontology 33(11): 1016-1017. | Book review |
| Bode C, De Ridder DTD. (2007) Investing in the future - identifying participants in an educational program for middle-aged and older adults. Health Education Research 22(4): 473-482. | Mean age 61 |
| Boone-Heinonen J, Gordon-Larsen P, Kiefe CI et al. (2011) Fast food restaurants and food stores: longitudinal associations with diet in young to middle-aged adults: the CARDIA study. Archives of Internal Medicine 171(13): 1162-1170. | Mean age at follow -up 39, not midlife |
| Borrell LN, Kiefe CI, Diez-Roux AV et al. (2013) Racial discrimination, racial/ethnic segregation, and health behaviors in the CARDIA study. Ethnicity & Health 18(3): 227-243. | Mean age at follow up 39 not midlife |
| Bosworth HB, Bastian LA, Kuchibhatla MN et al. (2001) Depressive symptoms, menopausal status, and climacteric symptoms in women at midlife. Psychosomatic Medicine 63(4): 603-608. | Not HB |
| Boyce T, Robertson R, Dixon A. (1999) Commissioning and behaviour change: kicking bad habits final report. Cambridge: Kings Fund. | Not primary study but useful background |
| Britton A, Shipley M, Singh-Manoux A et al. (2008). Successful aging: the contribution of early-life and midlife risk factors. Journal of the American Geriatrics Society 56(6): 1098-1105. | Review 2 |
| Brummett BH, Siegler IC, Day RS et al. (2008) Personality as a predictor of dietary quality in spouses during midlife. Behavioral Medicine 34(1): 5-10. | X-sectional, personality |
| Bull FC. (2010) Physical Activity Guidelines in the UK: review and recommendations. BHF National Centre Physical Activity + Health. | Not barriers and facilitators - |
| Burazeri G, Kark JD. (2010. Prevalence and determinants of binge drinking in middle age in a transitional post-communist country: a population-based study in Tirana, Albania. Alcohol & Alcoholism 45(2): 180-187. | X-sect |
| Burton NW, Khan A, Brown WJ et al. (2012) The association between sedentary leisure and physical activity in middle-aged adults. British Journal of Sports Medicine 46(10): 747-752. | X-sectional |
| Cameron LD, Marteau TM, Brown PM et al. (2011) Communication strategies for enhancing understanding of the behavioural implications of genetic and biomarker tests for disease risk: The role of coherence. Journal of Behavioral Medicine 35(3): 286-298. | Not HB |
| Caspi CE, Sorensen G, Subramanian SV et al. (2012) The local food environment and diet: A systematic review. Health & Place 18(5): 1172–1187. | Relevant but includes a lot of child studies and not poss to separate data for midlife. |
| Cattan M, White M, Bond J et al. (2005) Preventing social isolation and loneliness among older people: a systematic review of health promotion interventions. Ageing & Society 25: 41-67. | SR, rev 3? |
| Cavill N, Roberts R. (2011) Data Sources: environmental influences on physical activity and diet. National Obesity Observatory | Not primary study, no methodology |
| Cerin E, Leslie E. (2008) How socio-economic status contributes to participation in leisure-time physical activity. Social Science & Medicine 66(12): 2596-2609. | X-sectional |
| Chambers JL. (2000) Body image and physical activity in midlife women. Thesis. | Thesis - but look for full paper |
| Chao S, Roberts JS, Marteau TM et al. (2008) Health behaviour changes after genetic risk assessment for Alzheimer Disease: the REVEAL study. Alzheimer Disease and Associated Disorders 22(1): 94-7. | Review 3 |
| Charreire H, Kesse-Guyot E, Bertrais S et al. (2011) Associations between dietary patterns, physical activity (leisure-time and occupational) and television viewing in middle-aged French adults. British Journal of Nutrition 105(6): 902-910. | Cross-sectional |
| Choi YH. (2005) The factors influencing the compliance of breast self-examination of middle-aged women. Daehan Ganho Haghoeji 35(4): 721-727. | Cross-sectional |
| Choi EJ, Jekal Y, Kim S et al. (2010) Middle-aged women's awareness of cholesterol as a risk factor: results from a national survey of Korean Middle-aged Women's Health Awareness (KomWHA) study. International Journal of Nursing Studies 47(4): 452-460. | Out of scope – review 2? |
| Choi J, Guiterrez Y, Gilliss C et al. (2011) Body mass index in multiethnic midlife women: Influence of demographic characteristics and physical activity. Health Care for Women International 32(12): 1079-1087. | Not HB - prevalence |
| Chou KL, Liang K, Mackenzie CS. (2011) Binge drinking and Axis I psychiatric disorders in community-dwelling middle-aged and older adults: results from the National Epidemiologic Survey on Alcohol and Related Conditions (NESARC). Journal of Clinical Psychiatry 72(5): 640-647. | X-sect |
| Claassen L, Henneman L, Nijpels G et al. (2007) Causal beliefs and perceptions of risk for diabetes and cardiovascular disease, the Netherlands, 2007. Preventing Chronic Disease 8(6): A130. | X-sectional in people at risk |
| Claassen L, Henneman L, van der Weijden T et al. (2010) Being at risk for cardiovascular disease: perception and preventive behavior in people with and without a known genetic predisposition. Psychology, Health & Medicine 17(5): 511-521. | X-sectional in people at risk |
| Claassen L, Henneman L, De Vet R et al. (2010) Fatalistic responses to different types of genetic risk information: exploring the role of self-malleability. Psychology & Health 25(2): 183-196. | X-sectional in people at risk |
| Claassen L, Henneman L, Kindt I et al. (2012) Perceived risk and presentations of cardiovascular disease and preventive behaviour in people diagnosed with hypercholesterolemia: a cross-sectional questionnaire study. Journal of Health Psychology 15(1): 33-43. | X-sect |
| Cleland VJ, Ball K, Magnussen C et al. (2009) Socioeconomic position and the tracking of physical activity and cardiorespiratory fitness from childhood to adulthood. American Journal of Epidemiology 1;170(9): 1069-77. | Not midlife |
| Colby SE, Johnson AL, Eickhoff A et al. (2009) Promoting community health resources: preferred communication strategies. Health Promotion Practice 12(2): 271-9. | Not midlife |
| Collins RE, Wright AJ, Marteau TM. (2011) Impact of communicating personalized genetic risk information on perceived control over the risk: A systematic review. Genetics in Medicine 13(4):273-7 | Not HB |
| Conklin AI, Maguire ER, Monsivais P. (2013) Economic determinants of diet in older adults: systematic review. Journal of Epidemiology and Community Health 67(9): 721-727. | Older >60 |
| Cooper H, Ginn J, Arber S. (1999) Health-related behaviour and attitudes of older people: a secondary analysis of national datasets. Health Education Authority. | >55 y |
| Cooper R, Mishra G, Clennell S et al. (2008) Menopausal status and physical performance in midlife: findings from a British birth cohort study. Menopause 15(6): 1079-1085. | Not HB |
| Cooper R, Hyppönen E, Berry D et al. (2010) Associations between parental and offspring adiposity up to midlife: the contribution of adult lifestyle factors in the 1958 British Birth Cohort Study. American Journal of Clinical Nutrition 92(4): 946-953. | Not HB |
| Cooper R, Mishra GD, Kuh D. (2011) Physical activity across adulthood and physical performance in midlife: findings from a British birth cohort. American Journal of Preventive Medicine 41(4): 376-384. | Not HB |
| Crandall CJ, Merkin SS, Seeman TE et al. (2012) Socioeconomic status over the life-course and adult bone mineral density: The Midlife in the U.S. Study. Bone 51(1): 107-113. | Not review 1 – rev2 ? |
| Crane PB, Wallace DC. (2007) Cardiovascular risks and physical activity in middle-aged and elderly African American women. Journal of Cardiovascular Nursing 22(4): 297-303. | Not HB |
| Crockett RA, Weinman J, Hankins M et al. (2009) Time orientation and health-related behaviour: Measurement in general population samples.” Psychology and Health 24(3): 333-50. | Unclear how this related to modifiable HB |
| Cullati S, Charvet-Bérard AI, Perneger TV. (2009) Cancer screening in a middle-aged general population: factors associated with practices and attitudes. BMC Public Health 29;9:118. | About screening which is excluded |
| Dainese SM, Allemand M, Ribeiro N et al. (2011) Protective factors in midlife: How do people stay healthy? GeroPsych: The Journal of Gerontopsychology and Geriatric Psychiatry 24(1): 19-29. | Consider for review 2 |
| Damiani G, Federico B, Bianchi CB et al. (2011) Socio-economic status and prevention of cardiovascular disease in Italy: evidence from a national health survey. European Journal of Public Health 21(5): 591-596. | But X-sectional |
| Darling CA, Coccia C, Senatore N. (2012) Women in midlife: stress, health and life satisfaction. Stress & Health 28(1): 31-40. | Review not SR |
| Dawson J, Hillsdon M, Boller I et al. (2007) Perceived barriers to walking in the neighborhood environment: a survey of middle-aged and older adults. Journal of Aging & Physical Activity 15(3): 318-335. | But mainly older people |
| De Mendonça SN, Brandão HC, Brandão WA et al. (2013) Food preferences of middle aged and elderly subjects in a Brazilian city. Journal of Nutrition, Health & Aging 17(2): 130-135. | Cross-sectional study, Brazil |
| Diepeveen S, Ling T, Suhrcke M et al. (2013) Public acceptability of government intervention to change health-related behaviours: A systematic review and narrative synthesis. BMC Public Health 15;13:756. | Barriers and facilitators to policy interventions |
| Dormandy E, Marteau T. (2005) The need to facilitate informed choice equitably. Psychology & Health 20: 69-70. | Abstract only |
| Duncan MJ, Vandelanotte C, Rosenkranz RR et al. (2012) Effectiveness of a website and mobile phone based physical activity and nutrition intervention for middle-aged males: Trial protocol and baseline findings of the ManUp Study. BMC Public Health 5;12:656. | Intervention - more relevant for review 3 |
| Ecob R, Sutton G, Rudnicka A et al. (2008) Is the relation of social class to change in hearing threshold levels from childhood to middle age explained by noise, smoking, and drinking behaviour? International Journal of Audiology 47(3): 100-108. | X-sectional |
| Ekkekakis P, Lind E, Vazou S. (2010) Affective responses to increasing levels of exercise intensity in normal-weight, overweight, and obese middle-aged women. Obesity 18(1): 79-85. | Responses not B and F |
| El-Sayed AM, Scarborough P, Galea S. (2012) Unevenly distributed: a systematic review of the health literature about socioeconomic inequalities in adult obesity in the United Kingdom. BMC Public Health 9;12:18. | Risk of obesity not HB |
| Elavsky S, Gold CH. (2009) Depressed mood but not fatigue mediate the relationship between physical activity and perceived stress in middle-aged women. Maturitas 64(4): 235-240. | Not B and F to HB |
| Elavsky S. (2010) Longitudinal examination of the exercise and self-esteem model in middle-aged women. Journal of Sport & Exercise Psychology 32(6): 862-880. | No info on effects on PA outcomes |
| Elgan C, Fridlund B. (2011) Middle-aged women and everyday life: implications for health. British Journal of Nursing 20(9): 570-575. | Not HB |
| Elsabagh S, Hartley D, Randall D et al. (2004) Mood changes after cognitive testing in late middle-age: impacts of sex and habitual alcohol consumption. Pharmacology, Biochemistry & Behavior 78(3): 621-628. | Not HB |
| Elstad JI. (2005) Childhood adversities and health variations among middle-aged men: a retrospective lifecourse study. European Journal of Public Health 15(1): 51-58. | More about risk factors than HB |
| Emberson JR, Whincup PH, Morris RW et al. (2004) Social class differences in coronary heart disease in middle-aged British men: implications for prevention. International Journal of Epidemiology 33(2): 289–296. | Effect on risk factors not PA |
| Emslie C, Hunt K, Lyons A. (2012) Older and wiser? Men's and women's accounts of drinking in early mid-life. Sociology of Health & Illness 34(4): 481-496. | X-sectional |
| Emslie C, Hunt K, Lyons A. (2013) The role of alcohol in forging and maintaining friendships amongst Scottish men in midlife. Health Psychology 32(1): 33-41. | X-sectional |
| Enjezab B, Farajzadegan Z, Taleghani F et al. (2012) Health promoting behaviors in a population-based sample of middle-aged women and its relevant factors in Yazd, Iran. International Journal of Preventive Medicine 3(Suppl 1): S191-198. | X-sectional |
| Erens B, Primatesta P, Prior G (Eds). (2001) Health Survey for England: The health of minority ethnic groups' 99: a survey carried out on behalf of the Department of Health. Stationery Office | 1999 not primary study no methodology |
| Estaquio C, Druesne-Pecollo N, Latino-Martel P et al. (2008) Socioeconomic differences in fruit and vegetable consumption among middle-aged French adults: adherence to the 5 A Day recommendation. Journal of the American Dietetic Association 108(12): 2021-2030. | X-sect |
| Evandrou M, Glaser K. (2002) Changing economic and social roles: the experience of four cohorts of mid-life individuals in Britain, 1985-2000. Population Trends (110): 19-30. | Not impact on HB |
| Evans GL, McNeil LH, Laufman L et al. (2009). Determinants of low-fat eating behaviors among midlife African American women. Journal of Nutrition Education & Behavior 41(5): 327-333. | X-sectional |
| Falba T. (2005) Health events and the smoking cessation of middle aged Americans. Journal of Behavioral Medicine 28(1): 21-33. | With existing disease |
| Finch H. (1997) Physical activity ‘at our age’: qualitative research among young people over the age of 50. Health Education Authority. | V relevant but 1997 |
| Findlay-King LJ. (2008) Understanding sport and physical activity participation in the transition into early mid-life. PhD thesis. | Thesis - find full paper |
| Fogelholm M, Kujala U, Kaprio J et al. (2000) Predictors of weight change in middle-aged and old men. Obesity Research 8(5): 367-373. | Not B/F |
| Ford E, Clark C, Stansfeld SA. (2011) The influence of childhood adversity on social relations and mental health at mid-life. Journal of Affective Disorders 133(1-2): 320-327. | Not HB |
| Fraser GE, Welch A, Luben R et al. (2000) The effect of age, sex, and education on food consumption of a middle-aged English cohort-EPIC in East Anglia. Preventive Medicine 30(1): 26-34. | X-sectional |
| French DP, Marteau TM, Senior V et al. (2000) Perceptions of multiple risk factors for heart attacks. Psychological Reports 87(2): 681-687. | Not specifically HB |
| French DP, Marteau TM, Sutton S et al. (2004) Different measures of risk perceptions yield different patterns of interaction for combinations of hazards: Smoking, family history and cardiac events. Journal of Behavioral Decision Making 17(5): 381-393. | Not specifically HB |
| French DP, Hevey D, Sutton S et al. (2006) Personal and social comparison information about health risk: Reaction to information and information search. Journal of Health Psychology 11(3): 497-510. | Not specifically HB |
| Friel S, Walsh O, McCarthy D. (2004) The financial cost of healthy eating in Ireland. Combat Poverty. | X-sectional |
| Fu SY, Anderson D, Courtney M et al. (2007) The relationship between culture, attitude, social networks and quality of life in midlife Australian and Taiwanese citizens. Maturitas 58(3): 285-295. | Not specifically HB |
| Gallo LC, Troxel WM, Matthews KA et al. (2003) Marital status and quality in middle-aged women: Associations with levels and trajectories of cardiovascular risk factors. Health Psychology 22(5): 453-463. | Risk factors not HB |
| Gaston MH, Porter GK, Thomas VG. (2011) Paradoxes in obesity with mid-life African American women. Journal of the National Medical Association 103(1): 17-25. | X-sectional |
| Gelberg L, Andersen RM, Leake BD. (2000) The behavioral model for vulnerable populations: application to medical care use and outcomes for homeless people. Health Services Research 34:6: 1273-302. | Prevalence of rf but not HB. |
| Geller J, Swetter SM, Leyson J et al. (2006) Crafting a melanoma educational campaign to reach middle-aged and older men. Journal of Cutaneous Medicine & Surgery 10(6): 259-268. | Review but not SR |
| Godino JG, van Sluijs EM, Marteau TM et al. (2012) Effect of communicating genetic and phenotypic risk for type 2 diabetes in combination with lifestyle advice on objectively measured physical activity: protocol of a randomised control trial. BMC Public Health 18;12:444. | Thesis and paper |
| Gollschewski S, Anderson D, Skerman H et al. (2005) Associations between the use of complementary and alternative medications and demographic, health and lifestyle factors in mid-life Australian women. Climacteric 8(3): 271-278. | Prevalence and functioning study |
| Gough B. (2006) ‘Real men don’t diet’: An analysis of contemporary newspaper representations of men, food and health. Social Science & Medicine 64(2): 326–337. | Based on newspaper articles, not written by population of interest |
| Greaves CJ, Sheppard KE, Abraham C et al. (2011) Systematic review of reviews of intervention components associated with increased effectiveness in dietary and physical activity interventions.” BMC Public Health 18;11:119. | SR, review 3 |
| Grunheid, E. (2004) Einflüsse der Einkommenslage auf Gesundheit und Gesundheitsverhalten: Ergebnisse des Lebenserwartungssurveys des BiB. Research report. | German |
| Grzywacz JG. (2000) Work-family spillover and health during midlife: is managing conflict everything? American Journal of Health Promotion 14(4): 236-243. | Health and wellbeing rather than HB |
| Gu MO, Eun Y. (2002) Health-promoting behaviors of older adults compared to young and middle-aged adults in Korea. Journal of Gerontological Nursing 28(5): 46-53. | Separates mid-life people but not really about b/f, X-sect |
| Guan JW, Huang CQ, Li YH et al. (2011) No association between hypertension and risk for Alzheimer’s Disease: a meta-analysis of longitudinal studies.Journal of Alzheimer’s Disease 27(4): 799–807. | Risk - rev 2? |
| Gupta PC, Maulik PK, Pednekar MS et al. (2005) Concurrent alcohol and tobacco use among a middle-aged and elderly population in Mumbai. The National Medical Journal of India 18(2): 88-91. | Association between alcohol tobacco |
| Gustafsson PE, Janlert U, Theorell T et al. (2012) Social and material adversity from adolescence to adulthood and allostatic load in middle-aged women and men: results from the Northern Swedish Cohort. Annals of Behavioral Medicine 43(1): 117-128. | RF not HB |
| Håkansson C, Björkelund C, Eklund M. (2011) Associations between women's subjective perceptions of daily occupations and life satisfaction, and the role of perceived control. Australian Occupational Therapy Journal 58(6): 397-404. | Not HB |
| Hägglin C, Hakeberg M, Ahlqwist M et al. (2000) Factors associated with dental anxiety and attendance in middle-aged and elderly women. Community Dentistry and Oral Epidemiology 28(6): 451-460. | X-sect |
| Hall S, Bishop AJ, Marteau TM. (2003) Increasing readiness to stop smoking in women undergoing cervical screening: Evaluation of two leaflets. Nicotine and Tobacco Research 5(6): 821-826. | Rev 3? |
| Hall S J, Wienman J, Marteau TM. (2004) The motivating impact of informing women smokers of a link between smoking and cervical cancer: the role of coherence. Health Psychology 23 (4) 419-424. | Mean age 42/39, x-sect |
| Hall S, Vogt F, Marteau TM. (2005) A short report: survey of practice nurses’ attitudes towards giving smoking cessation advice. Family Practice 22(6): 614-6. | X-sectional |
| Hall S, Marteau TM. (2007) Practice nurses’ self-reported opportunistic smoking cessation advice in three contexts. Nicotine & Tobacco Research 9(9): 941-945. | X-sectional |
| Hall S, Reid E, Ukoumunne OC et al. (2007) Brief smoking cessation advice from practice nurses during routine cervical smear tests appointments: A cluster randomised controlled trial assessing feasibility, acceptability and potential effectiveness. British Journal of Cancer 96(7): 1057-1061. | Rev 3 int? |
| Hall S, French DP, Marteau TM. (2009) Do perceptions of vulnerability and worry mediate the effects of a smoking cessation intervention for women attending for a routine cervical smear test? An experimental study. Health Psychology 28(2): 258-263. | Not midlife |
| Hamer M, Kivimaki M, Steptoe A. (2012) Longitudinal patterns in physical activity and sedentary behaviour from mid-life to early old age: a substudy of the Whitehall II cohort. Journal of Epidemiology and Community Health, 66(12): 1110-1115. | Not bf to midlife PA |
| Hampson SE, Goldberg LR, Vogt TM. (2006) Forty years on: teachers' assessments of children's personality traits predict self-reported health behaviors and outcomes at midlife. Health Psychology 25(1): 57-64. | Personality on childhood not modifiable |
| Han HR, Kim KB, Kang J et al. (2007) Knowledge, beliefs, and behaviors about hypertension control among middle-aged Korean Americans with hypertension. Journal of Community Health 32(5): 324-342. | Existing hypertension |
| Hannöver W, Köpke D, Hannich HJ. (2010) Perceived barriers to prostate cancer screenings among middle-aged men in north-eastern Germany. Public Health Nursing 27(6): 504–512. | Screening |
| Hare-Bruun H, Togo P, Andersen LB et al. (2011) Adult food intake patterns are related to adult and childhood socioeconomic status. Journal of Nutrition 141(5): 928-34. | Age 37 at follow up |
| Hartman-Stein P.Potkanowicz E. (2003) Behavioral determinants of healthy aging: good news for the baby boomer generation. Online Journal of Issues in Nursing 8(2): 6. | Include for rev 2? |
| Haskell WL, Lee IM, Pate RR et al. (2007) Physical activity and public health: updated recommendation for adults from the American College of Sports Medicine and the American Heart Association. Medicine & Science in Sports & Exercise 39(8): 1423-34. | Not bf |
| Hatch SL, Frissa S, Verdecchia M et al. (2011) Identifying socio-demographic and socioeconomic determinants of health inequalities in a diverse London community: the South East London Community Health (SELCoH) study. BMC Public Health 11;11:861. | Include for alcohol use, mainly x-sectional, not much qualitative data. Exclude?? SM |
| Hayward MD, Miles T P, Crimmins EM et al. (2000) The significance of socioeconomic status in explaining the racial gap in chronic health conditions. American Sociological Review 65: 910-930. | Not b/f |
| He FJ, Li J, Macgregor GA. (2013) Effect of longer-term modest salt reduction on blood pressure. The Cochrane Library 30(4): CD004937. | SR, review 3, intervention not HB |
| Helvik AS, Krokstad S, Tambs K. (2009) Socioeconomic inequalities in hearing loss in a healthy population sample: The HUNT Study. American Journal of Public Health 99(8): 1376-1378. | Hearing loss per se, not b/f |
| Henretta JC. (2010) Lifetime marital history and mortality after age 50. Journal of Aging and Health 22(8): 1198–1212. | Not HB |
| Hevey D, French DP, Sutton S et al. (2004) Effects of assessment procedure on levels of optimistic bias. Psychology and Health 19 (S1): 78-79. | Not HB |
| Hillsdon M, Jones A, Coombes E. (2011) Green space access, green space use, physical activity and overweight. Natural England Commissioned Reports: 067. | Not primary paper, no methodology |
| Ho LH, Tai Y, Chang CM et al. (2013) The image of health food brands, experience recognition and the purchase behavior of middle aged and older people. Pakistan Journal of Nutrition 12(3): 285-290. | Brand image, purchasing, not specically healthy or unhealthy |
| Hoffman GJ, Lee J, Mendez-Luck CA. (2012) Health behaviors among Baby Boomer informal caregivers. Gerontologist 52(2): 219-230 | Not population based |
| Holahan CK, Holahan CJ, Velasquez KE et al. (2011) Purposiveness and leisure-time physical activity in women in early midlife. Women & Health 51(7): 661-675. | Average age 63 |
| Holahan CJ, North RJ, Holahan CK et al. (2012) Social influences on smoking in middle-aged and older women. Psychology of Addictive Behaviors 26(3): 519-526. | X-sect |
| Hollands GJ, Hankins M, Marteau TM. (2010) Visual feedback of individuals' medical imaging results for changing health behaviour. Cochrane Database of Systematic Reviews (1): CD007434. | Intervention, review 3 SR? |
| Hollands GJ, Prestwich A, Marteau TM. (2011) Using aversive images to enhance healthy food choices and implicit attitudes: an experimental test of evaluative conditioning. Health Psychology 30(2): 195-203. | Intervention, not b/f |
| Hollands GJ, Whitwell SC, Parker RA et al. (2012) Effect of communicating DNA based risk assessments for Crohn’s disease on smoking cessation: randomised controlled trial. BMJ 20;345: e4708. | RCT - rev 3? |
| Hollands GJ, Marteau TM. (2013) The impact of using visual images of the body within a personalized health risk assessment: an experimental study. British Journal of Health Psychology 18(2): 263-278. | Mean age 27 |
| Hollands GJ, Sutton S, McDermott MS et al. (2013) Adherence to and consumption of nicotine replacement therapy and the relationship with abstinence within a smoking cessation trial in primary care. Nicotine Tobacco Research 15(9): 1537-1544. | Not bf |
| Holmbäck I, Ericson U, Gullberg B et al. (2010) A high eating frequency is associated with an overall healthy lifestyle in middle-aged men and women and reduced likelihood of general and central obesity in men. British Journal of Nutrition 104(7): 1065-1073. | X-sect |
| Hori Y, Toyoshima H, Kondo T et al. (2003) Gender and age differences in lifestyle factors related to hypertension in middle-aged civil service employees. Journal of Epidemiology 13(1): 38-47. | Not directly HB |
| Howard LM, Bekele D, Rowe M et al. (2013) Smoking cessation in pregnant women with mental disorders: a cohort and nested qualitative study. BJOG*:* An International Journal of Obstetrics & Gynaecology 120(3): 362-370 | Mean age 27, pregnancy |
| Howell LC, McEvatt L. (2005) Urban black women at midlife: a counseling perspective. Journal of Women & Aging 17(4): 43-57. | Not directly HB |
| Hudson AL, Taylor D, Lee KA et al. (2005) Symptom experience and self-care strategies among healthy, midlife African-American women. Journal of National Black Nurses Association 16(2): 6-14. | Not directly HB |
| Hunt K, Ford G, Mutrie N. (2001) Is sport for all? Exercise and physical activity patterns in early and late middle age in the West of Scotland. Health Education 101(4): 151-158. | Not directly HB |
| Hunte HE. (2011) Association between perceived interpersonal everyday discrimination and waist circumference over a 9-year period in the Midlife Development in the United States cohort study. American Journal of Epidemiology 173(11): 1232-1239. | Not directly HB |
| Hyde J, Hankins M, Deale A et al. (2008) Interventions to increase self-efficacy in the context of addiction behaviours: a systematic literature review. Journal of Health Psychology 13(5): 607-623. | Addiction |
| Im EO, Chee W, Lim HJ et al. (2008) Midlife women's attitudes toward physical activity. Journal of Obstetric, Gynecologic, & Neonatal Nursing 37(2): 203-213. | X-sect |
| Im EO, Stuifbergen AK, Walker L. (2010) A situation-specific theory of Midlife Women's Attitudes Toward Physical Activity (MAPA). Nursing Outlook 58(1): 52-58. | Not primary, theory development |
| Im EO, Lee B, Chee W, Stuifbergen A et al. (2011) Attitudes toward physical activity of white midlife women. Journal of Obstetric, Gynecologic & Neonatal Nursing 40(3): 312-321. | X-sect |
| Im EO, Chang SJ, Chee W et al. (2012) Attitudes of women in midlife to web-based interventions for promoting physical activity." Journal of Telemedicine & Telecare 18(7): 419-422. | X-sect |
| Im EO, Chang SJ, Ko Y, Chee W et al. (2012) A national internet survey on midlife women's attitudes toward physical activity. Nursing Research 61(5): 342-352 | X-sect |
| Infurna FJ, Gerstorf D, Zarit SH. (2011) Examining dynamic links between perceived control and health: longitudinal evidence for differential effects in midlife and old age. Developmental Psychology 47(1): 9-18. | Consider rev 2 |
| Iwai N, Yoshiike N, Saitoh S et al. (2000) Leisure-time physical activity and related lifestyle characteristics among middle-aged Japanese. Japan Lifestyle Monitoring Study Group. Journal of Epidemiology 10(4): 226-233. | X-sect |
| Jacobs RJ, Kane MN. (2012) Correlates of loneliness in midlife and older gay and bisexual men. Journal of Gay & Lesbian Social Services 24(1): 40-61. | Loneliness a HB? Not much else in this population, X-sect |
| Janssen E. (2010) Psychosocial correlates of leisure-time walking among Australian adults of lower and higher socio-economic status." Health Education Research 25(2): 316-324. | X-sect |
| Jayalath VH, de Souza RJ, Sievenpiper JL et al. (2013) Effect of dietary pulses on blood pressure: A systematic review and meta-analysis of controlled feeding trials. American Journal of Hypertension 27(1): 56-64. | Review 2/3? |
| Jilcott SB, Evenson KR, Laraia BA et al. (2007) Association between physical activity and proximity to physical activity resources among low-income, midlife women. Preventing Chronic Disease 4(1): A04. | X-sect |
| Johnson C, Zartman J, Gizlice Z et al. (2013) Psychosocial factors related to weight loss among low income midlife women. FASEB Journal 27. | X-sect |
| Jones IR, Papacosta O, Whincup PH et al. (2011) Class and lifestyle 'lock in' among middle-aged and older men: a Multiple Correspondence Analysis of the British Regional Heart Study. Sociology of Health & Illness 33(3): 339-419. | Tracks into older age |
| Jovanović GK, Zezelj SP, Malatestinić D et al. (2010) Diet quality of middle age and older women from Primorsko-Goranska County evaluated by healthy eating index and association with body mass index. Collegium Antropologicum 34 Suppl 2: 155-160 | Rev 2? |
| Jozwiak JL. (2007) The significance of religion on health factors related to aging among American adults using the national survey of midlife development in the United States. Dissertation Abstracts International: Section B: The Sciences and Engineering. 69(1B): 257. | Not much about specific HB? |
| Justine M, Azizan A, Hassan V et al. (2013) Barriers to participation in physical activity and exercise among middle-aged and elderly individuals. Singapore Medical Journal 54(10): 581-586. | X-sect |
| Kaewpan W, Kalampakorn S, Luksamijarulkul P. (2007) Factors related to health-promoting behaviors among Thai middle-aged men. Journal of the Medical Association of Thailand 90(9): 1916-1924. | X-sect |
| Kahler CW, Daughters SB, Leventhal AM et al. (2009) Personality, psychiatric disorders, and smoking in middle-aged adults. Nicotine & Tobacco Research 11(7): 833-841. | X-sect, pers factors |
| Kamon Y, Okamura T, Tanaka T et al. (2008) Marital status and cardiovascular risk factors among middle-aged Japanese male workers: the High-risk and Population Strategy for Occupational Health Promotion (HIPOP-OHP) study. Journal of Occupational Health 50(4): 348-356. | X-sect |
| Kauhanen L, Leino J, Lakka HM et al. (2011) Adverse childhood experiences and risk of binge drinking and drunkenness in middle-aged Finnish men. Advances in Preventive Medicine 2011: 478741. | Not bf |
| Kelaher M, Paul S, Lambert H et al. (2010) Ethnicity, health and health services utilisation in a British study. Critical Public Health 13(3): 231-249. | Health status rather than HB |
| Kellar I, Mann E, Kinmonth AL et al. (2011) Can informed choice invitations lead to inequities in intentions to make lifestyle changes among participants in a primary care diabetes screening programme? Evidence from a randomized trial. Public Health 125(9): 645-52. | Screening |
| Kern ML. (2010) Physical activity, personality, social contexts and health: interjections within a lifestyle perspective. PhD Dissertation. | Thesis |
| Kesäniemi A, Riddoch CJ, Reeder B et al. (2010) Advancing the future of physical activity guidelines in Canada: An independent expert panel interpretation of the evidence. The International Journal of Behavioral Nutrition and Physical Activity 11;7:41. | Not bf? |
| Kesse-Guyot E, Bertrais S, Péneau S et al. (2009) Dietary patterns and their sociodemographic and behavioural correlates in French middle-aged adults from the SU.VI.MAX cohort. European Journal of Clinical Nutrition 63(4): 521-528. | X-sect |
| Kiefe CI, Williams OD, Lewis CE et al. (2001) Ten-year changes in smoking among young adults: are racial differences explained by socioeconomic factors in the CARDIA study? American Journal of Public Health 91(2): 213-218. | Not bf |
| Kilmer G, Bynum L, Balamurugan A. (2010) Access to and use of eye care services in rural arkansas. The Journal of Rural Health 26(1): 30-35. | Eye care services in Arkansas, X-sect |
| Kim J. (2011) The mediating effects of lifestyle factors on the relationship between socioeconomic status and self-rated health among middle-aged and older adults in Korea. International Journal of Aging & Human Development 73(2): 153-173. | Not bf |
| Kim JW, Lee DY, Lee BC et al. (2012) Alcohol and cognition in the elderly: a review. Psychiatry Investigation 9(1): 8-16. | Rev 2? |
| King AC, Castro C, Wilcox S et al. (2000) Personal and environmental factors associated with physical inactivity among different racial-ethnic groups of U.S. middle-aged and older-aged women. Health Psychology 19(4): 354-364. | X-sect |
| Kirk JK, Bell RA, Bertoni AG et al. (2005) A qualitative review of studies of diabetes preventive care among minority patients in the United States, 1993-2003. American Journal of Managed Care 11(6): 349-360. | Mainly people with existing diabetes |
| Kivipelto M, Helkala EL, Laakso MP et al. (2002) Apolipoprotein E _4 Allele, elevated midlife total cholesterol level, and high midlife systolic blood pressure are independent risk factors for late-life Alzheimer Disease. Annals of Internal Medicine 6;137(3): 149-155. | Rev 2? |
| Kivipelto M, Solomon A. (2006) Cholesterol as a risk factor for Alzheimer’s disease – epidemiological evidence. Acta Neurologica Scandinavica 114 (Suppl. 185): 50–57. | Rev 2? |
| Kloppenborg RP, van den Berg E, Kappelle LJ et al. (2008) Diabetes and other vascular risk factors for dementia: Which factor matters most? A systematic review. European Journal of Pharmacology 585(1): 97-108. | Risk factors - rev 2 |
| Koh WP, Yuan JM, Sun CL et al. (2005) Middle-aged and older Chinese men and women in Singapore who smoke have less healthy diets and lifestyles than nonsmokers. Journal of Nutrition 135(10): 2473-2477. | Association between unhealthy behaviours not bf |
| Kowal J, Fortier MS. (2007) Physical activity behavior change in middle-aged and older women: the role of barriers and of environmental characteristics. Journal of Behavioral Medicine 30(3): 233-242. | Thesis |
| Krantz G, Ostergren PO. (2000) Common symptoms in middle aged women: their relation to employment status, psychosocial work conditions and social support in a Swedish setting. Journal of Epidemiology & Community Health 54(3): 192-199. | Health status rather than HB |
| Krueger PM, Saint Onge JM, Chang VW. (2000) Race/ethnic differences in adult mortality: the role of perceived stress and health behaviors. Social Science & Medicine 73(9): 1312-22. | Rf for mortality |
| Kubicka L, Matejcek Z, Dytrych Z et al. (2001) IQ and personality traits assessed in childhood as predictors of drinking and smoking behaviour in middle-aged adults: a 24-year follow-up study. Addiction 96(11): 1615-1628 | IQ and behavioural predictors? |
| Kuh D, Hardy R, Butterworth S et al. (2006) Developmental origins of midlife grip strength: findings from a birth cohort study. Journals of Gerontology Series A-Biological Sciences & Medical Sciences 61(7): 702-706. | Childhood exposure |
| Lacey RE, Cable N, Stafford M et al. (2011) Childhood socio-economic position and adult smoking: are childhood psychosocial factors important? Evidence from a British birth cohort. European Journal of Public Health 21(6): 725-731. | Childhood exposure |
| Lachman ME, Agrigoroaei S. (2010) Promoting functional health in midlife and old age: long-term protective effects of control beliefs, social support, and physical exercise. PLoS One 5(10): e13297. | Not HB |
| Lakshman R, McConville A, How S et al. (2011) Association between area-level socioeconomic deprivation and a cluster of behavioural risk factors: cross-sectional, population-based study. Journal of Public Health 33(2): 234-245. | Adults in general, mean age 47, X-sect |
| Lallukka T, Rahkonen O, Lahelma E et al. (2010) Sleep complaints in middle-aged women and men: the contribution of working conditions and work–family conflicts. Journal of Sleep Research: 19(3): 466-77. | Risk factors for sleep complaints |
| Lambiase MJ, Thurston RC. (2013) Physical activity and sleep among midlife women with vasomotor symptoms. Menopause 20(9): 946-952. | Effect of PA on sleep |
| Laosupap K, Sota C, Laopaiboon M. (2008) Factors affecting physical activity of rural Thai midlife women. Journal of the Medical Association of Thailand 91(8): 1269-1275. | X-sectional |
| LaRusse S, Roberts JS, Marteau TM et al. (2005) Genetic susceptibility testing versus family history-based risk assessment: Impact on perceived risk of Alzheimer disease. Genetics in Medicine 7(1): 48-53. | Not bf |
| Lautenschlager NT, Cox K, Kurz AF. (2010) Physical activity and mild cognitive impairment and Alzheimer’s Disease. Current Neurology and Neuroscience Reports 10(5): 352-358. | Not bf |
| Lavin T, Metcalfe O, Higgins C. (2011) Active travel – healthy lives. The Institute of Public Health in Ireland. | No methodology |
| Lee YM, Park NH, Kim YH. (2006) Process of change, decisional balance, self-efficacy and depression across the stages of change for exercise among middle aged women in Korea. Daehan Ganho Haghoeji 36(4): 587-595. | X-sect |
| Lee SH, Im EO. (2010) Ethnic differences in exercise and leisure time physical activity among midlife women. Journal of Advanced Nursing 66(4): 814-27. | SR older adults |
| Lee Y, Back JH, Kim J et al. (2010) Systematic review of health behavioral risks and cognitive health in older adults. International Psychogeriatrics 22(2): 174-187. | SR for review 2 |
| Letenneur L, Larrieu S, Barberger-Gateau P. (2004) Alcohol and tobacco consumption as risk factors of dementia: a review of epidemiological studies. Biomedicine & Pharmacotherapy 58(2): 95-99. | Review 2? |
| Leung CW, Ding EL, Catalano PJ et al. (2012) Dietary intake and dietary quality of low-income adults in the Supplemental Nutrition Assistance Program. American Journal of Clinical Nutrition 96(5): 977-88. | X-sect |
| Li F, Fisher KJ, Bauman A et al. (2005) Neighborhood influences on physical activity in middle-aged and older adults: a multilevel perspective. Journal of Aging & Physical Activity 13(1): 87-114. | X-sect |
| Li F, Harmer P, Cardinal BJ, Bosworth M et al. (2009) Built environment and 1-year change in weight and waist circumference in middle-aged and older adults: Portland Neighborhood Environment and Health Study. American Journal of Epidemiology 169(4): 401-408. | Barriers and facilitators to weight gain? |
| Li F, Harmer P, Cardinal BJ et al. (2009) Built environment and changes in blood pressure in middle aged and older adults. Preventive Medicine 48(3): 237-241. | Not HB |
| Li KK, Cardinal BJ, Acock AC. (2013) Concordance of physical activity trajectories among middle-aged and older married couples: impact of diseases and functional difficulties. Journals of Gerontology Series B-Psychological Sciences & Social Sciences 68(5): 794-806. | Not bf |
| Lilgendahl JP, McAdams DP. (2011) Constructing stories of self-growth: how individual differences in patterns of autobiographical reasoning relate to well-being in midlife. Journal of Personality 79(2): 391-428. | Health but not HB |
| Lin FR, Thorpe R, Gordon-Salant S et al. (2011) Hearing loss prevalence and risk factors among older adults in the United States. Journals of Gerontology Series A: Biological Sciences & Medical Sciences 66(5): 582-590. | Effect on hearing loss but not bf |
| Lin FR, Maas P, Chien W et al. (2012) Association of skin color, race/ethnicity, and hearing loss among adults in the USA. Journal of the Association for Research in Otolaryngology 13(1): 109-117. | Outcome is hearing loss not related HB/bf |
| Lind E, Joens-Matre RR, Ekkekakis P. (2005) What intensity of physical activity do previously sedentary middle-aged women select? Evidence of a coherent pattern from physiological, perceptual, and affective markers. Preventive Medicine 40(4): 407-419. | Not bf |
| Liu-Ambrose T, Donaldson MG. (2009) Exercise and cognition in older adults: is there a role for resistance training programmes? British Journal of Sports Medicine 43(1): 25–27. | Review 2/3 |
| Loef M, Walach H. (2012) Fruit, vegetables and prevention of cognitive decline or dementia: a systematic review of cohort studies. The Journal of Nutrition, Health & Aging 16(7):626-30. | Review 2 |
| Loef M, Walach H. (2013) Midlife obesity and dementia: meta-analysis and adjusted forecast of dementia prevalence in the United States and China. Obesity 21: E51-E55. | More about prevalence |
| Lorenz FO, Wickrama KA, Conger RD et al. (2006) The short-term and decade-long effects of divorce on women's midlife health. Journal of Health & Social Behavior 47(2): 111-125. | health effects not HB |
| Lovejoy JC, Champagne CM, Smith SR et al. (2001) Ethnic differences in dietary intakes, physical activity, and energy expenditure in middle-aged, premenopausal women: the Healthy Transitions Study. American Journal of Clinical Nutrition 74(1): 90-95. | Ethnicity effects on diet, X-sect? |
| Lu FP, Lin KP, Kuo HK. (2009) Diabetes and the risk of multi-system aging phenotypes: a systematic review and meta-Analysis. PLoS One 4(1): e4144. | Diabetes effect on risk |
| Luncheon C, Zack M. (2011) Health-related quality of life and the physical activity levels of middle aged women, California Health Interview Survey, 2005. Preventing Chronic Disease 8(2): A36. | Effect of PA on HRQoL |
| Macintyre S, Mutrie N. (2004) Socio-economic differences in cardiovascular disease and physical activity: stereotypes and reality. The Journal of the Royal Society for the Promotion of Health 124(2):66-9. | X-sectional |
| Mäkinen T, Kestilä L, Borodulin K et al. (2010) Effects of childhood socio-economic conditions on educational differences in leisure-time physical activity. European Journal of Public Health 20(3): 346-353. | Effect of childhood SES |
| Malmberg JJ, Miilunpalo SI, Vuori IM et al. (2002) A health-related fitness and functional performance test battery for middle-aged and older adults: feasibility and health-related content validity. Archives of Physical Medicine & Rehabilitation 83(5): 666-677. | Measurement of PA |
| Malmberg J, Miilunpalo S, Pasanen M et al. (2005) Characteristics of leisure time physical activity associated with risk of decline in perceived health--a 10-year follow-up of middle-aged and elderly men and women. Preventive Medicine 41(1): 141-150. | Not bf |
| Mann E, Kellar I, Sutton S et al. (2010) Impact of informed-choice invitations on diabetes screening knowledge, attitude and intentions: An analogue study. BMC Public Health 17;10:76. | Screening |
| Margolis R. (2013) Educational differences in healthy behavior changes and adherence among middle-aged Americans. Journal of Health & Social Behavior 54(3): 353-368. | HB response to illness |
| Marques-Vidal P, Arveiler D, Evans A et al. (2000) Patterns of alcohol consumption in middle-aged men from France and Northern Ireland. The PRIME study. European Journal of Clinical Nutrition 54(4): 321-328. | Cross-sectional |
| Marteau TM, Rana S, Kubba A. (2002) Smoking and cervical cancer: A qualitative study of the explanatory models of smokers with cervical abnormalities. Psychology, Health and Medicine, 7(1): 107-109. | Not midlife, specific to hypercholesterolaemia |
| Marteau T, Senior V, Humphries SE et al. (2004) Psychological impact of genetic testing for familial hypercholesterolemia within a previously aware population: A randomized controlled trial. American Journal of Medical Genetics 128A(3): 285-293. | Model development |
| Marteau TM, Dormandy E, Crockett R. (2005) Informed choice: why measuring behaviour is important. Archives of Disease in Childhood 90(5): 546–549. | Letter |
| Marteau T, Dieppe P, Foy R et al. (2006) Behavioural medicine changing our behaviour - a growing body of evidence shows how to make behavioural interventions effective. BMJ 332(7539): 437-438. | Editorial |
| Marteau TM, Weinman J. (2006) Self-regulation and the behavioural response to DNA risk information: A theoretical analysis and framework for future research. Social Science & Medicine 62(6): 1360-1368. | Not midlife, not general population? |
| Marteau TM, Oliver A, Ashcroft RE. (2008) Changing behaviour through state intervention: when does an acceptable nudge become an unacceptable shove? BMJ 337(a2543): 121-122. | Editorial |
| Marteau TM, Hollands GJ, Fletcher PC. (2008) Changing human behaviour to prevent disease: the importance of targeting automatic processes. Science 337(6101): 1492-5. | Not midlife, review not primary study |
| Marteau TM, Munafò MR, Aveyard P et al. (2010) Trial Protocol: Using genotype to tailor prescribing of nicotine replacement therapy: A randomised controlled trial assessing impact of communication upon adherence. BMC Public Health 9;10:680. | Rev 3? |
| Marteau TM, Mann E, Prevost AT et al. (2010) Impact of an informed choice invitation on uptake of screening for diabetes in primary care (DICISION): Randomised trial. BMJ 13;340:c2138. | Screening |
| Marteau TM, French DP, Griffin SJ et al. (2010) Effects of communicating DNA-based disease risk estimates on risk-reducing behaviours. Cochrane Database of Systematic Reviews 6(10): CD007275. | SR, Review 3 |
| Marteau TM, Ogilvie D, Roland M et al. (2011) Judging nudging: can nudging improve population health? BMJ 25;342:d228. | Editorial/analysis |
| Marteau TM, Aveyard P, Munafò MR et al. (2012) Effect on adherence to nicotine replacement therapy of informing smokers their dose is determined by their genotype: A randomised controlled trial. PLoS One 7(4): e35249. | Review 3? |
| Marteau TM, Thorne J, Aveyard P et al. (2013) Financial incentives for smoking cessation in pregnancy: Protocol for a single arm intervention study. BMC Pregnancy and Childbirth 15;13:66. | Rev 3? |
| Martikainen P, Brunner E, Marmot M. (2003) Socioeconomic differences in dietary patterns among middle-aged men and women. Social Science & Medicine 56(7): 1397-1410. | X-sectional |
| Matthews KA, Abrams B, Crawford S et al. (2001) Body mass index in mid-life women: relative influence of menopause, hormone use, and ethnicity. International Journal of Obesity & Related Metabolic Disorders 25(6): 863-873. | Review 2? |
| McCloskey N, McKinley MC, Arveiler D et al. (2012) A comparison of dietary patterns of middle aged men in France and northern Ireland: the PRIME Study. Proceedings of the Nutrition Society 71 (OCE2): E102. | Abstract |
| McDermott MS, Marteau TM, Hajek P. (2011) Effects of a brief cognitive intervention aimed at communicating the negative reinforcement explanation for smoking on relevant cognitions and urges to smoke. Journal of Smoking Cessation 6(2): 112-118. | Review 3? |
| McGee S. (2012) The lived experiences of internally motivated, healthy, middle-aged women. Doctoral dissertation, Walden University. | Thesis - full paper? |
| Michie S, Weinman J, Miller J et al. (2002) Predictive genetic testing: high risk expectations in the face of low risk information. Journal of Behavioral Medicine 25(1): 33-50. | Risk communication |
| Mielke MM, Zandi PP, Shao H et al. (2010) The 32-year relationship between cholesterol and dementia from midlife to late life. Neurology 75(21): 1888-1895. | Review 2? |
| Mills JP, Perry CD, Reicks M. (2011) Eating frequency is associated with energy intake but not obesity in midlife women. Obesity 19(3): 552-559. | X-sect |
| Minich LM, Rospenda KM, Richman JA. (2009) Mental health service utilization and drinking outcomes in a national population sample: Are there racial/ethnic differences? Journal of Addictive Diseases 28:4: 281-293. | Mental health service utilisation |
| Mishra GD, McNaughton SA, Ball K et al. (2010) Major dietary patterns of young and middle aged women: results from a prospective Australian cohort study. European Journal of Clinical Nutrition 64(10): 1125-1133. | X-sect |
| Morgan TK, Williamson M, Pirotta M et al. (2012) A national census of medicines use: a 24-hour snapshot of Australians aged 50 years and older. The Medical Journal of Australia 16;196(1): 50-3. | Medicines |
| Mori K, Suzuki H, Wang DH et al. (2009) Relationship of psychological factors with physical activity stage of change in prime-and middle-aged Japanese. Acta Medica Okayama 63(2): 97-104. | X-sectional |
| Murray M, Pullman D, Rodgers TH. (2003) Social Representations of Health and Illness among ‘Babyboomers’ in Eastern Canada. Journal of Health Psychology 8(5): 485–499; 035215. | Not much specifically about HB |
| Murray ET, Southall H, Aucott P et al. (2012) Challenges in examining area effects across the life course on physical capability in mid-life: findings from the 1946 British Birth Cohort. Health & Place 18(2): 366-374. | Physical capability not PA |
| Murray ET, Ben-Shlomo Y, Tilling K et al. (2013) Area deprivation across the life course and physical capability in midlife: findings from the 1946 British Birth cohort. American Journal of Epidemiology 178(3): 441-450. | No differences between younger and older women |
| Niederdeppe J, Farrelly MC, Nonnemaker J et al. (2010) Socioeconomic variation in recall and perceived effectiveness of campaign advertisements to promote smoking cessation. Social Science & Medicine 72(5): 773-80 | X-sect survey |
| Nixon K. (2006) Alcohol and adult neurogenesis: roles in neurodegeneration and recovery in chronic alcoholism. Hippocampus 16(3): 287–295. | Not HB |
| Oh AY, Zenk SN, Wilbur J et al. (2010) Effects of perceived and objective neighborhood crime on walking frequency among midlife African American women in a home-based walking intervention. Journal of Physical Activity & Health 7(4): 432-441. | Prescribed walking freq so B/F not generally applicable |
| Oliver A, Marteau TM, Ashcroft RE. (2009) Can financial carrots improve health? Journal of Health Services Research Policy 14(1): 1-2. | Editorial |
| Opree SJ, Kalmijn M. (2012) Exploring casual effects of combining work and intergenerational support on depressive symptoms among middle-aged women. Ageing and Society 1(1): 1-17. | Rev 2? |
| Orsini N, Bellocco R, Bottai M et al. (2007) Correlates of total physical activity among middle-aged and elderly women. International Journal of Behavioral Nutrition and Physical Activity 11;4:16. | X-sect |
| Osler M, Tjønneland A, Suntum M et al. (2002) Does the association between smoking status and selected healthy foods depend on gender? A population-based study of 54,417 middle-aged Danes. European Journal of Clinical Nutrition 56(1): 57-63. | X-sect |
| Osler M, McGue M, Lund R et al. (2008) Marital status and twins' health and behavior: an analysis of middle-aged Danish twins. Psychosomatic Medicine 70(4): 482-487. | X-sectional |
| Osler M, Godtfredsen NS, Prescott E. (2008) Childhood social circumstances and health behaviour in midlife: the Metropolit 1953 Danish male birth cohort. International Journal of Epidemiology 37(6): 1367-1374. | Childhood relationship with midlife |
| Osler M, Madsen M, Nybo Andersen AM et al. (2009) Do childhood and adult socioeconomic circumstances influence health and physical function in middle-age? Social Science & Medicine 68(8): 1425-1431. | Health status |
| Pal S, Cheng C, Ho S. (2011) The effect of two different health messages on physical activity levels and health in sedentary overweight, middle-aged women. BMC Public Health 31;11:204. | Intervention - review 3? |
| Park Y, Quinn J, Florez K et al. (2011) Hispanic immigrant women’s perspective on healthy food and the New York City retail food environment: a mixed-method study. Social Science & Medicine 73(1): 13-21. | Not mid-life |
| Parke H, Ashcroft R, Brown R et al. (2013) Financial incentives to encourage healthy behaviour: An analysis of UK media coverage. Health Expectations 16(3): 292-304. | Newspaper coverage |
| Patel R, Lawlor DA, Ebrahim S et al. (2007) Socio-economic position and the use of preventive health care in older British women: a cross-sectional study using data from the British Women's Heart and Health Study cohort. Family Practice 24(1): 7-10. | But X-sectional |
| Paterson DH, Warburton DE. (2010) Physical activity and functional limitations in older adults: a systematic review related to Canada's Physical Activity Guidelines. International Journal of Behavioral Nutrition and Physical Activity 11;7:38. | For review 2 |
| Patrick JH, Stahl ST. (2009) Understanding disordered eating at midlife and late life. The Journal of General Psychology 136(1): 5-20. | Eating disorders |
| Pechey R, Jebb SA, Kelly MP et al. (2013) Socioeconomic differences in purchases of more vs. less healthy foods and beverages: analysis of over 25,000 British households in 2010. Social Science & Medicine 92: 22-36. | X-sect |
| Pechey R, Spiegelhalter D, Marteau TM. (2013) Impact of plain packaging of tobacco products on smoking in adults and children: an elicitation of international experts' estimates. BMC Public Health 9;13:18. | Policy related - plain packaging of cigarettes |
| Perrin AE, Simon C, Hedelin G et al. (2002) Ten-year trends of dietary intake in a middle-aged French population: relationship with educational level. European Journal of Clinical Nutrition 56(5): 393-401. | Time trends |
| Peters R, Peters J, Warner J et al. (2008) Alcohol, dementia and cognitive decline in the elderly: a systematic review. Age and Ageing 37(5): 505-512. | Review 2 |
| Peters R, Poulter R, Warner J et al. (2008) Smoking, dementia and cognitive decline in the elderly, a systematic review. BMC Geriatrics 23;8:36. | Review 2 |
| Peters R, Beckett N, Forette F et al. (2008) Incident dementia and blood pressure lowering in the Hypertension in the Very Elderly Trial cognitive function assessment (HYVET-COG): a double-blind, placebo controlled trial. Lancet Neurology 7(8): 683-89. | Review 2 |
| Peters J, Parry GD, Van Cleemput P et al. (2009) Health and use of health services: a comparison between Gypsies and Travellers and other ethnic groups. Ethnicity & Health 14(4): 359-377. | Travellers uptake of services/X-sectional |
| Peters R. (2012) Blood pressure, smoking and alcohol use, association with vascular dementia. Experimental Gerontology 47(11): 865-872. | Review 2 |
| Piazza-Gardner AK, Gaffud TJ et al. (2013) The impact of alcohol on Alzheimer's disease: A systematic review. Aging & Mental Health 17(2): 133-146. | Review 2 |
| Pijl M, Timmermans DR, Claassen L et al. (2009) Impact of Communicating Familial Risk of Diabetes on Illness Perceptions and Self-Reported Behavioral Outcomes. Diabetes Care 32(4): 597-599. | Review 3 |
| Pillemer K. (2010) Environmental volunteering and health outcomes over a 20-year period. Gerontologist 50(5): 594-602. |  |
| Power MC, Weuve J, Gagne JJ et al. (2011) The association between blood pressure and incident Alzheimer disease: a systematic review and meta-analysis. Epidemiology 22(5): 646–659. | Review 2 |
| Profenno LA, Porsteinsson AP, Faraone SV. (2010) Meta-analysis of Alzheimer’s Disease risk with obesity, diabetes, and related disorders. Biological Psychiatry 67(6): 505-512. | Review 2 |
| Promberger M, Brown RC, Ashcroft RE et al. (2011) Acceptability of financial incentives to improve health outcomes in UK and US samples. Journal of Medical Ethics 37(11):682-7. | Review but not SR |
| Promberger M, Dolan P, Marteau TM. (2012) ‘Pay them if it works’: discrete choice experiments on the acceptability of financial incentives to change health related behaviour. Social Science & Medicine 75(12): 2509-2514. | Intervention - more for review 3? |
| Promberger M, Marteau TM. (2013) When do financial incentives reduce intrinsic motivation? Comparing behaviors studied in psychological and economic literatures. Health Psychology 32(9): 950-957. | Not specifically HB |
| Pullen C, Noble Walker S. (2002) Midlife and older rural women's adherence to U.S. Dietary Guidelines across stages of change in healthy eating. Public Health Nursing 19(3): 170-178. | X-sect |
| Råberg Kjøllesdal MK, Holmboe-Ottesen G, Wandel M. (2010) Associations between food patterns, socioeconomic position and working situation among adult, working women and men in Oslo. European Journal of Clinical Nutrition 64(10): 1150-1157. | X-sectional |
| Rasmussen M, Holstein BE, Due P. (2012) Tracking of overweight from mid-adolescence into adulthood: consistent patterns across socio-economic groups. European Journal of Public Health 22(6): 885-7. | Review 2? |
| Reid JL, Hammond D, Driezen P. (2010) Socio-economic status and smoking in Canada, 1992-2006: Has there been any progress on disparities in tobacco use? Canadian Joyrnal of Public Health 101(1): 73-78. | Prevalence trends |
| Reiner M, Niermann C, Jekauc D et al. (2013) Long-term health benefits of physical activity – a systematic review of longitudinal studies.” BMC Public Health 8;13:813. | SR, rev 2 |
| Rickards T, Wuest J. (2006) The process of losing and regaining credibility when coming-out at midlife. Health Care for Women International 27(6): 530-547. | Not really impact on health behaviours |
| Ridley NJ, Draper B, Withall A. (2013) Alcohol-related dementia: an update of the evidence. Alzheimer’s Research & Therapy 25;5(1):3. | Rev 2? |
| Rooks RN, Wiltshire JC, Elder K et al. (2011) Health information seeking and use outside of the medical encounter: is it associated with race and ethnicity? Social Science & Medicine 74(2): 176-84. | X-sectional |
| Roper ASW. (2002) Exercise attitudes and behaviours: a survey of adults age 50-79. AARP. | X-sect |
| Rosenberg DE, Huang DL, Simonovich SD et al. (2013) Outdoor built environment barriers and facilitators to activity among midlife and older adults with mobility disabilities. Gerontologist 53(2): 268-279. | Mean age 67 |
| Rotem M, Epstein L, Ehrenfeld M. (2009) Does the conservation of resources motivate middle-aged women to perform physical activity? Western Journal of Nursing Research 31(8): 999-1013. | But cross-sectional |
| Rundle A, Field S, Park Y et al. (2008) Personal and neighborhood socioeconomic status and indices of neighborhood walk-ability predict body mass index in New York City. Social Science & Medicine 67(12): 1951-1958. | Not barriers and facilitators |
| Saito Y, Oguma Y, Inoue S et al. (2013) Environmental and individual correlates of various types of physical activity among community-dwelling middle-aged and elderly Japanese. International Journal of Environmental Research & Public Health 10(5): 2028-2042. | X-sectional |
| Sakalauskiene Z, Maciulskiene V, Vehkalahti MM et al. (2009) Characteristics of dental attendance among Lithuanian middle-aged university employees. Medicina 45(4): 312-9. | Determinants of dental attendance in Lithuania, 35-44 years old, X-sect |
| Sakalauskienė Z, Vehkalahti MM, Murtomaa H et al. (2011) Factors related to gender differences in toothbrushing among Lithuanian middle-aged university employees. Medicina 47(3): 180-6. | Determinants of oral care in Lithuania, X-sect |
| Savva GM, Stephan BC; Alzheimer's Society Vascular Dementia Systematic Review Group. (2010) Epidemiological studies of the effect of stroke on incident dementia: a systematic review. Stroke 41(1): e41-6. | Rev 2? |
| Scales J, Scase R. (2000) Fit and Fifty?: a report prepared for the Economic and Social Research Council, August 2000. Economic and Social Research Council. | Social characteristics of 50-59 years but not health behaviours |
| Scarmeas N, Stern Y, Mayeux R et al. (2006) Mediterranean diet, Alzheimer Disease, and vascular mediation. Archives of Neurology 63(12): 1709-17. | Rev 2? |
| Scroggs NH. (2010) Life patterning of women in midlife transition. Dissertation. | Not HB |
| Secker J, Bowers H, Webb D et al. (2005) Theories of change: what works in improving health in mid-life? Health Education Research 20(4): 392-401. | Very broad and general, difficult to get to specific information about health behaviour |
| Senior V, Weinman J, Marteau TM. (2002) The influence of perceived control over causes and responses to health threats: A vignette study. British Journal of Health Psychology 7(2): 203-211. | Not bf |
| Senior V, Marteau T. (2004) Causal models of raised cholesterol and perceptions of effective risk-reduction: Self-regulation strategies for increased risk of cardiovascular disease. Psychology and Health 19(Supp1): 156. | Abstract only |
| Senior V, Marteau TM. (2007) Causal attributions for raised cholesterol and perceptions of effective risk-reduction: Self-regulation strategies for an increased risk of coronary heart disease. Psychology and Health 22(6): 699-717. | Not lifestyle behaviours |
| Shahab L, Hall S, Marteau T. (2007) Showing smokers with vascular disease images of their arteries to motivate cessation: a pilot study. British Journal of Health Psychology 12(Pt 2): 275-283. | Intervention - more rev 3? |
| Sharp SI, Aarsland D, Day S, et al. (2011) Hypertension is a potential risk factor for vascular dementia: systematic review. International Journal of Geriatric Psychiatry 26(7): 661-9. | More rev 2 |
| Sheeran P, Harris P, Vaughan J et al. (2013) Gone exercising: mental contrasting promotes physical activity among overweight, middle-aged, low-SES fishermen. Health Psychology 32(7): 802-809. | Consider for rev 3 |
| Shropshire J. (1998) Motivating sedentary people to walk. Thesis, Loughborough University/BLDSC. | Thesis |
| Silverwood RJ, Nitsch D, Pierce M et al. (2011) Characterizing longitudinal patterns of physical activity in mid-adulthood using latent class analysis: Results from a prospective cohort study. American Journal of Epidemiology 174(12):1406-15. | Analysis method |
| Skelton D, Young A, Walker A et al. (1999) Physical activity in later life: further analysis of the Allied Dunbar National Fitness Survey and the Health Education Authority National Survey of Activity and Health. Health Education Authority 174(12). | 1999, older people 50+ |
| Sliwińska-Kowalska M, Dudarewicz A, Kotyło P et al. (2006) Individual susceptibility to noise-induced hearing loss: choosing an optimal method of retrospective classification of workers into noise-susceptible and noise-resistant groups. International Journal of Occupational Medicine & Environmental Health 19(4): 235-245. | Classification of susceptibility to noise |
| Smith GD, Chaturvedi N, Harding S et al. (2010) Ethnic inequalities in health: a review of UK epidemiological evidence. Critical Public Health 10(4): 375-408. | Health status rather than health behaviours |
| Smith JM. (2012) Toward a better understanding of loneliness In community-dwelling older adults. The Journal of Psychology 146(3): 293-311. | Not bf |
| Smith NR, Kelly YJ, Nazroo JY. (2012) The effects of acculturation on obesity rates in ethnic minorities in England: evidence from the Health Survey for England. European Journal of Public Health 22(4): 508-13. | Acculturation and obesity, risk factor rather than HB |
| Sofi F, Cesari F, Abbate R et al. (2008) Adherence to Mediterranean diet and health status: meta-analysis. BMJ 11;337:a1344. | SR, not midlife, poss rev 2? |
| Solomon A, Kivipelto M, Wolozin B et al. (2009) Midlife serum cholesterol and increased risk of Alzheimer’s and Vascular Dementia three decades later. Dementia and Geriatric Cognitive Disorders 28(1): 75-80. | Consider for review 2 |
| Stadler G, Oettingen G, Gollwitzer PM. (2009) Physical Activity in Women Effects of a Self-Regulation Intervention. American Journal of Preventive Medicine 36(1): 29-34. | Intervention, consider review 3 |
| Stafford M, Mcmunn A, De Vogli R. (2012) Neighbourhood social environment and depressive symptoms in mid-life and beyond. Ageing & Society 31(6): 893–910. | Determinants of depression in midlife |
| Steptoe A, Marmot M (2003) Burden of psychosocial adversity and vulnerability in middle age: associations with biobehavioral risk factors and quality of life. Psychosomatic Medicine 65(6): 1029-1037. | Health status rather than HB |
| Stewart R, White LR, Xue QL et al. (2007) Twenty-six–Year Change in Total Cholesterol Levels and Incident Dementia. Archives Neurology 64(1): 103-107. | Consider for review 2 |
| Strand BH, Cooper R, Hardy R et al. (2011) Lifelong socioeconomic position and physical performance in midlife: results from the British 1946 birth cohort. European Journal of Epidemiology 26(6): 475-483. | Physical performance |
| Strand BH, Mishra G, Kuh D et al. (2011) Smoking history and physical performance in midlife: results from the British 1946 birth cohort. Journals of Gerontology Series A-Biological Sciences & Medical Sciences 66(1): 142-149. | Physical performance not activity levels |
| Strazdins L, D'Souza RM, Clements M et al. (2011) Could better jobs improve mental health? A prospective study of change in work conditions and mental health in mid-aged adults. Journal of Epidemiological Community Health 5(6): 529-34. | More about risk factors for MH than behaviours, consider review 2 |
| Sudo N, Degeneffe D, Vue H et al. (2009) Relationship between needs driving eating occasions and eating behavior in midlife women. Appetite 52(1): 137-146. | X-sect |
| Szoeke CE, Cicuttini FM, Guthrie JR et al. (2006) Factors affecting the prevalence of osteoarthritis in healthy middle-aged women: Data from the longitudinal Melbourne Women's Midlife Health Project. Bone 39(5): 1149-1155. | Consider review 2 |
| Tao Z, Zhong W. (2010) Eating attitudes and weight concern among Chinese middle-age women: A comparison between different age and BMI groups. European Journal of Psychiatry 24(3): 146-157. | Eating disorders |
| Thomas S, Ness RB, Thurston RC et al. (2013) Racial differences in perception of healthy body weight in midlife women: results from the Do Stage Transitions Result in Detectable Effects study. Menopause 20(3): 269-273. | Cross-sectional |
| Thompson D, Batterham AM, Markovitch D et al. (2009) Confusion and conflict in assessing the physical activity status of middle-aged men. PLoS One 4(2): e4337. | Measurement of PA |
| Thornton LE, Bentley RJ, Kavanagh AM. (2010) Individual and area-level socioeconomic associations with fast food purchasing. Journal of Epidemiological Community Health 65(10): 873-80. | X-sectional |
| Topal K, Eser E, Sanberk I et al. (2002) Challenges in access to health services and its impact on quality of life: a randomised population-based survey within Turkish speaking immigrants in London. Health and Quality of Life Outcomes 26;10:11. | Outcomes are more about health status than health behaviours |
| Tsuboi S, Hayakawa T, Kanda H et al. (2009) The relationship between clustering health-promoting components of lifestyle and bone status among middle-aged women in a general population. Environmental Health and Preventive Medicine 14(5): 292-298. | Review 2? |
| Tucker JS, Klein DJ, Elliott MN. (2004) Social control of health behaviors: A comparison of young, middle-aged, and older adults." Journals of Gerontology - Series B Psychological Sciences and Social Sciences 59(4): 147-150. | More about older adults that midlife |
| Tucker LA, Earl BA. (2010) Emotional health and weight gain: a prospective study of midlife women. American Journal of Health Promotion 25(1): 30-35. | More about risk factors than behaviours |
| Turner LW, Wallace LS, Hunt SB et al. (2003) Changes in behavior and behavioral intentions among middle-age women: results from an osteoporosis prevention program. Psychological Reports 93(2): 521-526. | RF for OP rather than HB |
| Vagelatos NT, Eslick GD. (2013) Type 2 diabetes as a risk factor for Alzheimer’s Disease: the confounders, interactions, and neuropathology associated with this relationship. Epidemiologic Reviews 35: 152–160. | Review 2? |
| Van Cleemput P, Parry G. (2001) Health status of gypsy travellers. Journal of Public Health 23(2): 129-134. | Not HB |
| van Gool CH, Kempen GI, Penninx BW et al. (2003) Relationship between changes in depressive symptoms and unhealthy lifestyles in late middle aged and older persons: results from the Longitudinal Aging Study Amsterdam. Age & Ageing 32(1): 81-87. | Depression-smoking relationship |
| van Stralen MM, Lechner L, Mudde AN et al. (2010) Determinants of awareness, initiation and maintenance of physical activity among the over-fifties: a Delphi study. Health Education Research 25(2): 233-247. | Not a primary study, more expert opinion. |
| van Vliet P. (2012) Cholesterol and late-life cognitive decline. Journal of Alzheimer’s Disease 30 S147-162. | Review 2? |
| Vanden Bosch M. (2011) Comparative analysis of the demographic, clinical, and social-cognitive factors associated with physical activity among middle-aged women with and without diabetes. Michigan State University Ph.D. | Not HB |
| Vogt F, Hall S, Marteau T. (2005) Cognitive predictors of GPS' intentions to recommend smoking cessation services to smokers that want to stop smoking. Psychology & Health 20: 283-284. | Not HB |
| Vogt F, Hall S, Marteau T. (2004) Smokers' beliefs about nicotine replacement therapy: A qualitative study. Psychology and Health, 19(Supp 1): 186-187. | In people with existing disease |
| Vogt F, Hall S, Marteau TM. (2005) General practitioners' and family physicians' negative beliefs and attitudes towards discussing smoking cessation with patients: A systematic review. Addiction 100(10): 1423-1431. | SR, not midlife, GP beliefs? |
| Vogt F, Hall S, Marteau T. (2006) Increasing general practitioners' recommendations of NHS-Stop-Smoking-Services: An experiment. Psychology & Health 21: 159-160. | Abstract only |
| Vogt F, Hall S, Marteau TM. (2007) General practitioners’ beliefs about effectiveness and intentions to recommend smoking cessation services: qualitative and quantitative studies. BMC Family Practice 8(1): 39. | Mean age 46 |
| Vogt F, Hall S, Marteau TM. (2007) Understanding why smokers do not want to use nicotine dependence medications to stop smoking: qualitative and quantitative studies. Nicotine & Tobacco Research 10(8): 1405-1413. | Mean age 46 |
| Vogt F, McEwen A, Ashworth M et al. (2008) Understanding smokers' perceptions of the effectiveness of health-related interventions: A repertory grid approach. Psychology & Health 23: 23-24. | Abstract only |
| Vogt F, Ashworth M, Hall S, Sniehotta FF et al. (2010) What underlies the perception that a medical intervention is effective? An exploratory study among smokers. Nicotine and Tobacco Research 12(5): 508-515. | Mean age 36 |
| Vogt F, Hall S, Marteau TM. (2010) Examining why smokers do not want behavioral support to stop smoking. Patient Education and Counseling 79: 160-166. | Mean age 46 |
| Vogt F, Marteau TM. (2012) Perceived effectiveness of stop smoking interventions: Impact of presenting evidence using numbers, visual displays, and different timeframes. Nicotine and Tobacco Research 14(2): 200-208. | More for rev 3? |
| von Stumm S, Deary IJ, Kivimäki M et al. (2011) Childhood behavior problems and health at midlife: 35-year follow-up of a Scottish birth cohort. Journal of Child Psychology & Psychiatry & Allied Disciplines 52(9): 992-1001. | Childhood behaviour problems, not specifically modifiable HB factors |
| Wallace LS, Gupta R. (2003) Predictors of screening for breast and colorectal cancer among middle-aged women. Family Medice 35(5): 349-54. | Screening |
| Wallhagen MI. (2010) The stigma of hearing loss. Gerontologist 50(1): 66-75. | Relevant but in older adults only >60 |
| Wang Y, Chen X. (2011) How much of racial/ethnic disparities in dietary intakes, exercise, and weight status can be explained by nutrition-and health-related psycosocial factors and socioeconomic status among US adults? Journal of the American Dietetic Association 111(12): 1904-1911. | Mediators rather than direct HB |
| Wardle J, Steptoe A. (2003) Socioeconomic differences in attitudes and beliefs about healthy lifestyles. Journal of Epidemiology and Community Health 57(6): 440–443. | Not midlife, broad age range |
| Watkinson C, van Sluijs EM, Sutton S et al. (2010) Randomised controlled trial of the effects of physical activity feedback on awareness and behaviour in UK adults: the FAB study protocol [ISRCTN92551397]. BMC Public Health 18;10(1):144. | More for review 3 |
| Weinstein G, Wolf PA, Beiser AS et al. (2013) Risk estimations, risk factors, and genetic variants associated with Alzheimer's disease in selected publications from the Framingham Heart Study. Journal of Alzheimer’s Disease 33: S439-S445. | Consider review 2 |
| Weismayer C, Anderson JG, Wolk A et al. (2006) Changes in the stability of dietary patterns in a study of middle-aged Swedish women. Journal of Nutrition 136(6): 1582-1587. | Trends rather than bf |
| Wennberg P, Andersson T, Bohman M. (2000) Associations between different aspects of alcohol habits in adolescence, early adulthood, and early middle age: a prospective longitudinal study of a representative cohort of men and women. Psychology of Addictive Behaviors 14(3): 303-307. | More rev 2? |
| West R, Marteau T. (2013) Commentary on Casswell (2013): the commercial determinants of health. Addiction 108(4): 686-687. | Commentary |
| Weuve J, Tchetgen EJT, Glymour MM et al. (2012) Accounting for bias due to selective attrition: The example of smoking and cognitive decline. Epidemiology 23(1): 119–128. | Analysis/methodology |
| Whaley DE. (2003) Future-oriented self-perceptions and exercise behavior in middle-aged women. Journal of Aging and Physical Activity 11(1): 1-17. | Not clearly BF |
| Whalley LJ, Dick FD, McNeil G. (2006) A life-course approach to the aetiology of late-onset dementias. Lancet Neurology 5: 87-96 | Consider review 2? |
| White IR, Blane D, Morris JN. (1999) Educational attainment, deprivation-affluence and self-reported health in Britain : a cross sectional study. Journal of Epidemiological Community Health 53(9): 535–541. | 1999 |
| White L. (2010) Educational attainment and mid-life stress as risk factors for dementia in late life. Brain 133: 2180-2184. | Review 2 |
| Whitwell SC, Mathew CG, Lewis CM. (2011) Trial Protocol: Communicating DNA-based risk assessments for Crohn’s disease: a randomised controlled trial assessing impact upon stopping smoking. BMC Public Health 2011, 11:44. | More rev 3 |
| Wilbur J, Vassalo A, Chandler P et al. (2005) Midlife women's adherence to home-based walking during maintenance. Nursing Research 54(1): 33-40. | Rev 2? |
| Wilbur J, Miller AM, McDevitt J et al. (2006) Menopausal status, moderate –intensity walking, and symptoms in midlife women. Research and Theory for Nursing Practice: An International Journal, Summer 19(2): 163-80. | Menopausal symptoms |
| Wilcox S, King AC. (2000) Self-favoring bias for physical activity in middle-aged and older adults." Journal of Applied Social Psychology 30(9): 1773-1789. | Not HB |
| Will JC, Farris RP, Sanders CG et al. (2004) Health promotion interventions for disadvantaged women: overview of the WISEWOMAN projects. Journal of Women’s Health Volume 13(5): 484-502. | Rev 3? |
| Williams A, Fries J, Koppem J et al. (2010) Connecting and giving: a report on how mid-life and older Americans spend their time, make connections and build communities. AARP. | Existing MH |
| Williams J. (2003) Women at the crossroads: a literature review of the mental health risks facing women in mid-life. Mental Health Foundation. | Occupation/mental health |
| Willis SL., Martin M, Rocke C. (2010) Longitudinal perspectives on midlife development: stability and change. European Journal of Ageing 7(3): 131–134. | Not HB |
| Wilmoth JR, Boe C, Barbieri M. (2010) Geographic differences in life expectancy at age 50 in the United States compared with other high income countries. International differences in mortality at older ages: Dimensions and sources, 333-366. | Not bf |
| Wilson K. (2006) Baby boomer health dynamics: how are we aging? Canadian Journal on Aging 25: 419-421. | Book review |
| Wilson LA, Giles-Corti B, Burton NW et al. (2011) The association between objectively measured neighborhood features and walking in middle-aged adults. American Journal of Health Promotion 25(4): e12-21. | X-sect |
| Wilson D, Peters R, Ritchie K et al. (2011) Latest advances on interventions that may prevent, delay or ameliorate dementia. Therapeutic Advances in Chronic Disease 2(3): 161-173. | Rev 2/3? |
| Wingfield A, Panizzon M, Grant MD et al. (2007) A twin-study of genetic contributions to hearing acuity in late middle age. Journals of Gerontology Series A: Biological Sciences & Medical Sciences 62A(11): 1294-1299. | Not HB |
| Winpenny E, Marteau TM, Nolte E. (2012) Youth exposure to online alcohol advertising in the UK through social media websites, 2010-12. European Journal of Public Health 22: 224. | Abstract |
| Wirth CK. (2011) Weight concerns and weight loss practices of Baby Boomer men." Dissertation Abstracts International: Section B: The Sciences and Engineering 72(2 B): 834. | Thesis |
| Woodside JV, Yarnell JW, Patterson CC et al. (2012) Do lifestyle behaviours explain socioeconomic differences in all-cause mortality, and fatal and non-fatal cardiovascular events? Evidence from middle aged men in France and Northern Ireland in the PRIME Study. Preventive Medicine 54(3-4): 247-253. | Rev 2 |
| Woollcott M. (2008) Access to primary care services for homeless mentally ill people. Nursing Standard 22(35): 40-4. | X-sect |
| Worsley A, Wang WC, Hunter W. (2010) Baby boomers' food shopping habits. Relationships with demographics and personal values. Appetite 55(3): 466-472. | X-sectional |
| Wray LA, Alwin DF, McCammon RJ et al. (2006) Social status, risky health behaviors, and diabetes in middle-aged and older adults. Journals of Gerontology Series B-Psychological Sciences & Social Sciences 61(6): S290-298. | Not bf, rev 2? |
| Wright AJ, French DP, Weinman J et al. (2006) Can genetic risk information enhance motivation for smoking cessation? An analogue study. Health Psychology 25(6): 740-752. | More rev 3? |
| Wright AJ, Aveyard P, Guo B et al. (2007) Is attributing smoking to genetic causes associated with a reduced probability of quit attempt success? A cohort study. Addiction 102(10): 1657-1664. | Not midlife |
| Wright AJ, Takeichi C, Whitwell SC et al. (2008) The impact of genetic testing for Crohn's disease, risk magnitude and graphical format on motivation to stop smoking: An experimental analogue study. Clinical Genetics 73(4): 306-314. | More rev 3? |
| Wright AJ, Takeichi C, Whitwell SC et al. (2012) Why does genetic causal information alter perceived treatment effectiveness? An analogue study. British Journal of Health Psychology 17(2): 294-313. | More rev 3? |
| Zambón D, Quintana M, Mata P et al. (2010) Higher incidence of mild cognitive impairment in familial hypercholesterolemia. The American Journal of Medicine 123(3): 267-74. | MCI incidence |
