# Supplementary material for: Barriers and Facilitators to the Uptake and Maintenance of Healthy Behaviours by People at Mid-Life: A Rapid Systematic Review
Source: PLoS One. 2016 Jan 27;11(1):e0145074. doi: 10.1371/journal.pone.0145074 (PMC4731386; doi:10.1371/journal.pone.0145074)
Supplement: S2 Table — (DOCX) [file pone.0145074.s003.docx]

Quality Assessment of Included Studies

## 1. Quality Assessment of Systematic Reviews (AMSTAR)

Key: 1. ‘a priori design? 2. Duplicate study selection and data extraction? 3. Comprehensive literature search? 4. Status of publication as an inclusion criterion? 5. List of studies (included and excluded provided)? 6. Characteristics of the included studies provided? 7. Scientific quality of the included studies assessed and documented? 8. Scientific quality of the included studies considered in formulating conclusions? 9. Appropriate method to combine findings? 10. Publication bias? 11. Conflict of interest?

| **Author  (Year)** | **1** | **2** | **3** | **4** | **5** | **6** | **7** | **8** | **9** | **10** | **11** | **Ranking** |
| --- | --- | --- | --- | --- | --- | --- | --- | --- | --- | --- | --- | --- |
| Amireault 2013 | No | No | Yes | Yes | No | Yes | Can't | Can't | Yes | Yes | No | + |
| Babakus 2012 | No | Yes | Yes | Yes | No | Yes | Yes | Yes | Yes | No | No | ++ |
| Bader 2007 | No | Can't | Can't/no | Can't/no | No | No | Can't/no | No | Yes | No | No | - |
| Becares 2012 | No | Can't | Can't/no | No | No | Yes | No | No | Yes | No | No | - |
| Beenackers 2012 | No | No | No | No | No | Yes | No | No | Yes | No | No | - |
| Bisogni 2012 | No | Can't | Can't | Can't | No | No | No | No | Yes | No | No | - |
| Bock 2012 | No | Yes | Can't | No | No | Can't | No | No | N/A | No | No | - |
| Brienza 2002 | No | Can't | Can't | No | No | No | No | No | NA | No | No | - |
| Bryden 2012 | No | Can't/no | Yes | Yes | Yes | Yes | Yes | Yes | Yes | No | No | ++ |
| Bryden 2013 | No | Can't/no | Yes | Yes | Yes | Yes | Yes | Yes | Yes | No | No | ++ |
| Coles 2012 | No | No | Yes | No | No | Yes | Yes | Yes | Can't | No | No | + |
| Daniel 2011 | No | Yes | Can't | Yes | No | Yes | No | No | N/A | No | No | - |
| De Irala-Estevez 2000 | No | Can't | Can't/no | Yes | No | Yes | No | No | Yes | No | No | - |
| Dryden 2012 | No | Can't/no | No | No | No | No | No | No | Yes | No | No | - |
| Engberg 2012 | No | No | No | No | No | Yes | No | No | Yes | No | No | - |
| Eyler 2002 | No | Can't | No | No | No | Can't | No | No | Yes | No | No | - |
| Fischbacher ‘04 | No | No | Yes | No | No | Yes | Can't | Yes | Yes | No | No | + |
| Fleischhacker 2011 | No | Can't | No | No | No | No | No | No | Yes | No | No | - |
| Fransson 2012 | No | No | No | No | No | Can't/no | No | No | Can't | No | No | - |
| Gidlow 2005 | No | Can't | Yes | No | No | Can't | No | No | Can't | No | No | - |
| Gidlow 2006 | No | Can't | Can't | No | No | Yes | Yes | Yes | Yes | No | No | + |
| Giskes 2010 | Yes | Yes | Yes | No | No | Yes | No | No | Can't | No | No | - |
| Giskes 2011 | No | Yes | Can't | No | No | Yes | No | No | Yes | No | No | - |
| Guillaumie 2010 | No | No | Yes | No | No | Can't | No | No | Yes | No | No | - |
| Hart 2005 | No | No | No | No | No | Yes | No | No | Yes | No | No | - |
| Jansen 2012 | No | Yes | Yes | No | No | No | No | No | Yes | No | No | - |
| Kakde 2012 | Can't | Can't | Yes | Yes | Yes | Yes | No | No | Yes | No | No | - |
| Kamphuis 2006 | No | Yes | No | No | No | No | Yes | Yes | Yes | No | No | + |
| Kirk 2011 | No | No | No | No | No | Yes | Yes | Yes | Yes | No | No | + |
| Kurian 2006 | No | Can't | Can't/no | No | No | Yes | No | No | Yes | No | No | - |
| Lachat 2012 | No | Yes | No | No | No | Yes | Yes | No | Yes | No | No | - |
| Lewis 2002 | No | Can't | Can't/no | No | No | Can't/no | No | No | Yes | No | No | - |
| Lovasi 2009 | No | Can't | Yes | No | No | No | No | No | Yes | No | No | - |
| Murray 2012 | Can't | Can't | Can't | No | No | No | Yes | Can't | Yes | No | No | - |
| Niederdeppe’08 | No | Can't | Yes | No | No | No | No | No | Yes | No | No | - |
| Pavey 2002 | No | No | Can't | No | No | Can't/no | No | No | Yes | No | No | - |
| Power 2005 | No | Can't/no | Yes | Yes | No | No | No | No | Yes | No | No | - |
| Rhodes 2012 | No | Yes | Yes | No | No | No | Yes | Can't/no | Yes | No | No | - |
| Rhodes 2013 | No | No | Can't | No | No | Yes | Yes | Yes | Yes | No | No | + |
| Ryan 2009 | No | Can't | Yes | Yes/can't | No | Can't/no | Yes | Yes | Yes | No | No | + |
| Siddiqi 2011 | No | Can't | No | No | No | Yes/can't | Yes | Yes/can't | Yes | No | No | + |
| Trost 2002 | No | Can't/no | Can't | No | No | No | No | No | Yes | No | No | - |
| Vangeli 2011 | Yes | No | Yes | No | No | Yes | No | No | Yes | No | No | - |
| Vrazel 2008 | No | Can't | Yes/can't | No | No | Yes | No | No | Yes | No | No | - |
| Wendell-Vos 2007 | No | Yes | No | No | No | Yes | No | No | Yes | No | No | - |
| Yarcheski 2004 | No | Yes/can't | Can't/no | Yes | No | No | Yes | Yes | Yes | No | No | + |

## 2. Quality Assessment of Cohort Studies

Key to headings – Section 1: Population; 1.1 Source population; 1.2 Eligible population; 1.3 Selected participants or areas. Section 2: Methods of Selection; 2.1 Comparison group; explanatory variables; 2.3 Contamination; 2.4 Confounding factors; 2.5 Setting applicable to the UK. Section 3: Outcomes; 3.1 Reliable outcome measures; 3.2 Outcome measurement; 3.3 Important outcomes assessed; 3.4 Follow-up time in exposure; 3.5 Follow-up time meaningful. NA: Not applicable; NR: Not reported.

|  | **Population** | | | | | | | **Method of selection of exposure**  **(or comparison) group** | | | | | | | **Outcomes** | | | | | | | | |
| --- | --- | --- | --- | --- | --- | --- | --- | --- | --- | --- | --- | --- | --- | --- | --- | --- | --- | --- | --- | --- | --- | --- | --- |
| **Author  (Year)** | **1** | **1.2** | **1.2b** | **1.3.** | **1.3b** | **1.3c** | **1.3d** | **2.1** | **2.2** | **2.3** | **2.3b** | **2.4a** | **2.4b** | **2.5** | **3.1** | **3.1b** | **3.1c** | **3.2** | **3.3** | **3.3b** | **3.4** | **3.4b** | **3.5** |
| Benzies 2008 | – | + | – | – | – | + | – | – | + | NA | NA | + | + | + | – | – | + | – | – | NA | NA | + | ++ |
| Caldwell 2008 | – | – | + | – | ++ | – | + | NR | ++ | NA | NA | – | + | ++ | – | – | – | + | NA | NA | NA | NA | ++ |
| Honjo 2010 | + / - | – / + | + / - | – | ++ | – | – | – | + | NA | NA | + | + | – | – | – | + / - | + / - | – | NA | NA | NA | NA / ++ |
| King 2007 | + | ++ | ++ | ++ | ++ | ++ | ++ | NR | + | NA | NA | – | – | – | + | – | + | – | NA | NA | NA | NA | NA |
| Mejean 2011 | – | – | – | – | – | – | + | – | + | NA | NA | + | + | + | + | – | + | – | + | NA | NA | NA | + |
| Petersson 2008 | + | – | + | + | ++ | + | ++ | + | + | NA | NA | – | – | + | + | – | + | + | + | NA | NA | NA | ++ |
| Segar 2008 | – | – | – | – | – | – | + | – | + | NA | NA | + | + | – | + | + | + | – | – | NA | NA | NA | ++ |
| Shi 2004 | – | – | – | – | + | – | – | – | + | NA | NA | – | + | – | – | – | + | – | – | NA | NA | NA | ++ |
| Sorensen 2005 | – | – | – | – | – | – | – | – | + | NA | NA | + | + | + | – | – | + | – | – | NA | NA | NA | ++ |
| Teixeira 2002 | – | + | – | – | + | – | + | – | + | + | + | + | + | – | + | + | + | – | + | + | NA | – | – |
| Wurm 2010 | + | – | – | + | + | – | – | – | + | NA | NA | + | + | + | – | – | + | + | NA | NA | NA | NA | ++ |
| Yates 2012 | + | – | – | – | – | – | + | – | + | NA | NA | + | + | – | – | + | + | – | – | NA | NA | NA | ++ |

**Quality Assessment for Cohort Studies (continued)**

Key to headings – Section 4: Analyses; 4.1 Powered to; 4.2 Multiple explanatory variables; 4.3 Analytical methods; 4.4 Precision. Section 5: Summary; 5.1 Internal validity; 5.2 External validity. NA: Not applicable; NR: Not reported.

|  | **Analyses** | | | | | | | **Summary** | | |  |
| --- | --- | --- | --- | --- | --- | --- | --- | --- | --- | --- | --- |
| **Author  (Year)** | **4.1** | **4.1b** | **4.2** | **4.3** | **4.4** | **4.4b** | **4.4c** | **5.1** | **5.1b** | **5.2** | **Ranking** |
| Benzies 2008 | – | + | + | – | + | – | – | – | – | + | + |
| Caldwell 2008 | NR | ++ | – | – | + | + | NA | + | ++ | ++ | ++ |
| Honjo 2010 | – | – / + | + | NA | + | + | + | – / + | – / + | + | – |
| King 2007 | NR | ++ | + | + | ++ | ++ | ++ | – | + | + | ++ |
| Mejean 2011 | – | + | + | + | + | + | + | – | – | + | + |
| Petersson 2008 | – | + | + | – | + | + | + | + | – | + | + |
| Segar 2008 | – | – | + | – | + | + | – | – | – | + | + |
| Shi 2004 | – | – | + | – | + | + | – | – | – | + | – |
| Sorensen 2005 | – | + | + | – | + | + | + | – | – | + | + |
| Teixeira 2002 | – | – | + | – | + | + | + | – | + | + | + |
| Wurm 2010 | NR | + | + | ++ | ++ | NR | + | – | – | + | ++ |
| Yates 2012 | + | + | + | – | + | + | ++ | – | – | + | + |

## 3. Quality Assessment of Qualitative Studies

Key: As far as can be ascertained from the paper, how well was the study conducted?

| **Author (year)** | **Ranking** | **Author (year)** | **Ranking** |
| --- | --- | --- | --- |
| Berg 2002 | + | Jilcott 2009 | ++ |
| Brown 2012 | + | Meadows 2001 | ++ |
| Caperchione 2012 | + | Pettinato | *+* |
| DH Insight report 2010 | - | Rimmer 2004 | + |
| Enjezab 2012 | + | Segar 2006 | + |
| Folta 2008 | + | Smith-Dijulio 2010 | + |
| Gower 2013 | + | Vandelanotte 2013 | + |
| Hammond 2010 | + | Vaughn 2009 | + |
| Hooker 2011 | + | Vue 2008 | + |
| Hooker 2012 | + | Withall 2010 | ++ |
| Im 2012 JOGNN | + | Yarwood 2005 | + |
| Im 2013 | + |  |  |
