# Supplementary material for: Barriers and Facilitators to the Uptake and Maintenance of Healthy Behaviours by People at Mid-Life: A Rapid Systematic Review
Source: PLoS One. 2016 Jan 27;11(1):e0145074. doi: 10.1371/journal.pone.0145074 (PMC4731386; doi:10.1371/journal.pone.0145074)
Supplement: S3 Table — (DOCX) [file pone.0145074.s004.docx]

Data Extraction Tables

Systematic Reviews

## Physical Activity/Exercise

| **Authors:** Wendell-Vos W, Droomers M, Kremers S, Brug, van Lenthe F  **Year:** 2007  **Citation:** Obesity Reviews (8): 425-440  **Country of study:** International  **Aim of study:** Systematic review of observational studies on potential environmental determinants of physical activity in adults.  **Study design:** Systematic review (of observational longitudinal (n=3) and cross-sectional (n= 44) studies)  **Quality score: (++, + or -):** - |
| --- |
| **Population and setting** |
| **Source population/s:**  **Country of study:** International, developed countries (46 of 47 included studies in developed countries). **Sample characteristics:**  **Population:** 18 years and older, men and/or women, no specific selection on education level, ethnicity or health conditions (studies on specific health populations e.g. cancer, CVD excluded). Studies in specific ethnic groups and qualitative studies excluded. No information given on individual study population characteristics apart from age range and gender.  **Setting**: All environmental settings considered, defined as everything outside the individual including physical, socio-cultural, economic or political and micro and macro environments |
| **Study design** |
| **Exposure/s description:**  **Physical environment:** environment aesthetics; environment score; accessibility, convenience of facilities; availability, accessibility, convenience of recreational activities; availability of sidewalks, trails; convenience of trails, public transport; streetlights; traffic safety, volume; home age, urban sprawl, coastal location, hills, bad weather, air/noise pollution, satisfaction with neighbourhood and services, recreational facilities, land use mix; urbanisation; coastal location; region; bad weather.  **Socio-cultural environment:** safety, unattended dogs, social isolation, social support, frequency of social contact; having a companion for PA, seeing people exercise, frequency of social contact, quantity of social contact.  **Economic environment:** Costs for PA, household income.  **Political environment**: None found  **Potential confounders:** Not reported |
| **Outcomes and methods of analysis** |
| **Outcomes:** General physical activity, sedentary lifestyle, moderate activity, vigorous activity/sports, combination of moderate/vigorous activity, commuting activities (walking, bicycling or both), bicycling, walking.  **Follow-up periods:** Most studies cross-sectional, only three longitudinal studies included, follow-up of longitudinal studies not reported. |
| **Results** |
| - Only environmental factors showing a ‘convincing’ association are given here. - Social support, having a companion for PA were found to be convincingly associated with different types of physical activity (walking, bicycling, vigorous PA/sports, active commuting, leisure time PA in general, sedentary lifestyle, moderately intense PA and a combination of moderately intense and vigorous PA). - Availability of PA equipment was convincingly associated with vigorous physical activity/sports and connectivity of trails with active commuting. - No evidence was found for differences between men and women.   **Attrition details**: Not reported for individual included studies |
| **Notes by review team** |
| **Limitations identified by author:** 1) Most studies used non-validated measures of environments and/or behaviour. Only a few studies used objective measures of environment and/or physical activity.  2) Diversity of methods reported in included studies to measure PA and environmental attributes.  **Evidence gaps and/or recommendations for future research noted by study author:** More research of better quality is needed with clear, possibly standardised definitions and measurements of physical activity with stronger study designs.  **Source of funding:** Netherlands Organisation for Health Research and Development (Zon Mw) |

| **Authors:** Fransson EI, Heikkila K, Nyberg ST, Zins M, Westerlund H, Westerholm P, Kivimäki M  **Year:** 2012  **Citation:** American Journal of Epidemiology 176(12): 1078-89  **Country of study:** International - European  **Aim of study:** Job strain as a risk factor for leisure time physical activity  **Study design:** Systematic review (individual participant meta-analysis)  **Quality score: (++, + or -):** - |
| --- |
| **Population and setting** |
| **Source population/s:** Eight European countries **Setting:** Data was from 14 European cohort studies, baseline years 1985-1988 to 2006-2008 **Sample characteristics:** 56,735 (prospective analyses) employees (50% women, mean age 43.5 years) **Attrition details:** Participants with missing data on more than half of the items for job demands or job control were excluded from the analysis (n = 1,793, 1% of the total population). |
| **Study design** |
| **Exposure/s description:** Unfavourable work characteristics, job demands and job control **Physical environment:** Workplace **Socio-cultural environment:** Workplace stress **Economic environment**: Job demands, work characteristics, job control **Political environment:** Not reported  **Potential confounders:** Age, sex, SES, smoking status **Inclusion:** Six cohort studies |
| **Outcomes and methods of analysis** |
| **Methods of analysis:** One-stage individual-level meta-analysis to examine prospective associations between work characteristics and leisure-time physical inactivity in six cohort studies. **Outcomes:** Leisure-time physical activity. Measured by self-report in all studies. **Follow-up period:** Leisure time physical inactivity at two-nine years follow up. |
| **Results** |
| - There were increased odds of becoming physically inactive at follow-up among those who had high job strain at baseline (OR= 1.21, 95% CI 1.11, 1.32) or in those with passive jobs (OR 1.20, 95% CI 1.11, 1.30) compared with those who had low strain jobs. (Analyses excluded those who were physically inactive at baseline). - Analysis restricted to those who were physically inactive at baseline showed no clear association between work characteristics at baseline and becoming physically active at follow-up. - Increased odds of having a high-strain or passive job and with decreased odds of having an active or low-strain job at follow-up. - 26% higher odds for inactivity among participants working in high-strain and passive jobs compared with those with low-strain jobs. - No differences in the association between work characteristics and leisure-time physical activity by sex, age, SES, smoking status, or time of the study. |
| **Notes by review team** |
| **Evidence gaps and/or recommendations for future research noted by study author:** Examine whether issues such as social relations, physically demanding work, or sedentary work, economic circumstances, cultural contexts, and length of exposure to work characteristics might modify association between work characteristics and leisure-time physical activity by sex, age, SES, smoking status, or time of the study. **Source of funding:** **Limitations from author:** 1) Inconsistency due to different definitions of physical activity also differences in the categorisation of psychosocial work characteristics.  2) Data based on multi exposure-multi outcome cohort studies that were not specifically designed to measure the impact of work characteristics on physical activity.  3) Leisure-time physical activity was self-reported.  4) Unclear whether these findings are generalisable. |

| **Authors:** Siddiqi Z, Tiro JA, Shuval K  **Year:** 2011  **Citation:** Health Education Research 26(6): 1010-24  **Country of study:** US  **Aim of study:** Understanding impediments and enablers to physical activity among African American adults  **Study design:** Systematic review qualitative studies  **Quality score: (++, + or -):** + |
| --- |
| **Population and setting** |
| SR of qualitative literature of impediments and enablers to physical activity among African Americans. Participants in studies were African American adults (>/= 18 years) living in the US. Studies were excluded if the authors did not analyse and describe findings by race/ethnicity in multi-ethnic studies. |
| **Study design** |
| Studies that explored participants’ impediments and facilitators to physical activity at the individual and/or socio-economic level. |
| **Outcomes and methods of analysis** |
| **Methods of analysis:** Qualitative evidence synthesis. |
| **Results** |
| - 29 articles included in the review. Adults aged 18-50 years. - African American men and women cited lack of time, lack of motivation and lack of knowledge as primary barriers to taking part in physical activity. - Both men and women mentioned specific health conditions such as joint pain or injuries that inhibited activity as hindrances to an active lifestyle. - Hair maintenance was perceived as a barrier specifically by African American women. - Lack of childcare, family responsibilities and monetary costs of joining a fitness club or equipment were perceived as barriers. - Long working hours and hard manual labour were mentioned by both genders. - Neighbourhood safety and lack of parks and open spaces were also barriers. - In older adults >50 years, physical health concerns were inhibiting factors, tiredness, lack of knowledge and motivation. |
| **Notes by review team** |
|  |

| **Authors:** Rhodes RE, Dickau L  **Year:** 2013  **Citation:** British Journal of Sports Medicine 47(4): 215-25  **Country of study:** International  **Aim of study:** Systematic review of the intention-behaviour relationship in the PA domain  **Study design:** Systematic review (of observational longitudinal (n=52) and cross-sectional (n= 8) studies)  **Quality score: (++, + or -):** +  **External validity score: (++, + or -):** |
| --- |
| **Population and setting** |
| **Source population/s:** US 14, Europe 16, Canada 27, Australia 2, China 1.  **Setting:** Universities (n=35), community settings (n= 14), secondary school (n=7), special population (n=2) and others (n=2).  **Sample characteristics:** Sample size, median (min, max), N 228 (56, 3,533)  **Attrition details:** |
| **Study design** |
| **Exposure/s description:**  **Physical environment:** Perceived residence structure, access to retail, commute time, car accessibility, proximity to recreation, infrastructure quality, aesthetics and safety. **Socio-cultural environment:** Intention stability, Past behaviour and habit, Anticipated regret, Perceived behavioural control/self-efficacy, Planning, Cross-behavioural conflict, Neuroticism, Extraversion, Openness to experience and agreeableness, Conscientiousness **Economic environment: Political environment: Potential confounders:** Age, BMI, gender, ethnicity  **Inclusion:** Only English language studies included. Eligible studies had to report an empirical test of moderation of intention-PA with a third variable. |
| **Outcomes and methods of analysis** |
| **Methods of analysis:** Narrative appraisal and quantitative evidence synthesis **Outcomes:** Leisure time PA **Follow-up period:** Duration, median, mode (min, max) four weeks, two weeks (one week, one year) |
| **Results** |
| - 57 studies were included, representing 38 different moderators of the intention-PA. - Mixed evidence for a specific age effect on the I-PA relationship. - No evidence for weight class (BMI) as a moderator. - Gender does not moderate the I-PA relationship. - Ethnicity does not appear to moderate the I-PA relationship. - Intention stability was the most consistent moderator of intention-PA. - Evidence for past behaviour and habit as a moderator of I-PA is inconclusive. - Anticipated regret and conscientiousness were also moderators of intention-PA. Additionally perceived control, self-efficacy, planning, extraversion, habit and environmental proximity to recreation showed some evidence for moderation. - Extraversion may moderate the I-PA relationship - Gender, agreeableness, openness, body mass index and ethnicity did not appear to moderate intention-PA. - Proximity to recreation was identified as a significant moderator of intention-walking relations. |
| **Notes by review team** |
| **Evidence gaps and/or recommendations for future research noted by study author:** Research employing direct assessments of PA **Source of funding:** One of the authors (RER) is supported by a new investigator award from the Canadian Institutes of Health Research, a Senior Scientist Award from the Canadian Cancer Society and with funds from the Social Sciences and Humanities Research Council of Canada, the Canadian Cancer Society and the Canadian Diabetes Association. **Limitations from author:** Self-reported PA, social variables have not received very much research attention as moderators, publication bias. **Limitations from reviewer:** Age of participants is very young. |
| **Authors:** Pavey T, Taylor A, Hillsdon M, Fox K, Campbell J, Foster C…Taylor R.  **Year:** 2012  **Citation:** Journal of Epidemiology and Community Health 66(8): 737-744  **Country of study:** Predominantly UK  **Aim of study:** Systematic review of levels and predictors of exercise referral scheme uptake and adherence  **Study design:** Systematic review of randomised controlled trials (n=6) and observational studies (n=14)  **Quality score: (++, + or -):** - |
| **Population and setting** |
| **Source population/s**: UK (n17). **Setting:** Not reported **Sample characteristics:** Sample sizes ranged from 28 to 6610 participants (median 419). Participants were predominately middle aged (mean age 51-64 years) and female (57-100%). Six observational studies and two RCTs. **Attrition details**: Not reported |
| **Study design** |
| **Exposure/s description:**  **Physical environment:** Deprivation, rural area. **Socio-cultural environment:** Referrer, leisure provider, self-determination, expectations for change, personal development **Economic environment**: Occupation **Political environment:** Not reported **Potential confounders:** Gender, age, diagnosis **Inclusion:** RCTs or observational studies.  Reported a numerical measure of Exercise Referral Scheme uptake or adherence and an estimate of the statistical association between participant demographic or psychosocial factors or programme factors and uptake and adherence to ERS.  Any sig (p<0.05) association between predictive factors and PA was included. |
| **Outcomes and methods of analysis** |
| **Methods of analysis:** Meta-analysis was used to pool data on the levels of uptake and adherence across studies. **Outcomes:** PA uptake, adherence **Follow-up period:** Not reported |
| **Results** |
| - Gender is complex. Women were more likely to begin an ERS but were less likely to adhere to it than men. Older people were more likely to begin and adhere to an ERS. Some studies reported no association between gender and uptake in ERS - Diagnosis is complex. Those with mental health problems were more likely to participate in ERS than those with no specified reason for referral. But those with mental health problems were less likely to participate in ERS than patients with cardiovascular disease or those with physical health problems. Patients referred with a musculoskeletal problem were more likely to participate in ERS. Patients referred for overweight/obesity problems were less likely to participate in ERS than patients with cardiovascular disease. Individuals referred for obesity were more likely to participate in ERS than those referred for smoking. Those with low physical fitness were more likely to participate in ERS than those with no specified referral reason. Patients with respiratory problems and who were most deprived were more likely to participate in ERS. - Increasing age was a predictor of increased ERS adherence in five studies but two studies showed no association. Deprivation, rural area, referrer, leisure provider and occupation were not found to be significant predictors of ERS adherence. - It is unclear if participants who adhered to ERS had significantly higher self-determination. Participants who did not adhere to ERS had significantly higher expectations for change in personal development. |
| **Notes by review team** |
| **Evidence gaps and/or recommendations for future research noted by study author:**  **Source of funding:** This project was funded by the NIHR Health Technology Assessment programme (project number 08/72/01). **Limitations from author:** Statistical heterogeneity in the levels of uptake and adherence across studies **Limitations from reviewer:** Some similar factors were reported in an earlier systematic review (Gidlow et al. 2005) but data has been extracted from Pavey et al. as it is much more up to date. |

| **Authors:** Kirk MA, Rhodes RE  **Year:** 2011  **Citation:** American Journal of Preventive Medicine 40(4): 476-85  **Country of study:** International  **Aim of study:** To critically appraise the relationship between occupation and LTPA status to help identify potential limitations and to outline targets for future LTPA interventions  **Study design:** Systematic review  **Quality score: (++, + or -):** + |
| --- |
| **Population and setting** |
| **Source population/s:** US 18 (29.0), Finland 10 (16.1), Australia 8 (12.9), United Kingdom 6 (9.7), Japan 3 (4.8), Spain 3 (4.8), Poland 2 (3.2), Sweden 2 (3.2), Denmark 2 (3.2), Canada 2 (3.2), Germany 1 (1.6), Greece 1 (1.6), Ireland 1 (1.6), Island Nation of Mauritius 1 (1.6), Nigeria 1 (1.6), Portugal 1 (1.6)  **Setting:** General population **Sample characteristics:** Participants were primarily of both genders (n 52, 83.9%), and sample sizes ranged from 158 participants to 203,120 participants. Both-gender sample n=52 (83.9); Male-only sample n=5 (8.1) and Female-only sample n=5 (8.1).  Occupation variables were most commonly assessed using measures of occupation category/status (n 44, 70.1%), followed by OPA (n 23, 37.1%); weekly work hours (n 18, 29.0%); and psychological work demands (n 12, 19.4%). |
| **Study design** |
| **Exposure/s description:**  **Physical environment:** Not reported **Socio-cultural environment:** Not reported **Economic environment:** Not reported **Political environment:** Not reported **Potential confounders:** Confounding factors such as hours of work, work demands, and work-related physical activity were not accounted for. **Inclusion:** Eligible studies were from English peer-reviewed published articles that examined a relationship between an occupation variable and LTPA status. Studies that measured occupation category/class, work hours, mental work demands, and OPA as independent variables were included. Studies were limited to those examining LTPA behaviour of adults, aged 18–64 years, since this is the standard age range of employment. Excluded studies were those that:  (1) Used a dichotomous employment classifıcation (e.g., employed versus unemployed) because an occupation variable could not be determined; (2) Investigated OPA as the primary outcome measure; (3) Examined clinical populations, because the results may deviate from the general population |
| **Outcomes and methods of analysis** |
| **Methods of analysis:** The common themes and major fındings are discussed and synthesised in a narrative review. Qualitative appraisal of occupation category/status, OPA, work hours, and psychological work demands with LTPA were included. The quantitative appraisal of the studies included summarising effect sizes when the necessary statistical information was available. **Outcomes:** Self-reported physical activity measures 60 (96.8) MLTPAQ 4 (6.7) 7-day PAR 2 (3.3) EPAQ-2 1 (1.7) GLTEQ 1 (1.7) IPAQ 1 (1.7) MAQ 1 (1.7) Study-created self-report measure 50 (83.3) Objective physical activity measure 2 (3.2) **Follow-up periods:** Duration (years; M, min, max) 15.4 (4,30). Most included studies were cross-sectional. |
| **Results** |
| - Quantitative. - Occupation category/status was linked to LTPA status, with the majority of studies indicating that those employed in higher-status occupations had higher levels of LTPA compared to those employed in lower-status occupations. - The fındings indicated that those employed in lower-status occupations (e.g. blue-collar) demonstrated higher total physical activity than professionals. |
| **Notes by review team** |
| **Evidence gaps and/or recommendations for future research noted by study author:**  **Source of funding:** MAK is supported by the Canadian Institutes of Health Research. Frederick Banting and Charles Best Canada Graduate Scholarship and a University of Victoria President’s Research Scholarship. RER is supported by a new investigator award from the Canadian Institutes of Health Research, and with funds from the Social Sciences and Humanities Research Council of Canada, the Canadian Diabetes Association, and the Canadian Cancer Society. **Limitations identified by author:** 1) The heterogeneous measures and fındings from this review highlight the need for focused research.  2) Longitudinal change models that consider mixed factors and test for interactions between occupation and certain sociodemographic profıles would help identify when declines in LTPA most notably occur. **Limitations identified by reviewer:** The majority of studies were cross-sectional (n 51, 82.3%) followed by longitudinal (n 11, 17.7%). |

| **Authors:** Eyler AE, Wilcox S, Matson-Koffman D, Evenson KR, Sanderson B, Thompson J… Rohm-Young D  **Year:** 2002  **Citation:** Journal of Women’s Health & Gender-Based Medicine 11(3): 239-53  **Country of study:** United States or Canada  **Aim of study:** Correlates of physical activity among women from diverse racial/ethnic groups.  **Study design:** Systematic review  **Quality score: (++, + or -):** - |
| --- |
| **Population and setting** |
| **Source population/s:** American: Black, White, Hispanic, Indian and Asian  **Setting:** North America **Sample characteristics:** Not reported **Attrition details:** Not reported |
| **Study design** |
| **Exposure/s description:  Physical environment:** Physical, environmental, and public policy. General physical environment  Bad weather  Lessened daylight hours  Lack of personal safety/crime  Transportation  Lack of public policy  Community resources  Work incentives  Worksite facilities  Provision of child care  Monetary cost **Socio-cultural environment:** Environmental **Social environment:** Social support  Professional support  Physicians/other health professionals Family responsibility  Number of children  Culture issues  Appearance after exercise During workday  Importance of relaxation  Already perceived active enough Acculturation  Social stigma  Language **Economic environment:** Not reported **Political environment:** Not reported  **Potential confounders:** Differences among racial/ethnic groups is often confounded by type of physical activity measured.  Biological/health Perceived health  Health status  Body mass index  Health behaviours Attempting weight loss  Smoking status  Alcohol consumption  Pap smear/breast   Psychological Self-efficacy  Attitudes and beliefs Perceived benefits  Lack of time  Lack of motivation  Fatigue/lack of energy Self-conscious  Negative outcome expected  Competitiveness  Need to excel  Type A/hostility  Enjoyment of exercise  Self-esteem  Stress  Stress reduction  Knowledge Past PA behaviour  **Inclusion:** Studies were included if they:  1. Defined physical activity as leisure time recreational, household, or transportation activities deemed to be at least moderate;  2. Identified correlates of physical activity or exercise;  3. Had a sample of all women or included gender-specific analysis;  4. Had a study population of adults (older than 18 years). |
| **Outcomes and methods of analysis** |
| **Methods of analysis:** The determinations of association were generalised by the outcome and number of studies reviewed for each factor. **Outcomes:** Physical activity  **Follow-up periods:** Not reported |
| **Results** |
| **Quantitative**   - Education was positively related to physical activity. Education was positively associated with sports/exercise and LTPA but was negatively associated with household or caregiving physical activities. - Less consistently than education, age was negatively related in some studies to physical activity in white women, black women, American Indian women, and ethnically diverse samples. - The results of studies examining the relationship between employment and physical activity did not show a consistent pattern. Marital status had an inconsistent relationship with physical activity among women. - Two studies showed that urban residence was not associated with physical activity in samples of black, Hispanic, American Indian, or white women. - Alcohol consumption was inconsistently related to physical activity. - Self-efficacy was positively associated with sport/exercise, but it was negatively associated with household or caregiving activities. Self-efficacy was not related to vigorous sports or exercise or domestic activity in white women. Among black women with diabetes, self-efficacy regarding diabetes care was positively associated with physical activity. - Social support from spouses, family, and friends was consistently correlated with level of physical activity. High levels of perceived social support (family, friends) were associated with greater activity levels in many studies of white women. - Not having an exercise partner was reported to be a barrier to physical activity among black women and among combined samples of both white and black women. - Support from a woman’s physician is a potentially important source of her motivation to become physically active. - Several studies with white women indicated an inverse relationship between having children and family responsibilities and physical activity. Having children was associated with lower physical activity levels in white, black, Hispanic, and American Indian populations. The number of children a woman had was negatively related to physical activity participation in a study of black women and in some studies with white, Hispanic, and American Indian women. - Associations between physical environmental factors and physical activity levels have received relatively little empirical study. Bad weather, lessened daylight hours, lack of personal safety or neighbourhood crime, and lack of transportation were environmental factors that are viewed as barriers to physical activity by black women. - Lack of community resources and neighbourhood environment were other factors influencing physical activity levels of white women. Safety concerns and lack of places to exercise were reported by American Indian women as environmental barriers. - Among white and Hispanic women, the presence of hills in the neighbourhood was positively associated with physical activity. In addition, frequently seeing others exercise was positively associated with physical activity for black women.   **Qualitative**   - Associations between physical activity and income were evaluated less frequently. Low income is perceived by black women to be a barrier to physical activity and it was shown to be associated with lower levels of physical activity in combined samples of black and white women. Lower income was also associated with lower physical activity levels in one study of American Indian women. - In focus groups, black, Hispanic, Asian, and American Indian women identified chronic health concerns as a barrier to physical activity. Physical disability is also viewed as a constraint to physical activity among black women. In contrast, health constraints were negatively associated with sport or exercise but were unrelated to household or caregiving activities in an ethnically diverse sample of women. - Illness was a significant predictor of noncompliance with an exercise program. Medical reasons were the largest predictors of dropout. Health problems were reported as a barrier to physical activity for American Indian women. - Attempting to lose weight was positively associated with physical activity in black and white women. Similarly, in black women, trying to change one’s diet was associated with attempts to become more active. Smoking status was inconsistently related to physical activity. - Among an ethnically diverse population, a negative relationship between smoking and sport/exercise was detected, but no relationship was observed between smoking and household or caregiving activities. - Women who perceive greater benefits of physical activity and fewer barriers are more likely to be physically active. This relationship was shown for black, Hispanic, and white women and in biracial samples. - One attitude commonly reported as a barrier to physical activity is the perception of lack of time. Lack of time has been a reported barrier in samples of white, multi-ethnic, American Indian, and black women. - Lack of motivation was shown to be negatively related to exercise or sport and other physical activities but not to household or caregiving activities in ethnically diverse women and white women. Fatigue or lack of energy was associated with inactivity in older white and Hispanic women, as well as in black women and American Indian women. - The belief that exercise has health or mental health benefits has been shown to be a positive correlate of physical activity in white and black women, one study of white women did not show this association. - The belief that exercise is a physical stressor or could cause injury (termed negative outcome expectations) appeared to be a barrier to physical activity in white and black women. - Focus groups with black women reported that exercise enjoyment was positively related to physical activity. - John Henryism (defined as vigour, tenacity, and self-efficacy) and competitiveness were positively related to physical activity in black and in white women. In addition, the need to excel was positively associated with physical activity in white but not black women. - Stress was a major barrier to physical activity for white women. Perceived stress level was a significant predictor of low levels of physical activity in Hispanic and white women. Stress reduction achieved with exercise was a factor that enhanced compliance with programs. - Past experience or success with physical activity was cited as a facilitator of current levels of physical activity in focus groups of black, Hispanic, Asian, and American Indian women. - Policy incentives are needed to promote physical activity in this population. - The monetary costs associated with physical activity were cited as a barrier to activity by black, white, and American Indian women and by multi-ethnic samples of women. The lack of available facilities or programs for exercise, particularly culturally appropriate programs, was also cited as a barrier by black and American Indian women and by multi-ethnic samples of women. |
| **Notes by review team** |
| **Evidence gaps and/or recommendations for future research noted by study author:**  **Source of funding:** This project was funded through Centers for Disease Control and Prevention Contract U48/CCU710806 and additional funding from the Robert Wood Johnson Foundation. **Limitations identified by author:** 1) Most of the studies were cross-sectional, and 16 were qualitative.  2) Public policy and physical activity have received little study. |

| **Authors:** Engberg E, Alen M, Kukkonen-Harjula K, Peltonen JE, Tikkanen HO, Pekkarinen H.  **Year:** 2012  **Citation:** Sports Medicine 42(5): 433-47  **Country of study:** International  **Aim of study:** Examine the effects of life events on changes in leisure PA by focusing on the following categories: transition to university; change in employment status; marital transitions and changes in relationships; pregnancy/having a child; experiencing harassment at work, violence or disaster; and moving into an institution.  **Study design:** Systematic review  **Quality score: (++, + or -):** - |
| --- |
| **Population and setting** |
| **Source population/s:** Participants in the 34 studies consisted mostly of well-educated Caucasian adults.  **Setting:** None reported. **Sample characteristics:** The studies were published between 1992 and 2012. The sample size varied between 26 and 80,944 participants. The mean age of the study populations ranged from 17 years to 70 years. 19 studies examined both males and females, 12 examined females only, and three males only. **Attrition details:** None reported |
| **Study design** |
| **Exposure/s description:  Physical environment:** Transition to university; disaster; moving into an institution. **Socio-cultural environment:** Marital transitions and changes in relationships (starting a new close personal relationship, starting to live with someone, marriage, separation, divorce, widowhood, interpersonal loss); pregnancy/having a child; experiencing harassment at work, violence (being pushed, grabbed, shoved, kicked or hit). **Economic environment:** Change in employment status (beginning work, changing work conditions, changes in income, retirement). **Political environment:** None reported  **Potential confounders:** None reported **Inclusion:** Articles were excluded if they: (i) Did not include a life event;  (ii) Did not assess a change in PA by assessing PA at two time points at least (before and after the life event); (iii) Assessed a disease as a life-change event;  (iv) Were abstracts or unpublished dissertations. |
| **Outcomes and methods of analysis** |
| **Methods of analysis:** Not reported **Outcomes:** Leisure physical activity **Follow-up periods:** In the prospective cohort studies, the study duration varied from five months to 13 years. In the two included randomised-controlled trials, the study duration was one year and two years. |
| **Results** |
| **Quantitative**   - No relationship between changes in the number of life events and changes in PA was found. - Four studies found an association between transition from high school to university and decreased PA. - Changing conditions at work and reduced income were associated with decreased PA in young women, but with increased PA in middle-aged women. - Three longitudinal studies found no associations between changes in PA and getting married, divorced, separated or widowed. - Among middle-aged women, a family member being arrested or jailed was associated with decreasing PA, while infidelity of a spouse/partner was associated with reduced odds of decreased PA. - Experiencing an interpersonal loss was associated with decreased participation in class-based exercise in older men and women. Participation in home-based exercise was not associated with experiencing an interpersonal loss. - Distressing harassment at work was associated with increased PA in young women. - Being pushed, grabbed, shoved, kicked or hit was associated with decreased PA among middle-aged women.   **Qualitative** |
| **Notes by review team** |
| **Evidence gaps and/or recommendations for future research noted by study author:**  **Source of funding:** Elina Engberg, Juha E Peltonen and Heikki O Tikkanen, were partially funded by The Finnish Funding Agency for Technology and Innovation (40043/07). **Limitations identified by author:** 1) Possible cross contamination of responses when PA levels before and after a life event was assessed at the same time. 2) PA data were not always reported in detail 3) Life events tend to overlap: for example, marriage and pregnancy may closely follow each other. Assessment of the effects of one specific life event on health behaviour is difficult. **Limitations identified by reviewer:** Seven studies were cross-sectional retrospective ones, 25 were prospective longitudinal studies and two were randomised-controlled trials. |

| **Authors:** Babakus WS, Thompson JL  **Year:** 2012  **Citation:** International Journal of Behavioral Nutrition and Physical Activity 9:150  **Country of study:** International  **Aim of study:** Assess what is currently known about the levels of physical activity (PA) and sedentary time (ST) and to contextualize these behaviours among South Asian women with an immigrant background.  **Study design:** Non-systematic Review  **Quality score: (++, + or -):** ++ |
| --- |
| **Population and setting** |
| **Source population/s:** South Asian women  **Setting:** 15 studies were conducted in the UK; six in the US, two in Canada, one in New Zealand, one in Australia/India, one in Guadeloupe and one in Norway. Eight studies obtained samples from large-scale population studies, five recruited from community centres, five from census/birth records or electoral registers, three from general practice lists, two did not state recruitment strategy, two recruited based on postcode and one recruited from a university campus. **Sample characteristics:** Five studies were limited to women, while the remaining included both women and men. Five studies conducted their analyses on men and women as one group; 16 provided analyses by gender **Attrition details:** |
| **Study design** |
| **Exposure/s description:  Physical environment:** Weather **Socio-cultural environment:** Cultural differences  **Economic environment:** Social economic status **Political environment:** Not reported  **Potential confounders:** Employment status, disease status, religion, stress levels and racial discrimination were reported and controlled for in physical activity analyses, although these were not collected in all studies. **Inclusion:** Inclusion criteria were:  (1) Randomised and non-randomised controlled studies, observational and qualitative studies;  (2) Studies that include data on PA and/ or ST; studies on SA;  (3) Studies published from 1980 on to obtain the most current data;  (4) Studies with data on adult women aged 18 and older;  (5) Studies published in English.  Exclusion criteria included: Studies without adult data and studies focusing on migrant groups instead of permanent immigrants, and studies on children. |
| **Outcomes and methods of analysis** |
| **Methods of analysis:** Not reported **Outcomes:** Physical activity and sedentary time, LTPA **Follow-up periods:** One study was observational longitudinal and 25 were cross-sectional designs |
| **Results** |
| - Quantitative. - In three studies respondents reported awareness that they should be participating in regular PA and that it has some general health benefits. Although there was a general awareness, five studies reported that there was confusion as to what types and how much PA to perform as well as confusion about specific health benefits. - Nine studies reported barriers to PA participation among SA. Major barriers were those due to cultural differences with the dominant society and structural barriers. Five studies reported that SA as well as their families and communities would view taking time out to participate in PA as a selfish act. Women reported that in SA culture, a woman’s focus is meant to be on the family and she should perform domestic duties over all other activities - Five studies cited culturally inappropriate facilities as a barrier to PA participation in this population. Examples included mixed-sex facilities such as swimming pools that do not consider the women’s requirement for modesty, and the use of male instructors. Four studies found that women were less likely to participate in PA outside their home if they had difficulties speaking English, the language of the wider society. - Structural barriers such as fear for personal safety were cited in five studies. Many women were worried for their safety if they were to go out into the neighbourhood unaccompanied, while others were fearful of exacerbating an illness or disability by doing too much PA or becoming too tired while out in the neighbourhood alone. - Three studies cited poor weather as the main barrier for low PA participation. Finally, lack of time, money, and access to open spaces were additional structural barriers noted. - A common facilitator seen in all studies was motivation to participate in PA as a way to care for the health of the body and to prevent or alleviate illness and disease. Having exercise equipment in the home was seen as one way to motivate people to be physically active and eliminate several barriers to participation. Education about Muslim faith was also seen as a way to motivate the South Asian community since PA was seen as central to the Muslim way of life. |
| **Notes by review team** |
| **Evidence gaps and/or recommendations for future research noted by study author:** More high quality studies with rigorous study designs and methods are needed to assess levels of PA and ST in this population. **Source of funding:** No funding was received. **Limitations identified by author:** 1) There were no randomised controlled trials available for inclusion in this review, which may indicate that there is not enough high quality evidence on PA or ST in this population from which to draw conclusions. 2) Heterogeneity within SA groups based on country of origin/birth and diversity of socio-economic status (SES), religious beliefs and cultural practices make insights from these studies difficult to generalise and should be interpreted with caution. |

| **Authors:** Gidlow C, Johnston LH, Crone D, James D  **Year:** 2006  **Citation:** Health Education Journal 65(4) 338–367  **Country of study:** International  **Aim of study:** Examine epidemiological evidence to determine if there is strong evidence of a positive gradient of increasing physical activity across the socio-economic strata  **Study design:** Systematic review  **Quality score: (++, + or -):** + |
| --- |
| **Population and setting** |
| **Source population/s:** America (n=16), Australia (n=5), Canada (n=3), Spain (n=1), England (n=3), Finland (n=1), Sweden (n=1), France (n=1), the Netherlands (n=1) and Greece (n=1).  **Setting:** Not reported **Sample characteristics:** Study samples were generally large (range=84 to 61,239; mean=6960, calculated using numbers available for analysis in longitudinal studies). **Attrition details:** Where response rate was reported it was relatively high, with some exceptions (range=31.3 to 97.5; mean=68%, calculated from mean response at baseline and follow-up in longitudinal studies). |
| **Study design** |
| **Exposure/s description:  Physical environment:** Area of residence **Socio-cultural environment:** The physical activity–SEP relationship is thought to be largely dependent on a country’s level of development. **Economic environment:** Regional differences in socio-economic measurement **Political environment:** Not reported  **Potential confounders:** SEP–physical activity relationship might be influenced by gender and age. Relationships between SEP and leisure-time or vigorous intensity activity were stronger in women than men. Age was identified as an important factor in fewer studies and no consistent themes emerged. **Inclusion:** For inclusion, studies were required to meet the following criteria:  1. English language; 2. Published in peer-reviewed journal; 3. Report a recognized socio-economic outcome: social class, income, education, asset-based, or based on area of residence; 4. Report physical activity as a separate outcome; 5. Original study (reviews were excluded); 6. Adult populations (≥16yrs, at baseline if longitudinal); 7. Conducted in Western countries to limit cultural differences. |
| **Outcomes and methods of analysis** |
| **Methods of analysis:** Not reported **Outcomes:** Physical activity is characterised by frequency, intensity, duration and mode Occupational social class Income Education Area of residence **Follow-up periods:** Not reported |
| **Results** |
| **Quantitative**   - All eight cross-sectional studies reported significantly higher physical activity in the highest versus lowest social classes. - The only study to measure social class and physical activity in older adults reported high versus low social class differences for moderate–vigorous intensity activity (P<0.05). - Nine cross-sectional studies found that income and physical activity were positively related, six reported no relationship, and a negative association was reported in one of only two European studies. - Several studies that did not find a gradient observed higher activity in the highest versus lowest income groups. - Out of the two longitudinal studies exploring changes in LTPA, one reported that being in the highest versus lowest income quintile at baseline had a positive effect on subsequent LTPA changes (P<0.01), the other observed a similar positive association that remained only for women when all variables were entered into the model. A total of six studies did not find a significant relationship. One study reported a significant negative relationship. It was conducted in Finland and found higher LTPA in women on lower incomes (P<0.05) but not men. - The majority of cross-sectional studies found positive relationships between education and physical activity; seven did not. Four longitudinal studies measured education. Three reported a positive effect of education on changes in LTPA and habitual physical activity. - Three studies socially stratified by area of residence. Greater decrease in physical activity in residents of poverty areas. Those in low SEP areas were less likely than the high SEP group to meet recommendations for total and vigorous physical activity - Despite finding significant associations for social class and income in the former, researchers failed to find significant differences in moderate–vigorous activity between homeowners and those renting properties.   **Qualitative** |
| **Notes by review team** |
| **Evidence gaps and/or recommendations for future research noted by study author:** Further use of area-level socio-economic measurement in epidemiology. **Source of funding:** Not reported. **Limitations identified by author:** 1) In most studies that specified, non-working adults were excluded from analyses or treated separately. 2) Despite generally large samples (range=1000 to 61,239), all but one study used just three occupational classes. 3) Crude physical activity measurements (a single closed question).  4) Neither longitudinal study reporting occupational class was of high quality. 5) Inconsistency in area classification. **Limitations identified by reviewer:** 1) 28 cross-sectional and five longitudinal studies.  2) Failure to report socio-economic data at follow-up in three out of five longitudinal studies. |

| **Authors:** Becares L, Shaw R, Nazroo J, Stafford M, Albor C, Atkin K… Pickett K  **Year:** 2012  **Citation:** American Journal of Public Health 102(12): e33-66  **Country of study:** International  **Aim of study:** To systematically review the literature examining the ethnic density effect on physical health, mortality, and health behaviours.  **Study design:** Systematic review (57 records included), narrative review  **Quality score: (++, + or -):** - |
| --- |
| **Population and setting** |
| **Source population/s:** The majority (n = 42) of analyses focused on US Blacks, followed by examinations among US Hispanics, which were analysed by 15 studies, UK racial/ethnic minorities (n =6), and other populations (n = 5). **Setting:** International **Sample characteristics:** Studies covered wide range of racial/ethnic and demographic groups.  **Attrition details**: Not reported |
| **Study design** |
| **Exposure/s description:**  **Physical environment:** Studies that included a measure of ethnic density measured at a geographical scale smaller than a US state or equivalent. **Socio-cultural environment:** Ethnic density **Economic environment:** Not reported **Political environment:** Racism **Potential confounders:** Age, individual level deprivation, education, and social class. Socio-economic status, marital status, health care access and insurance, and nativity. **Inclusion:** Studies were included if:  1. Published in journal or book;  2. Sample contained racial/ethnic minority group;  3. Included a measure of ethnic density, measured at a geographical scale smaller than a US state or equivalent, as an independent variable;  4. Included physical morbidity, mortality, or health behaviour as an outcome, measured via self-report or clinical assessment. |
| **Outcomes and methods of analysis** |
| **Methods of analysis:** Contextual analysis **Outcomes:** Mortality **Follow-up period:** Not reported |
| **Results** |
| **UK**   - An exploration of the protective properties of ethnic density against the detrimental association between racism and health reported that, although main effects of ethnic density on self-rated health were not found for any racial/ethnic minority group, a reduction in the odds of reporting poor self-rated health among Pakistani and Indian people who had experienced interpersonal racism was observed as own ethnic density increased. The opposite was found for Black Caribbean people. - Studies only on women found protective effects for Pakistani and Bangladeshi densities, whereby Bangladeshi women living at densities between 5% and 30% were found to have reduced risk of limiting longstanding illness. Pakistani women were found to be protected at all levels of own ethnic density. - A continuous measure of own ethnic density was associated with reduced odds of reporting limiting longstanding illnesses among Black Caribbean people. - All racial/ethnic minority people who perceived greater own ethnic density in their area tended to report less limiting long-term illness, although results were statistically significant only for Bangladeshi people. Caribbean people were found to be more likely to report limiting longstanding illness when living in an area perceived to have high own ethnic density. - In a multilevel analysis on current alcohol consumption and sensible drinking among Black Caribbean, Black African, Indian, Pakistani, and Bangladeshi people, increased own ethnic density was associated with lower odds of reporting current drinking among all racial/ethnic minorities. Protective ethnic density effects were found for sensible drinking among Black African people living in areas of high own ethnic density. - White people were found to be more likely to be current drinkers as their own density increased, and less likely to drink if they lived in a non-White area, although this was only significant in the case of area types characterised as mixed and Black.   **USA**   - Detrimental effects of ethnic density were only found among Black men and women aged between 25 and 44 years. For older groups, a null association was reported between Black ethnic density and mortality. - Two studies reported an age effect, whereby ethnic density was only protective for people aged 65 years and older. - Differing mechanisms of ethnic density occurring across the age spectrum. - Among the five studies that examined the association between BMI and Black ethnic density, one reported a null association, and four reported adverse ethnic density effects. - Possible gender differences in the association with ethnic density and physical morbidity mediating effect of physical disorder on women’s BMI and obesity. - One study found a protective Black ethnic density effect among older Black adults. Protective ethnic density effects among older Black adults were also reported for cancer. The only study to examine the ethnic density effect on hypertension reported a null association. - Foreign-born Black mothers living in areas of high Black ethnic density were more likely to smoke and drink alcohol during pregnancy. - Gender and age effects were reported in an ecological study that found that an increase in ethnic density was associated with a decreased risk of all-cause mortality among Hispanic men aged 25 to 64 years. A null association was found for Hispanic women and for Hispanic men aged 65 years and older. - Association between high Hispanic density and lower age adjusted incidence rate ratios of lung cancer for men and women, breast cancer for women, and colorectal cancer for men. Association between increased Mexican American density and increased consumption of cornbread and flour tortillas, tomatoes, beans, and hot red chili peppers, but a decrease in the consumption of fruits, carrots, and greens. |
| **Notes by review team** |
| **Evidence gaps and/or recommendations for future research noted by study author:** Studies to precisely specify their study population, so that ethnic density effects can be accurately attributed to well-defined populations. Future studies should aim to include, whenever possible given survey data constraints, a measure of perceived ethnic density to their explorations of the ethnic density effect. **Source of funding:** This study was funded by the UK Economic and Social Research Council (ESRC; grant RES-163-25-0043 to M. Stafford) and the Medical Research Council (MRC; grant R1032101 to K. Pickett). L. Bécares is supported by an ESRC/MRC Interdisciplinary Postdoctoral Fellowship (PTA-037-27-0167). **Limitations from author:** 1) Discrepancies in terms of the geographical level of analyses, with studies exploring the ethnic density effect at levels ranging from block group up to counties. 2) Studies differed methodologically in their analytical approach 3) Levels of geography used to measure the ethnic density effect varied greatly across studies. **Limitations from reviewer:** 1) Most studies from USA examined childhood outcomes, including five studies focused on infant mortality, and 21 studies exploring other birth outcomes.  2) Limitations of the literature include inadequate adjustment for area deprivation and limited statistical power across ethnic density measures and study samples. |

| **Authors:** Amireault S, Godin G, Vezina-Im LA  **Year:** 2013  **Citation:** Health Psychology Review 7(1):55-91  **Country of study:** International  **Aim of study:** To identify the psychosocial and socio-demographic determinants of physical activity maintenance (PAM) among adults by examining baseline differences between individuals who did and did not maintain physical activity participation over time (Part-I) and by examining how well combinations of psychosocial constructs and socio-demographic characteristics predict PAM (Part-II).  **Study design:** Systematic review (of longitudinal & experimental studies) & meta-analyses  **Quality score: (++, + or -):** + |
| --- |
| **Population and setting** |
| **Source population/s:** Participants from the general population, university students and clinical samples **Setting:** International **Sample characteristics:** The sample mean age varied between 20.1 and 64.8 years (median 52.2). The majority of the samples included men and women, with two based exclusively on men. Sample size ranged from 23 to 1957 participants (median 78). **Attrition details:** Loss of participants from baseline to follow-up was <20% for nine studies. Among studies reporting a loss to follow-up >20% (s=12). |
| **Study design** |
| **Exposure/s description:**  **Physical environment:** Availability of home equipment **Socio-cultural environment:** Self-efficacy, exercise knowledge, consequences, descriptive norms, depression **Economic environment:** Not reported **Political environment:** Not reported **Potential confounders:** Sample size and the number of predictors, age, BMI, education, gender, marital status and smoking habit **Inclusion:** Studies that examined prospective associations between psychosocial constructs as well as socio-demographic variables and PAM were included. Only studies published since 1980. Studies conducted among trained athletes were excluded. |
| **Outcomes and methods of analysis** |
| **Methods of analysis:** Separate meta-analyses were performed, and the summary standard mean difference for psychosocial constructs and summary odds ratio for socio-demographic characteristics. A meta-analysis was performed and the adjusted overall explained variance (R2) for PAM is reported. **Outcomes:** Physical activity maintenance **Follow-up period:** Not reported |
| **Results** |
| - Overall, 67.7% of the participants maintained their level of physical activity over a median time period of nine months. About three quarters of individuals maintained a self-change in their physical activity (78.0%) over a median time period of 39 weeks (ranged from four to 416 weeks) whereas about half maintained an intervention-induced change in their physical activity (51.8%) over a median time period of 39 weeks (ranged from 13 to 208 weeks). - For all samples, exercise knowledge was not significantly associated with PAM. - The pooled analyses revealed that: maintainers had higher baseline self-efficacy and perceived less barriers compared with those who relapsed; maintainers held more positive attitudes, perceived more positive and less negative consequences for physical activity compared with individuals who relapsed; maintainers had higher levels of intention compared with individuals who relapsed; all studies reported a non-significant association between perceived availability of home equipment/facilities for exercise and PAM; maintainers had similar descriptive norm scores compared with relapsers; maintainers were neither more nor less depressed compared with individuals who relapsed; maintainers were more likely to have participated in a structured exercise programme. - Age, gender, marital status and perceived pain were not significantly associated with the maintenance of physical activity. - BMI, education, income, perceived health status and smoking habit were significantly associated with the maintenance of physical activity. |
| **Notes by review team** |
| **Evidence gaps and/or recommendations for future research noted by study author:**  **Source of funding:** SA is supported by the Training Program in Obesity of the Merck Frosst-Canadian Institute of Health Research (CIHR) Research Chair on Obesity. **Limitations from author:** 1) Small number of studies available to calculate some effect sizes. 2) No adequate statistical power to detect significant differences in some sub-group analyses. 3) Classification of study participants as ‘maintainers’ and ‘relapsers’. 4) The duration of follow-up was often longer than the recall period of the physical activity questionnaire. 5) High lost to follow-up. |

| **Authors:** Beenackers MA, Kamphuis CBM, Giskes K, Brug J, Kunst AE, Burdorf A… Bentley R  **Year:** 2012  **Citation:** International Journal of Behavioral Nutrition and Physical Activity 9(1): 116  **Country of study:** European regions  **Aim of study:** To describe socio-economic inequalities in different domains of physical activity, across different SEP indicators, in men and women, and across different regions in Europe  **Study design:** Systematic review  **Quality score: (++, + or -):** - |
| --- |
| **Population and setting** |
| **Population:** Working age adults (18-65). Studies conducted among the general population (studies of patient groups were excluded). **Settings:** Different regions in Europe. |
| **Study design** |
| **Included:** Studies included were:   1. Studies published between 01 January 2000 and 31 December 2010; 2. Publications in English-written peer-reviewed journals; 3. Studies conducted among the general population; 4. Study participants of working age (18–65); 5. Studies quantitatively assessed the association between at least one SEP indicator and one domain of physical activity. Outcomes included were total physical activity, leisure-time physical activity, active transport, and occupational physical activity.   Manuscripts that elicited concerns about the study quality were excluded. |
| **Outcomes and methods of analysis** |
| Classification of the studies by domain of PA (total, leisure-time including sport, occupational, active transport), indicator of socio-economic position (education, income, occupation), and European region.  As many studies included more than one PA domain and/or more than one SEP indicator, the results were analysed on the level of the separate associations rather than the level of complete studies.  Production of detailed tables in which all the associations reported in the included studies were synthesised. Distributions of reported positive, negative, and null associations were evaluated. |
| **Results** |
| - 131 studies included. These reported on 105 study populations and 447 unique associations between a SEP indicator and PA outcome. Most studies conducted in Scandinavia (n = 47). - Leisure-time PA was the most frequently studied PA outcome (n = 112). Considerable differences in the direction of inequalities were seen for the different domains of PA. Most studies reported that those with high socio-economic position were more physically active during leisure-time compared to those with low socio-economic position (68% positive associations for total leisure-time PA, 76% for vigorous leisure-time PA). - Occupational PA was more prevalent among the lower socio-economic groups (63% negative associations). Socio-economic differences in total PA and active transport PA did not show a consistent pattern (40% and 38% positive associations respectively). Some inequalities differed by European region or socio-economic indicator, however these differences were not very pronounced. - The direction of socio-economic inequalities in PA in Europe differed considerably by domain of PA. The contradictory results for total PA may partly be explained by contrasting socio-economic patterns for leisure-time PA and occupational PA. These inconsistent results in total PA indicate that total PA may not be a suitable summary measure when investigating inequalities in PA and their effects on morbidity and mortality. The inequalities found indicate that leisure-time PA should be an important focus in improving physical activity levels and reducing inequalities. However, interventions aimed at improving leisure-time PA in lower socio-economic groups needs to acknowledge their potential higher levels of occupational PA. |
| **Notes by review team** |
| 1. Potential publication bias. Some relevant studies may have been missed because only English-language studies that were available in electronic databases and that were published in peer-reviewed journals were included. By analysing the data on the level of the associations instead of the level of studies, more weight was given to studies that reported more than one association. 2. Methodological differences between the included studies could have influenced the reported associations. |

| **Authors:** Daniel M, Wilbur J **Year:** 2011  **Citation:** Public Health Nursing 28(5): 389-401  **Country of study:** International  **Aim of study:** To portray the correlates of lifestyle physical activity (PA) behaviour of healthy South Asian Indian (SAI) immigrants comprehensively by identifying, synthesising, and critically analysing the existing research literature.  **Study design:** Integrative review, cross-sectional (n=11) and qualitative (n=4) studies.  **Quality score: (++, + or -):** - |
| --- |
| **Population and setting** |
| **Source population/s:** Healthy South Asian Indian immigrants **Setting:** International **Sample characteristics:** Regardless of the PA measure used, all studies reported low PA levels in at least 40% of the participants. **Attrition details**: Not reported |
| **Study design** |
| **Exposure/s description:**  **Physical environment: Socio-cultural environment:** Background (current health, acculturation, discrimination, social support, environmental) and intrapersonal (motivation), Leisure time **Economic environment:** Household, occupation **Political environment:** Not reported **Potential confounders:** Not reported **Inclusion:** Studies were included if they:  (1) Were published between 1990 and 2009;  (2) Were published in English;  (3) Included adults who identified as SAI and there were sufficient numbers of SAIs for separate analyses if other South Asian groups were included;  (4) Examined correlates of PA behaviour: static and dynamic characteristics. |
| **Outcomes and methods of analysis** |
| **Methods of analysis:** A thematic construction (PA model for SAIs) was used to present an account of findings, disregarding the presentation of weight of evidence in studies. **Outcomes:** PA **Follow-up period:** Mostly cross-sectional |
| **Results** |
| - The correlates of PA most often studied were sociodemographic variables, current health, and acculturation; female sex; poorer health; and less time since immigration. Few studies focused on social support, environmental factors, or included dynamic motivational factors. - Increased knowledge of the factors that impact lifestyle PA is needed so that public health nurses can develop targeted interventions to increase the lifestyle PA of SAI immigrants at risk for cardiovascular disease, diabetes, and central obesity. - PA was higher in those who were male, older, and had higher education and income. Those living in rural communities tended to have more family-oriented PA than those living in urban communities. Racial discrimination may be a barrier to PA in this population, especially among women. Better PA measures are needed in this population. |
| **Notes by review team** |
| **Evidence gaps and/or recommendations for future research noted by study author:** Further examination of background factors identified the notable absence of attention to environmental and social support for PA, factors found in prior work to influence PA behaviour. Lack of studies that looked at intrapersonal factors. **Source of funding:** Not reported **Limitations from author:** 1) None of the studies, however, queried participants’ self-efficacy or confidence in their ability to overcome barriers.  2) Failure to use PA measures that had been tested for reliability and validity. 3) Lack of studies that included an objective measure of PA **Limitations from reviewer:** 11 cross-sectional and four qualitative studies. |

| **Authors:** Vrazel J, Saunders RP, Wilcox S  **Year:** 2008  **Citation:** American Journal of Health Promotion 23(1): 2-12  **Country of study:** USA and Europe  **Aim of study:** Assess the social-Environmental Influences on the Physical-Activity Behaviour of Women  **Study design:** Review of the literature  **Quality score: (++, + or -): -** |
| --- |
| **Population and setting** |
| **Source population/s:** Women  **Setting:** Not reported  **Sample characteristics:** Not reported  **Attrition details**: Not reported.  43 studies - 25 quantitative and 18 qualitative. |
| **Study design** |
| **Exposure/s description:**  **Physical environment**: Not reported.  **Socio-cultural environment:** Social support and social networks. Life transitions and multiple roles, and cultural standards and gender role expectations. Life transitions include key life events such as marriage, parenthood, and employment, and may be associated with multiple roles, such as wife, mother, employee, caretaker, and head of household. Issues related to life transitions and multiple roles include increasing responsibilities that may come with life transitions, reduced discretionary time related to responsibilities, lower priority of physical activity, and belief that activity involved in performing role tasks such as caregiving provides sufficient activity, instrumental direct help or assistance with tasks that are associated with multiple roles, such as household chores or caring for children in order to have time to be physically active, informational support may be provided verbally, through advice and suggestion, or through print or internet sources, i.e. tailored information that focuses on the health benefits of physical activity for women. Health professionals have been identified as an important source of informational support, emotional support - women want encouragement and support for physical activity from important people in their lives, such as spouse, family, and friends. Emotional support from important others has also been identified as a positive factor in exercise adherence; including physician and health care professionals.  **Economic environment**: Not reported  **Political environment:** Not reported  **Potential confounders:** Not reported  **Inclusion:**   1. Studies that focused primarily on adult women aged 20 to 60 years were selected; 2. Or included gender-specific analysis, identified or measured some aspect of the social environment in relation to physical activity; 3. Were published in English within the last two decades. |
| **Outcomes and methods of analysis** |
| **Methods of analysis:** Narrative synthesis.  **Outcomes:** Physical activity.  **Follow-up periods:** Not reported but mostly cross-sectional |
| **Results** |
| - Social support includes aid or assistance exchanged by individuals, groups, or organisations; (social networks) through one of four methods: emotional, instrumental, informational, or appraisal support. Having someone to exercise with was a facilitator to exercise. - Women with more social role constraints had lower levels of self-efficacy for physical activity compared with those who had fewer social-role constraints. As women transition into middle-age years, they may be faced with increased caregiving responsibilities of older adult relatives. Lack of time is one of the most significant barriers, due to multiple roles, such as wife, mother, employee, caregiver and head of household. All women, regardless of race, perceived that family, household, and caregiving responsibilities presented a major barrier to leisure time physical activity. - Women also reported that they did not have structured time compared with their husbands and that the majority of their time is spent doing intermittent, unstructured activities that did not allow time for exercise. Women did not consider themselves to be exercisers. They did consider themselves to be physically active because of their busy schedules, which revolved around social roles and responsibilities. - Lack of acceptance of physical activity from spouses is a major barrier. The support from wider family is also essential. Some women identified that social pressure and a lack of appropriateness of physical activity for women were a barrier to uptake of PA incl. gender-role expectations. Women often conform to caregiving role and put these responsibilities first. - Lack of role models is also a barrier. The scarcity of role models for women adds to a perceived lack of community and social support. |
| **Notes by review team** |
| **Evidence gaps and/or recommendations for future research noted by study author:** Little research has explored the effect of public policy on physical activity in general. Additional research is needed to expand and test the social-environmental framework and strategies based on framework components on physical-activity behaviour in women.  **Source of funding:** Not reported.  **Limitations identified by author:** Not reported  **Limitations identified by reviewer:** Mostly cross-sectional |

| **Authors:** Trost SG, Owen N, Bauman AE, Sallis JF, Brown W  **Year:** 2002  **Citation:** Medicine & Science in Sports & Exercise 34(12): 1996–2001  **Country of study:** Not reported  **Aim of study:** Review and update the evidence relating to the personal, social, and environmental factors associated with physical activity  **Study design:** Systematic review  **Quality score: (++, + or -):** - |
| --- |
| **Population and setting** |
| **Source population/s**: Not reported  **Setting:** Not reported **Sample characteristics:** Not reported **Attrition details:** Not reported |
| **Study design** |
| **Exposure/s description:**  **Physical environment:** Exercise equipment at home, access to facilities, satisfaction with recreation facilities, neighbourhood safety, hilly terrain, frequent observation of others engaging in physical activity, and enjoyable scenery. **Socio-cultural environment:** Age and gender, socio-economic status, and educational attainment, marital status, weight, dietary habits, past exercise behaviour, smoking status, attitudes, barriers to physical activity, enjoyment of physical activity, expected benefits, value of physical activity outcomes, intentions, exercise self-schemata, perceived behavioural control, normative beliefs, knowledge of health and exercise, perceived health, psychological health, self-efficacy, self-motivation, and stage of change. **Economic environment:** Occupational status **Political environment**: Not reported  **Potential confounders:** Occupational and home activity, hours spent sitting down, recent weight change, social class, country of origin, and smoking/ **Inclusion:** Studies were included when:  1. The dependent variable was physical activity, exercise, or exercise adherence;  2. If the study included participants aged 18 years or older.  Studies in which the dependent variable was aerobic fitness, intention, self-efficacy, or other intermediate (non-behavioural) measures were not included. Qualitative reports or case studies were not included. |
| **Outcomes and methods of analysis** |
| **Methods of analysis:** Not reported **Outcomes:** Physical activity **Follow-up periods:** Not reported |
| **Results** |
| - Quantitative. - Physical activity participation was consistently higher in men than in women and was inversely associated with age. - Physical activity self-efficacy emerged as the most consistent correlate of physical activity behaviour. - Socio-economic status, occupational status, and educational attainment were also consistent determinants of physical activity behaviour. - Inclusion of occupational and home activity eliminated the positive association between physical activity and occupational status in men. The inclusion of occupational and home activity had little effect on the association between occupational status and physical activity for women. - The association between marital status and physical activity behaviour produced mixed findings. Some studies reported a positive association between marital status and physical activity participation others reported none. The transition from a single to a married state resulted in significant positive changes in physical activity relative to individuals remaining single. In contrast, the transition from a married to a single state did not influence physical activity. - Overweight or obesity also emerged as a consistent negative influence on physical activity. - Barriers to physical activity - lack of time, too tiring, too weak, fear of falling, bad weather, no facilities, and lack of exercise partners emerged as the strongest influence on leisure time activity for both men and women. Perceived barriers of fatigue, ill health, lack of energy, and self-consciousness about appearance emerged as significant correlates of physical activity. - Past exercise behaviour or exercise habit emerged as a consistent predictor of current activity status. There were positive associations with healthy diet. - Social support emerged as a consistently important correlate. - Six studies examined the impact of urban location on physical activity participation. All of them found physical activity to be significantly lower among adults living in rural areas, although most of the studies assessed leisure time physical activity and not occupational physical activity. |
| **Notes by review team** |
| **Evidence gaps and/or recommendations for future research noted by study author:** 1) Need to determine whether environmental measures add variance to the explanation of behaviour, above that provided by intrapersonal and social and cultural domains. 2) Multiple geographic and cultural settings may need to be studied to achieve sufficient variation in environmental characteristics to study their associations with behaviour 3) Need for future studies to identify the determinants of physical activities and sedentary behaviours in the context of work and daily living 4) More information about physical activity patterns at different life stages **Source of funding:**  Funding for this project was provided by the Commonwealth Department of Health and Aged Care, Canberra, Australia. **Limitations identified by author:**  Not reported **Limitations identified by reviewer:** Due to previous review, limited to 1998. |

| **Authors:** Fischbacher CM, Hunt S, Alexander L  **Year:** 2004  **Citation:** Journal of Public Health 26(3): 250-258  **Country of study:** UK  **Aim of study:** To assess levels of physical activity in South Asian population in the UK  **Study design:** A systematic review of cross-sectional (n=18) and case control (n=1) studies  **Quality score: (++, + or -):** + |
| --- |
| **Population and setting** |
| **Source population/s:** GP lists, PAF, electoral and valuation rolls, secondary schools, etc. **Setting:** UK **Sample characteristics:** Adults (n=12 studies) and children (n=5 studies) **Attrition details:** Response rates between 58 and 90% |
| **Study design** |
| **Exposure/s description:**  **Physical environment:** Not reported **Socio-cultural environment:** Not reported **Economic environment:** Not reported **Political environment:** Not reported **Potential confounders**: Not reported **Inclusion:** Included studies that reported descriptions of patterns of physical activity, estimates of total energy expenditure or levels of physical fitness and which reported results for South Asian ethnic groups separately. The search was limited to papers in English and to studies of populations living in the UK. |
| **Outcomes and methods of analysis** |
| **Methods of analysis:** A descriptive analysis of questionnaires **Outcomes:** 1) physical activities and 2) fitness **Follow-up period:** Not reported |
| **Results** |
| - All studies reported lower levels of physical activity in South Asian groups than in the general population or white groups. The differences were substantial, particularly among women and older people. - Bangladeshis had the lowest and Indians the highest levels of activity. - Bangladeshi women in particular had very low levels of physical activity, reporting 35% of the level of activity of women from the general population. |
| **Notes by review team** |
| **Evidence gaps and/or recommendations for future research noted by study author:** Future research needs to take account of the principles of cross-cultural adaptation for survey questionnaires. Information is also needed on the validity and reliability of translated and adapted survey instruments. **Source of funding:** Not reported **Limitations from author:** 1) Limited information was provided about translation and adaptation of questionnaires. 2) Definition of beneficial level of physical activity not directly comparable **Limitations from reviewer:** Lot of young people included some adults. |

| **Authors:** Lewis BA, Marcus BH, Pate RR, Dunn AL  **Year:** 2002  **Citation:** American Journal of Preventive Medicine 23 (2 Suppl): 26-35  **Country of study:** Not reported  **Aim of study:** Examining theory-based, physical activity mediators  **Study design:** Systematic review  **Quality score: (++, + or -):** - |
| --- |
| **Population and setting** |
| **Source population:** Not reported  **Setting:** Not reported  **Sample characteristics:** Not reported  **Attrition details:** Not reported |
| **Study design** |
| **Exposure/s description:**  **Physical environment:** Not reported  **Socio-cultural environment**: Behavioural processes, cognitive processes, self-efficacy, decisional balance, social support, enjoyment of physical activity  **Economic environment:** Not reported  **Political environment:** Not reported  **Potential confounders:** outcome-expectancy value and self-regulation.  **Inclusion:** Not written in English, studies using non experimental designs, non-theoretical interventions, and intervention studies targeting multiple risk factors. |
| **Outcomes and methods of analysis** |
| **Methods of analysis:** Not reported.  **Outcomes:** Physical activity.  **Follow-up periods**: Not reported. |
| **Results** |
| - Qualitative. - Inconsistent findings. - Most studies indicated that physical activity interventions designed to change behavioural processes significantly increased use of behavioural processes, and increased use of behavioural processes was significantly related to increases in physical activity. Cognitive processes are likely to be important in shaping and changing physical activity behaviour. - Some studies indicate that interventions significantly increase self-efficacy, or that self-efficacy is significantly related to physical activity behaviour, or both, although support for self-efficacy has varied across time point, gender, and outcome variable. - The support for decisional balance as a mediator in physical activity–intervention studies appears mixed. The relationship between social support and physical activity behaviour was inconsistent; however, other correlational studies that did not directly examine the influence of the intervention on the mediator have found social support to be an important predictor of physical activity behaviour. |
| **Notes by review team** |
| **Evidence gaps and/or recommendations for future research noted by study author**: Inadequate measures. Psychometrically sound measurement tools should be used. Studies including control groups and prospective designs are needed. Conduct studies examining mediators among children. Studies to examine the effect of changes in the mediators on physical activity behaviour at a later time point. Studies examining new/different theories.  **Source of funding:** This project was supported in part through grants from the National Heart, Lung, and Blood Institute (HL68422 and HL64342).  **Limitations from author:** Some studies have used shortened versions, adapted versions, or both shortened and adapted versions of previously validated measures of mediators some studies did not find differences between the intervention and control groups. |

## B.2 Sedentary Behaviour

| **Authors:** Rhodes RE, Mark RS, Temmel CP  **Year:** 2012  **Citation:** American Journal of Preventive Medicine 42(3): e3-e28  **Country of study:** International  **Aim of study:** Collect and appraise the current literature on correlates of sedentary behaviours among adults  **Study design:** Systematic review  **Quality score: (++, + or -):** - |
| --- |
| **Population and setting** |
| **Source population/s:** North American (n47), South America (n2); Europe (n17); Australia/New Zealand (n12); Asia (n3); and multicontinental (n1). Prospective (n 8) data sets representing Australia, Spain, France, Canada, Taiwan, Belgium, Scotland, a collection of EU countries, India, and the US.  **Setting:** International **Sample characteristics:** 82 independent samples represented a total of 724,478 participants with sample sizes ranging from 39 to 123,216. The ages of participants ranged between 18 and 91 years. 83 were cross-sectional, 24 followed a prospective design, one was experimental baseline data, and one was cohort design. **Attrition details:** Not reported |
| **Study design** |
| **Exposure/s description:** TV viewing or computer use, time spent sitting **Physical environment:** Urban–rural, environmental walkability, neighbourhood SES **Socio-cultural environment:** Eating behaviour, education, employment, gender, age  **Economic environment:** Car ownership, income, occupational status **Political environment:** Not reported  **Potential confounders**: Not reported  **Inclusion:** Studies featuring a correlate or correlates of sedentary behaviour were included within this review. Papers had to be from peer reviewed, English-language journals Exclusion criteria for this review were pre-established by all three authors. Studies were excluded if they:  (1) Examined child, adolescent, or clinical populations;  (2) Did not include an expression of at least one variable and its relationship to a sedentary behaviour;  (3) Did not include a measure of sedentary behaviour that was independent from physical activity;  (4) Were tests of reliability and validity of sedentary measurement tools/tool development. |
| **Outcomes and methods of analysis** |
| **Methods of analysis:** Correlates were evaluated by signifıcance within the study and then by meeting the minimum magnitude of a small effect size (e.g. d 0.19).  **Outcomes:** TV viewing, screen viewing , computer use, reading, general sitting **Follow-up periods:** Not reported |
| **Results** |
| **Quantitative**   - Evidence was present for sedentary behaviour and correlates of education, age, employment status, gender, BMI, income, smoking status, MVPA, attitudes, and depressive symptoms/ quality of life. - 11/20 found support for a relationship between higher age and corresponding higher hours of TV viewing, and two studies found support for this relationship only for women. - Higher levels of TV viewing were associated with lower values of formal education in 14 of 18 studies. However, four studies examined computer use and found it was positively correlated with years of education. - 14 of 15 studies on TV viewing supported a positive relationship between unemployment/retirement and higher viewing. TV viewing and its relationship with income is inconclusive. - Four studies examined the work sector and its potential impact on sedentary behaviour. Discrepancy among relationship between manual and non-manual work and sitting time may be due to the difference between total sitting (i.e. leisure and work) and leisure-time sitting outcomes. - Five of nine studies found men reported more computer use than women. Reading behaviour found no association with gender. Gender may not affect sedentary behaviours with the exception of video games, where more men play than women. - Some evidence for a relationship between TV and general screen viewing and BMI although the relationship between BMI and other sedentary behaviours does not appear strong. - Inconclusive for ethnicity as a correlate of any sedentary behaviour. - The presence of children is associated with less sedentary behaviour.   **Qualitative** Literature is limited but sedentary behaviours appear to be related to positive attitudes. All three studies that measured TV viewing supported an association with an attitude construct (preference, utility, enjoyment). Three of three samples also found support for a relationship between attitudes and computer use. |
| **Notes by review team** |
| **Evidence gaps and/or recommendations for future research noted by study author:** 1) Limited research has been conducted on the cognitive, social, or environmental categories. Need socioecologic models, with an emphasis on environmental and cognitive correlates, to better understand sedentary behaviour.  2) Occupational variables such as employment type and physical activity on the job are relatively under-researched at present.  3) Improved methodological characteristics (measurement, design); standardisation of the outcome measures; and the reporting of effect sizes **Source of funding:** No fınancial disclosures were reported by the authors of this paper. **Limitations identified by author:** 1) Studies employed self-reported estimates of sedentary behaviour and these have unknown validity. 2) Publication bias 3) Limited to English-language 4) Selection bias via strategy **Limitations identified by reviewer:** 1) Most of evidence base is cross-sectional (76%, n=83) 2) Large variation in correlates included in analysis of studies |

## B.3 Diet

| **Authors:** Lachat C, Nago E, Verstraeten R, Roberfroid D, Van Camp J, Kolsteren P  **Year:** 2012  **Citation:** Obesity Reviews 13(4): 329-346  **Country of study:** International  **Aim of study:** Systematic review of the association between eating out of home and dietary intake  **Study design:** Systematic review (of observational longitudinal (n=4) and cross-sectional (n= 25) studies)  **Quality score: (++, + or -):** - |
| --- |
| **Population and setting** |
| **Source population/s:** International (USA, UK, Europe, Australia, China, Kenya, Russia, Philippines).  **Setting:** Eating out of home defined as the place of consumption or preparation of food. **Sample characteristics:** Qualitative studies excluded studies in institutions hospitals, day care excluded. **Attrition details**: Not reported for individual included studies |
| **Study design** |
| **Exposure/s description:** All studies that used a quantified dietary assessment method to estimate the dietary contribution of foods and drinks consumed out of the home were included, so this incorporated solid food as well as alcoholic and non-alcoholic drinks. **Physical environment:** Not reported  **Socio-cultural environment:** Not reported **Economic environment:** Not reported **Political environment:** Not reported |
| **Outcomes and methods of analysis** |
| **Methods of analysis:** Calculated an adjusted R2 for each study correcting for the sample size and the number of predictors entered in the final regression model. A random-effect R2 was calculated for the prediction of behaviour and intention in relation with FVI, FI only and VI only. Assessed between-study heterogeneity using two common statistical approaches: a chi-squared test (Cochran’s Q) and the I2 compared the impact of a number of a priori defined potential moderators by comparing random-effect R2 for different categories of moderators using Fisher’s Z transformation procedures for correlations. **Outcomes:** **Follow-up periods:** 18 studies cross-sectional design and five studies used a longitudinal design. |
| **Results** |
| - 29 studies met inclusion criteria. - Foods eaten out of home were important sources of energy in all age groups. - Eating out of home was associated with higher total energy intake, higher energy contribution from fat and higher SES. - Eating out of home was also associated with lower micronutrient intake. |
| **Notes by review team** |
| **Evidence gaps and/or recommendations for future research noted by study author:  Source of funding:  Limitations identified by author:  Limitations identified by reviewer:** Included studies predominantly cross-sectional. Contains studies in children. |

| **Authors:** Guillaumie L, Godin G, Vezina-Im LA  **Year:** 2010  **Citation:** International Journal of Behavioral Nutrition and Physical Activity 7(12)  **Country of study:** International  **Aim of study:** Review social cognitive theory-based studies of fruit and vegetable intake and to identify its main psychosocial determinants  **Study design:** Systematic review  **Quality score: (++, + or -):** - |
| --- |
| **Population and setting** |
| **Source population/s:** United States (6), Netherlands (3) and Great-Britain (3)  **Setting:** International **Sample characteristics:** A total of 23 studies were included, involving 34,577 participants.  **Attrition details:** 18 studies cross-sectional design. Five studies used a longitudinal design. The time interval between baseline measurement of psychosocial variables and behaviour assessment ranged between one and five weeks. |
| **Study design** |
| **Exposure/s description:  Physical environment:** Not reported **Socio-cultural environment:** Not reported **Economic environment:** Not reported **Political environment:** Not reported  **Potential confounders:** Not reported **Inclusion**: Included studies that assessed the predictive value of social cognitive theories using the R2 statistic for FVI in the general adult population. Studies among elders (>65 years of age), children (<18 years of age), students or seriously ill population were excluded. |
| **Outcomes and methods of analysis** |
| **Methods of analysis:** Calculated an adjusted R2 for each study correcting for the sample size and the number of predictors entered in the final regression model. A random-effect R2 was calculated for the prediction of behaviour and intention in relation with FVI, FI only and VI only. Assessed between-study heterogeneity using two common statistical approaches: a chi-squared test (Cochran’s Q) and the I2 compared the impact of a number of a priori defined potential moderators by comparing random-effect R2 for different categories of moderators using Fisher’s Z transformation procedures for correlations. **Outcomes: Follow-up periods:** 18 studies cross-sectional design and five studies used a longitudinal design. |
| **Results** |
| - Quantitative. - Seven studies demonstrated a low level of correspondence between predictors and behaviour. - Variables most consistently associated with the prediction of FVI (at least 50% of time) were habit, motivation and goals, beliefs about capabilities and knowledge. Same variables were also most consistently associated for FI and VI. For VI, however, there was an additional association with taste. - Behavioural regulation was assessed only once and was found significant in the FI, VI and FVI predictions. - With respect to the factors explaining intention regarding FVI, the most consistently significant cognitive variables were beliefs about capabilities, beliefs about consequences and social influences. The same variables were also most consistently associated for FI and VI intention. |
| **Notes by review team** |
| **Evidence gaps and/or recommendations for future research noted by study author:** Compare the efficacy of different theories to predict FVI. **Source of funding:** Not reported **Limitations identified by author:** Small number of studies publication bias **Limitations identified by reviewer:** Both longitudinal and cross-sectional studies. 18 studies cross-sectional. |

| **Authors:** Fleischhacker SE, Evenson KR, Rodriguez DA, Ammerman AS  **Year:** 2011  **Citation:** Obesity Reviews 12(5): e460-71  **Country of study:** International  **Aim of study:** Examine the methodology and current evidence on fast food access and its associations with outcomes  **Study design:** Systematic review  **Quality score: (++, + or -):** - |
| --- |
| **Population and setting** |
| **Source population/s:**  USA (n = 25) Urban (n = 15) Urban and rural (n = 8) Did not indicate the settings of the hospitals (n = 2) Adult (n = 6) Australia (n = 5)  Urban (n = 4) (14,33,40,51) Rural (n = 1) (26) Adults (n = 1) (26) Canada (n = 5)  Urban (n = 3)  Urban and rural (n = 1)  Did not indicate the setting of the hospital (n = 1) UK (n = 4) Urban (n = 2) Did not indicate the urbanity/rural area of the settings (n = 2)  New Zealand (n = 2)  **Setting:** International **Sample characteristics:** One study was longitudinal while the remaining 39 were cross-sectional. **Attrition details:** Not reported |
| **Study design** |
| **Exposure/s description:  Physical environment:** Density count proximity ratio **Socio-cultural environment:** Ethnicity **Economic environment:** SES **Political environment:** Not reported  **Potential confounders:** SES **Inclusion:** Study included only research articles examining fast food access with data collection and analysis. Studies examining fast food restaurants on school campuses rather than near school campuses were excluded, as the school lunch policy implications for competitive foods differ significantly from the environmental and policy implications of off-school campus venues. |
| **Outcomes and methods of analysis** |
| **Methods of analysis:** Narrative synthesis **Outcomes:** Dietary intake, physical activity, mortality admissions for acute coronary syndromes **Follow-up periods:** Not reported |
| **Results** |
| - Quantitative. - The majority (n = 16, 76%) indicated fast food restaurants were more prevalent in low-income areas compared with middle- to higher-income areas. - 10 of 12 studies found fast food restaurants were more prevalent in areas with higher concentrations of ethnic minority groups in comparison with Caucasians. - One paper reported a negative association between physical activity levels and access to fast food restaurants |
| **Notes by review team** |
| **Evidence gaps and/or recommendations for future research noted by study author:** 1) Explore how individual-level economic changes relate to individual changes in fast food access, fast food consumption and health outcomes 2) How downturns in the economy impact fast food restaurants region’s SES and race/ethnicity interact with the region’s fast food restaurant supply and demand 3) If and how fast food access impacts dietary intakes and health outcomes 4) If fast food access has disparate socio-economic, race/ethnicity and age associations **Source of funding:** A National Institute of Health (NIH) University of North Carolina Interdisciplinary Obesity Training Grant (T 32 MH75854) supported this project. **Limitations identified by author:** 1) Inconsistent methods to characterise SES, the racial composition of neighbourhoods, lack of community input on ethnic/race and neighbourhood definitions and limited information on under-studied ethnic groups 2) Lack of consensus on the definition of fast food. **Limitations identified by reviewer:** 39 cross-sectional and only seven studies focused on adults (n = 7) |

| **Authors:** De Irala-Estevez J, Groth M, Johansson L, Oltersdorf U, Prattala R, Martinez-Gonzalez M  **Year:** 2000  **Citation:** European Journal of Clinical Nutrition 54(9): 706-14  **Country of study:** International  **Aim of study:** Evaluate the differences in the consumption of fruit and vegetables between groups with different socio-economic status (SES) in the adult population of European countries  **Study design:** Systematic review  **Quality score: (++, + or -):** - |
| --- |
| **Population and setting** |
| **Source population/s**: Belgium, Denmark, Estonia, Finland, Germany, Greece, Lithuania, Norway, Spain, Sweden and UK  **Setting:** Europe **Sample characteristics:** The number of subjects included in each individual study ranged from 704 to 41,178. Age was not available individually in these studies (range 18-85). **Attrition details:** Response rate was between 55% and 95%. |
| **Study design** |
| **Exposure/s description:  Physical environment:** Not reported **Socio-cultural environment:** Education level **Economic environment:** Occupational level **Political environment:** Not reported  **Potential confounders:** Country, gender, year of the study and method of dietary assessment **Inclusion:** The inclusion criteria of studies were:   1. Use of a validated method for assessing intake at the individual level; 2. Selection of a nationwide sample or a representative sample of a region; 3. Providing the mean and standard deviation of overall fruit and vegetable consumption for each level of education or occupation, and separately for men and women. |
| **Outcomes and methods of analysis** |
| **Methods of analysis:** Meta-analysis, random-effects model **Outcomes:** Consumption of fruit and vegetables **Follow-up periods:** Not reported |
| **Results** |
| - Quantitative. - Found a positive association between a higher level of education or occupation and a greater consumption of both fruit and vegetables. - A higher SES was associated with a greater consumption of both fruit and vegetables. The pooled estimate of the difference in the intake of fruit was 24.3g/person/day (95% CI 14.0-34.7) between men in the highest level of education and those in the lowest level of education. Similarly, this difference was 33.6g/person/day for women (95% CI 22.5-44.8). The differences regarding vegetables were 17.0g/person/day (95% CI 8.6-25.5) for men and 13.4g/person/day (95% CI 7.1-19.7) for women. |
| **Notes by review team** |
| **Evidence gaps and/or recommendations for future research noted by study author:  Source of funding:** The present study was supported by the European Union's FAIR programme (FAIR-97-3096). **Limitations identified by author:** 1) Only a few studies adjusted for total energy intake 2) Over-reporting consumption among those with higher levels of education. 3) Heterogeneity in methods across pooled studies 4) Response rate. Differences between educational/ occupational levels were lower as the response rate rose. **Limitations identified by reviewer:** Socio-economic position measured only as education and occupational level. |

| **Authors:** Power EM  **Year:** 2005  **Citation:** Canadian Journal of Public Health-Revue 96:S37-S42  **Country of study:** International  **Aim of study:** To discover the determinants of healthy eating among low-income Canadians  **Study design:** Systematic review  **Quality score: (++, + or -):** - |
| --- |
| **Population and setting** |
| **Source population/s:** Not reported  **Setting:** Not reported **Sample characteristics:** Not reported **Attrition details:** Not reported |
| **Study design** |
| **Exposure/s description:  Physical environment:** Not reported **Socio-cultural environment:** Education. Nutritional knowledge. Food skills. **Economic environment:** Income threshold (likelihood that, beneath the threshold, income is the most important determinant of consumption). **Political environment:** Not reported **Potential confounders:** Social inequalities, poverty, smoking.  **Inclusion:** The minimum methodological criteria for inclusion were as follows: 1. A clear statement of methods, including study population and selection of sample; identification of data collection methods; a discussion of data collection biases; 2. Elaboration of the details of data analysis; appropriate statistical tests or analytical approach used; 3. Interpretation of the findings that was appropriate for the data collected and the analytical framework. |
| **Outcomes and methods of analysis** |
| **Methods of analysis:** Narrative synthesis **Outcomes:** Healthy eating **Follow-up periods:** Not reported |
| **Results** |
| - Quantitative studies that have measured nutrient intake, rather than food consumption, have found the differences among socio-economic groups to be small. - Income is the most important determinant of food insecurity and hunger, but there is not a linear relation between income and measures of food security. - Higher levels of education do not protect households from food insecurity, nor does education appear to mitigate the dietary effects of inadequate income. Neither nutritional knowledge nor food skills appear to be significant factors affecting healthy eating in these populations. |
| **Notes by review team** |
| **Evidence gaps and/or recommendations for future research noted by study author:** 1) Longitudinal study design could provide data on how changes in cultural capital, income and food security status, as well as in factors such as age, family composition and children’s ages, affect food practices. 2) Little research on the interaction of income with other factors affecting food practices, such as housing status, social support, family roles and responsibilities, time constraints, the stage of the life course, ethnicity. 3) Important to explore how the food industry shapes social norms around eating. 4) Understand how social marketing campaigns to promote healthier diets can be more effective. **Source of funding:** Not reported **Limitations identified by author:** Not reported **Limitations identified by reviewer:** |

| **Authors:** Kamphuis CB, Giskes K, de Bruijn GJ, Wendel-Vos W, Brug J, van Lenthe FJ  **Year:** 2006  **Citation:** The British Journal of Nutrition 96(4): 620-35  **Country of study:** UK, Europe and Australia  **Aim of study:** Summarise the existing empirical evidence pertaining to environmental influences on fruit and vegetable consumption  **Study design:** Systematic review  **Quality score: (++, + or -):** + |
| --- |
| **Population and setting** |
| **Source population/s**: Studies were conducted in the UK (n=8), USA (n=7), Europe (n=7; e.g. Norway, Spain) and Australia (n=2). **Setting:** International **Sample characteristics:** Sample sizes ranged from 63 to 142,715. No other details reported **Attrition details:** The lowest response rate was 23% and the highest 95.2%. A number of studies did not provide RR details. |
| **Study design** |
| **Exposure/s description:**  **Physical environment:** 1. Accessibility and availability, including physical and financial accessibility of products and shops that are needed for an (un)healthy diet;  2. Social conditions, including social relationships (e.g. family/marital status), social support and psychosocial stress;  3. Cultural conditions, including culture-specific eating patterns, health value orientations, food experiences in childhood and cultural participation;  4. Material conditions, including financial situation, material and social deprivation, and unfavourable working, housing and neighbourhood conditions **Socio-cultural environment:** Defined as physical  **Economic environment:** Defined as physical  **Political environment:** Defined as physical  **Potential confounders:** **Inclusion**: Studies included were: 1. Observational studies published in English between 1 January 1980 and 31 December 2004;  2. Studies conducted among a population-based sample of adults (i.e. no patient groups) aged 18–60 years;  3. Dependent variable of intakes of energy, fat, fruits, vegetables, or fruits and vegetables combined as one outcome measure;  4. Independent variable: variables that could be classified as an ‘environmental’ factor according to the definition of Sallies & Owen (2002), i.e. ‘all factors external to the individual’;  5. Studies being conducted in an ‘established market economy’ as defined by the World Bank (2005). Intervention studies were excluded. |
| **Outcomes and methods of analysis** |
| **Methods of analysis:** Narrative synthesis **Outcomes:** Total energy, total fat, saturated fat, FV intakes **Follow-up periods:** All studies had a cross-sectional design |
| **Results** |
| **Quantitative**   - Most evidence was found for household income, as people with lower household incomes consistently had a lower FV consumption. People living in households with a higher income had greater fruit consumption. Association was found among people living in a neighbourhood with a higher median income, even after adjustment for individual socio-economic status. - Married people had higher intakes than those who were single, whereas having children showed mixed results. - Men and women who reported eating home-grown produce had a significantly higher FV consumption than those who did not. Good local availability (e.g. access to one’s own vegetable garden, having low food insecurity) seemed to exert a positive influence on intake. Having a vegetable garden was positively and significantly associated with fruit consumption. - Household income demonstrated a consistent and significantly positive association with vegetable intake in seven associations. People living in higher-income neighbourhoods generally had higher energy-adjusted intakes of vegetables.   **Qualitative**   - Considerable disparities between European countries in terms of the availability of fruit at the national level were found, which are probably an explanation for the diverse percentage of low fruit consumers |
| **Notes by review team** |
| **Evidence gaps and/or recommendations for future research noted by study author:** More longitudinal research on supportive food environments as relevant environmental factors may differ for various outcomes **Source of funding:** The project was supported by a grant from the Netherlands Organisation for Health Research and Development. K. G. is supported by an Australian National Health and Medical Research Council Sidney Sax Fellowship (ID 290540). **Limitations identified by author:** 1) Small population sizes 2) Poor reporting of samples 3) Non-longitudinal designs  **Limitations identified by reviewer:** 1) No formal attempt to gauge study quality 2) All studies had a cross-sectional design |

| **Authors:** Bisogni CA, Jastran M, Seligson M, Thompson A  **Year:** 2012  **Citation:** Journal of Nutrition Education and Behavior 44(4): 282-301  **Country of study:** International, developed countries (i.e. United States, European countries, Australia, New Zealand, Canada, and Japan)  **Aim of study:** To identify how qualitative research has contributed to understanding the ways people in developed countries interpret healthy eating  **Study design:** Systematic review of qualitative, empirical studies published in English , peer-reviewed journals (bibliographic database searches since 1995)  **Quality score: (++, + or -): ­**- |
| --- |
| **Population and setting** |
| **Source population/s**: From many different countries, not specified **Setting:** Both the individual (Behaviours; identity; knowledge and skills) and environments: social factors, resources (money and time involved in healthy eating) and competing priorities (conflicts people perceive between health and other considerations in food behaviours) **Sample characteristics:** Adolescents; adults; men; individuals living alone; couples; cancer survivors  **Attrition details:** |
| **Study design** |
| **Exposure/s description:**  **Physical environment:** Identity: A person’s identity or self-concept is often involved in the ways that they eat, and typically people seek identities that they see as positive and providing self-esteem; resisting their health care providers’ recommendations for eating because they wished to retain certain identities and avoid being stigmatised by their social group; strong ideals related to personal choices and responsibilities led them to resist government advice  **Socio-cultural environment:** Eating is a social activity for most people; social support in healthy eating; Applying family systems theory to their analysis, these researchers identified three different ways that couples initially adapted to the recommended diet ­- cohesive (teamwork), enmeshed (diabetic spouse dependent on non-diabetic spouse), and disengaged (diabetic spouse solely responsible for diet). **Economic environment**: Resources: people’s perspectives on how money, time, knowledge, and skills are involved in healthy eating; The changing and conflicting advice about healthy eating from experts and the media was a reason some study participants gave for not following current dietary recommendations **Political environment: Potential confounders: Inclusion:** |
| **Outcomes and methods of analysis** |
| **Methods of analysis:** Using an iterative process, the authors identified the following three main themes (see the outcomes)  **Outcomes:** 1) Meanings people associate with healthy eating;  2) Ways meanings develop and change in relation to life stage and life experiences;  3) Explanations people provide for the gaps between healthy eating ideals and their actual behaviours. **Follow-up period:** |
| **Results** |
| - Types of meanings associated with healthy eating (fruit and vegetables, animal food, safe food, functional food, general nutrients, vitamins and minerals, fat, carbohydrates, contaminants/toxins, natural, organic, homemade, balance, variety, moderation and regular meals and more....) - Life stages and life events and experiences related to the meanings for healthy eating (childhood; adolescence; adults and ageing; marriage/cohabiting; parenting; disease onset; women's transitions) - Types of explanations for the gap between healthy eating ideals and behaviours (Identity; Social factors; Resources; Food availability) - Qualitative |
| **Notes by review team** |
| **Evidence gaps and/or recommendations for future research noted by study author:** A particularly interesting and potentially important insight from this review relates to people’s holistic views about healthy eating that embrace psychosocial, physical well-being, and spiritual well-being. The moral aspects of healthy eating are also of interest to health professionals, and the theme of rejecting scientific advice is a particular concern. Although findings related to these themes seemed more common in recent papers, it is impossible to know whether people’s views are changing or whether researchers are uncovering or reporting themes that have always existed. Future studies should address these themes and how their specific interpretations are associated with social class, culture, personal factors, life course experiences, and/or world views. **Source of funding: Limitations from author:** **Limitations from reviewer:** Implications: People interpret healthy eating in complex and diverse ways that reflect their personal, social, and cultural experiences, as well as their environments. Their meanings include but are broader than the food composition and health outcomes considered by scientists. The rich descriptions and concepts generated by qualitative research can help practitioners and researchers think beyond their own experiences and are open to audience members’ perspectives as they seek to promote healthy ways of eating. |

## B.4 Smoking

| **Authors:** Vangeli E, Stapleton J, Smit ES, Borland R, West R  **Year:** 2011  **Citation:** Addiction 106(12): 2110-21  **Country of study:** International  **Aim of study:** To identify the predictors of attempts to stop smoking and the predictors of quit attempt success in adult general population samples.  **Study design:** Systematic review  **Quality score: (++, + or -):** - |
| --- |
| **Population and setting** |
| **Source population/s:** Australia, UK, Japan, USA, China, Thailand, Malaysia, Canada, France and Spain  **Setting:** Not reported **Sample characteristics:** Gender range from 30.3% male to 95% male. Age mean 42.7 (14.4 SD) to 44 years (15.6 SD). Sample sizes ranged from 267 to 16,458. **Attrition details:** Lowest rate 83.5%, highest 20% |
| **Study design** |
| **Exposure/s description:  Physical environment:** Not reported **Socio-cultural environment:**  Demographic and physical variables Gender Marital status/living with partner Have children living at home Age  Majority/minority group  Education Smoking allowed at work Home smoking ban  Current smoking Cigarette dependence  Cigarettes per day  Smoking and quitting history  Age when started smoking  Past attempts to quit Longest time off smoking Desire to quit Motivation to quit  Intention to quit  Evaluations of smoking and quitting Opinion of smoking  Health benefit outcome expectancy from quitting in the next 6 months Worries about the effect of smoking on health and QoL Enjoy smoking too much to give it up  Confidence of success in quitting **Economic environment:** Income (trend across increasing income category)  Employed  Social class (scale of increasing affluence)  **Political environment:** Not reported **Potential confounders:** No two studies include the same set of covariates. A total of 86 predictor variables were examined, 26 of which were examined in two or more studies. For review, these were grouped into six categories of conceptually similar measures: demographics and physical characteristics, current smoking, quitting history, desire to quit, evaluations of smoking and quitting and confidence in quitting. **Inclusion:** Non-intervention prospective studies written in English specifically examining predictors of cessation attempts and/or predictors of the success of attempts in adult (i.e. 16 years of age) general population samples were included in this review. |
| **Outcomes and methods of analysis** |
| **Methods of analysis:** The analysis of predictors of quit attempt success included only smokers known to have made a quit attempt. **Outcomes:** A quit attempt was defined as follows: participants were smokers at baseline and at a follow-up were recorded as having made an attempt to stop smoking between baseline and follow-up. Quit attempt success was defined as follows: participants were smokers at baseline and at a follow-up reported having stopped smoking and remained stopped at the time of the follow-up. **Follow-up periods:** Not reported |
| **Results** |
| - Quantitative - None of the socio-demographic variables were found to be predictive of making a quit attempt or quit attempt success. There was some evidence that higher social grade is predictive of quit attempt success, but this was examined in only two studies. - Other indicators of affluence (i.e. income, education, employment status) were not found to be predictive of either making a quit attempt or quit attempt success in most studies. |
| **Notes by review team** |
| **Evidence gaps and/or recommendations for future research noted by study author:** It is important to study the determinants of quit attempts separate to predictors of success. **Source of funding:** Cancer Research UK (grant no. C1417/A7972). **Limitations identified by author:** 1) Methodological heterogeneity 2) Univariate analysis (logistic regression)  **Limitations identified by reviewer:** No two studies include the same set of covariates. |

| **Authors:** Kakde S, Bhopal RS, Jones CM  **Year:** 2012  **Citation:** Public Health 126(8): 635-45  **Country of study:** International  **Aim of study:** A systematic review on the social context of smokeless tobacco use in the South Asian population: Implications for public health  **Study design:** Systematic review  **Quality score: (++, + or -):** - |
| --- |
| **Population and setting** |
| **Source population/s:** South Asian populations. The populations studied were Bangladeshi, Indian, Nepalese and Pakistani.  **Setting:** International. Irrespective of their current geographic location but in free-living settings (i.e. excluding institutions). **Sample characteristics:** Both smokeless tobacco users and non-users. Sample size varied in each study from 45 to 1590. Most studies encompassed both sexes; however, two studies in the UK only included women. The populations studied had a wide age range of 8-96 years. **Attrition details:** 11 studies reported good response rates of over 60%. |
| **Study design** |
| **Exposure/s description:  Physical environment:** Not reported **Socio-cultural environment:** Studies that compared the correlation of SLT users with non-users stated that social and contextual factors act as predictors of use among adolescents. Wide cultural acceptability of SLT use. In the UK, reasons for use varied widely; the main reasons were addiction (34.0-84.0%), taste (22.0-75.8%) and the perceived improvement of dental health (18.0-29.3%). **Economic environment:** Studies excluded if focused on socio-economic status and education levels of SLT users, not on attitudes, beliefs and perceptions **Political environment:** None found  **Potential confounders:** Not reported **Inclusion:** Studies included covered:  1. Attitudes and/or beliefs and/or perceptions towards SLT use (snuff/snus was not included); 2. Studies from any discipline or theoretical tradition that uses qualitative methods, quantitative methods and mixed methods; 3. Published and unpublished studies found by searches. |
| **Outcomes and methods of analysis** |
| **Methods of analysis:** Data extraction forms using tables for quantitative studies and textual summaries for qualitative studies were employed. As meta-analysis was not warranted, non-statistical analysis of quantitative studies and thematic synthesis of qualitative studies was integrated. Comparisons between studies were difficult given different methods and populations, and hence variations between studies are open to interpretation as no synthesis of results was possible due to the heterogeneity. **Outcomes:**  - Age at and reasons for commencement of SLT use - Current reasons for SLT use - Perceived knowledge of harmful effects - Source of information - Facilitators and barriers to SLT use **Follow-up periods:** None. Included studies were cross-sectional**.** |
| **Results** |
| **Quantitative**   - Reasons for starting included peer pressure, cultural and social acceptance, low cost and easy availability, medicinal use (both general and oral health), physical and mental relaxation, aid to concentration, and marketing strategies involving role models. - Studies conducted in India reported that amongst families where SLT use was a taboo, youngsters acquired the habit while living away from home. - Pregnant women were reported to start SLT use to ‘change the taste in their mouth’; however, they continued to use SLT postpartum due to addiction. - Reports SLT cessation attempts from five studies; however, none reported the duration of abstinence. Most users were unsuccessful in giving up the habit, a range of 33.3-62.5% thought of quitting. - The key requirements for cessation were social, physical and emotional support; these essential factors were primarily provided by parents, close family and friends (63.8%). In addition, media (53%) and advice from doctors/dentists (39%) also played a significant role in decision making. - In India, the main reasons for non-use were fear of cancer (20.1% and 59.1%), poor oral hygiene (39.7%), addiction (14.5%), parental disapproval (8.9%) and loss of social status (percentage not reported).   **Qualitative**   - Addiction was the main cause for the struggle to achieve cessation. Lack of information, resources, motivation and misconceptions associated with SLT use were also influential. - Users in India were positively influenced by physicians’ advice; however, this advice was devalued when doctors were users themselves. Studies also reported that peer pressure and isolation were the main reasons for resuming the habit, as abstinence restricted their social life with friends who were users. |
| **Notes by review team** |
| **Evidence gaps and/or recommendations for future research noted by study author:** The limitations of this review were mainly due to the limited studies (n=17), spanning 15 years. **Source of funding:** Partly funded by the University of Edinburgh Post-Graduate Research Fund. **Limitations identified by author:** The UK South Asian population probably differs from that of the Indian subcontinent, and care should be taken while extrapolating results from one population to another. **Limitations identified by reviewer:** 14 of the included studies were cross-sectional and employed questionnaires, two were qualitative and used interviews and focus groups, and one was mixed. |

| **Authors:** Niederdeppe J, Kuang X, Crock B, Skelton A  **Year:** 2008  **Citation:** Social Science & Medicine 67(9): 1343-55  **Country of study:** International  **Aim of study:** To identify promising media campaign strategies to increase smoking cessation and reduce tobacco-related disparities among socio-economically disadvantaged populations  **Study design:** Systematic Review  **Quality score: (++, + or -):** - |
| --- |
| **Population and setting** |
| **Source population/s:** USA and countries with comparable political systems and demographic profiles such as Canada, Australia and Western European nations  **Setting:** International **Sample characteristics:** Not clearly reported. Contains mixed sample of young and older adults, with some in college and school. Low SES but no ethnicity or gender details presented. **Attrition details:** Not reported |
| **Study design** |
| **Exposure/s description:  Physical environment:**  Health care access  Municipal smoking bans **Socio-cultural environment:** Education Community social capital **Economic environment:**  Income Occupation Workplace policies **Political environment:** Not reported  **Potential confounders:** Not reported **Inclusion:** Studies included either:  (1) Explicitly compared the effectiveness of general population media campaigns between lower and higher SES populations  (2) Assessed the overall effectiveness of media campaigns targeted specifically to low SES populations |
| **Outcomes and methods of analysis** |
| **Methods of analysis:** Development of a logic framework, to recognise that the causal chain of events linking media campaigns to sustained smoking cessation. Qualitative analysis due to wide variation between studies in measurement of outcomes. **Outcomes:** Message recall Motivational response Long-term abstinence from smoking **Follow-up periods:** Between 12 months and seven years |
| **Results** |
| - Quantitative - Reduced effectiveness among lower versus higher SES populations was observed at each stage of the hypothesized causal chain, including message recall, motivational response and long-term smoking abstinence |
| **Notes by review team** |
| **Evidence gaps and/or recommendations for future research noted by study author:** Insufficient evidence with which to draw stronger conclusions about the most promising messages (why to quit vs. how to quit) or executional styles (evocative testimonials vs. less emotional portrayals) to promote smoking cessation among low SES populations **Source of funding:** David Gundersen, the Wisconsin Tobacco Prevention and Control Program, and the Robert Wood Johnson Foundation Health and Society Scholars Program **Limitations identified by author:** 1) Focused search strategy on identifying low SES smokers. 2) Variety of operational definitions to identify low SES smokers **Limitations identified by reviewer:** Few covariates in the analysis |

| **Authors:** Bader P, Travis HE, Skinner HA  **Year:** 2007  **Citation:** American Journal of Public Health 97(8): 1434-43  **Country of study:** International/Canada  **Aim of study:** To synthesise evidence regarding effective strategies for smoking cessation among employed or unemployed young adults aged 18 to 24 years  **Study design:** Knowledge synthesis using three complementary approaches - systematic review, Delphi panel of experts, focus groups  **Quality score: (++, + or -):** - |
| --- |
| **Population and setting** |
| **Source population/s:** Young adults **Setting:** Canada  **Sample characteristics:** Not reported **Attrition details:** Not reported |
| **Study design** |
| **Exposure/s description: Physical environment:** Access to cigarettes, price of cigarettes, smoke-free indoor air restrictions, mass media (cigarette ads and sales bans). **Socio-cultural environment:** age of onset, intention to quit, nicotine addiction, association with drinking behaviour, friends who smoke, non-smoking parents, non-smoking partner, having children, living with children, education level, grades, perceived health status (physical and mental), attitudes regarding harmful effects of smoking, misperceptions regarding health risks, involved in physical activities (sports, exercise), attends bars or clubs, assuming adult social roles, able to resist peer pressure and other pro-smoking influences, psychological characteristics, adolescent rebelliousness and problem behaviour. **Economic environment:** Student, employed vs unemployed, employed: white-collar vs blue-collar and service workers **Political environment: Potential confounders:** **Inclusion:** |
| **Outcomes and methods of analysis** |
| **Methods of analysis:** Synthesis of knowledge from literature, experts & young adults. **Outcomes:** Smoking cessation **Follow-up period:** Not reported |
| **Results** |
| - Conflicting evidence in the literature regarding the effectiveness of smoking restrictions on young adult smoking behaviour. - Principal benefit of smoking is its significant social benefit - Contrasting views on perceived health risk - Participants held negative views toward traditional smoking cessation approaches - Important factors in selecting smoking cessation method include need of accurate information, cost, convenience, easy accessibility, location outside of hospitals or institutions, emphasis on benefits of quitting - Little is known about factors that affect smoking cessation among young adults; predictor variables with highest agreement among reviewed studies included extent of smoking among friends (eight studies), increased price of cigarettes (five studies), and intent to quit (four studies); inconsistent findings regarding education and employment - Adults smokers engage in risk minimisation, believe they are at less risk than others, undervalue health consequences of smoking, and do not fully understand short-term effects of smoking; strategies for smoking cessation need to be tailored to fit young adults’ health beliefs - Important factors in selecting a smoking cessation method include likelihood of its success; its cost, convenience, and flexibility; pain of quitting; low-demand interventions; social support; settings in naturally occurring social groups (such as community groups and fitness groups); participating in activities incompatible with smoking. |
| **Notes by review team** |
| **Evidence gaps and/or recommendations for future research noted by study author:** 1) Lack of intervention data on employed and unemployed young adults in the literature. 2) Few studies in the literature on information and communication technologies as a smoking cessation intervention for young adults **Source of funding:** This study was funded through the strategic initiative Advancing the Science to Reduce Tobacco Abuse and Nicotine Addiction, a partnership coordinated by the Canadian Tobacco Control Research Initiative. **Limitations from author:** Not reported  **Limitations from reviewer:** Young people, disadvantaged population - by midlife are we not dealing with addiction? |

## B.5 Alcohol

| **Authors:** Bryden A, Roberts B, Petticrew M, McKee M  **Year:** 2013  **Citation:** Health and Place 21: 70-85  **Country of study:** International  **Aim of study:** The associations between community level social factors and alcohol use  **Study design:** Systematic review  **Quality score: (++, + or -):** ++ |
| --- |
| **Population and setting** |
| **Source population/s:** Population of interest was adult and adolescent males and females.  North America (n = 14), United Kingdom (n = 3), Canada (n = 1), Bolivia (n=1) and Australia (n=1). **Setting:** Communities were defined as neighbourhoods, villages, towns or residential college campuses. Urban, rural and mixed. 33 studies were carried out in the United States, three in Canada, three in United Kingdom and nine in other countries. A range of community types were included in the studies, with 26 in urban communities, two in rural and 17 in mixed urban–rural communities. A further two studies were conducted on residential college campuses and one on an American Indian reservation. **Sample characteristics:** Sample sizes of single studies ranged from 206 to 52,780. **Attrition details:** Not reported |
| **Study design** |
| **Exposure/s description:  Physical environment:** Not reported  **Socio-cultural environment:** Disorder and crime, including social disorder (e.g. drug activity, divorce rate), physical disorder (e.g. graffiti), safety, crime and violence in the community , social capital (e.g. trust,  membership, support from neighbours), community norms about alcohol use **Economic environment:** Socio-economic deprivation(e.g. average income, unemployment rate) **Political environment:** Not reported  **Potential confounders:** Some studies did not adjust their results for any potential confounders. **Inclusion:** Studies which only explored individual level factors (e.g. individual level demographic or socio-economic characteristics), parental or peer characteristics (e.g. drinking norms among friends) or genetic characteristics (e.g. family history of harmful alcohol use) were excluded. |
| **Outcomes and methods of analysis** |
| **Methods of analysis:** A narrative synthesis is used to describe the studies and their results **Outcomes:** Quantity or frequency of alcohol consumption Binge drinking Alcohol dependency Problem drinking Prevalence of drinking among adolescents **Follow-up periods:** Not reported |
| **Results** |
| - Quantitative - 18 studies (20 papers with 36 effect estimates) examined the association between deprivation and alcohol use. They produced inconclusive results. - Among adults, six studies found no significant association between alcohol use and the level of deprivation in a community, all of which investigated heavy or problematic drinking. One study found that men were significantly more likely to experience alcoholism symptoms if they had lived in a more deprived community (b=0.77) - Two studies found no significant association between adult alcohol use and income. Three studies found that adult alcohol use and alcohol problems were significantly more likely in wealthier communities. There were mixed findings on adult alcohol use from the three studies that focused specifically on income inequality within communities. There is some indication that alcohol use may be higher in communities with higher unemployment levels. - Among adults, one study found no significant association between neighbourhood problems (including noise and antisocial behaviour) and regular heavy drinking in London, but this had a low response rate and gave no detailed results. - Ten studies (ten papers and 26 effect estimates) were found on the association between community attachment, closeness and supportiveness and alcohol use. - One study of adults (two papers) found mixed results on the association between social norms and alcohol use. After controlling for social network and individual norms, permissive drunkenness norms were associated with higher levels of binge drinking (OR=1.58) but not with moderate drinking (OR=1.14), and no associations were found between drinking and communities having permissive drinking norms. |
| **Notes by review team** |
| **Evidence gaps and/or recommendations for future research noted by study author:** More longitudinal data are required that follow people and communities over time to better estimate temporal associations between alcohol consumption and community level social factors. **Source of funding:** No external funding was used to conduct this review. **Limitations identified by author:** 1) 36 were cross-sectional, ten were longitudinal and two were before-after intervention studies. 24 studied adults, 26 adolescents and two students, with some including both adults and adolescents. The variety of exposure and outcome measures examined in the studies also means that it is very difficult to estimate the size of the overall effect that these community level factors may have on alcohol use. All of the studies used self-reported alcohol use data, which may have implications for the validity of the outcome measures **Limitations identified by reviewer:** Most of the evidence was from cross-sectional studies. |

| **Authors:** Bryden A, Roberts B, McKee M, Petticrew M  **Year:** 2012  **Citation:** Health & Place 18(2): 349-57  **Country of study:** International; 26 quantitative studies conducted in high-income countries (18 in US, 4 in Australia, and remaining four in Canada, The Netherlands, New Zealand and Switzerland)  **Aim of study:** To explore evidence on the influence on alcohol use of community level availability and marketing of alcohol.  **Study design:** Systematic review of observational (cross-sectional and longitudinal) and intervention studies was conducted according to PRISMA systematic review guidelines (Liberati etal.,2009); 26 quantitative studies included  **Quality score: (++, + or -):** ++ |
| --- |
| **Population and setting** |
| **Source population/s: Setting Sample characteristics:** Men and women of all ages **Attrition details:** |
| **Study design** |
| **Exposure/s description:**  **Physical environment:** 1) Availability of alcohol: the density of off-premise outlets (e.g. shops) and on-premise outlets (e.g. bars), distance to nearest outlet, willingness of retailers to sell alcohol to minors (e.g. measured by successful purchase attempts), percentage of adolescents that have purchased alcohol from commercial outlets and local licensing policies (e.g. community-wide restrictions on hours, days and volumes of alcohol sales).  2) Marketing related to alcohol: local advertising of alcohol (e.g. billboards, in-store adverts) and the presence of local protective messages (e.g. alcohol awareness advertising on billboards or in outlets as measured in the primary study). **Socio-cultural environment: Economic environment:** |
| **Outcomes and methods of analysis** |
| **Methods of analysis:** A narrative synthesis is used to describe the studies and their results, rather than a meta-analysis. The effect sizes reported in the original studies are presented in Tables 1 and 2 (regression coefficients, correlation coefficients, odds ratios and risk ratios). When confidence intervals were not provided in the papers, these were calculated where data were available. If no p value is given for a specific result, these results were only stated as ‘significant’ or ‘not significant’ in the original paper. **Outcomes:** Alcohol use, including prevalence of drinking, quantity or frequency of alcohol consumed (any type of alcohol), and also the extent of harmful alcohol use, including alcohol dependency and problem drinking. **Follow-up period:** |
| **Results** |
| - Qualitative/Quantitative - Availability of alcohol - Outlet density - Distance to nearest outlet - Willingness to sell alcohol to minors - Local changes to licensing regulation - Advertising and media - Exterior advertising - Interior advertising - Health protection message |
| **Notes by review team** |
| **Evidence gaps and/or recommendations for future research noted by study author:** This is the first systematic review of evidence on the relationship between alcohol use and availability and marketing of alcohol at the community level. Although the current status of the evidence base should be taken into account, policy makers should be aware of the possible influence that community level availability and advertising of alcohol have on drinking and heavy drinking, and particularly the possibility that adolescents may be more likely to start drinking if they are exposed to alcohol adverts in their community. In the United Kingdom, the health groups that recently pulled out of the government’s responsibility deal on alcohol claimed that it does not go far enough to protect young people and has a poor evidence base - and the evidence in this systematic review supports the need to limit the exposure of young people to alcohol advertising (Hastings and Sheron, 2011). **Source of funding:**  **Limitations from author:** As this research area is dominated by studies from the U.S., particularly on alcohol advertising, more studies are needed from other locations in order to provide a better understanding of the associations and also to provide international comparisons. Future studies on alcohol use should also consider the health and policy implications of their findings, and part of this will be to consider the usefulness of some of the outcome measures used in the current evidence base.  **Limitations from reviewer:** |

| **Authors:** Brienza RS, Stein MD  **Year:** 2002  **Citation:** Journal of General Internal Medicine 17(5): 387-97  **Country of study:** International?  **Aim of study:** To describe how alcohol use disorders (AUDs) affect women, focusing on gender-specific implications for primary care physicians (PCPs)  **Study design:** Overview of literature from 1966-2000  **Quality score: (++, + or -): -** |
| --- |
| **Population and setting** |
| **Source population/s:** Women **Setting:** International  **Sample characteristics:** Gender-specific data from cohort studies of general population or large clinical samples are primarily reviewed. **Attrition details:** Not reported |
| **Study design** |
| **Exposure/s description:**  **Physical environment: Socio-cultural environment:** Stigma, roles and partners, fear of loss of children  **Economic environment:** Financial dependency, employment  **Political environment:** **Potential confounders:** Psychiatric comorbidities, age **Inclusion:** Not reported |
| **Outcomes and methods of analysis** |
| **Methods of analysis:** Narrative synthesis **Outcomes:** AUD **Follow-up period:** Not reported |
| **Results** |
| - Societal norms and expectations may make admission of AUD more problematic for women. - Although prevalence higher in men, women with AUDs are more likely to seek help, but less likely to be identified by their physicians. - Common barriers for women to treatment include: income or underinsurance; fear of abandonment by their husband or partner after help seeking; lack of child care during treatment; exclusion of pregnant women from treatment programs; lack of transportation secondary to overall lower levels of socio-economic status; and fear of loss of custody of children. - Barriers for men seeking treatment have been shown to be more closely related to loss of career and financial instability. - Women begin drinking later than men and often drink alone in the home. - Alcoholic women are more likely to be left by their partners, especially at the time of entry into treatment, than are alcoholic men. - Women are much more likely to attribute their drinking to a traumatic event or stressor and often view their drinking as self-medication. - While men are more likely to have their drinking affect their jobs and career paths, women are more likely to initially experience disruptions in relationships and family life. - Women in their 30s to 60s are most at risk when they are divorced or widowed, not employed, and have no children living at home - Alcohol leads to increased vulnerability and potential for violence, especially toward women alcoholics. - Homosexually active women reported using alcohol more frequently and in greater amounts and experienced greater alcohol-related morbidity than did exclusively heterosexually active women. |
| **Notes by review team** |
| **Evidence gaps and/or recommendations for future research noted by study author:** Important areas for further research include: genetic studies focusing on distinguishing patterns of AUD inheritance in men and women; research specifically aimed at identifying why women have increased vulnerability to alcohol in non-reproductive organ systems; and the potential for gender differences in the performance of screening instruments for AUDs. **Source of funding:** Not reported  **Limitations from author:** Not reported  **Limitations from reviewer:** Unclear inclusion/exclusion. Unclear selected and reporting. |

## B.6 Overweight

| **Authors:** Giskes K, Avendano M, Brug J, Kunst AE  **Year:** 2010  **Citation:** Obesity Reviews 11(6): 413-29  **Country of study:** International  **Aim of study:** Examine socio-economic inequalities in intakes of dietary factors associated with weight gain, overweight/obesity among adults in Europe  **Study design: Systematic review**  **Quality score: (++, + or -): -** |
| --- |
| **Population and setting** |
| **Source population/s:** Most were conducted in Scandinavian and Baltic countries, and the UK and Ireland. No studies from eastern European countries were located.  **Setting:** EU, Norway and Switzerland **Sample characteristics:** Two studies had a time-series design, all other studies had cross-sectional study designs. The majority of study sample sizes were large (>4000 participants) and ranged from 297 to 69,383 participants. **Attrition details:** Study response rates were not reported for approximately 20% of the studies, but the majority were greater than 55% (ranging from 39% to 87%) for the remaining studies. |
| **Study design** |
| **Exposure/s description:**  **Physical environment:** Not reported **Socio-cultural environment:** Not reported **Economic environment:**  Education Occupation Income Car ownership Housing tenure Area based indicators of SEP (e.g. deprivation characteristics of areas) **Political environment:** Not reported  **Potential confounders:** Age, ethnicity and household composition/size-when household income was used as SEP indicator. **Inclusion:** Only included studies:   1. Published in the peer-reviewed literature; 2. Published in English; 3. Conducted among a population-based sample; 4. Examined at least one of the in-scope dietary factors.   In-scope studies must have also assessed SEP using at least one measure. Studies that did not report intakes of all socio-economic groups were excluded from the current study |
| **Outcomes and methods of analysis** |
| **Methods of analysis:** Narrative synthesis **Outcomes:** Consumption of energy, fat, fibre, fruit, vegetables, energy-rich drinks and meal patterns **Follow-up periods:** Not reported |
| **Results** |
| **Quantitative**   - Socio-economically disadvantaged groups consume less fibre, fruit and vegetables than their more advantaged counterparts, and these dietary inequalities are consistent by gender and region - Approximately half the associations examined between SEP and fat intakes showed higher total fat intakes among socio-economically disadvantaged groups. There were no regional or gender differences in the direction and magnitude of the inequalities in the dietary factors examined. - Of the 33 associations tested, 12 found no differences, 13 found higher energy intakes among socio-economically disadvantaged groups and eight demonstrated lower intakes among these groups. - There were no systematic patterns in associations found by region, socio-economic indicator or gender for fat intake. - There was no regional, gender or SEP indicator variation in the direction and magnitude of associations found related to fibre intake. - Thirty-five of the 50 associations showed socio-economic inequalities in fruit consumption, with all associations demonstrating lower consumption. These differences were generally moderate-to-large in magnitude (i.e. relative difference >10% and odds ratios 0.40–0.70). Studies with larger sample sizes and response rates found more associations between SEP and fruit consumption than those that had small sample sizes and lower participation rates. - The 23 studies located examined 58 associations between vegetable consumption and SEP; 47 of these associations showed lower consumption of vegetables among socio-economically disadvantaged groups.   **Qualitative** |
| **Notes by review team** |
| **Evidence gaps and/or recommendations for future research noted by study author:** Examine the contributions of energy-rich drinks, takeaway/convenience foods and meal patterns to socio-economic inequalities in overweight **Source of funding:** Financial support from the Commission of the European Communities, SP5A-CT- 2006-044128 **Limitations identified by author:** Broad range of dietary factors associated with overweight **Limitations identified by reviewer:** Mostly cross-sectional |

| **Authors:** Giskes K, van Lenthe F, Avendano-Pabon M, Brug J  **Year:** 2011  **Citation:** Obesity Reviews 12(501): e95-e106  **Country of study:** International; 28 studies conducted in developed countries (16 in US, three in Europe, two in Japan and seven in Australia or New Zealand)  **Aim of study:** To examine whether physical, social, cultural and economical environmental factors are associated with obesogenic dietary behaviours and overweight/obesity among adults.  **Study design:** Systematic review of 27 cross-sectional studies and one natural experiment  **Quality score: (++, + or -):** - |
| --- |
| **Population and setting** |
| **Source population/s: Setting: Sample characteristics:** Adults **Attrition details:** |
| **Study design** |
| **Exposure/s description:**  **Physical environment:** Accessibility and availability including physical and financial accessibility of products and shops that are needed for an (un)healthy diet (e.g. access to shops, and availability of high fat foods and less healthy snacks)  **Socio-cultural environment:** Social condition. These arise from inter-personal interactions (e.g. marketing) and social support **Economic environment:** Material conditions. Including unfavourable working, housing and neighbourhood conditions (e.g. neighbourhood deprivation). Obesogenic dietary factors: Dietary factors influence overweight/obesity through the energy balance pathway; excess energy intake is arguably the most important dietary factor in relation to weight gain and the development of overweight/obesity. **Political environment: Potential confounders: Inclusion:** |
| **Outcomes and methods of analysis** |
| 6 |
| **Results** |
| - Weight status was consistently associated with the food environment; greater accessibility to supermarket or less access to takeaway outlets was associated with a lower BMI or prevalence of overweight/obesity. However, obesogenic dietary behaviours did not mirror these associations; mixed associations were found between the environment and obesogenic dietary behaviours. - Living in a socio-economically deprived area was the only environmental factor consistently associated with a number of obesogenic dietary behaviours. Associations between the environment and weight status are more consistent than that seen between the environment and dietary behaviours. The environment may play an important role in the development of overweight /obesity, however, the dietary mechanisms that contribute to this remain unclear and the physical activity environment may also play an important role in weight gain, overweight and obesity. |
| **Notes by review team** |
| **Evidence gaps and/or recommendations for future research noted by study author:** The current review suggested that accessibility to supermarkets/takeaway outlets or residing in a socio-economically deprived area are environmental factors that may contribute to overweight or obesity and/or obesogenic dietary behaviours. These factors need to be targeted in multilevel health promotion interventions and policies aimed at decreasing overweight/obesity.The role of other environmental factors, however, should not be discarded without further investigation, namely those whose associations with dietary behaviours/weight status were not examined or are not possible to infer from the limited number of studies. **Source of funding:** **Limitations from author:** Our search strategy only located studies that were published in peer-reviewed journals and referenced in electronic databases, excluding ‘grey’ literature. We tried to minimise any potential bias that may be induced from only examining peer-reviewed literature by also performing searches in smaller and more specialized databases (e.g. CSA Illumine). There was also great variation between studies included in the current review in terms of the conceptualization, measurement and summary of both the environmental factors and dietary behaviours which may have contributed to heterogeneous findings. Although strict inclusion criteria were used, environmental or dietary intake measures sometimes differed markedly between studies. Most of the studies included in this review were cross-sectional, making it difficult to ascertain causality between environmental factors and obesogenic dietary intakes. **Limitations from reviewer:** |

| **Authors:** Lovasi GS, Hutson MA, Guerra M, Neckerman KM  **Year:** 2009  **Citation:** Epidemiologic Reviews 31: 7-20  **Country of study:** US  **Aim of study:** Review of published literature to identify promising approaches for reducing obesity-related health disparities affecting persons of low SES, black race and Hispanic ethnicity.  **Study design:** Systematic review of US studies with direct relevance to: 1) poor or low-SES individuals; 2) African Americans or individuals reporting their race as black; and 3) individuals reporting their ethnicity as Hispanic or Latino. Published evidence supplemented with county-level population from 2000 Census and sprawl index data.  **Quality score: (++, + or -):** - |
| --- |
| **Population and setting** |
| **Source population/s:** Persons of low SES, black race, Hispanic ethnicity.  **Setting:** USA.  **Sample characteristics:** Not reported **Attrition details:** Not reported |
| **Study design** |
| **Exposure/s description:**  **Physical environment:** Any built environment (environment search terms related to: a) obesity, b) access to healthy foods, c) physical activity), food environment, urban form or sprawl, places to exercise, aesthetics or physical disorder, and traffic or crime safety.  **Socio-cultural environment: Economic environment: Political environment: Potential confounders:** Area poverty, race, ethnicity, area education, minority population, household income, socio-economic status  **Inclusion:** Books and unpublished reports not included |
| **Outcomes and methods of analysis** |
| **Methods of analysis:** Narrative synthesis **Outcomes:** PA, BMI **Follow-up period:** Not reported |
| **Results** |
| - Correlates of built environment: the strongest support for food stores (supermarkets instead of smaller grocery/convenience stores), places to exercise, and safety as potentially influential for disadvantaged groups. - Disadvantaged groups were living in worse environments with respect to food stores, places to exercise, aesthetic problems, and traffic or crime-related safety. - Proximity to a supermarket is associated with less overweight, obesity, and hypertension, whereas proximity to grocery or convenience stores was associated with more overweight, obesity, hypertension, and diabetes - Proximity to ethnic markets and supermarkets was associated with higher body mass index among women but not men - Food prices also been associated with weight change - The proximity of food stores, but not restaurants, appears to be correlated with dietary intake and weight for our target groups - Restaurants appear to be more concentrated in poor neighbourhoods but less common in neighbourhoods with a high proportion of black residents - There are also studies that find that area poverty predicts more fast food restaurants while predominately black race predicts less - ‘‘Walkable’’ neighbourhoods with these characteristics have been reported to support physical activity and a lower body mass index - Residence in a high-sprawl county may hinder physical activity and promote obesity - Lacking access to parks, pools etc. may discourage physical activity - Proximity to exercise facilities may not be sufficient to affect behaviour for all populations, especially if additional barriers such as cost, restricted operating hours, or poor maintenance are present. - Well-maintained sidewalks, trails, and exercise facilities may support physical activity behaviour for our target populations - Low-income groups perceived less access to indoor and outdoor places to exercise. enjoyable scenery was a particularly important determinant of physical activity among lower-income participants - American women ranked lacking a safe place to exercise as their number one barrier to physical activity |
| **Notes by review team** |
| **Evidence gaps and/or recommendations for future research noted by study author:** Understand the historical context in which the current patterns arose and the way built environment characteristics vary in their importance for shaping behaviour and health **Source of funding:** This work was supported by the Robert Wood Johnson Foundation Health and Society Scholars Program. **Limitations from author:** 1) Lack of agreement on methods for assessing built environment characteristics and their consequences.  2) Quality of environmental measurement may differ across studies in a non-random way. **Limitations from reviewer:** |

| **Authors:** Jansen C, Sauter S, Kowalski C  **Year:** 2007  **Citation:** GMS Psycho Social Medicine 9: Doc 07  **Country of study:** Germany  **Aim of study:** Determine whether differences in the use of prevention and health promotion services in Germany can be attributed to health inequality between different social status groups  **Study design:** Systematic review  **Quality score: (++, + or -):** - |
| --- |
| **Population and setting** |
| **Source population/s:** Germans  **Setting:** Germany **Sample characteristics:** Not reported **Attrition details:** Not reported |
| **Study design** |
| **Exposure/s description:  Physical environment:** Not reported **Socio-cultural environment:** Education, occupation, income and gender **Economic environment:** Not reported **Political environment:** Not reported  **Potential confounders:** Not reported **Inclusion:** Not reported |
| **Outcomes and methods of analysis** |
| **Methods of analysis:** Not reported **Outcomes:** Health inequality **Follow-up periods:** Not reported |
| **Results** |
| - Qualitative - 20 of the 23 reviewed studies provided relatively clear evidence of a significant association between higher social status and greater use of prevention and health promotion services. - Evidence of this association was provided for almost the whole of Germany |
| **Notes by review team** |
| **Evidence gaps and/or recommendations for future research noted by study author:** 1) Lack of studies on tertiary prevention, especially with regards to prevention and health promotion services use among men, as well as general studies on health promotion among men and women. 2) Lack of published intervention studies demonstrating how to better reach the socially disadvantaged **Source of funding:** The project is funded by the Deutsche Forschungsgemeinschaft (German Research Foundation, grant no.: JA 1849/1-1). **Limitations identified by author:** 1) Publication bias 2) Search terms used in this review may have been too narrow 3) The time lag involved in empirical studies observing changes occurring in real life. **Limitations identified by reviewer:** |

| **Authors:** Bock C, Diehl K, Schneider S, Diehm C, Litaker D  **Year:** 2012  **Citation:** Medical Care Research & Review 69(5): 495-518.  **Country of study:**  **Aim of study:**  **Study design:**  **Quality score: (++, + or -):** - |
| --- |
| **Population and setting** |
| **Source population/s:** Two studies within the sample focused on primary care physicians in general; others explicitly targeted general practitioners (n = 5), resident physicians (n = 2), or family physicians (n = 1).  **Setting:** North America (n = 9), the United Kingdom (n = 8), and New Zealand (n = 1).  **Sample characteristics:** Data on a total of 6,338 physicians and 1,783 other primary care providers were represented in this review. The mean proportion of male physicians in the studies weighted by sample size was 71% (range: 46% to 79%). Although not every study reported participants’ ages, the weighted mean for those that did was 41 years (range: 34-45 years).Sample sizes of single studies ranged from 38 to 1,798 physicians (median: 175). **Attrition details:** Not reported |
| **Study design** |
| **Exposure/s description:**  **Physical environment:** Not reported  **Socio-cultural environment:** Socio-economic status  **Economic environment:** Not reported  **Political environment:** Not reported  **Potential confounders:** Not reported  **Inclusion:** Studies were included in this review if they met the following criteria defined a priori: (a) Study participants comprised primary care physicians (e.g. general practitioners, family physicians, general internists) or mixed samples including other care providers who might be involved in behavioural counselling (e.g. nurses, specialist physicians); (b) Observational study design using direct observation or self-report via surveys;  (c) Studies with a clinical focus on CVD prevention or health promotion;  (d) Outcomes focusing on either knowledge, attitudes, or actual delivery of behavioural counselling to reduce cardiovascular risk. Studies were excluded if they examined behavioural counselling for the prevention of diseases other than CVD (e.g. cancer, mental disorders, orthopaedic, or sexual transmitted diseases). |
| **Outcomes and methods of analysis** |
| **Methods of analysis:** Methodological heterogeneity between studies was estimated using I2 statistics. Unable to conduct a meta-analysis as originally planned; report in individual studies with unadjusted 95% CIs. **Outcomes:** Behavioural counselling Lifestyle modification Knowledge Attitudes **Follow-up periods:** Cross-sectional studies or health care surveys |
| **Results** |
| **Quantitative**   - Across studies, seven out of ten physicians felt that behavioural counselling was important and was the physician’s responsibility. A minority said that they had no time to spend on preventive medicine (13% [6%,19%]) or felt that lifestyle advice during routine consultations should not be part of their job (13% [9%,17%]) - Physicians rated lifestyle modification as important for good health in the areas of diet (75% to 84%), exercise (76% to 92%), and smoking (100%). - The proportion of physicians who felt prepared to offer counselling were low for nutrition (28% to 36%), but had adequate knowledge to give advice about physical activity. - Physicians’ perceived self-efficacy in helping patients change their lifestyle was also generally low in the areas of smoking (4% to 25%), nutrition (5% to 27%), exercise (7% to 29%), and alcohol consumption (7% to 21%).   **Qualitative**   - Physician characteristics most closely associated with behavioural counselling included female gender, specific training in health promotion, knowledge as well as positive attitudes, and greater self-efficacy in changing patients’ health behaviour. - Behavioural counselling was more frequently offered to new patients and to those with several CVD risk factors or chronic illness. - Patients’ age and male gender were not consistently observed across studies and differed by the lifestyle factor under consideration. |
| **Notes by review team** |
| **Evidence gaps and/or recommendations for future research noted by study author:** Studies that specifically address these aspects of preventive service delivery are needed for gaining a clearer understanding of their impact on the health of the individual and the public **Source of funding:** The author(s) received no financial support for the research, authorship, and/or publication of this article. **Limitations identified by author:** 1) Selective reporting or non-reporting in the original studies of narrowly defined outcomes may have affected ability to assess full scope of counselling practices for cardiovascular risk 2) Results were limited to studies conducted in developed nations 3) Sample represented in published 4) Studies may not have been representative of the larger population of physicians practicing in settings **Limitations identified by reviewer:** |

| **Authors:** Coles E, Themessl-Huber M, Freeman R  **Year:** 2012  **Citation:** Health Education Research 27(4): 624-44  **Country of study:** Developed industrialised countries  **Aim of study:** To systematically examine the literature to explore ‘What is known about community-based health and health promotion services for homeless people’  **Study design:** Structured review  **Quality score: (++, + or -):** + |
| --- |
| **Population and setting** |
| **Population:** Adults and families experiencing homelessness **Settings:** Developed industrialized countries. Community setting to include hostels, shelters, drop-in centres, food banks, churches, centres for homelessness, kerbside |
| **Study design** |
| **Inclusion:** Studies that met the following criteria:   1. English language; 2. Developed industrialised countries; 3. Adults and families experiencing homelessness; 4. Concerned with engagement with health services or health promotion services irrespective of health condition and to include any physical and/or mental ill-health conditions; 5. Community setting to include hostels, shelters, drop-in centres, food banks, churches, centres for homelessness, kerbside; 6. Primary research or empirical evidence; qualitative or quantitative or mixed methods design. |
| **Outcomes and methods of analysis** |
| Mixed-methods ‘combined separate synthesis’ approach, to blend quantitative and qualitative evidence within in a single review. Meta-synthesis of the combined quantitative and qualitative evidence. Purposive rather than exhaustive data search. Research questions were refined in the process. The quantitative and qualitative findings were merged in an interpretive narrative summary and thematic matrix to address the refined research questions. |
| **Results** |
| - 13 studies included. Three themes emerged:  1. Incorporating homelessness 2. Health improving 3. Health engaging.  - Evidence suggests that as part of a tailored approach to health promotion, homeless people must be actively involved in intervention development, ensuring that appropriate, acceptable and potentially effective individual elements are incorporated into community-based interventions. - The evidence suggests that within the specific context of homelessness, the inputs for health improvement and health promotion priorities must be those identified by homeless people (preparedness), with the role of staff enabling the formation and maintenance of mutually trusting relationships (interaction) with those experiencing homelessness |
| **Notes by review team** |
| **Limitations mentioned:** Review was limited to studies conducted in developed, industrialized countries. Combined separate synthesis methodology necessitates the reporting of the intervention and qualitative studies separately before combining in a thematic synthesis -> possible that critical information is lost as a consequence of combining. |

| **Authors:** Dryden R, Williams B, McCowan C, Themessl-Huber M  **Year:** 2012  **Citation:** BMC Public Health 12: 723  **Country of study:** Developed countries  **Aim of study:** To establish the nature and extent of current knowledge relating to the uptake and engagement with general health checks and preventative health checks for the risk factors of cardiovascular disease in particular  **Study design:** Exploratory narrative scoping review  **Quality score: (++, + or -):** - |
| --- |
| **Population and setting** |
| **Population:** Hard to reach populations, high risk groups **Settings:** Studies in Western/developed countries |
| **Study design** |
| **Inclusion criteria:  Population:** • Western/developed countries • Hard to reach populations  • High risk groups  **Intervention:**  • General health checks • Heart disease health checks • General/Heart AND other disease-specific health check  • Studies whose primary outcome was to increase uptake  • Studies where uptake was documented (of the above interventions) **Control:**  • Control group not necessary **Outcome:** • Initial uptake of screening and/or • Long term engagement with services |
| **Outcomes and methods of analysis** |
| Iterative scoping of the literature to explore the broad state of knowledge regarding attendance at general health checks. Narrative synthesis. |
| **Results** |
| - 39 papers included. - Those least likely to attend health checks were men on low incomes, low SES, unemployed or less well educated. In general, attenders were older than non-attenders. - Marital status was found to affect attendance rates with non-attenders more likely to be single. White individuals were more likely to engage with services than individuals from other ethnic backgrounds. Non-attenders had a greater proportion of cardiovascular risk factors than attenders, and smokers were less likely to attend than non-smokers. - The relationship between health beliefs and health behaviours appeared complex. Non-attenders were shown to value health less strongly, have low self-efficacy, feel less in control of their health and be less likely to believe in the efficacy of health checks. - Routine health check-ups appear to be taken up inequitably, with gender, age, socio-demographic status and ethnicity all associated with differential service use. Furthermore, non-attenders appeared to have greater clinical need or risk factors suggesting that differential uptake may lead to sub-optimal health gain and contribute to inequalities via the inverse care law. - Appropriate service redesign and interventions to encourage increased uptake among these groups is required. |
| **Notes by review team** |
| **Limitations:** Studies may have been missed. Widening inclusion criteria to include both geriatric health checks and non-developed countries might have been beneficial. |

| **Authors:** Murray J, Craigs CL, Hill KM, Honey S, House A  **Year:** 2012  **Citation:** BMC Cardiovascular Disorders 12(120)  **Country of study:** International  **Aim of study:** Clarify which influences reported by patients predict uptake and completion of formal lifestyle change programmes  **Study design:** Systematic review  **Quality score: (++, + or -):** - |
| --- |
| **Population and setting** |
| **Source population/s:** USA 5, UK 7, Australia/New Zealand 9, Canada 4, Rest of Europe (Sweden, Denmark, Poland) 4, Middle East 3  **Setting:** International **Sample characteristics:** Cross sectional 7, Cohort 24, RCT 1, Prospective 23, Retrospective 9 **Attrition details:** Not reported |
| **Study design** |
| **Exposure/s description:  Physical environment:** Not reported  **Socio-cultural environment:** Emotions - Increased anxiety  - Depression  - Stress  - Less distress, lower mental QOL, denial, greater health concerns, higher role resumption Psychological beliefs - Illness less attributed to lifestyles, increased denial of severity of illness - Less control/cure over course of illness/ lower self-efficacy - More symptoms attributed to illness/better understanding of illness/illness has greater consequences Information & communication - Less education  - Less awareness of blood pressure level - Less awareness/knowledge of total cholesterol level or recommended activity levels Friends & family support Not married / not living with a partner / being single Economic environment:  - Longer commute time  - Greater distance from venue  - Problems with transport, rural area - Occupation type - blue collar (vs white) - Unemployed / retired/home maker  - Higher income  - Having health insurance **Political environment:** Not reported  **Potential confounders** Not reported  **Inclusion:** Primary or secondary quantitative research studies examining uptake (attending at least one session) or completion (attending all sessions) of lifestyle behaviour change programmes in adults (>18 years of age) having experienced angina, myocardial infarction or transient ischemic attack, or with hypertension, diabetes type II, coronary artery disease or hypercholesterolemia.  Studies were excluded if they: 1. Investigated compliance with medication for cardiovascular risk management or long term maintenance of lifestyle change (these are arguably separate bodies of research and their inclusion would result in an unwieldy report); 2. Focused on a selected population with specialist needs: 3. Were culturally unrepresentative of the main ethnic groups residing in Europe; 4. Included only stroke, chronic obstructive pulmonary disease, peripheral artery disease, and heart failure patients or; consisted entirely of Diabetes type I patients (most likely to involve adolescents).  Primary research studies were further required to report statistical effects. Reviews were included if they were statistical meta-analyses of selected factors or narrative reviews with clear reporting of statistical effects of factors. |
| **Outcomes and methods of analysis** |
| **Methods of analysis:** Narrative synthesis **Outcomes:** Factors most consistently associated with uptake of lifestyle change related to support from family and friends, transport and other costs, and beliefs about the causes of illness and lifestyle change. **Follow-up periods:** Not reported |
| **Results** |
| **Quantitative**   - Key themes that contained factors most consistently associated with uptake at lifestyle programmes were ‘friends and family support’, ‘transport and other costs’, and ‘psychological beliefs’ - Problems with transport, perception of greater consequences to illness and attribution of more symptoms to illness were most consistently predictive of uptake. - Absence of a partner, lack of employment, transport / distance problems, low self-efficacy and perceptions of less control of the illness were commonly predictive of non-uptake   **Qualitative**   - Emotions  Uptake - Anxiety state; alexithymia; distress caused by symptoms; emotional health (profile of mood state; post-traumatic stress disorder, self-motivation Completion - problem focused coping; maladaptive coping - Psychological beliefs Uptake – Overall health beliefs; multidimensional health locus of control; illness perceptions personal control; illness perceptions treatment control; illness perceptions timeline Completion - Emotional representations; time cyclical (symptoms change) - Information and communication  Uptake - Knowledge of smoking recommendation - Family & friends support  Uptake - Living alone; relationship difficulties Completion - Living arrangements - Transport & cost  Uptake – occupation; transport cost and financial difficulty; distance from centre Completion – Transport problems; income; occupation, ‘practical barriers’ (broadly defined) |
| **Notes by review team** |
| **Evidence gaps and/or recommendations for future research noted by study author:** 1) There was a paucity of studies reporting the relationship between uptake or completion of lifestyle change support and factors relating to ‘referrals’, ‘culture’, ‘social support’, ‘the role of the health care professional’, ‘attitudes to rehabilitation’, ‘attitudes to exercise’ and ‘balancing and integrating health care needs with everyday life’ 2) Limited awareness of the main predictors of uptake of lifestyle change 3) Formalise these areas into an approach that can characterise patient responses in these areas and guide decision making about the most suitable type **Source of funding:** This work was supported by the National Institute of Health Research (KRD/012/001/006). **Limitations identified by author:** 1) Factors show inconsistent patterns with respect to uptake and completion of lifestyle change programmes 2) Representation of patient reported factors was in general not good 3) Publication bias 4) Studies reported in the current review that were derived from the systematic review by Cooper et al. were not assessed for quality by either the team or in the previous review **Limitations identified by reviewer:** Adults (>18 years of age) having experienced angina, myocardial infarction or transient ischemic attack, or with hypertension, diabetes type II, coronary artery disease or hypercholesterolemia. |

| **Authors:** Hart PL  **Year:** 2005  **Citation:** Journal of Cardiovascular Nursing 20(3): 170-6  **Country of study:** International  **Aim of study:** Report the results of an integrative review of nursing research related to women’s perceptions of risks for heart disease  **Study design:** Review  **Quality score: (++, + or -):** - |
| --- |
| **Population and setting** |
| **Source population:** More than half of the studies only had participants who were Caucasian women. Limited representation of African American, Hispanic, and Asian/Pacific women were in the study sample populations. **Setting:** International  **Sample characteristics:** Women.  **Attrition details:** Not reported |
| **Study design** |
| **Exposure/s description:**  **Physical environment:** Not reported  **Socio-cultural environment:** knowledge of risk, lack of time, family obligations, ethnicity, diet high in saturated fat, lack of exercise, Economic environment: Income or lack of money, fear for one’s own safety, job status.  **Political environment:** Not reported  **Potential confounders:** Age.  **Inclusion:** Not reported |
| **Outcomes and methods of analysis** |
| **Methods of analysis:**  **Outcomes:**  **Follow-up periods:** |
| **Results** |
| - Qualitative barriers to health-promoting behaviour are role and caretaking responsibilities, athletic incompetence or lack of exercise experience, lack of money, lack of time, and fear for one’s own safety and family obligations. Income plays a role in the type of health-promoting behaviours engaged in by women. - Women with higher incomes were involved in cardiovascular risk-reducing behaviour such as diet control, exercise, and weight management, diet high in saturated fat, lack of exercise, family history of CHD, and hypertension were the most common risk factors identified by women. Age, job status, knowledge of CHD risk factors, and family history do not positively influence health promoting behaviour of women. |
| **Notes by review team** |
| **Evidence gaps and/or recommendations for future research noted by study author:** Explore women’s perceptions of CHD risk factors. Understanding the relationship between perceived risk for CHD in women and motivation to engage in health-promoting behaviour, explore the conflict between current behaviour and barriers to behaviour change.  **Source of funding:**  **Limitations identified by author:**  **Limitations identified by reviewer:** Study selection was limited to the first author being a nurse researcher |

| **Authors:** Ryan A  **Year:** 2009  **Citation:** BMC Public Health 9(96)  **Country of study:** UK  **Aim of study:** Review evidence about who uses self-tests and other self-care activities  **Study design:** Review  **Quality score: (++, + or -):** + |
| --- |
| **Population and setting** |
| **Source population:** Not reported  **Setting:** UK  **Sample characteristics:** Not reported  **Attrition details:** Not reported |
| **Study design** |
| **Exposure/s description:**  **Physical environment**: Not reported  **Socio-cultural environment:** Not reported  **Economic environment:** Not reported  **Political environment:** Not reported  **Potential confounders:** Income and occupation.  **Inclusion:** Studies were included if they were published during the last 15 years in a peer-reviewed journal and they reported factors, reasons or characteristics associated with a relevant activity among adults resident in the UK.  Studies were excluded if they did not concern a relevant activity or report factors, reasons or characteristics associated with an activity. Remaining studies were then excluded:   1. If they did not involve adults or did not differentiate between children and adults; 2. If they specified that the activity was initiated by a doctor or nurse; 3. If they only studied intention or willingness to do an activity; 4. If they involved people with specific conditions where the results would not be generalisable; 5. If they did not involve UK residents or differentiate between residents of the UK and other countries. 6. Finally, reviews, letters or opinions were excluded, although reviews were retrieved so that relevant references could be identified. |
| **Outcomes and methods of analysis** |
| **Methods of analysis:**  **Outcomes:**  **Follow-up periods:** |
| **Results** |
| - Qualitative. - People who engaged in self-care activities were likely to be affluent and/or educated, taking non-prescribed alternative medicines was more likely in women than men, and people of Black African origin were more likely than white people or people of South Asian origin to take non-prescribed alternative medicines. - One high quality population-based survey reported on ethnicity: this found that people who were white were more likely to use herbal supplements than other people. Non-smokers and people who took regular exercise were more likely to have seen a chiropractor or osteopath than other people. - Two high quality population-based surveys examined behaviour. One found that herbal supplement use was associated with not smoking and being active, although only being active remained significant after adjusting for other variables |
| **Notes by review team** |
| **Evidence gaps and/or recommendations for future research noted by study author:**  **Source of funding:** Not reported  **Limitations identified by author:**  **Limitations identified by reviewer:** Only seven includes used population-based samples |

| **Authors:** Yarcheski A, Mahon NE, Yarcheski TJ, Cannella BL  **Year:** 2004  **Citation:** Journal of Nursing Scholarship 36(2): 102-8  **Country of study:** International  **Aim of study:** Determine magnitude between positive health practices and predictor variables  **Study design:**  **Quality score: (++, + or -):** + |
| --- |
| **Population and setting** |
| **Source population:** Not reported  **Setting:** USA (n=25), England (n=2), Canada (n=1)  **Sample characteristics:** Not reported:  **Attrition details:** Not reported |
| **Study design** |
| **Exposure/s description:**  **Physical environment:** Loneliness, social support, perceived health status, self-efficacy, future time perspective, self-esteem, hope and depression.  **Socio-cultural environment:** Stress, education, marital status, age, and sex.  **Economic environment:** Income  **Political environment:** Not reported  **Potential confounders:** Not reported  **Inclusion**: Studies were included when:   1. PLQ used to measure positive health practices- different measures could be used for each predictor; 2. Minimum of three reports included; 3. Adequate statistics were reported 4. Published in English |
| **Outcomes and methods of analysis** |
| **Methods of analysis:**  **Outcomes:**  **Follow-up periods:** |
| **Results** |
| - Quantitative. - Eight predictors (loneliness, social support, perceived health status, self-efficacy, future time perspective, self-esteem, hope and depression) had moderate effect sizes Six (stress, education, marital status, age, income, and sex) had small effect sizes. |
| **Notes by review team** |
| **Evidence gaps and/or recommendations for future research noted by study author:** Not reported .  **Source of funding:** Not reported.  **Limitations identified by author:** Not reported.  **Limitations identified by reviewer:** Unclear who the people included are; age; ethnicity etc. |

| **Authors:** Kurian AK, Cardarelli KM  **Year:** 2007  **Citation:** Ethnicity & Disease 16(1): 143-52  **Country of study:**  **Aim of study:** expand our understanding of the factors associated with racial/ethnic disparities in cardiovascular disease (CVD) risk factors  **Study design:** Review  **Quality score: (++, + or -):** - |
| --- |
| **Population and setting** |
| **Source population:** USA  **Setting:** USA.  **Sample characteristics:** Defined in racial/ethnic minority populations  **Attrition details:** Not reported |
| **Study design** |
| **Exposure/s description:**  **Physical environment:** Not reported.  **Socio-cultural environment:** Diet, Leisure-time.  **Economic environment:** Not reported.  **Political environment:** Not reported.  **Potential confounders:** Education and income levels  **Inclusion:** English-language, population-based CVD studies published from 1995 to the present, which included one or more racial/ethnic comparison, with two or more CVD risk factors studied, in adult population |
| **Outcomes and methods of analysis** |
| **Methods of analysis:**  **Outcomes:**  **Follow-up periods:** |
| **Results** |
| **Quantitative**   - No one racial/ethnic minority population was consistently found to have a higher or lower prevalence of hypercholesterolemia. Mexican American women had the highest prevalence of no leisure-time physical activity compared to Black and White women. - American Indian Alaskan Native had higher prevalence of no leisure-time physical activity compared Whites. - After adjusting for demographic differences, they reported that only Black people were significantly more likely to report a higher level of physical inactivity compared to White people.   **Qualitative**   - Mexican Americans had a significantly lower prevalence of smoking than other ethnic groups. - American Indian, Alaskan Native populations had significantly higher prevalence of smoking compared to White populations |
| **Notes by review team** |
| **Evidence gaps and/or recommendations for future research noted by study author:** Consider the impact of more fundamental determinants of CVD risk factors  **Source of funding:** Not reported  **Limitations identified by author:** Race is often considered a proxy for socio-economic conditions and environmental factors diverse study designs lack of standardized operational definition of modifiable CVD risk factors  **Limitations identified by reviewer:** Poor description of strategy and process |

Primary qualitative studies

## C1.1 Physical Activity: Men

| **Authors:** Caperchione CM, Vandelanotte C, Kolt GS, Duncan M, Ellison M, George E, Mummery WK.  **Year:** 2012  **Citation:** American Journal of Men’s Health 6(6): 453-461  **Country of study:** Australia  **Aim of study:** Understanding the challenges and motivations to physical activity participation and healthy eating in middle-aged Australian men  **Study design:** Qualitative - focus group study  **Quality score: (++, + or -):** + |
| --- |
| **Population and setting** |
| Australian men, central Queensland (n=30).  Area includes urban, rural and coastal areas; Mean age 43.8 (SD 10.84), 89% had university or technical studies education, 96.7% in full time work.  Ethnicity: not reported |
| **Study design** |
| Six focus group sessions conducted over three months. Participants recruited from local industries and organisations. |
| **Outcomes and methods of analysis** |
| Data analysis focused on themes concerning the challenges and motivations to men’s physical activity participation and health eating behaviours. Two researchers identified themes and reached consensus regarding emerging themes through discussion to resolve discrepancies. |
| **Results** |
| - **Knowledge and awareness of PA:** The majority of participants had a very good understanding of what constitutes PA and of PA guidelines. Many participants also acknowledged that being physically active meant decreasing sedentary time. - **Barriers and challenges of PA:** Lack of time to be physically active, other factors often took priority, work, child care and family responsibilities. Participants also reported laziness or lack of motivation to be physically active, many PA programs (other than sport) are not of interest to them. - **Motivations for engaging in PA:** For better health, to lose weight and feel better, prevent disease, to be good role models for children and educate them about healthy living, ensuring they stayed healthy enough to undertake essential daily activities and activities they enjoyed e.g. recreation, travel, hobbies as they got older, fear of becoming ill. |
| **Notes by review team** |
|  |

| **Authors:** Hooker SP, Wilcox S, Rheaume CE, Burroughs EL, Friedman DB  **Year:** 2011  **Citation:** Ethnicity & Disease 21(3): 261-267  **Country of study:** US  **Aim of study:** Factors related to physical activity and recommended intervention strategies as told by midlife and older African American men.  **Study design:**  **Quality score: (++, + or -):** + |
| --- |
| **Population and setting** |
| African American men (n=49), aged 45-88 years.  Recruitment was via mailings and announcements to county departments on ageing, senior centres, senior residential communities, participants in previous research projects, mass communication and word of mouth. |
| **Study design** |
| Personal interviews about barriers to PA, enablers and preferences, and components that would render a PA programme appropriate for and appealing to AA men of similar ages. |
| **Outcomes and methods of analysis** |
| Common themes were identified by multiple research staff. Inter-rater agreement of at least 85% between coders was considered an acceptable threshold for coding consistency. Major themes extracted from the data. |
| **Results** |
| - **Preferences:** Preference for walking as a good form of PA. - **Benefits:** Improving muscle tone, strength, stamina, sexual performance, physical appearance; medical benefits e.g. improving blood pressure; quality of life benefits - being healthier, feeling better, living longer. - **Barriers:** Physical ailments and chronic conditions , lack of time due to conflicts with work, family and other responsibilities, lack of motivation, lack of support from spouse, partner, children, friends; lack of access including costs for gyms, limited places and inconvenience e.g. having to drive to do PA. - **Intervention strategies:** Walking most commonly recommended PA, then sports-related activities e.g. tennis, golf, basketball etc. Gyms, recreation centres, church recommended as locations for PA, camaraderie and fellowship, a partner to be active with. However, most men favoured being in a program with only other men. - Competition with team sports, games etc mentioned by some men or being encouraged to compete within themselves for self-motivation. Other elements e.g. nutrition and healthy eating. |
| **Notes by review team** |
|  |

| **Authors:** Hooker SP, Wilcox S, Burroughs EL, Rheaume CE, Courtenay W.  **Year:** 2012  **Citation:** Journal of Men’s Health 9(2): 79-88  **Country of study:** US  **Aim of study:** The potential influence of masculine identity on health-improving behaviour in mid-life and older African American men.  **Study design:** Qualitative - Interview study  **Quality score: (++, + or -):** + |
| --- |
| **Population and setting** |
| African American men (n=49), aged 45-88 years.  Recruitment was via mailings and announcements to county departments on aging, senior centres, senior residential communities, participants in previous research projects, mass communication and word of mouth.  Population as above? |
| **Study design** |
| Personal interviews using an interview guide including questions about general health, masculine identity, about barriers, enablers and preferences to PA. A brief masculine identity survey was also completed to assess participants’ attitudes about being a man. |
| **Outcomes and methods of analysis** |
| Comparing and contrasting emerging themes within and across the interviews was used to detect similarities and differences in the data. |
| **Results** |
| - Potential negative and positive influences of manhood on health included avoiding healthcare appointments and being a good example to children/others. Responses could be assigned to either a positive or negative influence on health. The concept of being a man led them to hide any signs of pain or suffering. - The concepts of tough, macho and reckless were also related to poor health behaviours such as drinking, smoking, poor diet and being sedentary, avoiding doctor visits and other health appointments. - On the positive side, the concept of responsibility was prevalent in that many men described remaining physically active, eating better and getting adequate rest so that they could take care of themselves and live longer, and thereby provide better for their families. |
| **Notes by review team** |
|  |

| **Authors:** Vandelanotte C, Caperchione CM, Ellison M, George ES, Maeder A, Kolt GS… Mummery WK  **Year:** 2013  **Citation:** Journal of Health Communication 18(9): 1070-1083  **Country of study:** Australia  **Aim of study:** What kinds of website and mobile phone-delivered physical activity and nutrition interventions do middle-aged men want?  **Study design:** Qualitative - focus group study  **Quality score: (++, + or -):** + |
| --- |
| **Population and setting** |
| Australian men, central Queensland (n=30).  Age range 35-54 years, mean age 43.8 (SD 10.8).  Ethnicity: not reported.  Recruitment was from local industries and university. The majority of the men were confident using the internet (80%), owned a 3G capable mobile phone (80%) and were not meeting PA or fruit and vegetable intake guidelines. |
| **Study design** |
| Six focus group sessions using a discussion guide to facilitate the conversations and to unveil participants’ opinions, perceptions, beliefs about the use of the internet and mobile phones to improve physical activity and nutrition behaviours. |
| **Outcomes and methods of analysis** |
| Data analysis used an inductive approach, focusing on themes concerning the use of the internet and mobile phones to improve PA and nutrition behaviours. |
| **Results** |
| Six themes were identified:   1. Internet experience - about current experience and internet skills; 2. Preferred website characteristics - websites need to be fast, very easy to use, be clean with clutter-free pages and use concise language, with reliable factual information endorsed by a trustworthy organisation; 3. Web 2.0 and social networking applications: time is a major limiting factor so social networking is not a high priority, support for interactive features that could give feedback; 4. Specific website features - podcasts, instructional videos, and step-by-step pictures were highly supported by the participants, with ordinary average men portrayed in the pictures; 5. Online PA and diet self-monitoring - supportive of the concept of using self-monitoring tools for PA and diet on the internet, there was concern it might be inconvenient and time-consuming which would lead to lack of adherence over a short period of time. Majority more interested in personal goals or challenges than competing with others; 6. Mobile phones as a method of intervention delivery - not of interest to most participants, though more open to the idea if they had a smartphone. |
| **Notes by review team** |
|  |

## C1.2 Physical Activity: Women

| **Authors:** Berg JA, Cromwell SL, Arnett M  **Year:** 2002  **Citation:** Health Care for Women International 23(8): 894-904  **Country of study:** US  **Aim of study:** Physical activity perspectives of Mexican American and Anglo American Midlife women.  **Study design:** Qualitative - focus group study (Internet)  **Quality score: (++, + or -):** + |
| --- |
| **Population and setting** |
| Anglo-American (n=6), Mexican American (n=10) women in Arizona, not currently participating in an organised PA programme.  Women were recruited from community social groups and churches. |
| **Study design** |
| Three focus groups conducted: one Anglo-American, one Mexican American (conducted in Spanish) and one Mexican American (conducted in English). A semi-structured interview guide was used that posed questions about what participants believed were the benefits of and barriers to PA. |
| **Outcomes and methods of analysis** |
| Transcripts from each focus group analysed separately initially but data from the two Mexican American groups were combined |
| **Results** |
| **Anglo-American women**   - **Factors preventing PA:** Physical problems, no physical talent, inability to keep up with the group, lack of time and too many other activities, most PA not appropriate for women, social discomfort in engaging with group PA. - **Factors promoting involvement:** A formal group needed for motivation, a routine makes it easier to continue, the PA must be enjoyable in order for the women to participate, mixed responses to verbal encouragement, acceptable if it is sincere.   **Mexican American women**   - **Barriers to participation in PA:** too old to engage in PA, belief that no physical change possible, self-conciousness about current appearance, environmental factors including transport, cost of organised activity, other time commitments including taking care of family, church attendance, need approval of family members, particularly husbands. - **Motivators to participation in PA:** to join family/peers, live longer, improved health and energy, maintain or enhance ability to care for family, PA that could be shared with family members. |
| **Notes by review team** |
|  |

| **Authors:** Im EO, Ko Y, Hwang H, Chee W, Stuifbergen A, Lee H, Chee E  **Year:** 2012  **Citation:** JOGNN: Journal of Obstetric, Gynecologic& Neonatal Nursing 41(5): 650-658  **Country of study:** USA  **Aim of study:** Asian American midlife women’s attitudes towards physical activity (online forum**).**  **Study design:** Qualitative - focus group study  **Quality score: (++, + or -):** + |
| --- |
| **Population and setting** |
| Asian American women (n=17), aged 40 to 60 years (mean age 49 yrs, SD 5.9).  Women were recruited through internet communities for midlife women and internet communities for ethnic minorities. For inclusion, women had to be ambulatory and able to participate in all forms of PA, could read and write English. Those who had high CVD or musculoskeletal risk factors were excluded. |
| **Study design** |
| Internet online forum topics included attitudes to physical activity, and seven topics on ethnic specific topics, through a project website. |
| **Outcomes and methods of analysis** |
| Thematic analysis with constant monitoring of themes and ideas and quality of analysis throughout the process. |
| **Results** |
| Three major themes relating to Asian American midlife women’s attitudes towards PA:   1. Keeping traditions - maintaining traditional foods and culture, physical activity incorporated into their lives through household chores, extra time in the day was reserved for family, cultural networking and difficulty in participating in PA with those from other cultures because of language barriers or differences in cultural background, acculturation to US culture of more driving and less walking 2. Not a priority - priority for most was children, sacrifice their own needs, more opportunities as children become older, Asian culture prioritises intellectual activity over PA, family or social events prioritised 3. Not for Asian girls - lack of encouragement, perceived lack of physical ability |
| **Notes by review team** |
|  |

| **Authors:** Im EO, Ko Y, Hwang H, Chee W, Stuifbergen A, Walker L, Brown A  **Year:** 2013  **Citation:** Journal of Midwifery & Women’s Health 58(4): 440-450  **Country of study:** US  **Aim of study:** Exploring midlife women's attitudes toward physical activity  **Study design:** Qualitative - focus group study (online forum)  **Quality score: (++, + or -):** + |
| --- |
| **Population and setting** |
| Midlife women (n=90), aged 40-60 years (mean age 49.4, SD 5.2).  Ethnicity: 29 white, 23 Hispanic, 21 African American, 17 Asian women.  Women had to be ambulatory and able to participate in all forms of PA. Recruited through 2309 internet communities for midlife women and 4421 internet communities for ethnic minorities. |
| **Study design** |
| Internet online forum topics included attitudes to physical activity including gender and racial/ethnic differences in PA, and separate racial/ethnic specific online forums through a project website |
| **Outcomes and methods of analysis** |
| Two sets of online forum topics on attitudes towards PA and racial/ethnic specific contexts were used for all the online forums. Data from the four groups was analysed as a whole by three analysts at the level of codes using thematic analysis. |
| **Results** |
| Attitudes towards PA that were common across all ethnic groups were represented in five themes:   1. PA is good for health - importance of PA to maintain a healthy life; 2. Not as active as I could be - main reason was lack of time, sedentary lifestyle, family responsibilities, life events 3. PA was not encouraged - gender, and culture cited by Hispanic and Asian women; 4. Inherited diseases motivated participation in PA e.g. high blood pressure, diabetes, obesity, stroke; 5. Lack of accessibility to PA - safety of neighbourhood environment and financial issues. |
| **Notes by review team** |
|  |

| **Authors:** Folta SC, Goldberg JP, Lichtenstein AH, Seguin R, Reed PN, Nelson ME  **Year:** 2008  **Citation:** Preventing Chronic Disease 5(1): A06.1-9  **Country of study:** US  **Aim of study:** Factors related to cardiovascular disease risk reduction in midlife and older women  **Study design:** Qualitative - focus group study/interview study  **Quality score: (++, + or -):** + |
| --- |
| **Population and setting** |
| Sedentary, white women (n=38), aged 40 years or older (age range 40 to late 80s) in Kansas and Arkansas and 25 Cooperative State Research, Education and Extension Service agents in those states. |
| **Study design** |
| Four focus groups with women and interviews with the Cooperative State Research, Education and Extension Service agents. Also, environmental audits of grocery stores and the physical environment were done in three communities. |
| **Outcomes and methods of analysis** |
| The discussion guide for the focus groups was designed to address four key topic areas:   1. Awareness and knowledge about CVD risk factors; 2. Attitudes, perceptions and barriers about PA; 3. Attitudes, perceptions and barriers regarding a heart healthy diet; 4. Opinions about nutrition and PA interventions. |
| **Results** |
| 1. Most women were aware of modifiable risk factors for CVD 2. Barriers to PA included weather, disruption to routine, feeling self-conscious in the gym for indoor activity, PA boring, did not want another commitment and would like strategies for incorporating PA into their regular lifestyle e.g. taking stairs rather than the elevator. 3. Common barriers to achieving a heart-healthy diet were time (especially for women with children at home) and concern about wasting food. Although heart-healthy foods were readily available, women said they found it difficult to avoid less healthy foods and high calorie unhealthy snacks. Other barriers included pressure to eat at social events, perception of conflicting health messages, hunger when they try to cut down on portion sizes, lack of menu planning leading to eating out, not liking fruit and vegetables, difficulty in changing eating patterns from childhood. 4. Liked hands on interventions that helped putting knowledge into practice, with reasonable realistic goals and recognition for achieving goals. |
| **Notes by review team** |
|  |

| **Authors:** Yarwood J, Carryer J, Gagan MJ  **Year:** 2005  **Citation:** Nursing Praxis in New Zealand, 21 (3), 24-37  **Country of study:** New Zealand  **Aim of study:** Factors influencing ability of midlife women to maintain PA over time.  **Study design:** Qualitative - interview study  **Quality score: (++, + or -):** + |
| --- |
| **Population and setting** |
| Midlife women (n=10), aged 37-55.  One woman was Maori, two English, seven New Zealanders.  Educational qualifications ranged from completion of secondary schooling to postgraduate qualifications. |
| **Study design** |
| A qualitative feminist approach used to guide the study. Two semi-structured interviews conducted to explore factors influencing their ability to maintain PA over time. |
| **Outcomes and methods of analysis** |
| Thematic analysis. Early analysis produced 29 themes, all of which were integrated then distilled into four core themes. |
| **Results** |
| Four core themes:   1. ‘Exercise is part of me part of my life’ - PA became part of who they were. Positive feelings associated with exercise not only improved their self-esteem but also their body image. The possibility of a slim body is seen to focus resolve. Positive feelings related to exercise - feelings of wellbeing and enjoyment; 2. Exercise and ageing - a motivating factor was to be fit and healthy in later years in order to enjoy life; 3. Disease prevention, stress release, mental health and weight control. PA whether that was walking, running, biking, gardening etc. involvement on their terms; 4. Social roles - incorporating PA into a busy life can be difficult, family life. Lack of time was a constant challenge, financial constraints for gym membership etc, guilt about balancing work, childcare preventing activity; injuries, health issues and family concerns were all barriers. |
| **Notes by review team** |
|  |

| **Authors:** Segar M, Spruijt-Metz D, Nolen-Hoeksema S  **Year:** 2006  **Citation:** Sex Roles 54(3-4): 175-187  **Country of study:** US  **Aim of study:** To investigate the relationship between midlife women's physical activity motives and their participation in physical activity.  **Study design:** Qualitative - survey  **Quality score: (++, + or -):** + |
| --- |
| **Population and setting** |
| Midlife women (n= 59), European-Americans 86%, Latinas 2%, African Americans 10%; 71% had a college or advanced degree, (mean age 45.6 years, SD 6.8).  Inclusion criteria: women aged 35-60.  Participants recruited from the University of Michigan Women’s Health Registry, the purpose of which is to increase the participation of women in clinical research. |
| **Study design** |
| An introductory letter and copy of the survey were mailed to all participants who matched the study criteria. There was a 70% response rate to the surveys. |
| **Outcomes and methods of analysis** |
| Inductive qualitative methods used to determine participants motives for PA. Participants were asked to write a narrative about being physically active. Also two survey questions asked:   1. What would your most important goal be for doing your imagined PA? 2. What would your reasons be for choosing to participate in your chosen PA.   Participants were coded as having body shape motives (related to weight loss or body shape) if they if they wanted to lose or maintain weight or if they included the words shape, calories or toning in their open-ended responses. |
| **Results** |
| - 44% of participants were categorised as having motives related to weight loss and/or body shape - 56% reported motives that were not related to body shape - There were no significant differences between participants who had body shape motives in BMI or demographic variables. - Participants who had body shape motives for being physically active reported less PA participation than did those whose motives were related to things other than body shape, toning or losing weight. |
| **Notes by review team** |
|  |

| **Authors:** Vaughn S  **Year:** 2009  **Citation:** Rehabilitation Nursing 34(1): 17-23  **Country of study:** Latin America  **Aim of study:** Factors that influence the participation of middle-aged and older Latin-American women in physical activity  **Study design:** Interview study  **Quality score: (++, + or -):** + |
| --- |
| **Population and setting** |
| Latin-American women (n=25) divided into 2 cohorts:- women 40-60 years old were assigned to middle-aged group (n=13) and women 61-85 (n=12) were placed in the older group. |
| **Study design** |
| Ethnographic research design used to study the factors that influence participation of middle-aged and older Latin-American women in regular PA and exercise. Open-ended questionnaire used to guide interviews. Perceptions of health, the health activities in which they are engaged and the factors that influenced their participation in PA comprised the three categories of response. |
| **Outcomes and methods of analysis** |
| Transcribed data and field notes analysed for common themes. |
| **Results** |
| - Factors that facilitated the women's participation in PA were identified as 'sense of self', decreased feelings of stress, feeling good about one's self, a sense of wellbeing, a desire to manage chronic diseases such as hypertension, a desire to lose weight and having experienced a personal health event. - Barriers to physical activity: Physical illness or disability, pain, fatigue, lack of self-motivation, worry and embarrassment. Extrinsic barriers included various role demands of Latin-American women, including child care and household chores or tasks, time limitations cited by participants working outside the home, negative environmental considerations such as unsafe neighbourhoods or weather, availability of and access to community based programs. Some of the Latin-American women expressed fears about getting lost and not being able to ask for directions because of language barriers. |
| **Notes by review team** |
|  |

## C1.3 Physical Activity: General

| **Authors:** Withall J, Jago R, Fox KR  **Year:** 2010  **Citation:** Health Education Journal 70(2): 206-216.  **Country of study:** UK  **Aim of study:** Who attends physical activity programmes in deprived neighbourhoods?  **Study design:** Survey  **Quality score: (++, + or -):** ++ |
| --- |
| **Population and setting** |
| Setting was a highly deprived suburb of Bristol with the city's lowest life expectancy.  A questionnaire was completed by 152 adult and adolescent PA session participants: 74% were adults (19% 18-34y, 11% 35-54y, 45% >55y). 88% were White, 8% Black/AfroCaribbean, 3% Asian. |
| **Study design** |
| Desk research and venue visits were used to assess provision of physical activity sessions. Local publications, directories, websites and contacts used to generate a list of local PA sessions. Venues visited to confirm sessions were currently running and to add any additional activities.  The survey comprised a questionnaire collecting age, gender, postcode, height, weight, ethnicity, attendance duration, and regularity, attendance with a friend, communications channel and any other activity sessions or community groups. |
| **Outcomes and methods of analysis** |
| Chi squared tests of independence were used to examine differences in area of residence, gender, age and BMI range. |
| **Results** |
| - The majority of participants were female (76.3%), 56% over 55 years of age, 37.5% overweight/obese. - Only 45.4% of participants were resident in the study area. - There was no significant difference in the type of activity attended by gender. - The overweight/obese attended more strength and flexibility sessions such as yoga and tai chi. - 18-34 y attended aerobic and sports sessions. - 35-54 y predominantly attended aerobic sessions, with over 55 y spread more evenly over aerobic, strength and flexibility sessions, dance and sport. - Activities developed and delivered by local residents or groups attracted most local participants. - Local authority funded exercise initiatives are not very successful at reaching their target group, particularly for men. |
| **Notes by review team** |
|  |

| **Authors:** Rimmer JH, Riley B, Wang E, Rauworth A., Jurkowski J  **Year:** 2004  **Citation:** American Journal of Preventive Medicine 26(5), 419-425  **Country of study:** US  **Aim of study:** Physical activity participation among persons with disabilities: barriers and facilitators  **Study design:** Qualitative - focus group study  **Quality score: (++, + or -):** + |
| --- |
| **Population and setting** |
| Conducted in 10 regions of the US in 2001 to 2002.  Participants were:   1. Consumers with disabilities (n=42); mean age 40.2 (SD 12.8); 54.8% male; 2. Architects 3. Fitness and recreation professionals 4. City planners and park district managers   Number of participants not reported (except for consumers with disabilities (above)). |
| **Study design** |
| Focus groups were facilitated by two members of the research team. Participants recruited through the Disability and Business Instructional Technology Assistance Centres. |
| **Outcomes and methods of analysis** |
| Notes taken during the focus group sessions were analysed using a note-based approach to identify major themes. Tape recordings were then analysed according to the themes identified through note analysis |
| **Results** |
| 178 barriers and 130 facilitators to the physical activity participation of persons with disabilities. Only major themes are reported here due to lack of space. See original paper for full details. Major themes were   - Barriers and facilitators relating to the built and natural environment - Economic issues - Emotional and psychological barriers - Equipment barriers - Barriers related to the use and interpretation of guidelines - Information-related barriers - Professional knowledge, education and training issues - Perceptions and attitudes of people who are not disabled - Policies and procedures - Availability of resources |
| **Notes by review team** |
|  |

## C1.4 Diet

| **Authors:** Brown NA, Smith KC, Kromm EE  **Year:** 2012  **Citation:** Women & Health 52(3): 234-251  **Country of study:** US  **Aim of study:** To determine the perception of women of the relationship between recent life events, transitions and diet in midlife  **Study design:** Qualitative - focus group study  **Quality score: (++, + or -):** + |
| --- |
| **Population and setting** |
| Women (n=43) with limited financial resources (incomes below 250% of the federal poverty level), aged 40-64, conducted in Maryland.  Ethnicity not formally reported but authors report all groups were diverse in terms of race/ethnicity and culture of origin. |
| **Study design** |
| Four focus groups in women aged 40 to 50 years and four focus groups in women aged 51 to 64. Recruitment was from the county database of women served by the Centers for Disease Control and Prevention funded breast and cervical cancer early detection programme (the programme offers free screening to uninsured and underinsured women with incomes below 250% of the federal poverty level). |
| **Outcomes and methods of analysis** |
| The analysis was based on data initially coded for the thematic category ‘life course changes’. This was not the primary focus of the overall study, but during the data coding and analysis a theme related to issues of recent changes to family structure and household composition emerged as important factors in how women understood their diet. |
| **Results** |
| Transitions and events related to household structure, health status, phases of motherhood, and shifts in financial employment status all had the potential impact on women's dietary decisions and dietary behaviours. These themes were consistent across both age groups studies. |
| **Notes by review team** |
| The authors did not collect demographic participant data except age range. The data presented emphasises the complexity and interconnectedness of multiple factors and it is not clear from the data which factors are consistent barriers or facilitators to healthy dietary behaviours. Different factors may differ in different people e.g. children leaving home could disrupt eating habits and patterns, but for some people it encouraged other behaviours (e.g. taking up cooking again). |

| **Authors:** Hammond GK, Chapman GE, Barr SI  **Year:** 2011  **Citation:** Journal of Human Nutrition & Dietetics 24(1): 61-67  **Country of study:** Canada  **Aim of study:** Healthy midlife women: how bone health is considered in their food choice systems  **Study design:** Qualitative - focus group study  **Quality score: (++, + or -):** + |
| --- |
| **Population and setting** |
| Midlife women (n= 36) from upper, middle and lower income neighbourhoods.  Recruitment aimed at age 40-55, but two people recruited older than this and two younger. Women outside this age range expressed similar views to other women. |
| **Study design** |
| Six focus groups conducted, four at community sites, one in a participant's home and one in a women's housing complex. Each woman received a $20 honorarium on completion. The focus groups discussed factors the women currently consider when making food decisions and how bone health fits into their food choice processes. |
| **Outcomes and methods of analysis** |
| Thematic analysis was used to compare shared themes across the three income groups and themes within income groups that indicate how women consider bone health in their food choices. |
| **Results** |
| - All participants were aware of osteoporosis. Bone health was considered an important component of overall health but only one of many competing demands involved in making food decisions. Most women did not actively prioritise bone-health considerations in their diets. - The goal of women in all focus groups was to 'simplify' food decisions that support overall health rather than making multiple dietary decisions to address different aspects of health. - Most women were not motivated to change their diets. Few had deliberately increased their intake of calcium and vitamin D through foods and supplements. For those that had made changes a motivating factor was the diagnosis of osteoporosis for a family member. |
| **Notes by review team** |
|  |

| **Authors:** Jilcott SB, Laraia BA, Evenson KR, Ammerman AS  **Year:** 2009  **Citation:** Women & Health 49(2-3): 164-180  **Country of study:** US  **Aim of study:** Perceptions of the community food environment and related influences on food choice among midlife women residing in rural and urban areas.  **Study design:** Qualitative - Interview study  **Quality score: (++, + or -):** ++ |
| --- |
| **Population and setting** |
| Women in North Carolina (n= 28) from rural and urban areas aged 37-67 years.  Midlife defined as 40-64 years but two participants were slightly out with this range. 19 participants were black and 9 were white. 15 lived in urban areas and 13 in rural areas. Recruitment was through patrons of community centres. |
| **Study design** |
| Semi-structured interviews to understand women's perceptions of the food environment, including community barriers and resources. Participants were paid $25 after completing the interview. |
| **Outcomes and methods of analysis** |
| Thematic analysis with 26 nutrition themes identified. Transcripts coded independently by two coders, discrepancies were discussed to reach consensus. Mon themes. |
| **Results** |
| - Workplace food choices were affected by the social environment (co-workers), personal health concerns and the surrounding food environment. - There were perceived differences between urban and rural environments with rural areas having fewer supermarkets and fast food restaurants compared to urban areas, which had fewer produce stands. - Food chosen at home was primarily influenced by family members, health concerns and convenient food sources. |
| **Notes by review team** |
|  |

| **Authors:** Vue H, Degeneffe D, Reicks M  **Year:** 2008  **Citation:** Journal of Nutrition Education and Behavior 40(6): 378-84  **Country of study:** US  **Aim of study:** Need states based on eating occasions experienced by midlife women.  **Study design:** Qualitative - focus group study  **Quality score: (++, + or -):** + |
| --- |
| **Population and setting** |
| Multi-ethnic women (n=34), mean age 46 years (target age range 35-55 years).  Participants were recruited from fliers posted at a large metropolitan university in the Mid-west. Participants received $45. |
| **Study design** |
| Series of seven focus group interviews using an interview guide developed by researchers with expertise in marketing research and nutrition. |
| **Outcomes and methods of analysis** |
| Transcripts coded independently by three researchers, discrepancies were discussed to reach consensus. Analysis for common themes. Focus group findings were used to develop a hypothetical framework for describing the full range of eating occasions experienced by midlife women. |
| **Results** |
| - Eight need states were identified: mindless pastime, socialising, habitual, low effort, pursuing health, soothing, nurturing, social/celebratory. - Need states with a low level of emotional gratification were dominated by sets of functional needs, such as coping with stress, meeting external demands of time and effort and maintaining a routine. - Food was a means of reinforcing family identity, social expression, and celebration in need states with high levels of emotional gratification. |
| **Notes by review team** |
|  |

## C1.5 Alcohol

| **Authors:** Pettinato M  **Year:** 2008  **Citation:** Issues in Mental Health Nursing 29(6): 619-638  **Country of study:** US  **Aim of study:** Life experience of the misuse of alcohol among midlife and older lesbians.  **Study design:** Qualitative - Interview study  **Quality score: (++, + or -):** + |
| --- |
| **Population and setting** |
| Midlife and older lesbians (n=13) from the Northwest United States.  Age range: 43 to 63 years (mean age 49 years).  All of the women were in various stages of recovery from alcohol addiction except for one who was still misusing alcohol. Most of the women described themselves as totally or partially Caucasian, two were of mixed Native American heritage and another described her mixed ethnicity as including Japanese. |
| **Study design** |
| Interviews. |
| **Outcomes and methods of analysis** |
| Grounded theory methodology. The theory produced is grounded in the specific data created by inductively originated concepts from the interviewees. |
| **Results** |
| - One major theme about the use of alcohol was 'disconnecting from authentic self'. The most dominating disconnect for the majority of the women was a disconnection from their identity as a lesbian. - They also experienced disconnection from their other identities or roles such as student, wife, business woman and mother or from childhood issues/family of origin issues in childhood. - The authors concluded that interventions in this population may benefit from more empathic attempts to help lesbians to connect or reconnect with their authentic selves. |
| **Notes by review team** |
|  |

## C1.6 Eye Care

| **Authors:** Gower EW, Silverman E, Cassard SD, Williams SK, Baldonado K, Friedman DS  **Year:** 2013  **Citation:** Journal of Health Care for the Poor & Underserved 24(3): 1042-1052  **Country of study:** US  **Aim of study:** Barriers to attending an eye examination after vision screening referral within a vulnerable population.  **Study design:** Qualitative - Interview study  **Quality score: (++, + or -):** + |
| --- |
| **Population and setting** |
| Uninsured or underinsured patients (n=91) who attended vision screening and were referred for an eye examination but did not attend that exam. Mean age 48 years. |
| **Study design** |
| Telephone-based questionnaires administered by trained interviewers. Participants were asked if they were interested in attending a free eye exam. Two possible scripts used depending on whether answer was yes or no. |
| **Outcomes and methods of analysis** |
| Open-ended responses were reviewed and categorised by a single interviewer. Categories created to group responses. |
| **Results** |
| - Primary reasons for missing appointments were forgetting (34%), lacking transportation (36%) and scheduling conflicts (26%). - 24% said they could not afford transportation. - Authors concluded that transportation is a key barrier to eye care services in this disadvantaged population. Current eye care delivery can be improved by addressing these barriers to attendance. |
| **Notes by review team** |
|  |

## C1.7 Health and health behaviours in general: Women

| **Authors:** Smith-Dijulio K, Windsor C, Anderson D  **Year:** 2010  **Citation:** Qualitative Health Research 20(7): 966-976  **Country of study:** US  **Aim of study:** The shaping of midlife women's views of health and health behaviours  **Study design:** Interview study  **Quality score: (++, + or -):** + |
| --- |
| **Population and setting** |
| Women (n=23) who had participated in a women's wellness intervention trial as part of either the intervention or control group.  Age range 57-66, mean age 61.  All were white and the majority were married or with a partner, retired. |
| **Study design** |
| Interviews. Initial interview questions focused on types of changes in eating and exercise behaviours the women had or had not made and facilitators or barriers to change (or lack of change). |
| **Outcomes and methods of analysis** |
| Inductive analytical process with the goal to answer how and why participants constructed meanings about health and health behaviours in general, as well as in specific situations. |
| **Results** |
| - The extent to which women adhered to socially dominant gender roles appeared to affect their capacity to engage in healthy behaviours as they defined them. There was an expectation that one puts the needs of others first, only being 'allowed' to take care of self after having taken care of others. - It was difficult to sustain healthy practices if one’s partner did not, and living with someone who was supportive was important. - Women who maintained a strong sense of personal power were more able to choose desired behaviours. - Enjoyment of physical activity was a more important facilitator than doing it because the doctor told them to. - Midlife was associated with guilt at not doing enough to be healthy. |
| **Notes by review team** |
|  |

| **Authors:** Meadows LM, Thurston WE, Berenson CA  **Year:** 2001  **Citation:** Qualitative Health Research 11(4): 450-463  **Country of study:** Canada  **Aim of study:** Health promotion and preventive measures: Interpreting messages at midlife  **Study design:** Qualitative - Interview study  **Quality score: (++, + or -):** ++ |
| --- |
| **Population and setting** |
| Rural midlife women (n=24), age 40 to 65 years in the province of Alberta. |
| **Study design** |
| Interviews. Focus was on providing new information on women's experiences around preventive health and health care use in the context of popular and professional health sectors. Questions addressed during the interview included definitions of health and current health issues, use of health service, responsibilities, family roles and health behaviours. |
| **Outcomes and methods of analysis** |
| Ethnography and grounded theory approaches.  Thematic analysis. |
| **Results** |
| - There were geographical, time and financial barriers accessing the healthcare system from a rural residence. Key themes were being busy and time constraints - finding it difficult to find time for themselves, especially when it came to their health. Roles included caring for homes, jobs, volunteering, helping adult children and grandchildren, caring for parents, attending church, leisure activities and hobbies, friends and family. - Women reported also that their physicians were very busy and overworked so they did not want to bother their doctor with anything. - Most of the women reported going for regular, quick annual check-ups. - Women seeking healthcare often reported dismissive statements from healthcare professionals and prevented them seeking preventive health care. Women spent a lot of time analysing their own symptoms to decide if it was worth seeing a professional before getting to that point. - Family history of disease was important when weighing the pros and cons of preventive health care. |
| **Notes by review team** |
|  |

| **Authors:** Enjezab B, Farajzadegan Z, Taleghani F, Aflatoonian A  **Year:** 2012  **Citation:** Iranian Journal of Nursing and Midwifery Research 17(5): 390-398  **Country of study:**Iran  **Aim of study:** Internal motivations and barriers effective on the healthy lifestyle of middle-aged women: A qualitative approach.  **Study design:**  **Quality score: (++, + or -):** + |
| --- |
| **Population and setting** |
| Middle-aged women (n=21), aged 40-60 in Iran in a city with a strong religious and traditional culture. |
| **Study design** |
| In-depth interviews |
| **Outcomes and methods of analysis** |
| Thematic analysis |
| **Results** |
| Five main themes relating to the women’s internal barriers and motivation for health behaviours:   1. Women's knowledge of health-promoting behaviours 2. Importance of health and healthy behaviour for women 3. Affliction or fear of affliction of chronic disease 4. Responsibilities of women in the family and society 5. Skills of life management by women |
| **Notes by review team** |
|  |

**Primary cohort studies**

## C2.1 Physical Activity: Men and women

| **Authors:** Wurm S, Tomasik MJ, Tesch-Romer C  **Year:** 2008  **Citation:** Psychology & Health 25(1): 25-42  **Country of study:** Germany  **Aim of study:** Effect of a positive view on ageing on physical exercise among middle-aged and older adults  **Study design:** Cohort study  **Quality score: (++, + or -):** ++ |
| --- |
| **Population and setting** |
| **Setting:** Data based on the German Ageing Survey, a longitudinal population based survey on middle-aged and older adults conducted in 1996 and 2002.  **Sample characteristics:** Only data for middle-aged adults is reported here. The baseline sample (age 40 to 85 years) was selected using a national probability sampling technique with stratified sampling by age, gender and place of residence (Eastern or Western Germany). 50% of those contacted agreed to an interview and 83.4% of those additionally completed a questionnaire. Middle-aged adults (n=956 longitudinal analysis) were defined as 40-64 years. Mean age of the middle-aged adults was 52.0. (SD 7.0), 48.4% female, 63.5% from Western Germany, education score 1.70 (SD 0.78) from a range of 1(low)-3(high). |
| **Study design** |
| Level of positive view of ageing (PVA) was measured using a scale on the ageing-related cognition of ongoing development. The scale refers to the view of ageing as a time of personal growth and development and was assessed by the four items:   1. "Ageing means to me that I continue to make plans" 2. "Ageing means to me that my capabilities are increasing" 3. "Ageing means to me that I can still learn new things" 4. "Ageing means to me that I can still put my ideas into practice".   Participants could rate the items on a four-point scale ranging from 'definitely false' to definitely true'. |
| **Outcomes and methods of analysis** |
| Frequency of walking and doing sports were measured. Level of sports activity and walking measured on a 6 point scaling ranging from 'never' to 'daily'. Longitudinal analysis was conducted using path models using physical exercise at T2 as dependent variable while controlling for physical exercise at T1. Additionally multi-group models controlling for baseline health, hope, SES and Age were conducted. |
| **Results** |
| - There was stability over time (six years follow-up) in level of sports activity in middle-aged adults (r=0.44, p<0.001). - There was no lower decrease in sporting activity in models adjusted for physical exercise at baseline and in multivariate models or walking over time in those middle-aged adults with PVA . - PVA was not related to walking in middle-aged adults (Beta 0.00, p=0.92), however, middle-aged adults with high PVA increased their sporting activity provided they were healthy enough to do so in longitudinal analyses (Beta 0.05, p=0.07) models adjusted for PA at baseline. - Overall the effects on PA of PVA were small. |
| **Notes by review team** |
| **The authors report limitations of the study:** 1) Data are based on self-reports and therefore might be biased.  2) Sporting activity and walking were measured with single-item questions only.   1. A short-term recall method was used for the frequency of physical exercise. 2. There was some bias in the longitudinal sample due to attrition which meant the longitudinal sample was selected in favour of healthier and better-educated people who exercised more often. |

## C2.2 Physical Activity: Men

| **Authors:** Sorensen L  **Year:** 2005  **Citation:** Occupational Medicine 55(2): 136-138  **Country of study:** Finland  **Aim of study:** Correlates of physical activity among middle-aged Finnish male police officers  **Study design:** Cohort study  **Quality score: (++, + or -):** + |
| --- |
| **Population and setting** |
| **Setting:** Finland.  Participants were middle-aged male police officers (n=96) who were followed up for 15 years from 1981 to 1996. 62% of the police officers were physically active in their leisure time at least twice a week. |
| **Study design** |
| Frequency of and adherence to leisure time PA was assessed at baseline and follow-up. |
| **Outcomes and methods of analysis** |
| **Outcomes:** Physical activity and physical fitness were assessed by a physical activity scale questionnaire and submaximal bicycle ergometer test. Factors affecting adherence to PA were assessed by the PRECEDE-PROCEED model (referenced in paper) that assesses predisposing, enabling and reinforcing factors. Factors assessed were 'enjoyment' - reinforcing, 'lack of skills' - enabling and 'lack of knowledge’ - predisposing.  **Follow-ups:** 15 years |
| **Results** |
| - The factor 'enjoyment' was the most powerful determinant for both physical activity and fitness. 'Enjoyment' was also the only factor with a significant association with physical fitness, so the authors concluded that a certain degree of physical fitness is required before a person enjoys physical activity. - The three factors assessed had no significant correlation with each other so influenced PA independently. - Leisure time PA in 1981 correlated significantly with leisure time PA in 1996, so physical activity in early adulthood also in part predicts PA in middle-age. |
| **Notes by review team** |
| Only 96 of the 103 men recruited at baseline participated at follow-up. The authors concluded there may be a 'healthy survivor' effect so the results may overestimate the level of PA and fitness in middle-age. |

## C2.3 Physical Activity: Women

| **Authors:** Segar ML, Eccles JS, Richardson CR.  **Year:** 2008  **Citation:** Women’s Health Issues 18(4): 281-291  **Country of study:** US  **Aim of study:** To investigate the effects of PA goals on PA participation.  **Study design:** Cohort study  **Quality score: (++, + or -):** + |
| --- |
| **Population and setting** |
| **Setting:** US, university.  Participants were female university employees (n=156 at baseline) aged between 40 and 60 (mean age 49.3 (SD 5.3), working in full-time clerical jobs with internet and email access. Recruitment was via a random sample selected to participate in a mailed survey. Those who self-reported a chronic health condition or illness were excluded.  Mean BMI was 27.2 (SD 5.3); 88% were European American, 5.7% African American, 1.3% Asian, 1.9% Latina. |
| **Study design** |
| Physical activity participation and physical activity goals. |
| **Outcomes and methods of analysis** |
| PA participation was measured using a modified version of the Godin leisure time exercise questionnaire (GLTQ, 1985). Participants were asked how many times per week and minutes per session they participate in PA. To measure PA goals, participants were asked to select their three most important goals from a list of 18 reasons compiled from a comprehensive literature review. Cluster analysis was then used to identify homogenous groupings of the ranked goals. The methods used to identify the goal cluster had previously been reported and validated.  **Follow-ups:** one month (97% retention) and one year (87% retention); one year post-baseline. A linear mixed model fitted to the data to investigate the effects of PA goals on PA participation, controlling for BMI and social support. |
| **Results** |
| - Five goal clusters identified:  1. Health benefits; 2. Weight loss 3. Stress reduction 4. Sense of wellbeing 5. Weight maintenance/toning  - Participants with Weight loss (mean PA score 27.4 (SD 2.4) and Health benefits (27.7 (SD 1.7) goals participated in significantly less PA than those with Sense of wellbeing (36.7 (3.1) and Stress reduction (35.0, SD 2.8) goals. - The authors concluded that long-term participation among healthy women in PA might be more effective if programmes emphasise the goal of PA to enhance enjoyment and quality of life rather than to decrease weight or benefit health. |
| **Notes by review team** |
|  |

## C2.4 Diet

| **Authors:** Yates BC, Pullen CH, Santo JB, Boeckner L, Hageman PA, Dizona PJ, Walker SN  **Year:** 2012  **Citation:** Social Science & Medicine 75: 659-667  **Country of study:** US  **Aim of study:** To examine predictors of change over time in healthy eating behaviours in mid-life and older women in response to a one year health-promoting intervention  **Study design:** Cohort study (for predictors of change in eating behaviours, part of an intervention study  **Quality score: (++, + or -):** + |
| --- |
| **Population and setting** |
| **Setting:** Data for this secondary analysis were from the Wellness for Women community-based trial. Women (n=225) between the ages of 50-69 (mean age 58, SD 5.5) were recruited in rural Nebraska. Specific demographic characteristics not reported in this paper, but the authors reported that on average they were Caucasian, married, employed outside the home, had attended college, had an average BMI of 30 kg/m2 and had annual incomes > $20,000. |
| **Study design** |
| Perceived benefits and barriers to healthy eating were measured by selected items from the HEBBS (Healthy eating benefits and barriers scales), that used a four-point response range for benefits and five points for barriers. Self-efficacy for healthy eating was measured by selected items from the self-efficacy for healthy eating habits scale. Family support for healthy eating was measured by the Family Support for Healthy Eating habits scale that measure positive encouragement for healthy eating among family members (four items on a five-point Likert scale). Geographic areas were randomised to intervention (tailored newsletter with personal goals assessment of benefits, barriers, self-efficacy, and support) or standard newsletter. |
| **Outcomes and methods of analysis** |
| A food frequency questionnaire was used to measure healthy eating behaviour and provide estimates of the nutrients and dietary constituents. The healthy eating index (HEI) was used to create a composite score of diet quality. Latent growth curve modelling was used to model change in diet. |
| **Results** |
| - Perceived barriers had the strongest impact on eating behaviour during all time points. - Compared to participants in the standard newsletter group, those in the tailored newsletter group perceived more family support (p-0.289, beta 0.366, z=2.40) and fewer barriers to healthy eating at the end of the intervention (b=0.140, Beta 0.369, z 2.42, p<0.05). - Authors recommend that both family support and perceived barriers should be central components of interventions focused on healthy eating behaviour in rural midlife and older women. |
| **Notes by review team** |
|  |

| **Authors:** Méjean C, Macouillard P, Castetbon K, Kesse-Guyot E, Hercberg S.  **Year:** 2011  **Citation:** British Journal of Nutrition 105(5): 776-786  **Country of study:** France  **Aim of study:** To determine sociodemographic, lifestyle and health characteristics associated with consumption of fatty-sweetened and fatty-salted foods in middle-aged French adults.  **Study design:** Cohort study  **Quality score: (++, + or -):** + |
| --- |
| **Population and setting** |
| **Setting:** Participants in the Supplementation en Vitamins Mineraux et Antioxidants cohort study, a large cohort of middle-aged adults recruited throughout mainland France. A total of 7876 women aged 35-60 years and 5141 men aged 45-60 years were included at baseline in 1994-5. Participants were volunteers recruited through the media. |
| **Study design** |
| Methods for assessment of demographic, socio-economic, lifestyle and health self-administered questionnaire. The official French classification was used to classify subjects into occupational categories according to their self-reported occupation or most recent employment if they were retired or unemployed. Physical activity was assessed using a non-validated method via a single question. |
| **Outcomes and methods of analysis** |
| Dietary intake assessed using a minimum of six x 24 hour dietary records collected over a two-year period. Scoring was used to measure amount of fat-sweetened and fatty-salted food consumption. One point was attributed to one serving assessed from commonly used French portion sizes. Two year follow up. Logistic regression analysis was used to assess demographic, socio-economic, lifestyle and health factors related to elevated and intermediate consumption of fatty sweetened and fatty salted foods. Univariate and multivariate analyses conducted. All multivariate models were adjusted for energy intake and sex. |
| **Results** |
| - Risk of moderate or high consumption of fatty-salted foods decreased with increasing age. - Current smokers (OR 1.29, 95% CI 1.09, 1.53, P= 0.01), drinkers (OR 1.86, 95% CI 1.41, 2.45, p<0.0001), individuals with overweight (OR 1.57, 95% CI 1.32, 1.86, p<0.0001), and with hypertension (OR 1.33, 95% CI1.11, 1.60, p=0.002) were more likely to consume moderate or high amounts of fatty-salted foods. - Risk of moderate or high consumption of fatty sweetened foods decreased with increasing age. - Current smokers, drinkers, individuals with overweight and with hypertension were more likely to consume moderate or high amounts of fatty sweetened foods. |
| **Notes by review team** |
|  |

## C2.5 Smoking

| **Authors:** Honjo K, Iso H, Inoue M, Tsugane S  **Year:** 2010  **Citation:** Nicotine & Tobacco Research 12(10): 1050-1054  **Country of study:** Japan  **Aim of study:** To determine predictive factors for smoking cessation among middle-aged Japanese  **Study design:** Cohort study  **Quality score: (++, + or -):** - |
| --- |
| **Population and setting** |
| **Setting:** Participants were from the Japan Public Health Center-based Prospective Study (JPHC) Cohort 1 that was initiated in 1990 and recruited a cohort from four public health center areas. Participants were those members of the cohort that were smokers at baseline (n= 9887). Smokers for whom there was no information available on smoking status at follow up were excluded (n=363), so the total number of eligible participants for this study was 9524. |
| **Study design** |
| A baseline questionnaire was used to collect information on baseline age, gender, education level, occupation, marital status, number of cigarettes smoked per day, age of smoking initiation, passive smoking, perceived stress, leisure time physical activity, frequency of alcohol intake and participation in health check-ups in the previous year. |
| **Outcomes and methods of analysis** |
| Smoking cessation was identified by responses to the question on smoking status in the 10 year follow-up questionnaire. Ordinal logistic regression analysis used to calculate age-gender and area adjusted odds ratios for smoking cessation of each predictor (Model 1). Multivariate analyses also conducted adjusted for age, gender, education level, occupation, marital status, age of initiating smoking, number of cigarettes consumed per day, passive smoking, stress, physical activity frequency, frequency of alcohol intake obesity, health check-ups, prescribed drug use and development of diseases (Model 2). |
| **Results** |
| Significant predictors of smoking cessation for middle-aged Japanese men and women were:   - A white-collar job (multiv OR1.18 , 95% CI 1.05-1.32) - A small number of cigarettes smoked per day, older age at initiation of smoking (multiv OR 1.62, 95% CI 1.38-1.91) - Physical activity, participation in health check-ups, initiation of prescribed medicine use (multiv OR 1.92, 95% CI 1.72-2.14) - Diseases newly developed (multiv OR 1.21, 95% CI 1.08-1.36) during follow-up |
| **Notes by review team** |
|  |

## C2.6 Alcohol

| **Authors:** Caldwell TM, Rodgers B, Clark C, Jefferis BJMH, Stansfeld SA, Power C.  **Year:** 2008  **Citation:** Drug & Alcohol Dependence 95(3): 269-278  **Country of study:** UK  **Aim of study:** To determine life course socio-economic predictors of midlife drinking patterns  **Study design:** Cohort study  **Quality score: (++, + or -):** ++ |
| --- |
| **Population and setting** |
| **Setting:** Data was from the 1958 British Birth Cohort Study (n=9146). This included 98% of all births in England, Scotland and Wales. Only the mid-life data has been extracted: socio-economic conditions at age 42 as a predictor of drinking behaviour at age 45 years. |
| **Study design** |
| Socio-economic information included: manual socio-economic position, owner/buyer residential tenure, educational attainment. Occupational SEP was categorised using the British Registrar General Classifications. Residential tenure was assessed as owner/buyer vs non-owner/buyer. Educational attainment was measured using the highest qualification participants had completed by 33 years. |
| **Outcomes and methods of analysis** |
| At age 45 years the overlap between drinking patterns was explored using the alcohol use disorders identification test. Patterns included: 'Moderate-binge' drinkers with low-problem scores, consuming within UK sensible drinking guidelines); Low-Problem Heavy (LPH) drinkers; 'Problem' (and heavy or binge) and Non/occasional (</= monthly) drinkers. Logistic regression analysis was used to link socio-economic models at age 42 years with drinking at age 45 years. Follow-up 3 years. |
| **Results** |
| - Socio-economic disadvantage was consistently linked to moderate-binge, non-/occasional and problem but not LPH drinking across all analyses. - The highest risk was associated with multiple and persistent risk across childhood and adulthood which was partially accounted for by education. - For midlife exposure and outcomes, occupational SEP at age 42y was significantly associated with risk of binge drinking (OR 1.53, 95% CI 1.35-1.73, p<0.001; and problem drinking (OR 1.42, 95% CI 1.17, 1.72; p<0.001). - Residential tenure at age 42y was significantly associated with binge drinking (OR 1.59, 95% CI 1.37-1.84, p<0.001), heavy drinking (OR 1.29, 95% CI 1.11, 1.51, P<0.01) and problem drinking (OR 1.88, 95% CI 1.53, 2.31). |
| **Notes by review team** |
|  |

## C2.7 Health behaviours in general

| **Authors:** Benzies KM, Wangby M, Bergman LR  **Year:** 2008  **Citation:** Health Care for Women International 29(10): 997-1018  **Country of study:** Sweden  **Aim of study:** To measure factors that predict change in health-related behaviours among midlife Swedish women  **Study design:** Cohort study  **Quality score: (++, + or -):** + |
| --- |
| **Population and setting** |
| **Setting:** Longitudinal programme on individual development and adaptation in central Sweden.  The original cohort was an entire school cohort that was in grade three in 1964/1965, majority born in 1955. For this paper, participants were studied at age 43 and followed up four years later. Of 682 women in the IDA main group recruited in 1964/5, 639 were still available for this 1998 data collection. Of those eligible, 569 (89%) participated. At follow-up four years later, 512 returned completed questionnaires and 349 participated in a medical examination from which the data was used to examine stability of health behaviours over time. |
| **Study design** |
| Eating habits, exercise, alcohol, smoking, medical surveillance. |
| **Outcomes and methods of analysis** |
| Questionnaires were specifically designed for the study and included five questions about eating habits (dairy products, low-fat meat, high fibre bread, snacks, eating healthy) and one about exercise, two questions on alcohol consumption (how much/often) and two on smoking (how much/often), three questions about medical surveillance (breast self-exam, mammography and cervical screening). Longitudinal correlations between specific HRBs and indexes in 1998 and 2002 were examined. Regression analysis was used to determine factors that contribute to change in health-related behaviours. |
| **Results** |
| - There was a high degree of stability for many health behaviours with longitudinal correlations ranging from 0.40 (cervical screening) to 0.87 (smoking frequency). - The specific behaviours with the highest degree of stability over the four-year follow-up were smoking (0.80) and alcohol consumption (0.70). - For the regression analyses, the specific behaviour in 1998 was the strongest predictor of the same behaviour four years later. Variables that predicted a positive change in specific behaviours were self-rated health in 1998 (Beta 0.16, p<0.01), self-rated change in lifestyle factors in 2002 (Beta 0.13, p<0.01), marital status (Beta 0.15, p<0.01) and education level (Beta -0.09, p<0.01), having a child at home (Bets 0.11, p<0.01). |
| **Notes by review team** |
|  |

| **Authors:** King DE, Mainous AG 3rd, Geesey ME  **Year:** 2007  **Citation:** American Journal of Medicine 120(7): 598-603  **Country of study:** US  **Aim of study:** To determine factors related to adopting a healthy lifestyle in a middle-aged cohort.  **Study design:** Cohort study  **Quality score: (++, + or -):** ++ |
| --- |
| **Population and setting** |
| **Setting:** Participants were adults (n=15708) aged 45-64 in the Atherosclerosis Risk in Communities Survey. Enrolment was from four communities in the US |
| **Study design** |
| Demographic variables collected included age, race, gender, education and family income, all self-reported at baseline (1987-1989). |
| **Outcomes and methods of analysis** |
| A healthy lifestyle was characterised by having all four of the following lifestyle characteristics: eating at least five fruits and vegetables daily, exercising a minimum of 2.5 hours per week; BMI between 18.5 and 30 kg/m2 and not smoking.  **Follow-up:** Six years |
| **Results** |
| - Of 15708 participants, 1344 (8.5%) had four healthy lifestyle habits at the first visit and 970 (8.4%) of the remainder had newly adopted a healthy lifestyle six years later. - In the logistic regression model predicting which individuals would switch to an overall healthy lifestyle, individuals who are older, female with a college education, with family incomes >$35,000 (1994) or with no history of hypertension were more likely to have switched than others. - Men, African Americans, individuals with lower socio-economic status or a history of hypertension or diabetes were less likely to adopt a healthy lifestyle (p<0.05). |
| **Notes by review team** |
|  |

| **Authors:** Petersson U, Ostgren CJ, Brudin L, Ovhed I, Nilsson PM  **Year:** 2008  **Citation:** Scandinavian Journal of Public Health 36(4): 389-396  **Country of study:** Sweden  **Aim of study:** To determine predictors of successful self-reported lifestyle changes in a defined middle-aged population  **Study design:** Cohort study  **Quality score: (++, + or -):** + |
| --- |
| **Population and setting** |
| **Setting:** The cohort was part of the Soderakra Cardiovascular Risk Factor Study in a community located on the south-eastern coast of Sweden with a predominantly rural population.  All subjects born 1931-1950, then aged 40-59 years, were invited to a screening study for CVD risk factors. In total 782 participants invited: 705 (90%) agreed to participate - 361 males/344 females. |
| **Study design** |
| At baseline, blood glucose, serum cholesterol, HDL cholesterol and serum triglycerides were analysed. Anthropomentric measurements were taken, blood pressure recorded and a questionnaire on lifestyle habits was completed focusing on fat intake, physical activity, smoking and alcohol consumption. |
| **Outcomes and methods of analysis** |
| A 10-year follow-up study was conducted as part of a structured telephone interview, conducted by a specially trained nurse. Of the original 705 participants in the baseline study cohort, 306 men and 323 women completed the follow-up telephone interview. Questions focused on changes in lifestyle habits - overweight, smoking, fat consumption, physical activity and alcohol use. To study predictors for success, univariate and multivariate analyses were done using logistic regression. All significant variables for success of lifestyle changes in the univariate analysis were further examined in multivariate analyses by stepwise adjustment for serum lipids, anthropometric data, blood pressure and smoking. The analyses also included marital status, SES, educational level, previous CVD risk factors and family history. |
| **Results** |
| - In multivariate analyses, female gender (OR 1.56, 95% CI 1.11-2.18) was associated with significant improvements in self-reported lifestyle changes. - Significant predictors of success in men were prevalent CVD risk conditions (OR 4.77, 95% CI 2.18-10.5, p<0.001) and previous myocardial infarction (OR =22.8, 95% CI 4.73-110, p<0.001). - For women, elevated blood pressure at baseline was associated with successful lifestyle changes (OR 1.84, 95% CI 1.12-3.02), p=0.016). - Smoking at baseline was also associated with successful lifestyle change in both men (OR 3.36, 95% CI 2.05-5.51, p<0.001) and women (1.81, 95% CI 1.11-2.95; P=0.017). |
| **Notes by review team** |
|  |

| **Authors:** Shi HJ, Nakamura K, Takano T  **Year:** 2004  **Citation:** Preventive Medicine 39(6): 1164-1171  **Country of study:** Japan  **Aim of study:** Health values and health-information seeking in relation to positive change of health practice among middle-aged urban men  **Study design:** Cohort study  **Quality score: (++, + or -):** - |
| --- |
| **Population and setting** |
| **Setting:** Middle aged men (n=334), aged 45-49, were selected from the central area of Tokyo by using multistage random strategy from resident registration records in 1998, follow up was conducted in 2001. Education, occupation, working hours, perceived health status and blood pressure and health practices (except diet) were similar among respondents and non-respondents to the follow up interview. Respondents were more likely to eat meals regularly, avoid excessive salt intake and stop eating when 80% full. |
| **Study design** |
| Interviews at baseline and follow up were conducted using standardised questionnaires by trained health survey specialists during face to face interviews at each person’s home. Four aspects of health behaviour - diet, physical exercise, smoking and stress control - during the preceding three months were assessed in both interviews, following the methods of general health survey by the Ministry of health, Welfare and Labour. |
| **Outcomes and methods of analysis** |
|  |
| **Results** |
| - Percentages of subjects who engaged in eight identified unhealthy behaviours ranged from 31.7% to 54.5% at baseline. At follow-up after three years the range was 5.7 to 33.6%. - A high value placed on health was independently associated with positive change of general health practice (OR 2.95, 95% CI 1.23 to 7.08) and was inversely associated with negative change (OR 0.45, 95% CI 0.18 to 1.10); consciously seeking health information was positively associated with positive change (OR=2.16, 95% CI= 1.07 to 4.36) after controlling for socio-economic and health status. - The authors conclude that health values saliency, sensitively designed health information and health status perception as well as SES should be considered for successful promotion of healthy lifestyle among adult males in Japan. |
| **Notes by review team** |
| The authors concluded that the results may be a little more generalisable among a health conscious and affluent population. Respondents were more likely to have healthy eating practices. |

## C2.8 Overweight

| **Authors:** Teixeira PJ, Going SB, Houtkooper LB, Cussler EC, Martin CJ, Metcalfe LL… Lohman TG  **Year:** 2002  **Citation:** Journal of Behavioral Medicine 25(6): 499-523  **Country of study:** US  **Aim of study:** To examine psychosocial predictors of success for behavioural weight reduction  **Study design:** Cohort study  **Quality score: (++, + or -):** + |
| --- |
| **Population and setting** |
| **Setting:** Participants were overweight and obese middle-aged women (n=112) aged 40-55 years with BMI between 25.0 and 38.0 kg/m2. Mean age 47.8 years, BMI 31.4+/- 3.9 kg/m2.  Participants were non-smokers and free of major illnesses, 10% were Hispanic, 86% were non-Hispanic white. Recruitment was from the community through newspaper and TV adverts. Of 466 women who enquired about the study 35% (168 people) met all inclusion criteria. These women attended an orientation session and 142 volunteered for the study. During the baseline run in phase a further 26 were excluded because of non-compliance with study requirements and four dropped out voluntarily. 89 women completed the study and provided follow-up data. |
| **Study design** |
| A comprehensive psychometric battery was administered before a six-month behavioural weight reduction programme to identify baseline characteristics of successful and unsuccessful participants. This covered several areas considered to be relevant for weight management, including eating, exercise, body image, quality of life, weight/dieting history, weight outcome evaluations/expectations and psychological measures (mood, self-esteem, self-motivation) variables. Previously validated instruments available in the literature were used. |
| **Outcomes and methods of analysis** |
| Associations between the psychosocial and historical variables assessed at baseline with changes in outcomes after the 16 week intervention programme were analysed. The intervention involved a meeting with the intervention team once a week, for 150 min per session, over 16 consecutive weeks. Multiple regression analysis was performed to assess the relationships among independent variables and the amount of variance in weight loss that could be predicted. |
| **Results** |
| Baseline psychosocial measures that showed statistically significant correlations (Spearman) with change in weight were:   - Weight/diet history - at least 10lb weight loss in the past two years (r=0.22, p<0.05); - Number of diets in past years (r= 0.37, p<0.001); - Self-motivation (r= -0.28, p< 0.01).   These factors were all associated with less weight loss after the intervention. |
| **Notes by review team** |
|  |
