# Supplementary material for: Barriers and Facilitators to the Uptake and Maintenance of Healthy Behaviours by People at Mid-Life: A Rapid Systematic Review
Source: PLoS One. 2016 Jan 27;11(1):e0145074. doi: 10.1371/journal.pone.0145074 (PMC4731386; doi:10.1371/journal.pone.0145074)
Supplement: S1 Text — (DOCX) [file pone.0145074.s005.docx]

**Search Strategies**

**1. Sample search strategy used to identify systematic reviews**

Sample search: Ovid MEDLINE(R) In-Process & Other Non-Indexed Citations and Ovid MEDLINE(R) <1946 to Present>

Note: Searches terms were modified where necessary when searching other databases.

--------------------------------------------------------------------------------

1 (prevent* or barrier* or facilitat* or hinder* or block* or obstacle* or restrict* or restrain* or obstruct* or inhibit* or impede* or delay* or constrain* or hindrance* or uptake or "take up" or increas* or decreas* or reduc* or impact* or effect* or improve* or enhance* or encourag* or support* or promot* or optimiz* or optimis* or adher* or access* or motivat* or accept* or satisfaction or compliance or comply or complie* or refus* or availabl* or provision* or provid* or offer or incentive* or utiliz* or utilis*).ti,ab. (11817672)

2 ((health* adj3 (behavior* or behaviour*))

or ((ageing or aging) adj3 (well or success* or positive* or active* or healthy))
or (food* adj3 choice*) or dieting or (diet* adj3 (health* or balance* or fat* or salt* or sugar* or mediterranean or choice* or improv* or unhealthy))
or ((fruit* or vegetable* or salt* or fat* or sugar*) adj3 (intake* or consum* or eat* or ate))
or (undernutrition or undernourish* or under-nutrition* or under-nourish*)
or (multimicronutrient* or multi-micronutrient* or micronutrient* or micro-nutrient* or multinutrient* or multi-nutrient*)
or ("five a day" or "5 a day")
or ("health check" or "check-up")
or "health MOT*"
or ((eye* or sight* or vision* or visual* or hearing) adj3 (test* or check* or screen*))
or (smok* or tobacco or cigar* or nicotine)
or ((alcohol* or drunk* or drink*) adj3 (consum* or misuse* or abuse* or intoxicat* or harmful or excess* or binge* or hazardous* or heavy or temperance or abstinence))
or temperan*
or teetotal*
or (lonely or lonli*)
or (socialis* or socializ*)
or (social* adj3 (isolat* or network* or contac* or alien*))
or (cognitive adj2 stimulat*)
or (sedentary or exercis* or sport*)
or "physical condition*"
or (balance* and (exercis* or retrain* or re-train* or reeducat* or re-educat*))
or inactiv*
or (walk* or run* or jog* or swim* or danc* or garden* or cycl* or bicycl* or bike* or recreation*)
or ("resistance training" or "acquatic exercis*" or "wellness centre*" or "wellness center*")
or ("weight gain*" or "weight los*" or "overweight" or "over weight") or (obesity and "related behavio*")
or (overeat* or "over eat")
or (waist* adj3 (circumference* or measur*))
or ((bmi or "body mass index") adj3 (gain* or loss* or lose* or lost or change*))
or (weight adj2 (cycling or reduc* or los* or maint* or decreas* or increas* or watch* or control*))
or "weight change*"
or ((behavio?r* or lifestyle or "life style") adj3 (change* or changing or modification or modify or modifying or therapy or therapies or program* or intervention* or counsel*))
or ((physical* or keep* or cardio* or aerobic or fitness) adj3 (fit* or activ* or train*))
or ((physical* or game* or leisure* or fitness) adj5 (event* or setting* or sector* or program* or venue* or site* or center* or centre*))
adj3
(prevent* or barrier* or facilitat* or hinder* or block* or obstacle* or restrict* or restrain* or obstruct* or inhibit* or impede* or delay* or constrain* or hindrance* or uptake or "take up" or increas* or decreas* or reduc* or impact* or effect* or improve* or enhance* or encourag* or support* or promot* or optimiz* or optimis* or adher* or access* or motivat* or accept* or satisfaction or compliance or comply or complie* or refus* or availabl* or provision* or provid* or offer or incentive* or utiliz* or utilis*)).ti,ab. (11815615)

3 exp health behavior/ (99406)

4 exp risk reduction behavior/ (7429)

5 exp health promotion/ (54839)

6 exp primary prevention/ (114054)

7 exp preventive medicine/ (32336)

8 exp life style/ (64645)

9 exp food habits/ (21115)

10 exp food preferences/ (10035)

11 exp nutrition therapy/ (80294)

12 exp vision tests/ (80635)

13 exp hearing tests/ (37970)

14 exp smoking/ (124015)

15 exp smoking cessation/ (20976)

16 exp "tobacco use disorder"/ (8399)

17 exp "tobacco use cessation"/ (21675)

18 exp tobacco smoke pollution/ (10679)

19 exp alcohol drinking/ (52735)

20 exp alcohol deterrents/ (4190)

21 exp drinking behavior/ (58207)

22 exp temperance/ (2609)

23 exp loneliness/ (2167)

24 exp exercise/ (111288)

25 exp sports/ (110890)

26 exp exercise therapy/ (29819)

27 exp physical exertion/ (52630)

28 exp physical fitness/ (21873)

29 exp "physical education and training"/ (13326)

30 exp exercise test/ (50193)

31 exp walking/ (19952)

32 exp running/ (13374)

33 exp jogging/ (690)

34 exp bicycling/ (7564)

35 exp swimming/ (18600)

36 exp dancing/ (1824)

37 exp gardening/ (462)

38 exp fitness centers/ (336)

39 exp sedentary lifestyle/ (2461)

40 or/3-39 (982650)

41 1 and 40 (624194)

42 2 or 41 (927623)

43 meta-analysis as topic/ (14016)

44 meta-analys*.tw. (61279)

45 metaanaly*.tw. (1363)

46 Meta-Analysis/ (50578)

47 (systematic adj (review*1 or overview*1)).tw. (52517)

48 exp Review Literature as Topic/ (7590)

49 or/43-48 (123666)

50 Comment/ (570485)

51 Letter/ (823596)

52 Editorial/ (347012)

53 animal/ (5460677)

54 human/ (13571801)

55 53 not (53 and 54) (3939518)

56 50 or 51 or 52 or 55 (5192193)

57 49 not 56 (115029)

58 exp middle age/ (3327213)

59 (middle adj age*).ti,ab. (33553)

60 (baby adj2 boomer*).ti,ab. (755)

61 (midlife or "mid life" or midlives or "mid lives").ti,ab. (3816)

62 or/58-61 (3339411)

63 adult*.ti,ab. (820544)

64 exp Young Adult/ (345598)

65 exp Adult/ (5579820)

66 or/63-65 (5994172)

67 "single parent*".ti,ab. (1849)

68 minorit*.ti,ab. (44436)

69 "free school meal*".ti,ab. (51)

70 ((low* or work*) adj4 class*).ti,ab. (18291)

71 unemployed*.ti,ab. (5351)

72 (low* adj3 (income* or wage* or pay*)).ti,ab. (28835)

73 ("income support*" or "housing benefit*" or "child support*" or "unemployment benefit*").ti,ab. (537)

74 poverty.ti,ab. (15360)

75 (deprive* or deprivation*).ti,ab. (63372)

76 ethnic*.ti,ab. (88627)

77 ((vulnerable or disadvantaged or "at risk" or "high risk" or "low socioeconomic status" or neglect* or affected or marginal* or forgotten or non-associative or nonassociative or unengaged or hidden or excluded or transient or inaccessible or underserved or stigma* or inequitable) and (people or population* or communit* or neighbourhood*1 or neighborhood*1 or group* or area*1 or demograph* or patient* or social*)).ti,ab. (740630)

78 (immigrant* or migrant* or asylum or refugee* or undocumented).ti,ab. (33474)

79 (born adj2 overseas).ti,ab. (217)

80 (displaced and (people or person*1)).ti,ab. (904)

81 (homeless or vagrant*).ti,ab. (5618)

82 (((language* or communicat*) and (barrier* or understand* or strateg* or proficien*)) or translat* or interpret* or (cultur* and competen*)).ti,ab. (527336)

83 (illiteracy or illiterate*).ti,ab. (3622)

84 (traveller*1 or Gypsies or Gypsy or Gipsy or Gipsies or Romany or Romanies or Romani or Romanis or Romani or Romanis or Roma).ti,ab. (6115)

85 exp Poverty/ (31438)

86 exp Ethnic Groups/ or exp Minority Groups/ (121433)

87 exp Unemployment/ (5168)

88 exp Single Parent/ (1044)

89 exp Homeless Persons/ (6489)

90 (homeless* or vagrant* or tramp or tramps or "street person" or "street people" or (sleep* adj3 rough)).ti,ab. (7739)

91 exp "Emigration and Immigration"/ (23559)

92 exp "Emigrants and Immigrants"/ (5643)

93 exp refugees/ (6697)

94 exp Communication Barriers/ (4818)

95 Language/ (27562)

96 exp gypsies/ (617)

97 exp bisexuality/ or exp homosexuality/ or exp homosexuality, female/ or exp homosexuality, male/ (22600)

98 exp Transgendered Persons/ (103)

99 (lesbian* or gay* or homosexual* or bisexual* or transgender* or trans-gender* or trans-sexual* or transsexual* or transexual* or "men who have sex with men" or "same-sex" or queer*).ti,ab. (29659)

100 exp Transsexualism/ (2855)

101 exp Poverty Areas/ (4608)

102 exp Vulnerable populations/ (5723)

103 exp Social Stigma/ (1349)

104 exp shame/ (1506)

105 exp Prejudice/ (23558)

106 exp Socioeconomic Factors/ (341937)

107 or/67-106 (1865530)

108 107 and 66 (766030)

109 62 or 108 (3646065)

110 42 and 57 and 109 (2800)

111 42 and 57 and 109 (2800)

**112 limit 111 to yr="2000 -Current" (2582) SRs Midlife or disadvantaged adults healthy behavior barriers**

113 42 and 57 and 62 (1950)

**114 limit 113 to yr="2000 -Current" (1757) SRs of midlife healthy behavior barriers**

115 42 and 57 and 108 (1363)

116 42 and 57 and 108 (1363)

**117 limit 116 to yr="2000 -Current" (1299) SRs of disadvantaged adults’ healthy behavior barriers**

**118 42 and 109 (269040) Midlife or disadvantaged adults healthy behavior barriers**

119 118 not 110 (266240)

**120 limit 119 to yr="2000 -Current" (178577) non-SRs of Midlife or disadvantaged adults healthy behavior barriers**

***************************

**2. Sample search strategy used to identify primary studies**

Sample search: Ovid MEDLINE(R) In-Process & Other Non-Indexed Citations and Ovid MEDLINE(R) <1946 to Present>

Note: Searches terms were modified were necessary when searching other databases.

--------------------------------------------------------------------------------

1 (((health* adj3 (behavior* or behaviour*)) or ((ageing or aging) adj3 (well or success* or positive* or active* or healthy)) or (food* adj3 choice*) or dieting or (diet* adj3 (health* or balance* or fat* or salt* or sugar* or mediterranean or choice* or improv* or unhealthy)) or ((fruit* or vegetable* or salt* or fat* or sugar*) adj3 (intake* or consum* or eat* or ate)) or (undernutrition or undernourish* or under-nutrition* or under-nourish*) or (multimicronutrient* or multi-micronutrient* or micronutrient* or micro-nutrient* or multinutrient* or multi-nutrient*) or ("five a day" or "5 a day") or ("health check" or "check-up") or "health MOT*" or ((eye* or sight* or vision* or visual* or hearing) adj3 (test* or check* or screen*)) or (smok* or tobacco or cigar* or nicotine) or ((alcohol* or drunk* or drink*) adj3 (consum* or misuse* or abuse* or intoxicat* or harmful or excess* or binge* or hazardous* or heavy or temperance or abstinence)) or temperan* or teetotal* or (lonely or lonli*) or (socialis* or socializ*) or (social* adj3 (isolat* or network* or contac* or alien*)) or ((cognitive or mental*) adj2 stimulat*) or (sedentary or exercis* or sport*) or "physical condition*" or (balance* and (exercis* or retrain* or re-train* or reeducat* or re-educat*)) or inactiv* or (walk* or run* or jog* or swim* or danc* or garden* or cycl* or bicycl* or bike* or recreation*) or ("resistance training" or "acquatic exercis*" or "wellness centre*" or "wellness center*") or ("weight gain*" or "weight los*" or "overweight" or "over weight") or (obesity and "related behavio*") or (overeat* or "over eat") or (waist* adj3 (circumference* or measur*)) or ((bmi or "body mass index") adj3 (gain* or loss* or lose* or lost or change*)) or (weight adj2 (cycling or reduc* or los* or maint* or decreas* or increas* or watch* or control*)) or "weight change*" or ((behavio?r* or lifestyle or "life style") adj3 (change* or changing or modification or modify or modifying or therapy or therapies or program* or intervention* or counsel*)) or ((physical* or keep* or cardio* or aerobic or fitness) adj3 (fit* or activ* or train*)) or ((physical* or game* or leisure* or fitness) adj5 (event* or setting* or sector* or program* or venue* or site* or center* or centre*))) adj3 (prevent* or barrier* or facilitat* or hinder* or block* or obstacle* or restrict* or restrain* or obstruct* or inhibit* or impede* or delay* or constrain* or hindrance* or uptake or "take up" or increas* or decreas* or reduc* or impact* or effect* or improve* or enhance* or encourag* or support* or promot* or optimiz* or optimis* or adher* or access* or motivat* or accept* or satisfaction or compliance or comply or complie* or refus* or availabl* or provision* or provid* or offer or incentive* or utiliz* or utilis*)).ti,ab. (446597)

2 (prevent* or barrier* or facilitat* or hinder* or block* or obstacle* or restrict* or restrain* or obstruct* or inhibit* or impede* or delay* or constrain* or hindrance* or uptake or "take up" or increas* or decreas* or reduc* or impact* or effect* or improve* or enhance* or encourag* or support* or promot* or optimiz* or optimis* or adher* or access* or motivat* or accept* or satisfaction or compliance or comply or complie* or refus* or availabl* or provision* or provid* or offer or incentive* or utiliz* or utilis*).ti,ab. (11910955)

3 exp health behavior/ (100016)

4 exp risk reduction behavior/ (7539)

5 exp health promotion/ (55103)

6 exp primary prevention/ (114407)

7 exp preventive medicine/ (32402)

8 exp life style/ (65061)

9 exp food habits/ (21286)

10 exp food preferences/ (10111)

11 exp nutrition therapy/ (80646)

12 exp vision tests/ (80919)

13 exp hearing tests/ (38091)

14 exp smoking/ (124725)

15 exp smoking cessation/ (21110)

16 exp "tobacco use disorder"/ (8455)

17 exp "tobacco use cessation"/ (21814)

18 exp tobacco smoke pollution/ (10742)

19 exp alcohol drinking/ (53034)

20 exp alcohol deterrents/ (4205)

21 exp drinking behavior/ (58520)

22 exp temperance/ (2625)

23 exp loneliness/ (2175)

24 exp exercise/ (114811)

25 exp sports/ (113459)

26 exp exercise therapy/ (30184)

27 exp physical exertion/ (54932)

28 exp physical fitness/ (22421)

29 exp "physical education and training"/ (13706)

30 exp exercise test/ (51046)

31 exp walking/ (20209)

32 exp running/ (13881)

33 exp jogging/ (697)

34 exp bicycling/ (7886)

35 exp swimming/ (18935)

36 exp dancing/ (1843)

37 exp gardening/ (466)

38 exp fitness centers/ (338)

39 exp sedentary lifestyle/ (2551)

40 or/3-39 (992780)

41 2 and 40 (632416)

42 1 or 41 (939031)

43 meta-analysis as topic/ (14071)

44 meta-analys*.tw. (62370)

45 metaanaly*.tw. (1373)

46 Meta-Analysis/ (51199)

47 (systematic adj (review*1 or overview*1)).tw. (53469)

48 exp Review Literature as Topic/ (7628)

49 or/43-48 (125460)

50 Comment/ (577154)

51 Letter/ (829297)

52 Editorial/ (350296)

53 animal/ (5488706)

54 human/ (13639147)

55 53 not (53 and 54) (3959628)

56 ((middle adj age*) or (midlife* or "mid life*" or midlives or "mid lives") or (baby adj2 boomer*)).ti. (11512)

57 exp *Middle age/ (844)

58 49 or 50 or 51 or 52 or 55 (5339612)

59 56 or 57 (12088)

60 42 and 59 (2659)

61 60 not 58 (2560)

62 limit 61 to yr="2000 -Current" (1772)

63 exp Middle age/ (3344360)

64 56 or 63 (3346787)

65 42 and 64 (234019)

66 65 not 58 (230787)

67 limit 66 to yr="2000 -Current" (152412)

***************************

**3. Sample targeted search for primary studies (where no systematic reviews)**

Sample search: targeted vision and disadvantaged populations or adults IN TITLES using Ovid MEDLINE(R) In-Process & Other Non-Indexed Citations and Ovid MEDLINE(R) <1946 to Present>

Note: Searches terms were modified were necessary when searching other databases.

--------------------------------------------------------------------------------

1 adult*.ti,ab. (827307)

2 exp Young Adult/ (351950)

3 exp Adult/ (5611750)

4 or/1-3 (6029894)

5 "single parent*".ti. (219)

6 minorit*.ti. (5689)

7 "free school meal*".ti. (5)

8 ((low* or work*) adj4 class*).ti. (1279)

9 unemployed*.ti. (423)

10 (low* adj3 (income* or wage* or pay*)).ti. (5984)

11 ("income support*" or "housing benefit*" or "child support*" or "unemployment benefit*").ti. (138)

12 poverty.ti. (3044)

13 (deprive* or deprivation*).ti. (17067)

14 ethnic*.ti. (18691)

15 ((vulnerable or disadvantaged or "at risk" or "high risk" or "low socioeconomic status" or neglect* or affected or marginal* or forgotten or non-associative or nonassociative or unengaged or hidden or excluded or transient or inaccessible or underserved or stigma* or inequitable) and (people or population* or communit* or neighbourhood*1 or neighborhood*1 or group* or area*1 or demograph* or patient* or social*)).ti. (25665)

16 (immigrant* or migrant* or asylum or refugee* or undocumented).ti. (15142)

17 (born adj2 overseas).ti. (10)

18 (displaced and (people or person*1)).ti. (163)

19 (homeless or vagrant*).ti. (3367)

20 (((language* or communicat*) and (barrier* or understand* or strateg* or proficien*)) or translat* or interpret* or (cultur* and competen*)).ti. (62987)

21 (illiteracy or illiterate*).ti. (279)

22 (traveller*1 or Gypsies or Gypsy or Gipsy or Gipsies or Romany or Romanies or Romani or Romanis or Romani or Romanis or Roma).ti. (2929)

23 (homeless* or vagrant* or tramp or tramps or "street person" or "street people" or (sleep* adj3 rough)).ti. (4271)

24 (lesbian* or gay* or homosexual* or bisexual* or transgender* or trans-gender* or trans-sexual* or transsexual* or transexual* or "men who have sex with men" or "same-sex" or queer*).ti. (13886)

25 (shame* or stigma* or socioeconomic or socio-economic or prejudic*).ti. (18591)

26 or/5-25 (190725)

27 4 and 26 (70318)

28 ((middle adj age*) or (baby adj2 boomer*) or (midlife or "mid life" or midlives or "mid lives")).ti. (11505)

29 exp *Middle Aged/ (844)

30 28 or 29 (12081)

31 27 or 30 (82144)

32 ((eye or eyes or eyesight or sight* or vision* or visual* or hearing) adj3 (test* or check* or screen*)).ti,ab. (21305)

33 exp vision tests/ or exp hearing tests/ (118748)

34 32 or 33 (131886)

35 31 and 34 (458)

36 meta-analysis as topic/ (14071)

37 meta-analys*.tw. (62302)

38 metaanaly*.tw. (1372)

39 Meta-Analysis/ (51199)

40 (systematic adj (review*1 or overview*1)).tw. (53390)

41 exp Review Literature as Topic/ (7628)

42 or/36-41 (125334)

43 Comment/ (576830)

44 Letter/ (828887)

45 Editorial/ (350125)

46 animal/ (5488705)

47 human/ (13639146)

48 46 not (46 and 47) (3959628)

49 43 or 44 or 45 or 48 (5222217)

50 35 not 49 (427)

51 50 and 42 (2)

52 35 not 51 (456)

53 limit 52 to yr="2000 -Current" (274)
