# Supplementary material for: Barriers and Facilitators to the Uptake and Maintenance of Healthy Behaviours by People at Mid-Life: A Rapid Systematic Review
Source: PLoS One. 2016 Jan 27;11(1):e0145074. doi: 10.1371/journal.pone.0145074 (PMC4731386; doi:10.1371/journal.pone.0145074)
Supplement: S2 Text — (DOCX) [file pone.0145074.s006.docx]

**Websites Searched**

- NHS Evidence Search ([www.evidence.nhs.uk](http://www.evidence.nhs.uk))
- Open Grey ([www.opengrey.eu](http://www.opengrey.eu))
- Public Health Observatories ([www.apho.org.uk](http://www.apho.org.uk))
- Health Evidence Canada ([www.healthevidence.org](http://www.healthevidence.org))

Alzheimer’s Society ([www.alzheimers.org.uk](http://www.alzheimers.org.uk))

- RNIB ([www.fightforsight.org.uk](http://www.fightforsight.org.uk))
- Fight for Sight ([www.fightforsight.org.uk](http://www.fightforsight.org.uk))
- Action on Hearing Loss ([www.actiononhearingloss.org.uk](http://www.actiononhearingloss.org.uk))
- Beth Johnson Foundation ([www.bjf.org.uk](http://www.bjf.org.uk))
- British Library (<http://www.bl.uk>)
- Campbell Collaboration (<http://www.campbellcollaboration.org>)
- Department of Health (<https://www.gov.uk/government/publications>)
- E-Print Network (<http://www.osti.gov/eprints/>)
- Google Scholar (<http://scholar.google.co.uk>)
- Grey Literature Report (<http://www.greylit.org>)
- Lenus (<http://www.lenus.ie/hse/>)
- OAIster (<http://www.oclc.org>)
- Public Health Europe (<http://ec.europa.eu/health/index_en.htm>)
- RAND Health (<http://www.rand.org/health.html>)
- Scirus (<http://www.scirus.com>)
- World Health Organisation (<http://www.who.int/en/>)
