# Supplementary material for: Barriers and Facilitators to the Uptake and Maintenance of Healthy Behaviours by People at Mid-Life: A Rapid Systematic Review
Source: PLoS One. 2016 Jan 27;11(1):e0145074. doi: 10.1371/journal.pone.0145074 (PMC4731386; doi:10.1371/journal.pone.0145074)
Supplement: S3 Text — (DOCX) [file pone.0145074.s007.docx]

**PROTOCOL**

**REVIEW 1 – A review of the issues that prevent or limit the uptake and maintenance of healthy behaviours by people in mid-life (barriers and facilitators)**

Cambridge Institute of Public Health

20^th^ September 2013 (v2)

**Principal Investigator:**

Professor Carol Brayne

**Correspondence to:**

Dr Louise Lafortune

Institute of Public Health, Forvie Site
University of Cambridge School of Clinical Medicine
Box 113 Cambridge Biomedical Campus
Cambridge, CB2 0SP

[ll394@medschl.cam.ac.uk](mailto:ll394@medschl.cam.ac.uk)

1. Guidance title

Disability, dementia and frailty in later life - mid-life approaches to prevent or delay the onset of these conditions.

- 1. **Short title**

Disability, dementia and frailty in later life – mid-life approaches to prevention

- 1. **Review team**

| **Staff/resource description^a^** | **No. of days per staff/ resource** | **Review 1^b^** | **Email address** |
| --- | --- | --- | --- |
| Systematic Reviewer (Sarah Kelly) | 220 days (1 FTE) ^[[1]](#footnote-1)^  (1 Aug ’13 – 30 Nov ‘14) | 66 days  (1 Aug ’13 – 9 Jan 2014) | sak65@medschl.cam.ac.uk |
| Systematic Reviewer  (Steven Martin) | 55 days (0.25 FTE)  (1 Aug ’13 – 30 Nov ‘14) | 17 days  (1 Aug ’13 – 9 Jan 2014) | sm987@medschl.cam.ac.uk |
| Admin/Technical Support (Andy Cowan) | 110 days (0.5 FTE)  (Sept’13 – Feb ’14) | 33 days  (Sept ’13 – 9 Jan 2014) | ac798@medschl.cam.ac.uk |
| Scientific Coordinator / Project Manager  (Louise Lafortune) | 22 days (0.10 FTE)  (1 Aug ’13 – 30 Nov ‘14) | 7 days  (1 Aug ’13 – 9 Jan 2014) | ll394@medschl.cam.ac.uk |

^a^A third systematic reviewer (Dr Nadja Smailagic) is available to provide a third view on inclusion/exclusion of studies or data

^b^Likely division of time between reviews: review 1 (30%), review 2 (40%), review 3 (30%)

1. **Summary of the scope**

This review is the first of three to be conducted to inform the guidance on which primary prevention behaviours to be adopted in mid-life are most effective and cost-effective to prevent and delay the onset of dementia, frailty, disability and other non-communicable chronic conditions in later-life.

The full scope of the guidance is available in the final scope document (Final scope, NICE Public Health Guidance, Disability, dementia and frailty in later life – mid-life approaches to prevention) that incorporates stakeholder comments from a 4-week consultation 21 March to 18 April 2013. Further background information and details of the broad approach to the conduct of the three reviews to provide evidence to inform the guidance is detailed in the tender proposal submitted by Cambridge Institute of Public Health, 15^th^ July 2013 (invitation to tender reference DDER 42013). The key details of these documents have been incorporated into this protocol.

- 1. **Review title and main objectives**

This systematic review (**Review 1**) of the literature is entitled: A review of the key issues for people in midlife that prevent or limit or which help and motivate them to take up and maintain healthy lifestyle behaviours.

It will address the following question: What are the key issues for people in midlife that prevent or limit, or which help or motivate them to take up and maintain healthy lifestyle behaviours and to what extent do they have an effect?

- How does this differ for subpopulations, for example by ethnicity, socioeconomic status or gender?

The two other reviews (and study protocols) related to this one address the following questions:

- - **Review 2**: What behavioural factors in midlife are associated with successful ageing and the primary prevention or delay of dementia, non-communicable chronic conditions, frailty and disability? How strong are the associations and how does this vary for different subpopulations?
  - **Review 3**: What are the most effective and cost-effective midlife interventions for increasing the uptake and maintenance of healthy lifestyle behaviours? To what extent do the different health behaviours prevent or delay dementia? To what extent do the different health behaviours prevent or delay frailty and disability related to modifiable lifestyle risk factors? To what extent do the different health behaviours prevent or delay non-communicable chronic diseases?
  1. **Groups that will be covered**
- Adults aged 40-64 years, with a particular focus on people at increased risk of frailty, dementia, disability or other non-communicable chronic conditions due to health-related behaviour and lifestyle factors.
- Adults aged 39 and younger from disadvantaged populations (as they are at increased risk of ill health and more likely to develop multiple morbidities). Disadvantaged populations will include (but is not limited to) low socioeconomic status, ethnic minority groups, LGBT groups, travellers and other groups with protected characteristics under the equality and diversity legislation.
  1. **Groups that will not be covered**
- Adults with any type of dementia or pre-existing cognitive impairments.
- Adults who are receiving treatment for a chronic non-communicable condition or who have a disability associated with modifiable lifestyle risk factors will not be included for that particular condition or disability.
  1. **Activities**

**2.4.1 Activities that will be covered**

The key issues (barriers or facilitators) that prevent or limit the uptake or that help and motivate the uptake and maintenance of healthy lifestyle behaviours by people in midlife that may impact on the development and progression of: disability, dementia, frailty (including bone health) and common non-communicable chronic diseases. Examples of the latter include cardiovascular diseases, diabetes, chronic obstructive pulmonary disease, visual and hearing conditions and some cancers that may be associated with lifestyle.

The review will include both external factors and internal factors as outcomes. An example of an external factor could be access to resources; examples of internal factors could include peoples’ attitudes and expectations.

The scope includes (but is not limited to) barriers or facilitators to:

1. Uptake or maintenance of healthy lifestyle behaviours including less sedentary behaviour, increased physical activity, improved diet or components of diet (e.g. fat intake, fruit and vegetable intake), weight loss or control, cessation or reduction of smoking, reduction or modification of alcohol consumption, maintain sufficient levels of social activity and avoid loneliness (this may vary for individuals), to avoid excessive exposure to noise and address hearing and/or sight loss, or to improve/modify multiple behavioural risk factors and health behaviours in general.
2. Uptake or maintenance of healthy lifestyle behaviours at individual, family, community, subnational or national level (these may be targeted at specific groups, particularly those who are at increased risk, or who are from disadvantaged groups, or at healthcare professionals).
3. Uptake or maintenance of healthy lifestyle behaviours in a range of settings including primary and secondary care, and workplace and community settings in the private, public, voluntary or commercial sectors.

**2.4.2 Activities that will not be covered**

Barriers and facilitators to:

1. Use of drugs to prevent or treat dementia and non-communicable chronic conditions;
2. Use of dietary supplements;
3. Diagnosis and care of disability, dementia, frailty and common non-communicable chronic disease;
4. Management of existing disability, dementia, frailty and common non-communicable chronic disease.
5. Recreational drug use.
6. Management of obesity, including medical and surgical interventions for obesity.
7. Organisational interventions, policies and laws.

**2.4.3 Other aspects of the scope**

Interventions to promote lifestyle and/or behaviour change such as the primary prevention of overweight or obesity or hypertension or raised cholesterol are covered by the scope of the review and the guidance. However, interventions for the secondary prevention or management of such conditions or for people with existing dementia, disability, chronic disability or frailty are outside the scope of this review and the guidance.

1. **Reviews**

**3.1. Overview**

This review is the first of three to be conducted to inform the guidance on mid-life approaches to prevent or delay the onset of dementia, frailty or disability in later life.

This review (Review 1) aims to identify the key issues for people in midlife that prevent or limit or that help and motivate the uptake and maintenance of healthy behaviours using data from all available study types including qualitative and observational studies.

A conceptual overview of the 3 reviews is presented in Figure 1.

The model details health behavioural risk factors in midlife, interventions to improve or maintain healthy behaviours, intermediate biological risk factors that can be influenced by healthy behaviours and preventable outcomes relating to disability, frailty or dementia in later life. The model will be used to inform the searches and selection of studies for the review within the inclusion/exclusion criteria outlined above in section 2.

**Lifestyle behaviours**

- Physical activity / Sedentary behaviours
- Diet
- Tobacco smoking
- Alcohol consumption
- Cognitive activities
- Noise exposure
- Work / Social activities / Participation

**REVIEW 1**

**Uptake & maintenance of healthy behaviours in mid-life**

**Barriers**

**Facilitators**

- Personal factors (e.g. gender, SES, ethnicity, employment, family, previous experiences, expectations)
- Social factors (e.g. norms, support)
- Environmental factors (e.g. access to resources/interventions; residential & work environment)
- Organisational factors (e.g. design & delivery of intervention, resources)

**Effect on ageing Well Outcomes**

- Disability (ADL, IALD, independence, mobility)
- Dementia
- Frailty
- Healthy life span
- Quality of life
- Participation

**Effect on non-Communicable diseases**

- Cardiovascular diseases& stroke
- Renal disease
- Life style related cancers
- COPD
- Type II diabetes
- Osteoporosis / Bone health
- Hearing & Sight Loss

**Primary prevention of preconditions**

- Impaired glucose intolerance
- High blood pressure
- High cholesterol
- Overweight / Obesity (weight loss or control)
- Impaired cognitive function (MCI)
- Mood disorders
- Functional limitations

**Other relevant outcomes**

- Resource use, costs, cost effectiveness

**Effect on lifestyle behaviours**

- Increase/maintain “good” levels of physical activity OR decrease sedentary life styles OR maintain balance, strength and weight-bearing functions
- Improve/maintain good diet & nutrition
- Reduce/prevent/stop tobacco consumption
- Decrease/prevent excessive alcohol consumption
- Maintain/increase cognitive and social activities, and participation
- Prevent / decrease excessive noise/ sun exposure
- Improve/modify multiple behavioural risk factors
- Remove barriers / facilitate uptake & maintenance of any lifestyle behaviours WITH demonstration of impact.

**Intervention**

Effectiveness & cost effectiveness…

**REVIEW 2**

Association between lifestyle behaviours and ageing well Outcomes & NCD

**REVIEW 3**

**Figure 1.**

**3.2 Review question**

**Review question 1 (from a series of 3 reviews)**

What are the key issues for people in midlife that prevent or limit the uptake and maintenance of healthy behaviours (barriers and facilitators) and to what extent do they have an effect?

- How does this differ for subpopulations, for example by ethnicity, socioeconomic status or gender?

**3.3 Review outcomes**

The key issues for people in midlife that prevent or limit or which help and motivate them to take up and maintain healthy lifestyle behaviours (barriers or facilitators).

Both qualitative outcomes on barriers or facilitators to the type, level or amount of healthy behaviour and quantitative outcomes that include associations of any barrier or facilitator with the type, level and amount of healthy behaviour and will be included.

The review will include both internal and external factors as outcomes, including for those delivering and receiving interventions. Outcomes would include:

# Personal factors: e.g. gender, SES, ethnicity, employment, family, previous experiences, expectations

- Social factors: e.g. social norms, support
- Environmental factors: e.g. access to resources/interventions, residential and work environment
- Organisational factors: e.g. design and delivery of interventions/ resources,

**4. Methods**

**4.1 Inclusion criteria – types of studies**

The evidence base for this review is likely to be so large that resource limitations make it difficult to cover all primary studies within the available timescales (Chapter 3 of CPHE methods manual) so it has been agreed with the CPH project team that systematic review level evidence should be the first tier of evidence to be included in the review. Following the methods in Appendix J, CPHE methods manual, priority will be given to high quality systematic reviews, primary data published since the publication of these reviews (if available) and high quality primary data where reviews are not available.

All types of studies will be searched for including systematic reviews (of all types of studies), and primary observational and qualitative studies.

Qualitative studies will provide information on the key issues for people in mid-life, but will not provide any information about the extent of any effect.

Quantitative studies will provide answers to the question about the extent to which issues have an effect and also information on the key issues for people in midlife. While primary cross-sectional studies will not be excluded from the searches, they will be excluded from the review as they would only show a cross-sectional association.

Where no up to date systematic review evidence is available to cover a topic or area of interest, primary empirical research derived from quantitative, qualitative or mixed methods approaches or process evaluations will be sought.

The three evidence reviews will be developed in parallel and in line with the 2012 editions of the [CPHE methods manual](http://publications.nice.org.uk/methods-for-the-development-of-nice-public-health-guidance-third-edition-pmg4) and the [CPHE process manual](http://publications.nice.org.uk/the-nice-public-health-guidance-development-process-third-edition-pmg5); and build on other methodological resources available through experts groups (such as the Cochrane and Campbell Collaborations)^[[2]](#footnote-2)^, the team expertise and an extended network of collaborators.

**4.2 Inclusion criteria – Dates of studies to be included**

Systematic reviews and primary studies published from year 2000 onwards.

**4.3 Inclusion criteria – Qualitative studies**

Population**:** Adults at midlife (aged 40 to 64 years for the general population) with a particular focus on people at increased risk of the target conditions (to be defined) and adults in disadvantaged populations aged 18-39. Disadvantaged populations would include low socioeconomic status and ethnic minority groups. Studies would not be excluded on basis of country of origin, however studies conducted in the UK will be prioritised in the synthesis of data.

Exposure**:** Barriers or facilitators to the uptake of healthy lifestyle behaviours in midlife (defined as above). Healthy lifestyle behaviours include (but are not limited to) increase/maintain physical activity or decrease sedentary lifestyles; improve/maintain good diet and nutrition; smoking cessation or reduction or prevention of smoking; decrease/moderate alcohol consumption or prevent excessive consumption; improve/modify multiple behavioural risk factors; increase/maintain social activity or prevent loneliness; increase or maintain/address management of sight loss or hearing loss, body weight, avoid excessive noise.

Outcomes: Any qualitative data on barriers or facilitator to the uptake or maintenance of healthy behaviours (as defined above).

Timescale: No lower time limit for study duration.

Language: English language studies only.

**4.4 Inclusion criteria - observational studies**

For the purposes of this review, longitudinal observational studies to be included are: cohort, case-control, population, ecological studies or surveys. Cross-sectional studies will be excluded as they would only show a cross-sectional association. Any cross-sectional analyses in the other observational study types would also be excluded.

Population: Adults at midlife (aged 40 to 64 years for the general population) with a particular focus on people at increased risk of the target conditions (to be defined) and adults in disadvantaged populations aged 18-39. Disadvantaged populations would include low socioeconomic status and ethnic minority groups. Studies would not be excluded on basis of country of origin, however studies conducted in the UK will be prioritised in the synthesis of data.

Exposure: Barriers or facilitators to the uptake of healthy lifestyle behaviours in midlife (defined as above). Healthy lifestyle behaviours include (but are not limited to) increase/maintain physical activity or decrease sedentary lifestyles; improve/maintain good diet and nutrition; smoking cessation or reduction or prevention of smoking; decrease/moderate alcohol consumption or prevent excessive consumption; improve/modify multiple behavioural risk factors; increase/maintain social activity or prevent loneliness; increase or maintain/address management of sight loss or hearing loss, body weight.

Outcomes: Any measurable barrier or facilitator to the uptake or maintenance of healthy behaviours, including quantitative data on effect size.

Timescale: No limits to length of exposure

Language: English language studies only.

**4.6 Inclusion criteria – systematic reviews**

Systematic reviews of the study types detailed above will be included if they answer the review question and are of sufficient quality (see section 7). The process for using review level material is described in more detail in section 6.

Where there are existing systematic reviews published from 2000 onwards that cover all or part of a topic area, if the systematic review was published more than 5 years ago, the evidence would be updated using primary evidence published since. Where systematic reviews are unavailable, then primary evidence sources will be used as detailed in sections 4.3 to 4.4.

**5. Searching**

To develop the search strategies, an iterative approach will be taken involving the whole team, which consists of the following steps^[[3]](#footnote-3)^:

1. Initial team discussions around research questions (this process start at the same time as the development of specific and targeted review questions (PICO format);
2. Initial drafting of search building at least (but not exclusively) on the final scope for this guidance, comments received from key stakeholders on the draft scope, high quality peer-review systematic reviews (when available) on same or similar topics for each key domains of the strategy, (e.g. health, preventative interventions, behaviours, etc.);
3. Testing of individual components and development of the review specific strategies in key databases;
4. Refining of specific review strategies upon discussion with information specialist;
5. Updating of search strategies based on reviewers comments;
6. Adaptation of strategies to individual databases (i.e. Mesh terms or filters in one database don’t usually apply to other databases);
7. Running of search and uploading of references in individual Endnote data bases (for specified time period, i.e. since 2000);
8. Create a combined Endnote database (master file); delete duplicate and prepare for title screening;
9. Identification of potential included studies; selection of full text for further assessment; identification of included and excluded studies.

A structured search strategy will be developed and conducted for this review (see Appendix B for draft Medline Ovid search with number of hits for key term combinations). It is envisaged that the searches for reviews 1 and 3 of this series can be combined as both reviews focus on issues and interventions that prevent or limit the uptake or maintenance of healthy behaviours in midlife. Screening of search hits may be conducted for both reviews together or separately. Initial test screening will determine if it is feasible to screen search hits for both reviews at the same time.

As initial searches suggest a large volume of search hits, searching will be conducted in two stages: - 1) searching for systematic reviews using a systematic review filter agreed with CPH). 2) Where there are no systematic reviews covering a topic or area, targeted searches will then be conducted for primary studies as appropriate.

For stage 1, searching for systematic reviews the following databases (from the full list below) will be searched: Cochrane Database of Systematic Reviews, Medline, Embase, Database of Abstracts of Reviews of Effectiveness, HTA database, ASSIA, Sociological Abstracts and the websites listed below.

Databases to be searched (with host platform):

CINAHL (EBSCO host)

Cochrane Central Register of Controlled Trials ([www.thecochranelibrary.com](http://www.thecochranelibrary.com))

Cochrane Database of Systematic reviews ([www.thecochranelibrary.com](http://www.thecochranelibrary.com))

Database of Abstracts of Reviews of Effectiveness ([www.thecochranelibrary.com](http://www.thecochranelibrary.com))

HTA database ([www.thecochranelibrary.com](http://www.thecochranelibrary.com))

NHS EED database ([www.thecochranelibrary.com](http://www.thecochranelibrary.com))

EMBASE (Ovid)

Health Management Information Consortium (Ovid)

MEDLINE (including MEDLINE – in-process) (Ovid)

PsycINFO (Ovid)

ASSIA (Proquest)

Sociological Abstracts (Proquest)

Campbell Collaboration

Websites to be searched:

NHS Evidence Search [www.evidence.nhs.uk](http://www.evidence.nhs.uk)

Open Grey [www.opengrey.eu](http://www.opengrey.eu)

Public Health Observatories [www.apho.org.uk](http://www.apho.org.uk)

Health Evidence Canada [www.healthevidence.org](http://www.healthevidence.org)

Alzheimer’s Society [www.alzheimers.org.uk](http://www.alzheimers.org.uk)

RNIB [www.rnib.org.uk](http://www.rnib.org.uk)

Fight for Sight [www.fightforsight.org.uk](http://www.fightforsight.org.uk)

Action on hearing loss [www.actiononhearingloss.org.uk](http://www.actiononhearingloss.org.uk)

Beth Johnson Foundation [www.bjf.org.uk](http://www.bjf.org.uk)

A full search protocol (Chapter 4 CPHE methods manual) will be developed.

Records retrieved from the searches will be reported as in Appendix C, NICE methods manual.

**6.Identification &selection of relevant studies (Section 5.2 NICE methods manual)**

Titles and/or abstracts will be screened independently by 2 reviewers using the inclusion criteria detailed in the review protocol. Differences between reviewer’s results will be resolved by discussion and when necessary in consultation with a third reviewer. If after discussion, there is still doubt about a study’s relevance for the review it will be retained.

Full paper copies will be obtained for all reviews and studies identified by the title/abstract screening. A full paper screening tool with inclusion/exclusion criteria as defined in the review protocol will be developed for screening of the full papers. Full paper screening will be conducted independently by two people. Any differences of opinion about inclusion/exclusion will be resolved by discussion between the two reviewers or by consultation with a third reviewer.

Additionally, for systematic reviews, the process for using review level material as described in Appendix J of the CPHE methods manual will be followed. Systematic reviews will initially be screened using the review screening form in Appendix J of the CPHE methods manual to determine if the review is relevant to the guidance topic. Adaptations to the form will be jointly agreed with the CPH project team. If the review does not meet all the criteria in the full review screening form it may still be useful as a source of references but it would not be relied upon on its own to address a research question.

Additionally, experts in the field will be consulted to identify any further potentially relevant papers through contacts and links with the Institute of Public Health at University of Cambridge. This will include consultation with the Behaviour and Health Research Unit at the University of Cambridge, led by Professor Theresa Marteau. Also, responses to the NICE call for evidence relating to this guidance conducted between 31/5/2013 and 28/6/2013 will be screened for potential inclusion in the review.

A flow chart will be used to summarise the number of papers included and excluded at each stage of the process. Reviews or primary studies excluded at the full paper screening stage will be listed in the appendix of the review along with the reason for exclusion.

**7. Quality Assessment**

Systematic reviews

If a systematic review passes all the criteria in the CPHE full review screening form (see section 3.6, CPHE manual) the methodological quality of each review will be assessed using five priority criteria of the AMSTAR tool i.e. 1) was a comprehensive literature search performed?, 2) were the characteristics of the included studies provided?, 3) was the scientific quality of the included studies assessed and documented?, 4) was the scientific quality of the included studies used appropriately in formulating conclusions?, 5) was the method used to combine the findings of studies appropriate?. Each full review will be assessed by one reviewer and checked for accuracy by another. A minimum of 10% of the studies will be fully double assessed. Any discrepancy between reviewers would be resolved by discussion.

The quality of the evidence presented in the included systematic reviews would not be reassessed by the authors. The results would be extracted into the evidence tables and narrative summary and a narrative summary would be provided of the highest rated evidence presented in each of the systematic reviews, where this is different from the overall summary of the evidence provided in the review. This would be possible because all the included reviews would assess the quality of their primary studies (meeting AMSTAR criterion 7).

Other study designs

Study designs will be assigned using the glossary of study designs (appendix D, CPHE methods manual) and the algorithm for classifying experimental/interventional and observational study designs (appendix E, CPHE methods manual).

When a study design has been assigned, quality appraisal of studies will be assigned using the relevant quality appraisal checklist in the NICE methods manual (Appendices F, G, H, I; CPHE methods manual).

Each full paper will be assessed by one reviewer and checked for accuracy by another. A minimum of 10% of the studies will be fully double assessed.

The composite inter-rater reliability studies will be reported as a kappa statistic noting if it is good (0.60 to 0.74) or excellent (above 0.75). If the inter-rater reliability score is below 0.60, the reasons for digression would be explored and a course of action agreed.

**8. Data extraction**

We will extract data on participant, intervention or exposure (and comparators), study and health outcome characteristics. To ensure accurate reporting the data extraction pro-forma will be piloted against two included papers. Each included full paper will be assessed by one reviewer and checked for accuracy by another. A minimum of 10% of the studies will be fully double extracted (as above for quality assessment).

**9.Synthesis of evidence**

As both qualitative and quantitative evidence will be included in this review, findings will be narratively synthesised and presented to inform guidance. Data specific to health inequalities and vulnerable communities will be assessed and findings may be summarised separately if sufficient data is available. Studies conducted in the UK would be prioritised in the synthesis of data.

Information about included studies will be presented in both narrative and evidence table sections of the review, and in sufficient detail, to ensure clear and transparent links between recommendations and evidence (Section 5, Appendix K, CPH methods manual).

Narrative summaries of both quantitative and qualitative studies or systematic reviews will be written as outlined in section 5.4.2. CPHE methods manual. The data about barriers and facilitators to be incorporated into this review is unlikely to be in a format suitable for meta-analysis. Key themes based on analysis of the evidence tables across each topic area from both the quantitative and qualitative data will be synthesised in a narrative format (where sufficient data is available to identify themes). Otherwise, a descriptive approach to the available evidence will be taken (see section 5.4.5 CPHE methods manual).

For each key question or issue an evidence statement will be generated which will provide an aggregated summary of all of the relevant studies (Sections 5.5.1 to 5.5.5 CPHE methods manual). Applicability ratings will be used to assess each evidence statement to judge how similar the population(s), setting(s), intervention(s) and outcome(s) of the included studies are to those outlined in the review question (Section 5.6 CPHE methods manual). Each evidence statement will be assessed as ‘directly applicable, partially applicable or not applicable’.

**10.Project management**

The proposed timelines for each review (Appendix C, CPHE methods manual) are aligned with the project timelines (and the PHAC committee meetings) set forth by NICE in appendix B of the invitation to tender.

**10.1 Quality assurance**

We will adhere to the NICE process and comply with the highest standard of quality.

**10.2 Data Protection and Freedom of Information Acts**

As full time researchers at the University of Cambridge everything we do falls under the Data Protection and Freedom of Information Acts. We will keep a record of all activities pertaining to this project and comply to NICE’s request shall the situation arise.

This project does not involve collecting nor analysing personal data - the main source of information(i.e. scientific evidence) is already in the public domain. All project specific correspondence, documents, etc. will be available to the NICE project team. The CPHE Methods Manual (namely chapters 3 through 7) lists the key steps of the evidence review and the project information will be organised accordingly.

Date and version of the protocol: 20^th^ Sept 2013, version 2.

**Appendix A – Draft project timescale**

| Task | Review 1 | Review 2 | Review 3 |
| --- | --- | --- | --- |
| Submission of draft evidence review to NICE team | 7 November 2013 | 19 December 2013 | 6 February 2014 |
| NICE provide comments on draft review | 15 November 2013 | 9 January 2014 | 14 February 2014 |
| Submission of revised draft review to NICE | 2 December 2013 | 22 January 2014 | 3 March 2014 |
| Draft review mailed to PHAC members | 6 December 2013 | 24 January 2014 | 6 March 2014 |
| Submission of final slides for presentation of review to PHAC | 6 December 2013 | 24 January 2014 | 6 March 2014 |
| Presentation of draft review at PHAC meeting | 18 December 2013 | 5 February 2014 | 19 March 2014 |
| Final amendments to be made to report post PHAC meeting | 9 January 2014 | 20 February 2014 | 3 April 2014 |

**Appendix B – Ovid Search strategy**

Database: Ovid MEDLINE(R) In-Process & Other Non-Indexed Citations and Ovid MEDLINE(R) <1946 to Present>

Search Strategy:

--------------------------------------------------------------------------------

1 (prevent* or barrier* or facilitat* or hinder* or block* or obstacle* or restrict* or restrain* or obstruct* or inhibit* or impede* or delay* or constrain* or hindrance* or uptake or "take up" or increas* or decreas* or reduc* or impact* or effect* or improve* or enhance* or encourag* or support* or promot* or optimiz* or optimis* or adher* or access* or motivat* or accept* or satisfaction or compliance or comply or complie* or refus* or availabl* or provision* or provid* or offer or incentive* or utiliz* or utilis*).ti,ab. (11812295)

2 (

(((behavio?r or lifestyle or "life style") adj3 (change* or changing or modification or modify or modifying or therapy or therapies or program* or intervention* or counsel*))

or (health* adj3 (behavior* or behaviour*))

or ((ageing or aging) adj3 (well or success* or positive* or active* or healthy))

or (food* adj3 choice*)

or dieting

or (diet* adj3 (health* or balance* or fat* or salt* or sugar* or mediterranean or choice* or improv* or unhealthy))

or ((fruit* or vegetable* or salt* or fat* or sugar*) adj3 (intake* or consum* or eat* or ate))

or (undernutrition or undernourish* or under-nutrition* or under-nourish*)

or (multimicronutrient* or multi-micronutrient* or micronutrient* or micro-nutrient* or multinutrient* or multi-nutrient*)

or ("five a day" or "5 a day")

or ("health check" or "check-up")

or "health MOT*"

or ((eye* or sight* or vision* or visual* or hearing) adj3 (test* or check* or screen*))

or (smok* or tobacco or cigar* or nicotine)

or ((alcohol* or drunk* or drink*) adj3 (consum* or misuse* or abuse* or intoxicat* or harmful or excess* or binge* or hazardous* or heavy or temperance or abstinence))

ortemperan* or teetotal* or (lonely or lonli*)

or (socialis* or socializ*)

or (social* adj3 (isolat* or network* or contac* or alien*))

or (cognitive adj2 stimulat*)

or ((physcial* or keep* or cardio* or aerobic or fitness) adj3 (fit* or activ* or train*))

or (sedentary or exercis* or sport*)

or "physical condition*"

or (balance* and (exercis* or retrain* or re-train* or reeducat* or re-educat*))

orinactiv*

or (walk* or run* or jog* or swim* or danc* or garden* or cycl* or bicycl* or bike* or recreation*)

or ("resistance training" or "acquaticexercis*" or "wellness centre*" or "wellness center*")

or ((phsyical* or game* or leisure* or fitness) adj5 (event* or setting* or sector* or program*

or venue* or site* or center* or centre*))

or ("weight gain*" or "weight los*" or "overweight" or "over weight")

or (obesity and "related behavio*")

or (overeat* or "over eat")

or ((waist* adj3 (circumference* or measur*))

or ((bmi or "body mass index") adj3 (gain* or loss* or lose* or lost or change*)))

or ((weight adj2 (cycling or reduc* or los* or maint* or decreas* or increas* or watch* or control*)) or "weight change*"))

adj3 (prevent* or barrier* or facilitat* or hinder* or block* or obstacle* or restrict* or restrain* or obstruct* or inhibit* or impede* or delay* or constrain* or hindrance* or uptake or "take up" or increas* or decreas* or reduc* or impact* or effect* or improve* or enhance* or encourag* or support* or promot* or optimiz* or optimis* or adher* or access* or motivat* or accept* or satisfaction or compliance or comply or complie* or refus* or availabl* or provision* or provid* or offer or incentive* or utiliz* or utilis*)).ti,ab. (422282)

3 exp health behavior/ (99406)

4 exp risk reduction behavior/ (7429)

5 exp health promotion/ (54839)

6 exp primary prevention/ (114054)

7 exp preventive medicine/ (32336)

8 exp life style/ (64645)

9 exp food habits/ (21115)

10 exp food preferences/ (10035)

11 exp nutrition therapy/ (80294)

12 exp vision tests/ (80635)

13 exp hearing tests/ (37970)

14 exp smoking/ (124015)

15 exp smoking cessation/ (20976)

16 exp "tobacco use disorder"/ (8399)

17 exp "tobacco use cessation"/ (21675)

18 exp tobacco smoke pollution/ (10679)

19 exp alcohol drinking/ (52735)

20 exp alcohol deterrents/ (4190)

21 exp drinking behavior/ (58207)

22 exp temperance/ (2609)

23 exp loneliness/ (2167)

24 exp exercise/ (111288)

25 exp sports/ (110890)

26 exp exercise therapy/ (29819)

27 exp physical exertion/ (52630)

28 exp physical fitness/ (21873)

29 exp "physical education and training"/ (13326)

30 exp exercise test/ (50193)

31 exp walking/ (19952)

32 exp running/ (13374)

33 exp jogging/ (690)

34 exp bicycling/ (7564)

35 exp swimming/ (18600)

36 exp dancing/ (1824)

37 exp gardening/ (462)

38 exp fitness centers/ (336)

39 exp sedentary lifestyle/ (2461)

40 or/3-39 (982650)

41 1 and 40 (624194)

42 2 or 41 (918445)

43 meta-analysis as topic/ (14016)

44 meta-analys*.tw. (61219)

45 metaanaly*.tw. (1363)

46 Meta-Analysis/ (50578)

47 (systematic adj (review*1 or overview*1)).tw. (52451)

48 exp Review Literature as Topic/ (7590)

49 or/43-48 (123563)

50 Comment/ (569825)

51 Letter/ (823159)

52 Editorial/ (346802)

53 animal/ (5460677)

54 human/ (13571801)

55 53 not (53 and 54) (3939518)

56 50 or 51 or 52 or 55 (5191291)

57 49 not 56 (114934)

58 exp middle age/ (3327213)

59 (middle adj age*).ti,ab. (33535)

60 (baby adj2 boomer*).ti,ab. (755)

61 (midlife or "mid life" or midlives or "mid lives").ti,ab. (3812)

62 or/58-61 (3339389)

63 adult*.ti,ab. (820161)

64 exp Young Adult/ (345598)

65 exp Adult/ (5579820)

66 or/63-65 (5993789)

67 disadvantag*.ti,ab. (49721)

68 "single parent*".ti,ab. (1847)

69 minorit*.ti,ab. (44419)

70 "free school meal*".ti,ab. (51)

71 ((low* or work*) adj4 class*).ti,ab. (18284)

72 unemployed*.ti,ab. (5350)

73 (low* adj3 (income* or wage* or pay*)).ti,ab. (28823)

74 ("income support*" or "housing benefit*" or "child support*" or "unemployment benefit*").ti,ab. (537)

75 poverty.ti,ab. (15354)

76 (deprive* or deprivation*).ti,ab. (63346)

77 ethnic*.ti,ab. (88554)

78 ((vulnerable or disadvantaged or "at risk" or "high risk" or "low socioeconomic status" or neglect* or affected or marginal* or forgotten or non-associative or nonassociative or unengaged or hidden or excluded or transient or inaccessible or underserved or stigma* or inequitable) and (people or population* or communit* or neighbourhood*1 or neighborhood*1 or group* or area*1 or demograph* or patient* or social*)).ti,ab. (740215)

79 (immigrant* or migrant* or asylum or refugee* or undocumented).ti,ab. (33459)

80 (born adj2 overseas).ti,ab. (217)

81 (displaced and (people or person*1)).ti,ab. (904)

82 (homeless or vagrant*).ti,ab. (5617)

83 (((language* or communicat*) and (barrier* or understand* or strateg* or proficien*)) or translat* or interpret* or (cultur* and competen*)).ti,ab. (527061)

84 (illiteracy or illiterate*).ti,ab. (3621)

85 (traveller*1 or Gypsies or Gypsy or Gipsy or Gipsies or Romany or Romanies or Romani or Romanis or Romani or Romanis or Roma).ti,ab. (6113)

86 exp Poverty/ (31438)

87 exp Ethnic Groups/ or exp Minority Groups/ (121433)

88 exp Unemployment/ (5168)

89 exp Single Parent/ (1044)

90 exp Homeless Persons/ (6489)

91 (homeless* or vagrant* or tramp or tramps or "street person" or "street people" or (sleep* adj3 rough)).ti,ab. (7737)

92 exp "Emigration and Immigration"/ (23559)

93 exp "Emigrants and Immigrants"/ (5643)

94 exp refugees/ (6697)

95 exp Language/ (161133)

96 exp Communication Barriers/ (4818)

97 Language/ (27562)

98 exp gypsies/ (617)

99 exp bisexuality/ or exp homosexuality/ or exp homosexuality, female/ or exp homosexuality, male/ (22600)

100 exp Transgendered Persons/ (103)

101 (lesbian* or gay* or homosexual* or bisexual* or transgender* or trans-gender* or trans-sexual* or transsexual* or transexual* or "men who have sex with men" or "same-sex" or queer*).ti,ab. (29651)

102 expTranssexualism/ (2855)

103 exp Poverty Areas/ (4608)

104 expVulnerable populations/ (5723)

105 exp Social Stigma/ (1349)

106 exp shame/ (1506)

107 exp Prejudice/ (23558)

108 exp Socioeconomic Factors/ (341937)

109 or/67-108 (2014972)

110 66 and 109 (804874)

111 62 or 110 (3668298)

112 42 and 57 and 111 (2749)

113 112 (2749)

**114 limit 113 to yr="2000 -Current" (2532)**

115 from 1 keep 1 (1)

116 42 and 111 (267995)

117 116 not 112 (265246)

118 117 (265246)

**119 limit 118 to yr="2000 -Current" (177531)**

**Appendix D – Timelines review 1: A review of factors in midlife that prevent or limit the uptake and maintenance of healthy behaviours**

| **Project Activity** | **Actions** | **2013** | | | | | | **2014** | | | | | | | | | | |
| --- | --- | --- | --- | --- | --- | --- | --- | --- | --- | --- | --- | --- | --- | --- | --- | --- | --- | --- |
|  |  | **Jul** | **Aug** | **Sep** | **Oct** | **Nov** | **Dec** | **Jan** | **Feb** | **Mar** | **Apr** | **May** | **Jun** | **Jul** | **Aug** | **Sep** | **Oct** | **Nov** |
| **Protocol** | Initial discussions with the NICE team |  |  |  |  |  |  |  |  |  |  |  |  |  |  |  |  |  |
|  | Feedback from team incorporated into protocol & finalised |  |  |  |  |  |  |  |  |  |  |  |  |  |  |  |  |  |
|  | **Allocation of roles and responsibilities** |  |  |  |  |  |  |  |  |  |  |  |  |  |  |  |  |  |
| **Review** | Development of search strategy |  |  |  |  |  |  |  |  |  |  |  |  |  |  |  |  |  |
|  | Databases search & reference retrieval |  |  |  |  |  |  |  |  |  |  |  |  |  |  |  |  |  |
|  | Quality Assessment |  |  |  |  |  |  |  |  |  |  |  |  |  |  |  |  |  |
|  | Data Extraction |  |  |  |  |  |  |  |  |  |  |  |  |  |  |  |  |  |
|  | Missing information/data followed up |  |  |  |  |  |  |  |  |  |  |  |  |  |  |  |  |  |
|  | Analysis |  |  |  |  |  |  |  |  |  |  |  |  |  |  |  |  |  |
|  | Writing of report |  |  |  |  |  |  |  |  |  |  |  |  |  |  |  |  |  |
|  | Submission of draft evidence review to NICE team |  |  |  |  |  |  |  |  |  |  |  |  |  |  |  |  |  |
|  | Feedback from NICE team |  |  |  |  |  |  |  |  |  |  |  |  |  |  |  |  |  |
|  | Final amendments |  |  |  |  |  |  |  |  |  |  |  |  |  |  |  |  |  |
|  | Provide support for NICE guidance |  |  |  |  |  |  |  |  |  |  |  |  |  |  |  |  |  |

1. On the basis of 1650 working hours a year or 220 days at 7.5 hours a day. [↑](#footnote-ref-1)
2. For example, the *Methodological Standards for the conduct of new Cochrane Intervention Reviews* and the *Guidelines for Systematic Reviews of Health Promotion and Public Health Interventions*. [↑](#footnote-ref-2)
3. Although the 3 reviews will stand on their own, the initial phase of the search strategy will look at the whole project to make sure the scope of the guidance is covered, to avoid unnecessary overlap in the retrieved literature and to gain in efficiency. [↑](#footnote-ref-3)
